# Electrochemically Driven Regioselective Radical Functionalization of Allenes: Scope and Mechanistic Insights

**DOI:** 10.1002/advs.76939

**Published:** 2026-08-17

**Authors:** Pranjal Pyne, Prateek Saini, Rohel Hoque, Shivam Pandey, Srinivasan Ramakrishnan, Chandra M. R. Volla

**Affiliations:** ^1^ Department of Chemistry Indian Institute of Technology Bombay, Powai Mumbai India

**Keywords:** allenes, electrochemical, intramolecular, oxazolines, radical cyclization

## Abstract

Despite their rich reactivity and unique cumulated *π*‐system, allenes remain largely untapped platforms in electrochemical radical functionalizations. Herein, we report a regioselective electrochemical protocol that merges radical addition with anodic oxidative cyclization, enabling the construction of oxazoline and oxazolidine‐2‐imine frameworks under mild and sustainable conditions. Detailed mechanistic investigations along with DFT computational studies were carried out to probe the reaction pathway. These studies suggested selective radical addition at the central allenic carbon, forming an allyl radical intermediate that undergoes anodic oxidation and intramolecular nucleophilic cyclization, enabling efficient access to functionalized heterocyclic derivatives without external oxidants. This work unveils a new paradigm for electrochemically driven allene functionalization, expanding the scope of radical‐mediated electroorganic synthesis toward biologically relevant moieties.

## Graphical abstract

Allenes emerge as powerful platforms for electrochemical functionalization through radical pathways. Selective radical addition at the central carbon triggers anodic oxidation and intramolecular cyclization, enabling efficient synthesis of oxazolines and oxazolidine‐2‐imines without external oxidants. Combined mechanistic investigations and DFT calculations reveal a new strategy for electrochemically driven heterocycle construction.

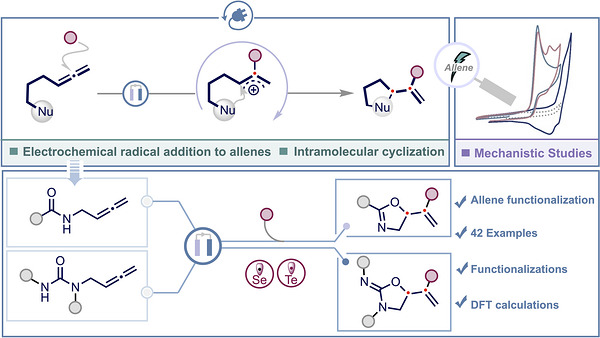

## 1 Introduction

**SCHEME 1:**
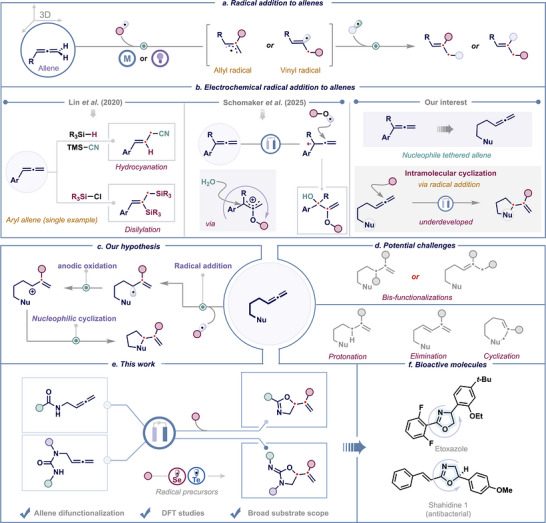
Overview of the work.

Allenes, with their cumulated diene framework and orthogonal *π*‐system, exhibit distinctive reactivity enabling the construction of molecular architectures that are elusive to traditional olefin or alkyne chemistry [1, 2, 3, 4, 5]. Consequently, allenes have been employed as versatile reaction partners in a variety of transformations including, transition metal‐catalyzed functionalizations [6, 7, 8, 9], nucleophilic [10, 11, 12, 13, 14], electrophilic [15], and radical addition reactions [16, 17, 18, 19, 20, 21]. Among these, radical additions to allenes are particularly attractive owing to their intriguing reactivity pattern and complex mechanistic pathways. Achieving regioselective radical additions to allenes remains challenging due to the presence of three reactive sites: addition at the central carbon leads to the formation of an allylic radical, whereas addition at terminal carbons generates vinyl radicals (Scheme 1a) [16, 17, 18]. This intrinsic competition often results in poor regio‐ and stereoselectivity and undesired byproducts, making predictable control of radical transformations at allenic centers difficult. To address these issues, significant efforts have been devoted toward metal‐catalyzed radical additions to allenes exploiting its unique reactivity [19, 20, 21, 22, 23, 24, 25, 26, 27]. More recently, with the growing emphasis on sustainability, photocatalytic radical addition to allenes have also been explored [19, 20, 21, 28, 29, 30, 31]. Despite these advances, electrochemical strategies for radical addition to allenes remain comparatively underexplored.

Electrochemistry offers precise control over redox events and avoids the need for stoichiometric oxidants or reductants, providing an inherently green and tunable platform toward organic synthesis [32, 33, 34, 35, 36, 37, 38, 39, 40, 41, 42, 43, 44, 45, 46, 47, 48, 49, 50, 51, 52, 53, 54, 55, 56, 57]. These advantages have begun to reshape allene chemistry, with recent electrochemical approaches enabling efficient C–H functionalizations [58, 59, 60, 61, 62, 63] and cyclization processes [64] involving allenic derivatives. Nevertheless, the potential of selective electrochemical functionalization of allenes through radical addition remains largely untapped. A few recent studies in this direction represent important initial progress. For example, Lin and co‐workers in 2020 showcased elegant electrochemical hydrocyanation [65] and disilylation [66] reactions proceeding via radical intermediates (Scheme 1b left). However, these studies were largely demonstrated with alkenes, with only a single example of allene reactivity in each case. Ye and co‐workers [67] later reported electrochemical hydrocarboxylation of allenes via CO_2_ reduction leading to the mixture of *α*‐ and *γ*‐carboxylated olefins. More recently, Schomaker group [68] disclosed the first general electrochemical allene dioxygenation via TEMPO trapping (Scheme 1b middle). Their mechanistic studies elegantly revealed the dual role of TEMPO as both a redox mediator and a radical trap. However, the method is largely confined to dioxygenation, leaving the broader scope of radical difunctionalization or heteroatom incorporation largely unexplored. Consequently, the potential of electrochemically driven selective radical additions to allenes, particularly beyond oxygenation pathways remains an area of active investigation. Motivated by these advances and the persistent gap in electrochemical allene functionalization via radical‐based additions, we sought to develop a selective electrochemical radical cyclization of allenes tethered with nucleophile (Scheme 1b right). We envisioned that a suitable electrochemically generated radical could add selectively at the central carbon of the allenyl framework (Scheme 1c). The resulting allyl radical would then undergo anodic oxidation to generate a stabilized cationic species capable of intramolecular nucleophilic cyclization. This sequence of radical addition, oxidation, and controlled cyclization offers a way to overcome the usual regioselectivity limitations and provides an efficient route to biologically relevant heterocycles in a single electrochemical step. Key challenges associated with the envisioned approach include undesired competing radical difunctionalizations and eliminations (Scheme 1d). Another challenge is to identify a suitable radical source capable of mediating these transformations under mild electrochemical conditions. Guided by these considerations, we identified diphenyl diselenide as an effective mediator, given the ability of selenium‐based radicals to engage *π*‐systems with high selectivity under anodic redox conditions [69, 70, 71]. Building on this mechanistic rationale, we developed an electrochemical protocol that employs allenyl amides in combination with diphenyl diselenide, enabling selective selenium radical addition at the central allenic carbon followed by oxidation‐induced cyclization to deliver oxazoline scaffolds (Scheme 1e). The methodology was further extended to allenyl urea derivatives, which furnished oxazolidin‐2‐imines. Both oxazoline and oxazolidin‐2‐imine units constitute biologically relevant heterocycles commonly encountered in bioactive molecules and therapeutic leads (Scheme 1f) [72, 73, 74, 75]. In particular, the oxazoline scaffolds have been widely explored as a directing group in C‐H activation, and oxazolinyl‐based ligands have been studied in asymmetric catalysis [76, 77]. However, the traditional methodologies for the synthesis of these heterocyclic core are often constrained by the use of transition metals and the requirement of harsh conditions [78, 79]. Consequently, the development of a mild, environmentally benign and sustainable alternative strategy that obviates the need for such metals remains highly desirable [80, 81, 82]. At the same time, among the organoselenium compounds, vinyl selenides are the most synthetically valuable building blocks, owing to their versatility in stereoselective transformations [83, 84].

In this study, along with establishing the substrate scope and limitations, comprehensive mechanistic investigations have been carried out that substantiate the proposed reaction pathway. Cyclic voltammetry reveals the relevant redox events and supports the sequential radical‐generation and oxidation steps. Radical‐trapping experiments confirm the intermediacy of radical species, while on/off electrolysis experiments demonstrate that continuous electron transfer is essential for productive reactivity. Free energy calculations by DFT further support the proposed mechanistic pathway. Together, these mechanistic insights converge into a clear picture that explains the origin of regioselectivity and validates the designed radical addition–oxidation–cyclization sequence.

## 2 Results and Discussion

### 2.1 Optimization of the Reaction Conditions

**SCHEME 2:**
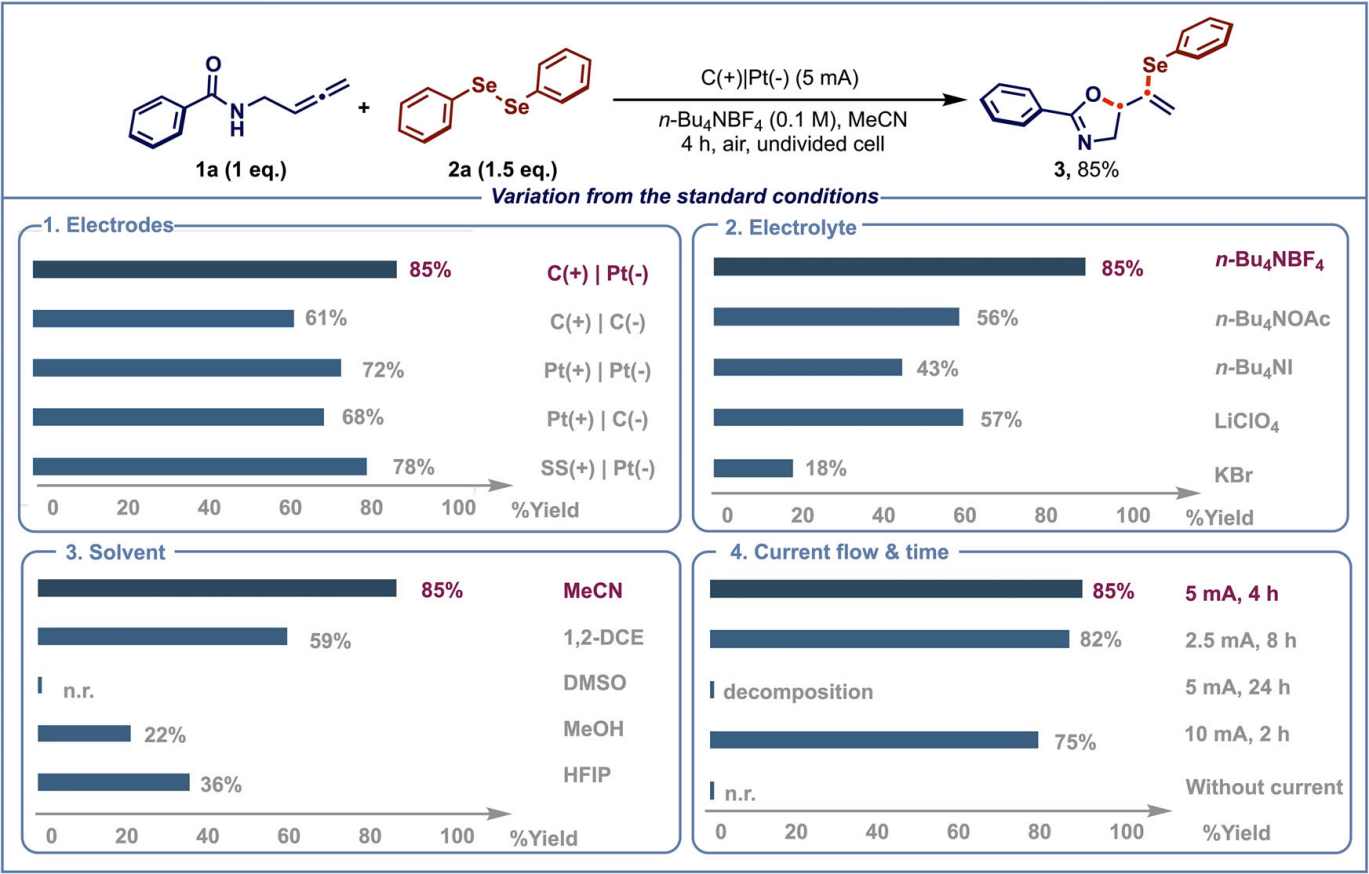
Optimization of the reaction conditions.

We commenced our investigation using *N*‐(buta‐2,3‐dien‐1‐yl)benzamide **1a** as the model substrate and diphenyl diselenide **2a** as the selenium radical source to optimize the electrochemical reaction conditions. After extensive screening of various reaction parameters, we were delighted to obtain the desired oxazoline derivative **3** in 85% yield under constant‐current electrolysis (I = 5 mA) in acetonitrile containing 0.1 m *n*‐Bu_4_NBF_4_ in an undivided cell equipped with a graphite anode and platinum cathode (Scheme 2). Using either graphite or platinum for both electrodes led to reduced yields and replacing graphite with stainless steel at the anode furnished **3** in only 78%. Evaluation of different supporting electrolytes suggested that *n*‐Bu_4_NBF_4_ is the optimum electrolyte as other electrolytes such as *n*‐Bu_4_NOAc, *n*‐Bu_4_NI, LiClO_4_ and KBr resulted in diminished yields ranging from 18% to 57%. Solvent screening identified acetonitrile as the optimal solvent as DCE afforded **3** in 59%, no reaction occurred in DMSO and protic solvents such as MeOH and HFIP gave inferior results. Varying the applied current above (10 mA) or below (2.5 mA) 5 mA also proved to be less efficient. It is worth mentioning that running the reaction for longer time (24 h) with 5 mA current resulted in complete decomposition of the product. Notably, stopping the flow of current stopped the reaction emphasizing the essential role of electric current in this electrochemical reaction protocol.

### 2.2 Substrate Scope

**SCHEME 3:**
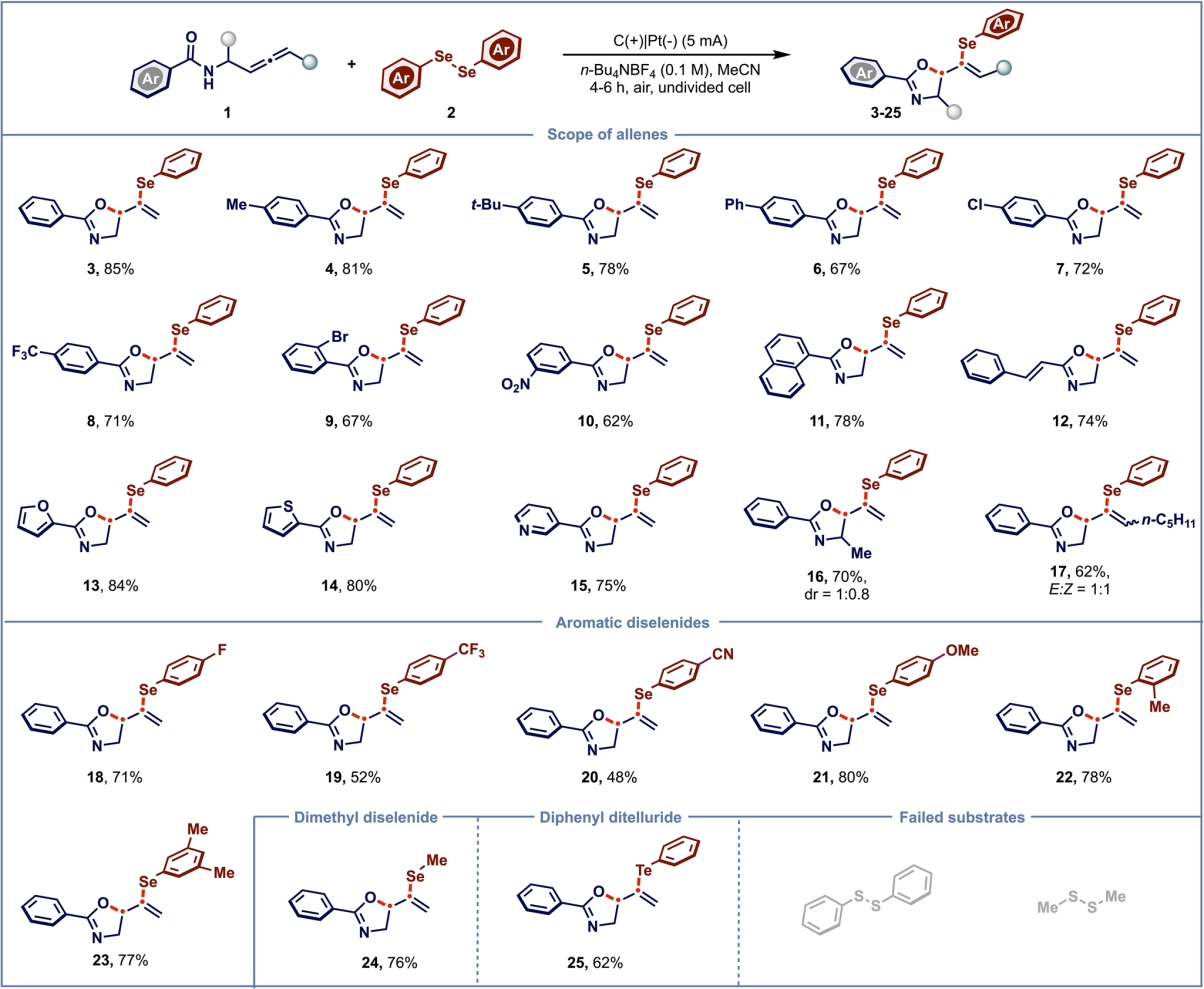
Substrate scope with allenyl amides.

After having the optimized reaction conditions in hand, we examined the generality of the developed protocol using diversely substituted allenyl amides **1** and diphenyl diselenide **2a** (Scheme 3). Both electron‐donating and ‐withdrawing groups at the *para*‐position of phenyl ring were well tolerated, affording oxazoline vinylselenides **4–8**. Electron‐donating substituents (methyl and *t*‐butyl) gave the desired products in higher yields (78%–81%), whereas slightly lower yields were observed for electron‐withdrawing substituents (phenyl, chloro, and trifluoro), producing **6–8** in 67%–72%. *ortho*‐Bromo derivative furnished **9** in 67% and *meta*‐NO_2_ substituted amide afforded **10** in 62%. 1‐Naphthyl‐ and *β*‐styrenyl‐substituted allenes underwent the transformation smoothly to produce **11–12** in good yields (74%–78%). Notably, allenyl amides bearing heterocyclic motifs such 2‐furanyl, 2‐thienyl and 3‐pyridyl were also compatible under the reaction conditions and furnished the desired products **13–15** in good yields (75%–84%). α‐Methyl substituted allene led to **16** as a 1:0.8 mixture of diastereomers in 70%. Similarly, in the case of 1,3‐disubstituted allene, the corresponding product **17** was obtained as a 1:1 mixture of *E*‐ and *Z*‐stereoisomers with a combined yield of 62%. We then examined the influence of electronic variation on diaryl diselenides **2**. Both electron‐withdrawing (*p*‐F, *p*‐CF_3_ and *p*‐CN) and ‐donating (*p*‐OMe, *o*‐Me and 3,5‐di‐Me) substituents at different positions of the aryl ring were well accommodated to provide the corresponding products **18–23**. Also, dimethyl diselenide was found to be amenable under the reaction conditions to furnish the corresponding product **24** in 76% yield. Remarkably, diphenyl ditelluride also proved effective as the source of aryltellurium radical and afforded the corresponding tellurium derivative **25** in 62%. However, both diphenyl disulfide and dimethyl disulfide failed to show any reaction under the optimized reaction conditions.

**SCHEME 4:**
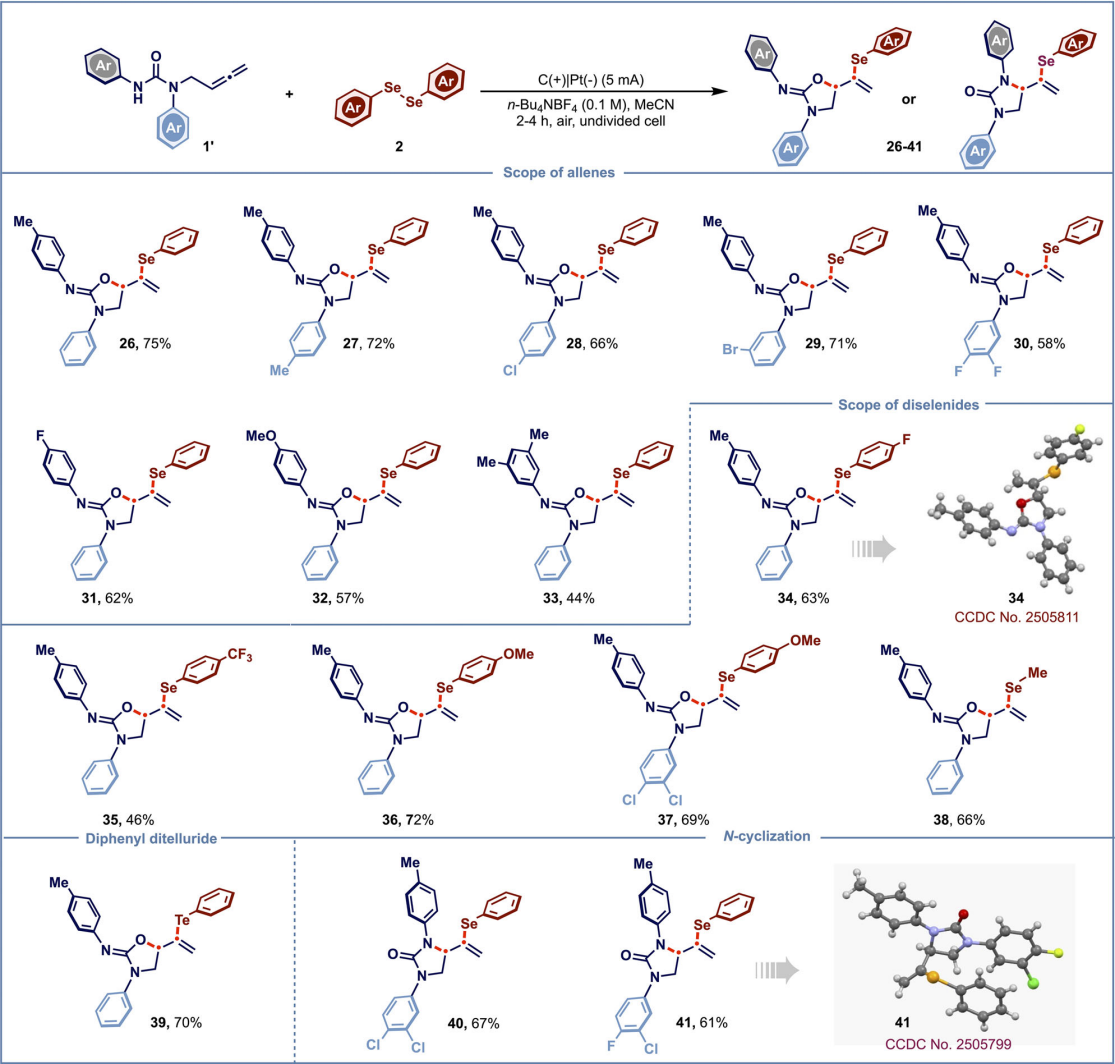
Substrate scope with allenyl urea derivatives.

Encouraged by the efficient formation of oxazoline derivatives from allenyl amides, we next turned our attention to allenes tethered with urea (Scheme 4). Under the optimized electrochemical conditions, allenyl urea **1**′**a** underwent selective *O*‐cyclization with diphenyl diselenide **2a** and produced oxazolidine‐2‐imine **26** in 75% yield. To assess the generality of this process, we examined allenes having various electron‐donating, ‐withdrawing, and halo‐substituents on the phenyl ring attached to internal nitrogen (tertiary), and the corresponding oxazolidine‐2‐imines **27–30** were obtained in moderate to good yields (58%–72%). Substitution at the *para*‐ and *meta*‐position of the phenyl ring connected to the secondary nitrogen was also compatible to deliver **31–33** in 44%–62% yields. Subsequent variation of diaryl diselenides bearing electron‐poor and ‐rich substituents at the *para*‐position led to products **34–37** in 46%–72% yield. Notably, dimethyl diselenide also proved effective as the source of methylselenium radical and afforded the corresponding derivative **38** in 66%. Moreover, diphenyl ditelluride again proved viable and furnished **39** in 70% yield.

Notably, some substrates bearing 3,4‐dihalogenated phenyl groups on the tertiary nitrogen displayed a switch in selectivity, delivering *N*‐cyclized products **40** and **41** in 67 and 61% yields respectively. Structures of both the *O*‐ and *N*‐cyclized compounds were unambiguously confirmed by the single‐crystal X‐ray diffraction analysis of compounds **34** and **41**.

### 2.3 Gram‐Scale Synthesis, Halocyclization and Synthetic Utility

**SCHEME 5:**
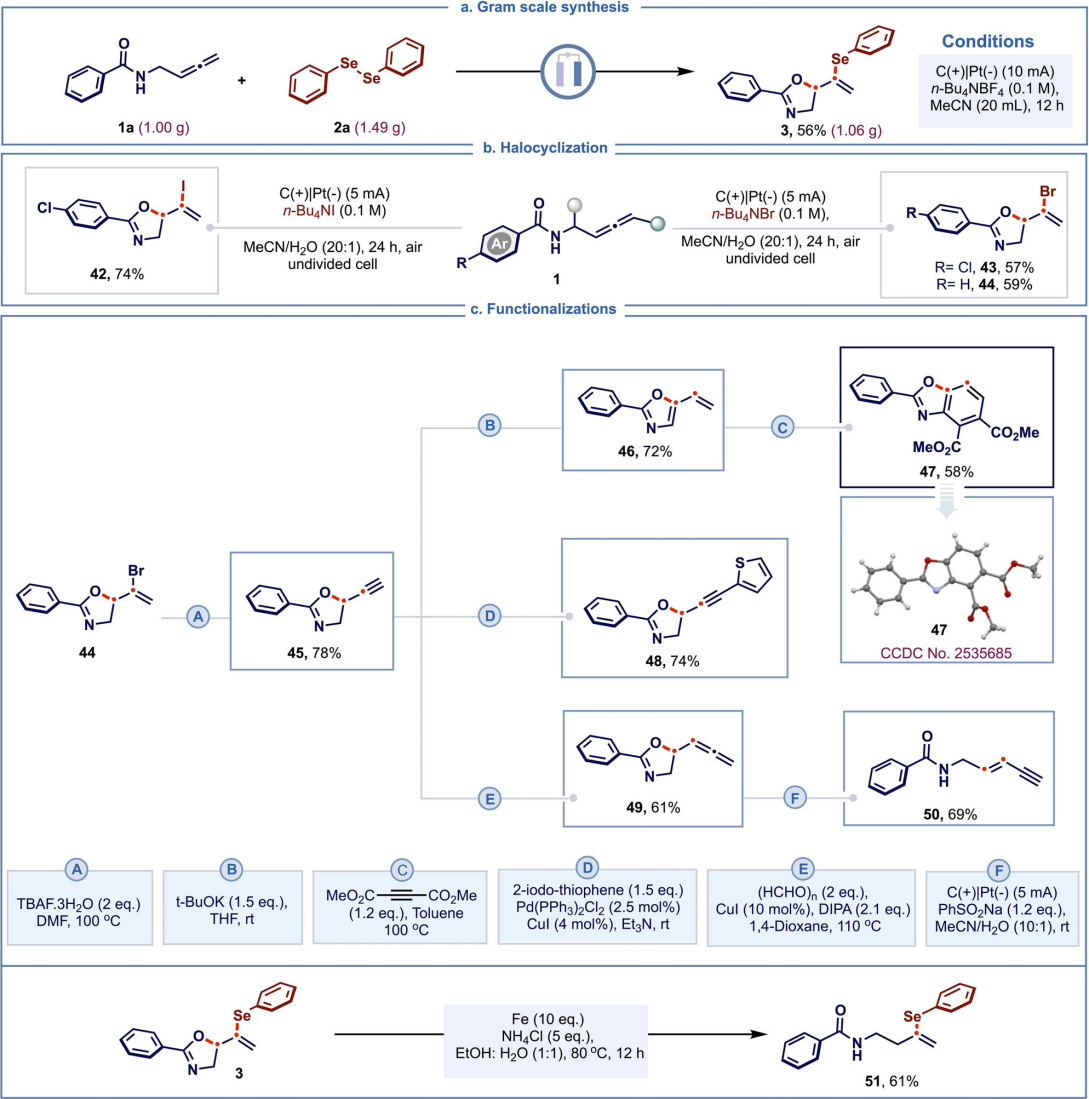
Gram scale synthesis, halocyclization and further functionalizations.

To illustrate the practicality of current electrochemical reaction protocol, a gram‐scale reaction was conducted with 1.0 g of *N*‐(buta‐2,3‐dien‐1‐yl)benzamide **1a** and 1.49 g of diphenyl diselenide **2a** to furnish 1.06 g of **3** in 56% yield highlighting the practicality of the protocol (Scheme 5a). Interestingly, during the control studies with various electrolytes, we observed that halocyclization products were formed when ammonium halides were used as the supporting electrolyte in the absence of diphenyl diselenide **2a** [85]. Given the synthetic relevance of vinyl halides, this intriguing reactivity prompted us to explore halogen mediated cyclization of allenyl amide. Screening various electrolytes as halogen source indicated that both tetrabutylammonium iodide and tetrabutylammonium bromide were effective precursors, providing the corresponding products **42–44** (Scheme 5b). To demonstrate the synthetic utility of the developed protocol (Scheme 5c), oxazoline **44** was efficiently converted to the corresponding terminal alkyne–bearing oxazoline **45** via dehydrobromination (78% yield). Gratifyingly, compound **45** was converted to 2‐phenyl‐5‐vinyloxazole **46** in 72% yield. Subsequent reaction of **46** as a diene precursor with dimethyl but‐2‐ynedioate as the dienophile proceeded via a Diels–Alder cycloaddition, and the resulting adduct underwent aerial oxidation to furnish compound **47** in 58% yield. The structure of **47** was unambiguously confirmed by the single‐crystal X‐ray diffraction analysis. Furthermore, installation of a thienyl moiety onto **45** via Sonogashira cross‐coupling afforded the desired product **48** (74% yield), thereby incorporating two distinct heterocyclic motifs within a single molecular framework. In addition, exposure of **45** to standard Crabbé homologation conditions enabled efficient conversion to the corresponding allene **49** in 61% yield. Simultaneously, reaction of compound **49** with sodium benzenesulfinate under constant‐current electrolysis (I = 5 mA) in acetonitrile/H_2_O (10:1) ended up with the formation of enyne **50** in 69% yield. Treatment of vinyl selenide **3** with 10 equiv. of Fe and 5 equiv. of NH_4_Cl resulted in reductive ring‐opening to deliver vinyl selenide **51** in 61% yield. Interestingly, the tandem electrochemical seleno‐cyclization of **1** followed by iron‐mediated reduction results in the formal hydroselenylation of allenes in a regioselective manner.

### 2.4 Mechanistic Studies

**SCHEME 6:**
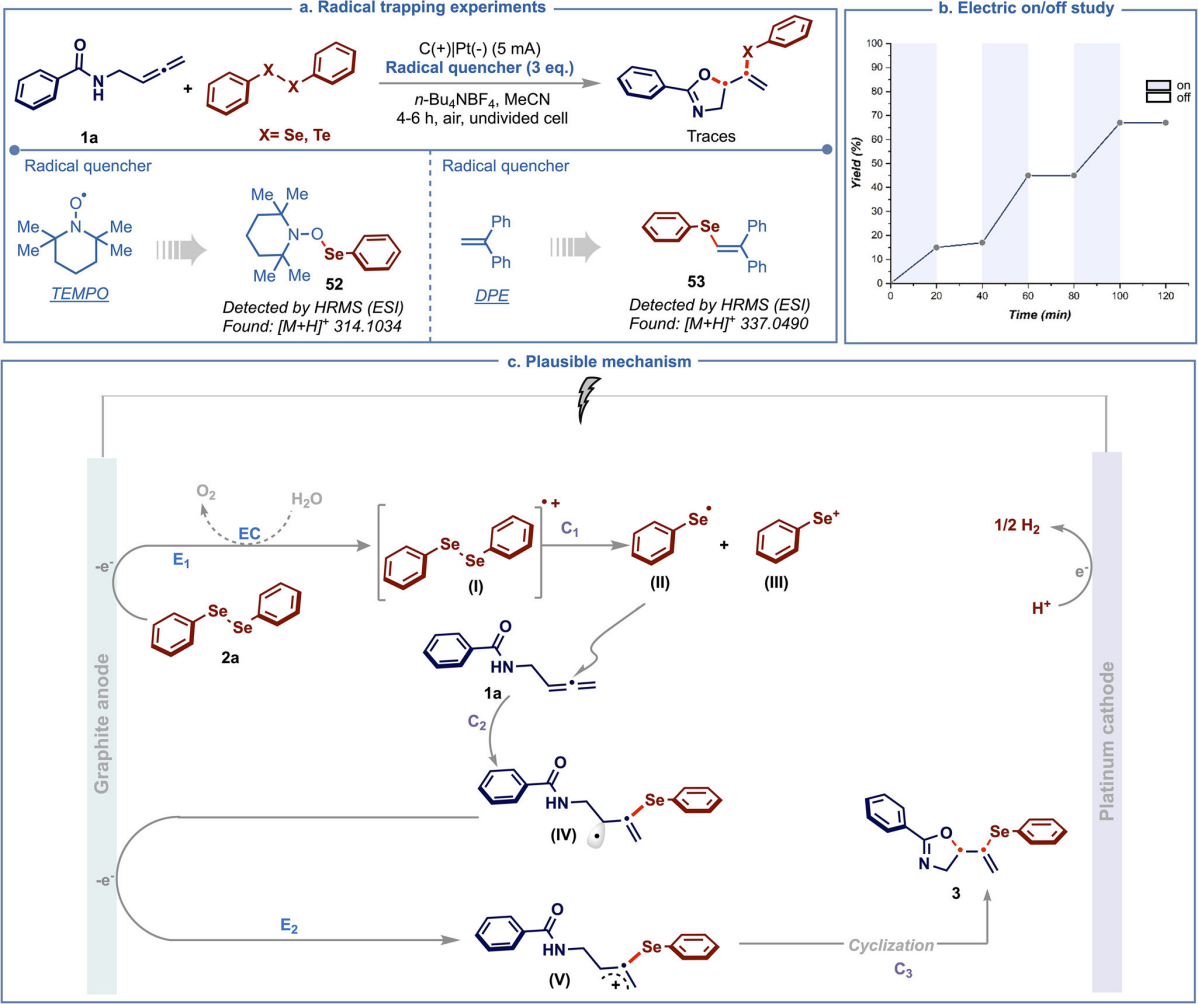
Radical trapping experiments, electric on‐off study and plausible mechanism.

To gain some insights on the reaction mechanism, radical inhibition studies were carried out. Electrolysis of **1a** under standard conditions with **2a** in the presence of radical scavengers, such as TEMPO or 1,1‐diphenylethene (DPE), completely inhibited the reaction and the selenium adducts **52** and **53** were observed in HRMS (Scheme 6a). Radical trapping experiments for the reaction of **1a** with diphenyl ditelluride also showed similar results. These studies suggest possible radical involvement in this pathway. Electric on–off experiment was also performed using **1a** and **2a** (Scheme 6b). The reaction mixture was subjected to alternating 20 min intervals of electrolysis and resting. No progress was observed during the off intervals, clearly demonstrating that a constant electric current is essential for this transformation. Based on these preliminary experiments, the plausible mechanism is depicted in Scheme 6c for the electrochemical selenocyclization of allenyl amides (detailed discussion below). Anodic oxidation of diphenyl diselenide **2a** likely generates radical cation intermediate **I**, which can dissociate to give phenylselenium radical **II** along with phenylselenium cation **III**. Phenylselenium radical **II** can then undergo regioselective addition to the allenyl amide **1a**, generating a stablized allyl radical intermediate **IV**. Subsequent anodic oxidation of radical **IV** leads to the corresponding allyl cation intermediate **V**, which can undergo rapid intramolecular nucleophilic cyclization through oxygen atom furnishing the cyclized product **3**. Proton reduction to dihydrogen at the Pt cathode is posited to provide the charge balance for the overall transformation. It is worth mentioning that radical trapping experiments with either TEMPO or DPE for the halocyclization leading to **42** or **43** didn't inhibit the electrochemical cyclization, which suggests that the halocyc might proceed via the formation of a halonium ion, followed by electrophilic addition to the allene and subsequent intramolecular nucleophilic cyclization.

### 2.5 Electrochemical Studies

**SCHEME 7:**
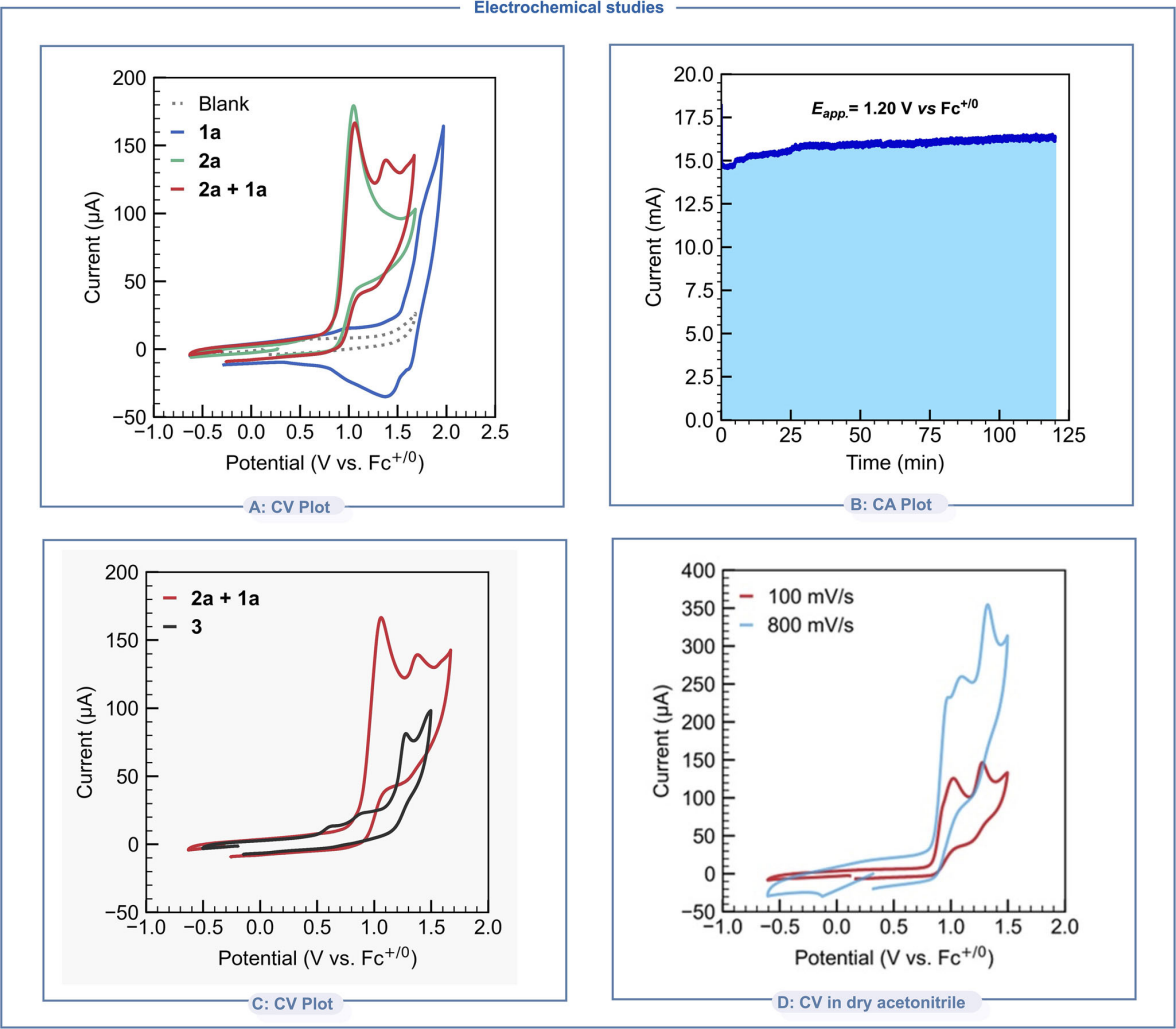
Electrochemical studies. (A) Cyclic voltammograms recorded at 100 mV s^−^^1^ in acetonitrile containing 0.2 m *n*‐Bu_4_NBF_4_, 2 mm of (PhSe)_2_ **2a** (green), 2 mm of allenyl amide **1a** (blue), and 2 mm of **1a** + 2 mm of (PhSe)_2_ **2a** (red) solutions. The background with no substrate or (PhSe)_2_ **2a** is shown as gray dotted lines. (B) Current–time trace in acetonitrile for bulk electrolysis at a constant potential of 1.20 V vs Fc^+^^/0^, containing 0.20 mmol of **1a** and 0.37 mmol of **2a**. (C) Cyclic voltammograms recorded at 100 mV s^−^^1^ in MeCN containing 0.2 m *n*‐Bu_4_NBF_4_ as supporting electrolyte, and 2 mm of **1a** + 2 mm of **2a** (red) solution and **3** (charcoal gray). (D) Cyclic voltammograms recorded in MeCN in the presence of desiccant with 2 mm of **1a** + 2 mm of **2a** at a scan rate of 100 mV/s (red) and 800 mV/s (blue).

To elucidate the mechanism of electrochemical functionalization of allenes, we performed cyclic voltammetry (CV) experiments (Scheme 7a). We first independently recorded CVs of allenyl amide **1a**, diphenyl diselenide **2a** and urea tethered allene **1**′**a** in acetonitrile to probe their oxidative features. A 2 mm solution of **2a** exhibits an irreversible oxidation peak at 1.05 V vs. Fc^+^^/0^ (Fc = Ferrocene) corresponding to the formation of [(PhSe)_2_]^•^^+^ (**I**), which dissociates into the phenylselenium radical [PhSe]^•^ (**II**) and the phenylselenium cation [PhSe]^+^ (**III**). We note that the observed peak current in the CV of **2a** is higher than the corresponding value predicted by the Randles–Sevcik equation, given the concentration, scan‐rate and electrode area [86]. We attribute this higher current to the background electrocatalytic oxidation of residual water by (PhSe)_2_, as reported previously [87].

In the CV of a 2 mm solution of allenyl amide **1a**, we observe an oxidative feature near ∼1.75 V vs Fc^+^^/0^, accompanied by irreversible reduction peaks at ∼1.65 and 1.45 V. Interestingly, in the presence of both **1a** and **2a** (2 mm), we observe the emergence of a new irreversible oxidation peak near 1.40 V (Scheme 7A). This new peak likely corresponds to the formation of a new species on the CV timescale, which could either correspond to a reaction intermediate or the product itself. Accordingly, we performed a constant potential electrolysis (CPE) experiment in a three‐electrode electrolysis cell setup (see Supporting Information), with the working electrode potential held at 1.20 V for 2 h (Scheme 7B), which is lower than the onset potential of the oxidation feature near 1.40 V. With subsequent workup of the CPE solution, we detected the formation of the oxazoline product **3** with 51% isolated yield and a Faradaic efficiency of ∼21.7%. Given this observation, it is therefore, likely that the new peak near 1.40 V in the CV with allenyl amide **1a** and (PhSe)_2_ **2a** (Scheme 7A) corresponds to the oxidation of the in situ generated product **3** instead of any intermediary species (vide infra). To further confirm this hypothesis, we recorded the CV of a 2 mm solution of the oxazoline product **3**, which indeed shows a distinct oxidation feature near 1.40 V (Scheme 7C). These results, therefore, strongly support the transformation of allenyl amide **1a** to product **3**, mediated by the oxidation of **2a**, at a potential that is lower than the onset of the new peak near 1.40 V in Scheme 7A (in red). The observation of the distinct oxidation feature near 1.40 V, corresponding to the in situ generated product **3** within the CV timescale, points to facile kinetics of the overall transformation. Yet, we do not observe a clear cathodic shift in the onset potential or current enhancement for the oxidation of (PhSe)_2_ **2a**, which is likely due to competing water oxidation [87]. Upon the addition of activated molecular sieves to the electrochemical cell to trap residual water present in acetonitrile, a small shoulder appears near 0.90 V in the CV at 100 mV/s, which grows in intensity relative to the other oxidative features at higher scan rates (Scheme 7D). Minimizing water content likely suppresses the rate of the background water oxidation catalyzed by (PhSe)_2_ **2a**, shifting the balance toward allene oxidation, as per the mechanistic schematic shown in Scheme 6c. As a result, we posit that the shoulder feature near 0.9 V is due to the E_1_C_1_C_2_E_2_C_3_ pathway (Scheme 6c) gaining dominance, resulting in a cathodic shift of the oxidation feature of (PhSe)_2_. Under these conditions, the product oxidation peak near 1.40 V grows proportionally to the shoulder feature, while the central (PhSe)_2_ peak grows more slowly, reflecting partial dependence on the water‐mediated pathway.

Analogs of allenyl amide **1** with various substituents on the phenyl ring (CF_3_, Me, or *t*‐Bu), show nearly identical redox features in the absence and presence of (PhSe)_2_ (Figures S3–S5). Further, CV studies with the urea‐tethered allene **1**′**a** also indicated that a similar pathway is operational with diphenyl diselenide **2a** (Figures S6 and S7).

### 2.6 DFT Studies

**SCHEME 8:**
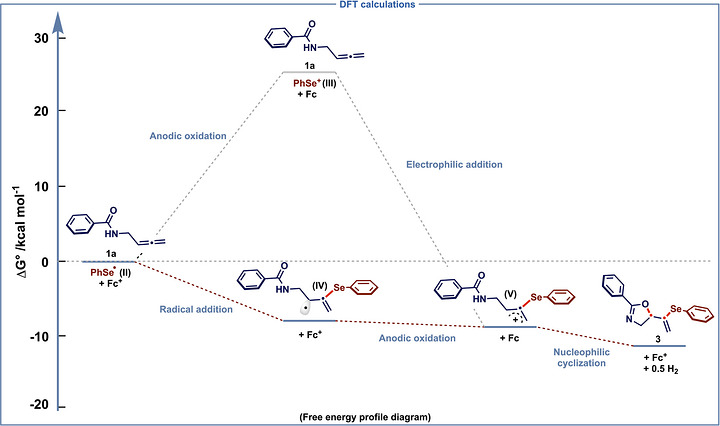
DFT Calculations: Computed free energy profile for the electro‐oxidative functionalization of allenyl amide **1a**.

The ordering of redox potentials, calculated by DFT benchmarked against the (PhSe)_2_^•+/0^ potential, follows **2a** < **3**< **1a,** in agreement with the experimental CV data (Table S1). The free‐energy profile of the plausible pathways, as computed by DFT, is shown in Scheme 8. Ferrocenium was employed as the one‐electron oxidant in the calculations. The first step after the dissociative oxidation of (PhSe)_2_ involves the exergonic addition of PhSe^•^ **(II)** onto the allene double bond to form **IV** (−7.9 kcal/mol). Subsequent oxidation of **IV** to the corresponding cation **(V)** is slightly exergonic (−0.9 kcal/mol). The alternative path for the formation of **V** via the PhSe^+^ cation **(III)** adding to **1a** is relatively endergonic (by ∼33 kcal/mol). While the addition of a second PhSe• to **IV** to form **VI** (Figure S9) was found to be exergonic (−37.8 kcal/mol), under the electrochemical conditions employed, the more facile one‐electron oxidation of **IV** to **V** is likely to be dominant. Finally, intramolecular cyclization, deprotonation and H_2_ evolution affords the oxazoline **3** in an exergonic step (−3 kcal/mol).

## 3 Conclusions

In summary, we have developed a regioselective electrochemical radical functionalization of allenes, wherein radical addition at the central carbon triggers an oxidative cyclization to furnish oxazoline scaffolds under mild conditions. Electrochemical radical additions to allenes remain scarcely explored, and their integration with intramolecular cyclization pathways has been particularly underdeveloped. This study addresses that gap with a mechanistically well‐defined platform where diphenyl diselenide (or ditelluride) serves as a redox‐compatible radical mediator enabling selective engagement of the allenic *π*‐system and overcomes regioselectivity challenges of radical addition to cumulenes. Cyclic voltammetry, radical probe experiments, and DFT calculations collectively support a pathway involving allyl‐radical formation followed by anodic oxidation to a cationic intermediate that undergoes intramolecular nucleophilic cyclization. The successful extension of this strategy to allenyl urea further highlights its generality and synthetic relevance. Overall, this work establishes a mechanistically distinct and synthetically versatile approach to electrochemical allene functionalization, broadening opportunities for radical‐mediated heterocycle construction within electroorganic synthesis.

## Author Contributions

**Pranjal Pyne**: Writing – original draft, Writing – review and editing, investigation, validation, methodology, software, formal analysis. **Shivam Pandey**: writing – original draft, writing – review and editing. **Chandra M. R. Volla**: conceptualization, funding acquisition, writing – original draft, methodology, validation, writing – review and editing, project administration, data curation, resources, supervision. **Prateek Saini**: investigation, validation, writing – original draft, writing – review and editing, methodology, software, formal analysis. **Srinivasan Ramakrishnan**: conceptualization, funding acquisition, writing – original draft, methodology, validation, writing – review and editing, project administration, data curation, supervision, resources. **Rohel Hoque**: investigation, formal analysis, data curation, software.

## Conflicts of Interest

The authors declare no conflicts of interest.

## Supporting information




**Supporting File**: advs76939‐sup‐0001‐SuppMat.pdf.

## Data Availability

The data that support the findings of this study are available from the corresponding author upon reasonable request.
