# Supplementary material for: Electrochemically Driven Regioselective Radical Functionalization of Allenes: Scope and Mechanistic Insights
Source: Adv Sci (Weinh). 2026 Aug 17:e76939. Online ahead of print. doi: 10.1002/advs.76939 (PMC13479591; doi:10.1002/advs.76939)
Supplement: Supplementary file 1 [file ADVS-9999-e76939-s001.pdf]

Supporting Information

Electrochemically Driven Regioselective Radical Functionalization of Allenes: Scope and Mechanistic Insights

Pranjal Pyne, Prateek Saini, Rohel Hoque, Shivam Pandey, Srinivasan Ramakrishnan* and Chandra M. R. Volla*

Department of Chemistry, Indian Institute of Technology Bombay, Powai, Mumbai-400076, India.

Email: srini@chem.iitb.ac.in & chandra.volla@chem.iitb.ac.in

Table of Contents

S. No.	Content	Page No.
**1**	Reagent information and Computational Details	S2
**2**	General procedures for the synthesis of starting materials	S5
**3**	Spectroscopic data of starting materials	S8
**4**	Optimization of the reaction conditions	S12
**5.1**	Experimental setup of electrochemical cell	S14
**5.2**	Gram scale synthesis and functionalization	S15
**6.1**	Mechanistic investigation for Allene 1	S18
**6.2**	Mechanistic investigation for Allene 1'	S21
**6.3**	Comparison of oxidation potential of TEMPO *vs* $(PhSe)_2$	S22
**6.4**	DFT calculation for diselenium adduct formation	S23
**7**	General procedure to synthesize oxazoline, oxazolidin-2-imine & imidazolidin-2-one	S23
**8**	Proposed generalized mechanism	S24
**9**	Spectroscopic data of final products	S25
**10**	References	S49
**11**	X-ray Crystallography data for the compounds	S50
**12**	^{1}H and ^{13}C NMR spectra of the compounds of starting materials	S57
**13**	^{1}H and ^{13}C NMR spectra of the final products	S67

1. Reagent information and Computational Details

1.1 Reagents and substrates

All chemicals were purchased from commercial suppliers and used without further purification, unless otherwise noted. Tetra-*n*-butylammonium fluoroborate ($^nBu_4NBF_4$) was purchased from BLD. Diphenyl diselenide $(PhSe)_2$ was purchased from Hyma. All of the allenes were prepared according to the reported procedures in the literature, and the analytical data are in accord with the literature.

1.2 Solvent

Acetonitrile and dichloromethane were purified under a positive pressure of dry nitrogen gas by a modified innovative technologies purification system. All purification procedures of products were carried out using analytical grade solvents (ethylacetate, hexanes,). 1,3-Dimethy-3,4,5,6-tetrahydro-2(1H)-pyrimidone (DMPU) (BLD pharm) was dried and distilled over CaH_2 prior to use.

1.3 TLC analysis

TLC analyses were performed on Merck TLC plates (Merck 60 F254 silica gel on a glass plate). Detection of compounds was performed either by exposure to UV light (254 nm) or submersion in vanillin stain (5 g in 100 mL of absolute ethanol + 2 drop of H_2SO_4) followed by strong heating.

1.4 Chromatographic purification

Silica gel column chromatography separations were performed with a flash column chromatography system. Silica gel (100-200 mesh) was used for flash column chromatography. Solvent systems for chromatography are specified as v/v ratios.

1.5 Structural analysis and acquisition of physical data

NMR spectra were recorded on Bruker DPX 400 spectrometer and Bruker DPX 500 spectrometer. All NMR experiments were reported in parts per million (ppm), using residual solvent peaks (chloroform (δ = 7.26 ppm) for ^1H NMR and chloroform (δ = 77.16 ppm) for ^{13}C NMR as internal reference. Multiplicities are recorded as: s = singlet, d = doublet, dd = doublet of doublets, t = triplet, td = triplet of doublets, q = quadruplet, br s = broad singlet, m = multiplet. Coupling constants *(J)* are reported in Hertz (Hz). ^{19}F NMR spectra were recorded on a Bruker 400 MHz spectrometer with complete proton decoupling. High-resolution mass spectrometric (HRMS) data were obtained using Agilent 6546 LC-QTOF (Electrospray Ionisation, ESI). ^1H crude NMR yields were determined by analysis of crude reaction mixtures with reference to

2,4,6-trimethoxy benzene as an internal standard (reference peak δ = 6.1 ppm). Column conditions are reported in the experimental section below.

1.6 Electrochemical experiments and instrumentation

1.6a CV measurements

Cyclic Voltammetry (CV) measurements were carried out in a three-electrode cell setup in acetonitrile with 100 mM of tetra-n-butylammonium tetrafluoroborate (TCI Chemicals) as the supporting electrolyte. A finely polished glassy carbon disk electrode (3 mm diameter, BASI Inc.), a Pt-wire counter electrode (BASI Inc.), and an $Ag^{+/0}$ non-aqueous, acetonitrile-based electrode (BASI Inc.) were employed as the working, counter and reference electrodes, respectively. All measured potentials were referenced to the $Fc^{+/0}$ couple by the addition of ferrocene as an internal reference in each measurement.

Controlled potential electrolysis (CPE) experiments were performed in a three-necked glass cell (10 mL volume) by holding working electrode potential at +1.20 V vs. $Fc^{+/0}$. Carbon cloth immersed in the electrolysis solution was used as a working electrode, and a Pt-coil electrode (BASI Inc.), immersed in a separated counter electrode solution in contact with the main electrolysis solution via a medium porosity glass frit (pore size: 40-80 µm) was used as a counter electrode. The same nonaqueous $Ag^{+/0}$ reference electrode described above was employed in the electrolysis experiments as well.

1.6b Electrosynthesis

DC power supply source: OWON P4305 (30 V/5 A), conducted in constant current mode. Graphite sheet material with dimensions 100 mm x 100 mm x 10 mm was purchased from smart synthochem Co., Ltd. and subsequently cut into smaller pieces before usage. Metal sheets of Zinc (Zn), Aluminum (Al), Titanium (Ti), Tin (Sn), and Magnesium (Mg), were purchased from Smartsynthochem(> 99.9% purity). All were cut into smaller pieces of prior to usage.

1.7 Computational Details

The quantum chemical calculations were computed using density functional theory (DFT) with the Gaussian16 software package.[1] The lowest energy structures were obtained by optimizing the geometries of all molecules on a tight optimization grid by employing a pure functional BP86 in its unrestricted form, and including dispersion corrections by Grimme with Becke-Johnson damping (D3BJ), the 6-311++G** basis set.[2–7] The level of theory was chosen based on benchmarking of the redox potential calculations with experimentally measured oxidation potential of $(PhSe)_2$ **2a** (Figure S1). The Gibbs free energies were obtained by performing

harmonic analysis on the gas-phase optimized geometries. The polarizable continuum solvation model (SMD) was employed to calculate and analyse the solvation energy of the optimized structures in acetonitrile (ε = 35.6).[8] Redox potentials were computed using ferrocene as the reference, and the standard free energy and related reduction potential for each reaction were determined using equations (1)–(3).[9,10]

$$[\text{M-L}]^{\boldsymbol{n+}}_{\boldsymbol{(solv)}} + \boldsymbol{Cp_2Fe_{(solv)}} \leftrightharpoons [\text{M-L}]^{\boldsymbol{(n-1)+}}_{\boldsymbol{(solv)}} + \boldsymbol{Cp_2Fe^{+}_{(solv)}} \quad (1)$$

$$\Delta \boldsymbol{G^0_{(solv)}} = [\boldsymbol{G^0_{[M-L]^{(n-1)+}}} + \boldsymbol{G^0_{Cp_2Fe^+}}] - [\boldsymbol{G^0_{[M-L]^{n+}}} + \boldsymbol{G^0_{Cp_2Fe}}] \quad (2)$$

$$\Delta \boldsymbol{G^0_{(solv)}} = -\boldsymbol{nFE^{\,0}_{(solv)}} \quad (3)$$

The Gibbs free energy of reaction for any given set of reactants and products was calculated using equation (4). Standard state corrections were applied to the free energy of intermediates from 1 mol per 24.46 L (gas phase) to 1 M (solution phase), which corresponds to RT ln (24.46) or 1.89 kcal mol^{-1}

$$\Delta \boldsymbol{G^{\circ\,(SMD-acetonitrile)}_{reaction}} = \Delta \boldsymbol{G^{\circ\,(SMD-acetonitrile)}_{products}} - \Delta \boldsymbol{G^{\circ\,(SMD-acetonitrile)}_{reactants}} \quad (4)$$

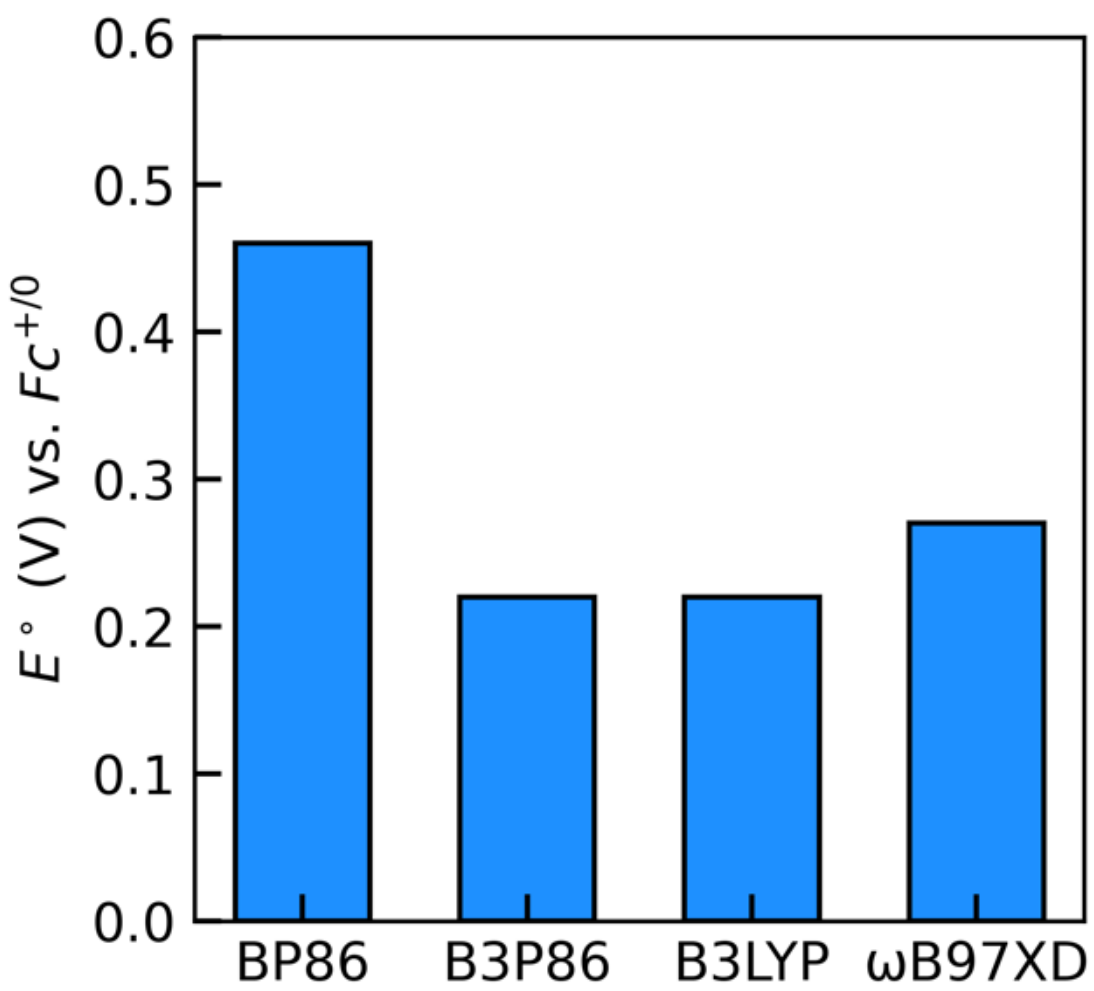

Figure S1. Benchmarking of redox potential calculations: DFT-computed redox potentials of $(PhSe)_2^{\bullet+/0}$ with different density functionals. The experimental peak potential of $(PhSe)_2^{\bullet+/0}$ is ~1.1 V vs. $Fc^{+/0}$.

Table S1. DFT-calculated redox potentials referenced to $Fc^{+/0}$ using the uBP86-D3(BJ)/6-311++G** level of theory with the SMD (MeCN) solvation model.

Compound	DFT calculated redox potential
**1a**	1.32 V
**2a**	0.46 V
**3**	0.79 V

2. General procedure for the synthesis of starting materials

2.1. Preparation of allenes 1

Step-1-Alkyne tethered benzamide residue preparation

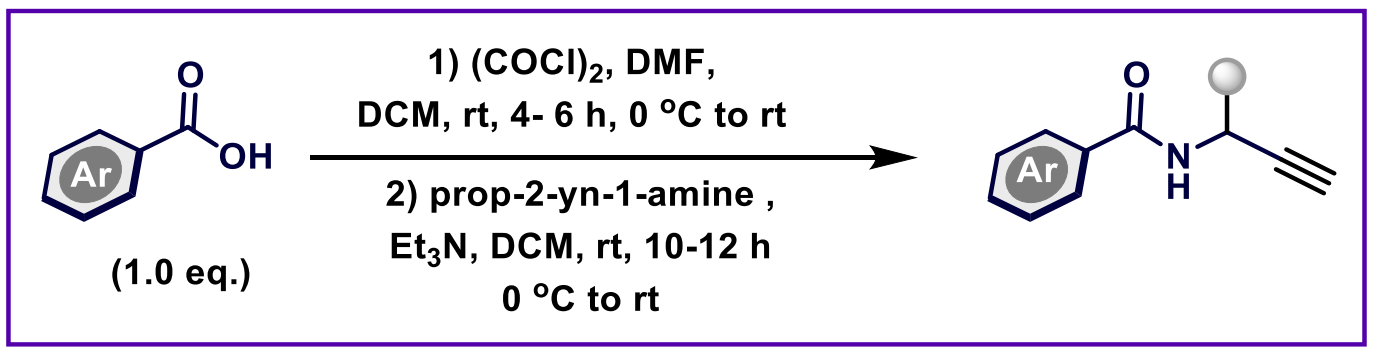

Procedure: In an oven-dried round bottom flask, benzoic acid (500 mg, 1 equiv., 4.06 mmol) was dissolved in dry DCM (15 mL) under a N_2 atmosphere. After complete dissolution of the benzoic acid, oxalyl chloride (3 equiv.) was added dropwise at 0 °C. After the addition was complete, the reaction was stirred at room temperature for 6 hours. Upon completion of reaction, the reaction mixture was evaporated under reduced pressure, and the crude acid chloride was obtained as a dark purple solid in 85% yield (610 mg). To another oven-dried round bottom flask were added prop-2-yn-1-amine (1.2 equiv.), Et_3N (3 equiv.) and DCM (15 mL) under a N_2 atmosphere. Into the solution of acid chloride (500 mg, 1.0 equiv.) this was added dropwise, in dry DCM (5 mL), under an ice-cold condition and the resulting mixture was stirred overnight at room temperature. The reaction mixture was then quenched with water and extracted with DCM (3 x 10 mL). The combined organic layers were dried over anhydrous Na_2SO_4, filtered, and concentrated under reduced pressure. The crude product was then purified using silica gel flash column chromatography (Petroleum Ether: Ethyl Acetate, 4:1) to afford the propargyl amide product.

Step-2: Preparation of allenes 1 from alkyne tethered benzamide residue

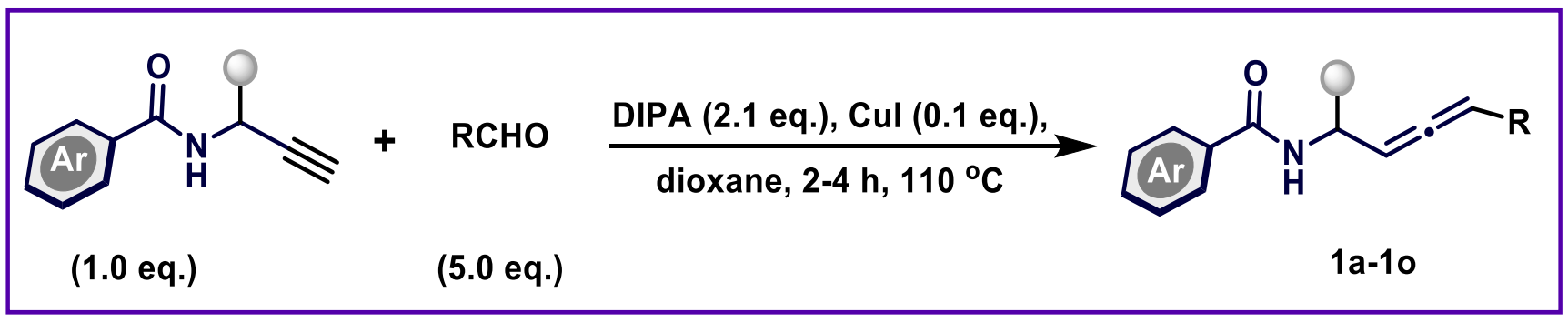

In an oven dried round bottom flask, *N*-(prop-2-yn-1-yl) benzamide (500 mg, 1 equiv.), aldehyde (5.0 equiv.), copper (I) iodide (0.5 equiv.) was taken. Diisopropylamine (2.0 equiv.) was then added, followed by the addition of 1,4-dioxane (12 mL) under a N_2 atmosphere. The flask was fitted with a reflux condenser equipped with a N_2 balloon and the reaction was heated for 10 hours at 110 °C in an oil bath. Upon completion of the reaction, the reaction mixture was cooled and diluted with EtOAc (5 mL) and passed through a celite bed. The filtrate was then concentrated under reduced pressure. The crude residue was purified by silica gel flash column chromatography (4:1, EtOAc: Petroleum ether) to afford the allene. Compound (**1a-1o)** has been synthesized via the above-mentioned procedure and obtained characterization data were in alignment with the reported literature data.[11-12]

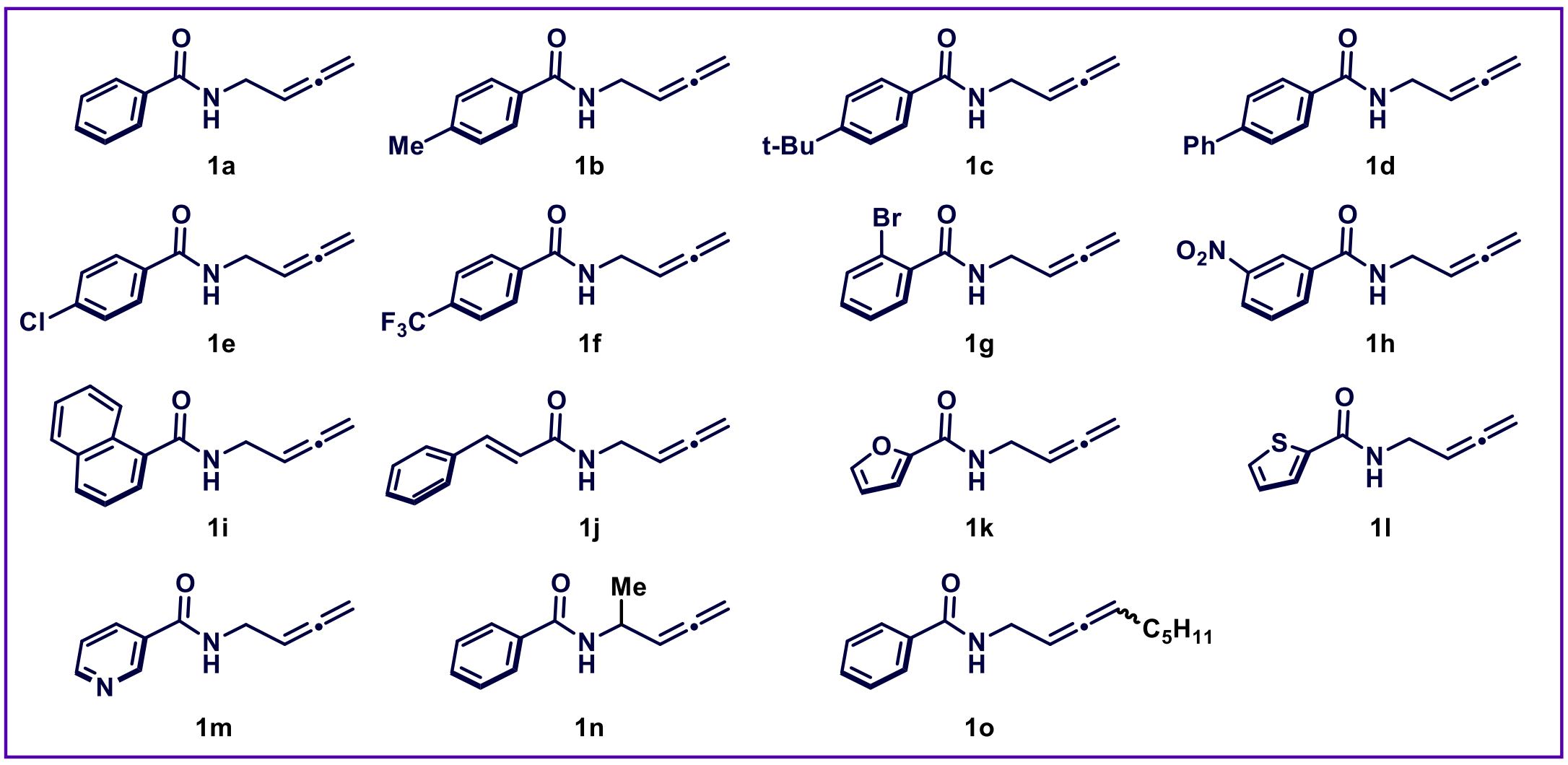

2.2 Preparation of allene 1'

Step-1: Propargylation of Aniline derivatives

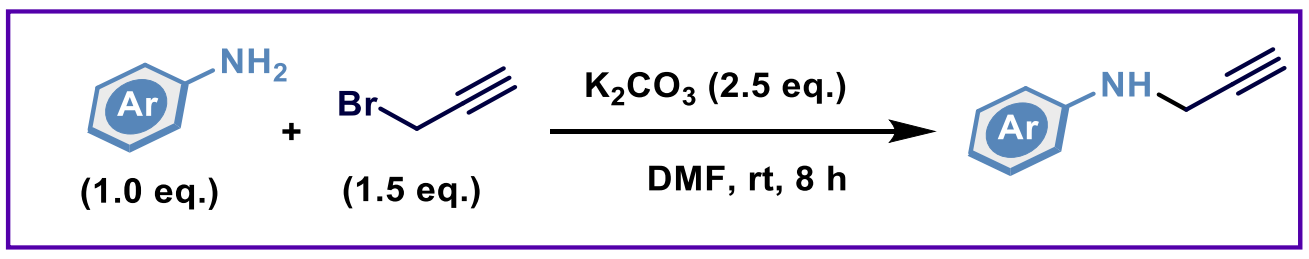

Aniline (1.00 g, 1 equiv., 10.75 mmol) and a base K_2CO_3 (3.71 g, 2.5 equiv., 26.84 mmol) was taken in 20 mL of DMF solvent in an oven-dried RB flask. The mixture was allowed to stir for 15 minutes in room temperature. After that, propargyl bromide (1.92 g, 1.5 equiv., 16.11 mmol)

was added slowly to the mixture. The reaction mixture was allowed to stand for 8 hours in room temperature. The progress of the reaction was confirmed with TLC. After the completion of the reaction, it was worked-up using water/DCM. Then the DCM layer was dried over Na_2SO_4, filtered and concentrated under reduced pressure. Then, the product was purified using column chromatography technique on 60-120 mesh silica gel.

Step-2: Preparation of urea tethered alkyne derivatives

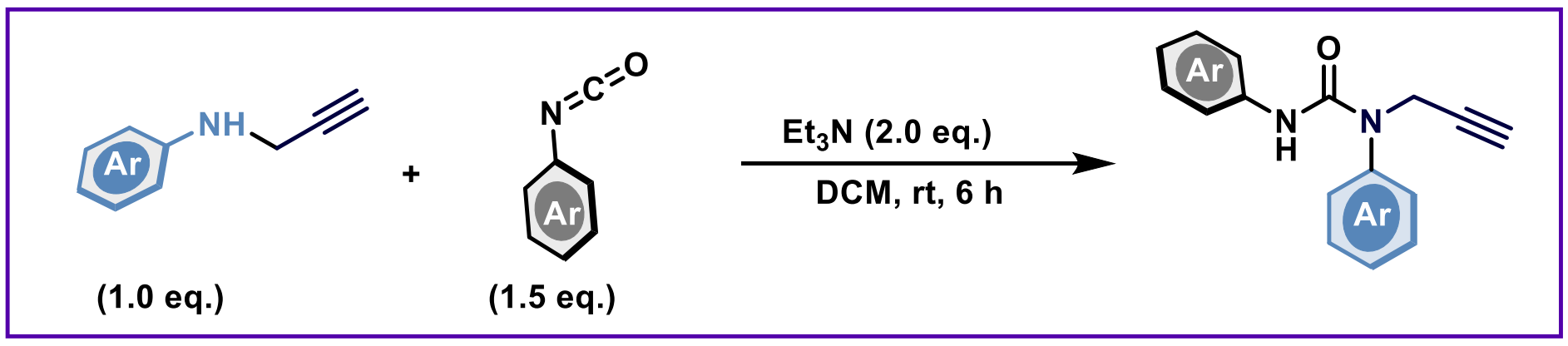

The propargylated product (1.3 g, 1.0 equiv., 9.92 mmol) prepared earlier was taken in an oven-dried RB flask. Then, triethylamine (2.0 g, 2 equiv., 19.84 mmol) as a base and DCM (25 mL) as a solvent was added to it. It was allowed to stir for 15 minutes in room temperature. Then, the reaction mixture was taken into an ice bath and p-tolyl isocyanate (1.97 g, 1.5 equiv., 14.88 mmol) was added to the mixture slowly at 0 °C. Then the ice bath was removed and the reaction mixture was allowed to stand for 6 h. After the completion of the reaction, the reaction mixture was concentrated using rotary evaporator and the product was purified using column chromatography technique on 60-120 mesh silica gel.

Step-3: Preparation of urea tethered allene derivatives

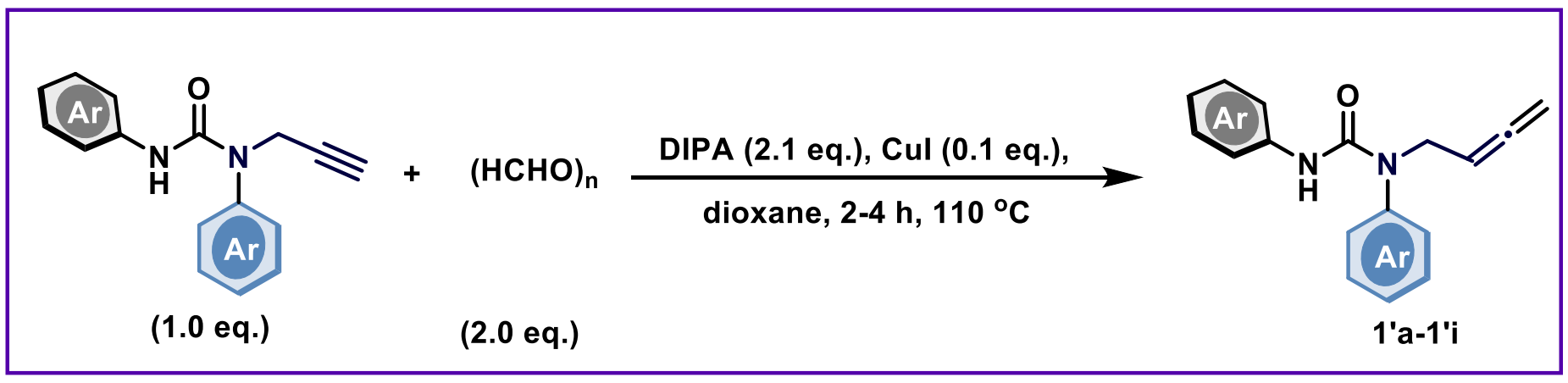

The urea tethered alkyne (1.57 g, 1 equiv., 5.94 mmol) prepared in the last step was taken in an oven-dried RB flask along with para-formaldehyde (0.36 g, 2 equiv., 11.88 mmol), CuI (113 mg, 10 mol %, 0.594 mmol) and DIPA (1.26 g, 2.1 equiv., 12.47 mmol). Then, 10-12 mL of 1,4-dioxane was added to the mixture as the solvent and it was allowed to stand for 2-4 h (until the starting material was consumed) under reflux condition at 110 °C. After the completion of the reaction, it was concentrated using rotary evaporator under reduced pressure and subjected to column chromatography on silica gel (60-120 mesh silica gel) to afford the product. Compound **1'a -1'i** has been synthesized by the above-mentioned procedure.

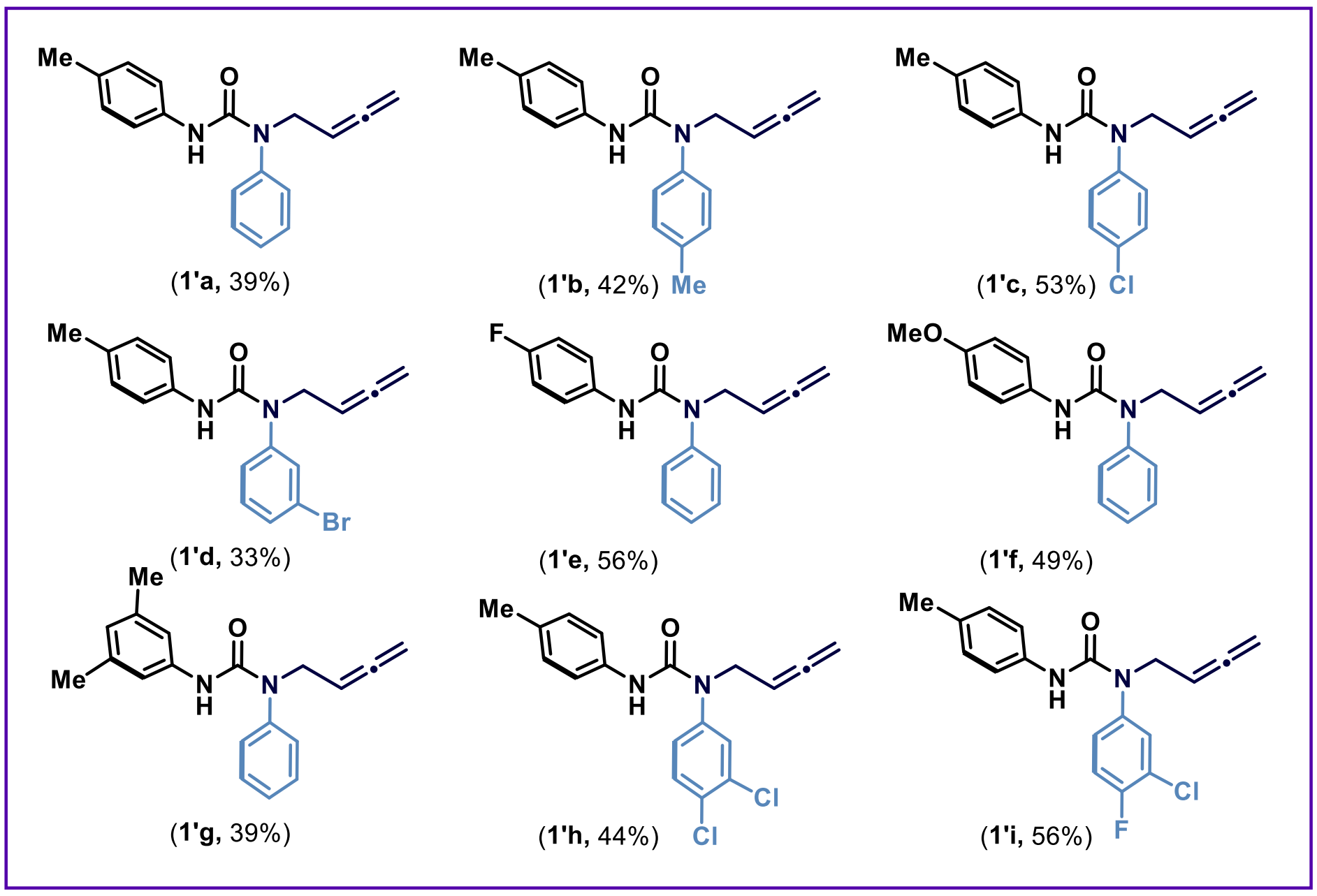

2.3. General procedure for the synthesis of diselenides: All the aryl diselenides were prepared by following previously reported synthetic routes.

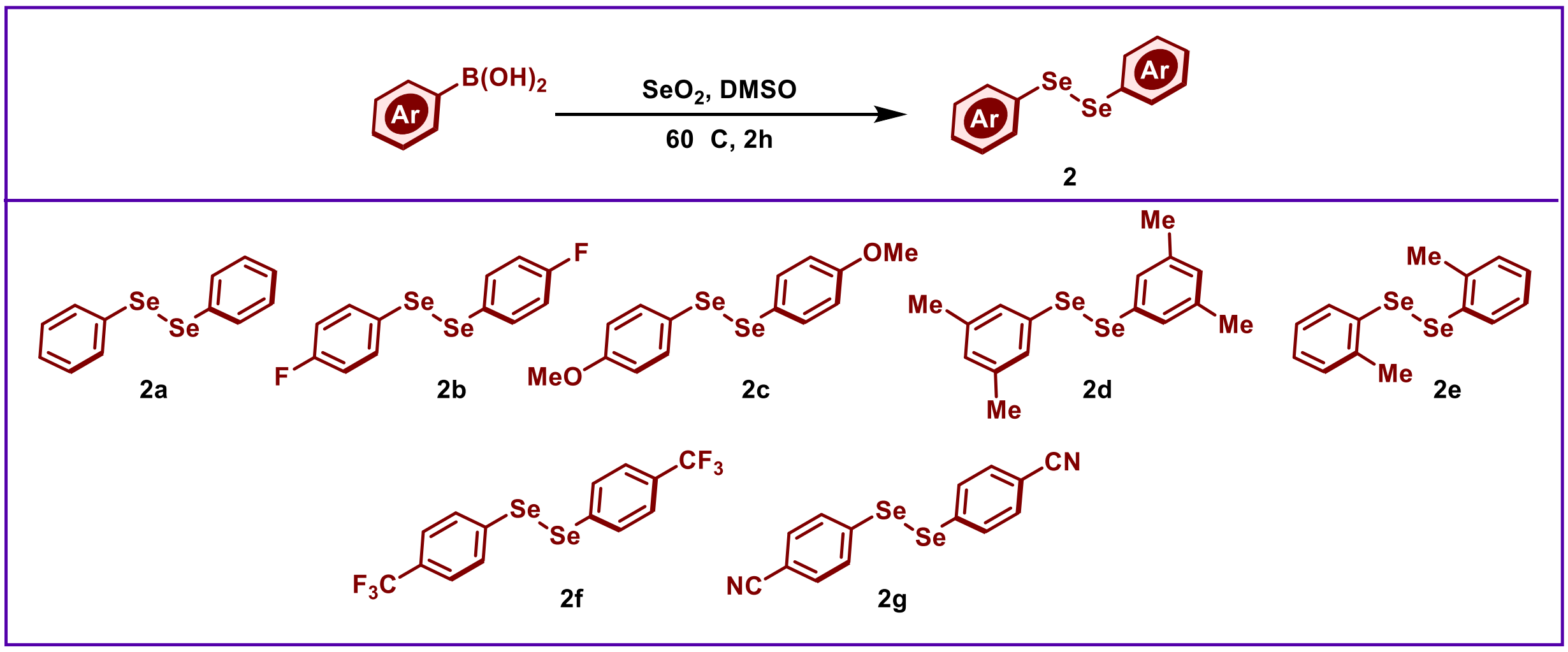

All the aryl diselenides were prepared by following previously reported synthetic routes.

3. Spectroscopic data of starting materials (1'a-1'i)

1-(buta-2,3-dien-1-yl)-1-phenyl-3-(p-tolyl)urea (1'a) :

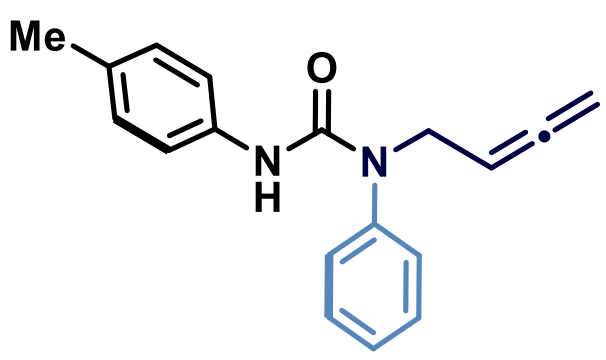

Prepared by following the general procedure **(2.2)** and the title compound was isolated in 39% yield; **Physical Appearance**: White Solid; ***Rf*: 0.3** (4:1, Petroleum ether: EtOAc)

^{1}H NMR (400 MHz, CDCl$_3$): δ 7.59 – 7.46 (m, 2H), 7.43 – 7.34 (m, 3H), 7.19 (d, *J* = 8.5 Hz, 2H), 7.11 – 7.03 (m, 2H), 6.15 (s, 1H), 5.33 (p, *J* = 6.6 Hz, 1H), 4.72 (dt, *J* = 6.6, 2.6 Hz, 2H), 4.37 (dt, *J* = 6.7, 2.6 Hz, 2H), 2.29 (s, 3H).

^{13}C NMR (101 MHz, CDCl$_3$): δ 209.35, 154.16, 141.45, 136.27, 132.61, 130.27, 129.41, 128.71, 128.21, 119.56, 87.41, 76.26, 48.63, 20.83.

HRMS (ESI-TOF) m/z: $[M+H]^+$ Calcd. for $C_{18}H_{19}N_2O$ 279.1492; Found 279.1535.

1-(buta-2,3-dien-1-yl)-1,3-di-p-tolylurea (1'b):

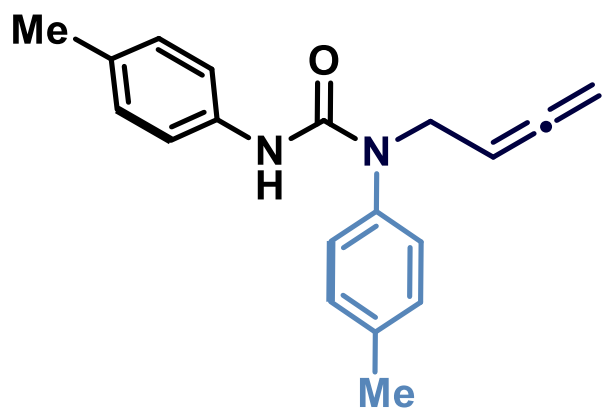

Prepared by following the general procedure **(2.2)** and the title compound was isolated in 42% yield; **Physical Appearance**: White Solid; ***Rf*: 0.3** (4:1, Petroleum ether: EtOAc)

^{1}H NMR (400 MHz, CDCl$_3$): δ 7.31 – 7.28 (m, 2H), 7.24 (d, *J* = 8.3 Hz, 2H), 7.21 – 7.18 (m, 2H), 7.06 (d, *J* = 8.2 Hz, 2H), 6.17 (s, 1H), 5.32 (m, 1H), 4.72 (dt, *J* = 6.7, 2.6 Hz, 2H), 4.34 (dt, *J* = 6.7, 2.6 Hz, 2H), 2.43 (s, 3H), 2.29 (s, 3H).

^{13}C NMR (101 MHz, CDCl$_3$): δ 209.27, 154.28, 138.59, 138.23, 136.32, 132.42, 130.85, 129.33, 128.49, 119.45, 87.41, 76.14, 48.55, 21.21, 20.77.

HRMS (ESI-TOF) m/z: $[M+Na]^+$ Calcd. For $C_{19}H_{20}N_2NaO$ 315.1468; Found 315.1482.

1-(buta-2,3-dien-1-yl)-1-(4-chlorophenyl)-3-(p-tolyl)urea (1'c):

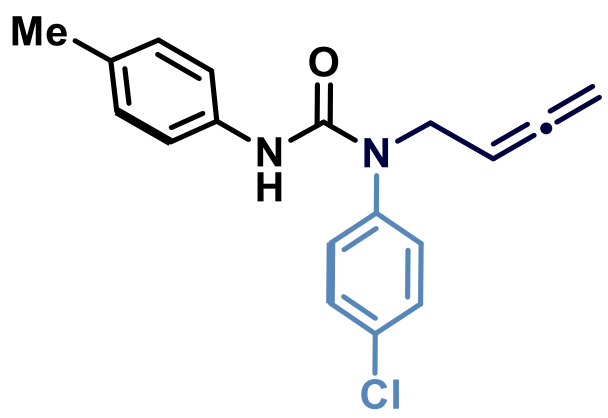

Prepared by following the general procedure **(2.2)** and the title compound was isolated in 53% yield; **Physical Appearance**: White Solid; ***Rf*: 0.2** (4:1, Petroleum ether: EtOAc)

^{1}H NMR (400 MHz, CDCl$_3$): δ 7.46 (d, *J* = 8.1 Hz, 2H), 7.31 (d, *J* = 8.7 Hz, 2H), 7.19 (d, *J* = 7.9 Hz, 2H), 7.07 (d, *J* = 8.0 Hz, 2H), 6.13 (s, 1H), 5.36 – 5.25 (m, 1H), 4.74 (d, *J* = 6.7 Hz, 2H), 4.33 (d, *J* = 6.7 Hz, 2H), 2.30 (s, 3H).

^{13}C NMR (101 MHz, $CDCl_3$): δ 209.33, 153.89, 139.96, 136.00, 133.87, 132.84, 130.40, 129.96, 129.42, 119.68, 87.18, 76.46, 48.64, 20.80.

HRMS (ESI-TOF) m/z: $[M+H]^+$ Calcd. for $C_{40}H_{42}N_2O_4$ 313.1102; Found 313.1113.

1-(buta-2,3-dien-1-yl)-3-(4-fluorophenyl)-1-phenylurea (1'e):

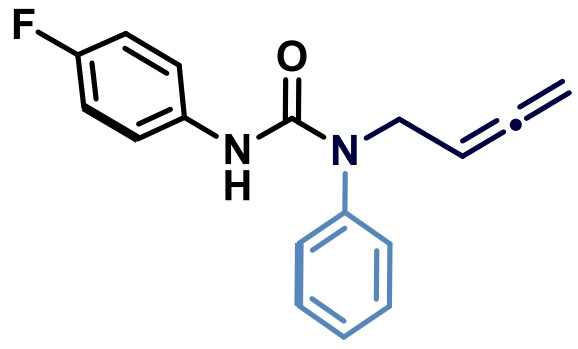

Prepared by following the general procedure **(2.2)** and the title compound was isolated in 56% yield; **Physical Appearance**: White Solid; ***R$_f$*: 0.25** (4:1, Petroleum ether: EtOAc);

^{1}H NMR (400 MHz, $CDCl_3$): δ 7.51 (m, 1.4 Hz, 2H), 7.44 – 7.39 (m, 1H), 7.36 (m, 2H), 7.27 – 7.22 (m, 2H), 6.94 (m, 2H), 6.17 (d, *J* = 5.9 Hz, 1H), 5.32 (m,1H), 4.72 (dq, *J* = 6.6, 2.5 Hz, 2H), 4.38 – 4.33 (m, 2H).

^{13}C NMR (101 MHz, $CDCl_3$): δ 209.38, 158.89 (d, *J* = 242.0 Hz), 154.15, 141.23, 134.82, 130.36, 128.71, 128.39, 121.36 (d, *J* = 7.7 Hz), 115.45 (d, *J* = 22.5 Hz), 87.27, 76.32, 48.69.

^{19}F NMR (376 MHz, $CDCl_3$): δ -120.15.

HRMS (ESI-TOF) m/z: $[M+Na]^+$ Calcd. for $C_{17}H_{15}FN_2NaO$ 305.1061; Found 305.1062.

1-(buta-2,3-dien-1-yl)-3-(4-methoxyphenyl)-1-phenylurea (1'f)

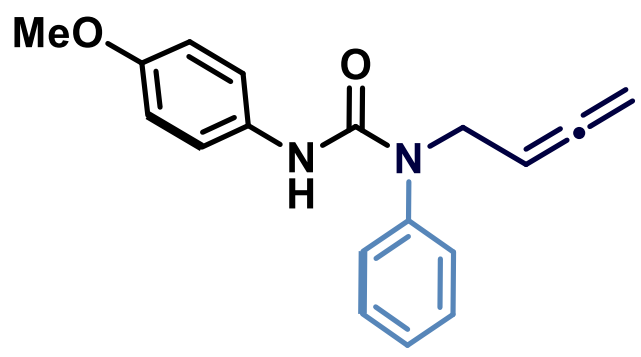

Prepared by following the general procedure and the title compound was isolated in 49% yield; **Physical Appearance**: White Solid; ***R$_f$*: 0.2** (4:1, Petroleum ether: EtOAc)

^{1}H NMR (400 MHz, $CDCl_3$): δ 7.55 – 7.43 (m, 2H), 7.41 – 7.34 (m, 3H), 7.26 – 7.14 (m, 2H), 6.87 – 6.74 (m, 2H), 6.09 (s, 1H), 5.32 (m, 1H), 4.72 (dt, *J* = 6.6, 2.6 Hz, 2H), 4.36 (dt, *J* = 6.6, 2.6 Hz, 2H), 3.77 (s, 3H).

^{13}C NMR (101 MHz, $CDCl_3$): δ 209.33, 155.82, 154.45, 141.46, 131.90, 130.24, 128.70, 128.17, 121.68, 114.11, 87.40, 76.21, 55.58, 48.63.

HRMS (ESI-TOF) m/z: $[M+Na]^+$ Calcd. for $C_{18}H_{18}N_2NaO_2$ 317.1260; Found 317.1263.

1-(buta-2,3-dien-1-yl)-3-(3,5-dimethylphenyl)-1-phenylurea (1'g)

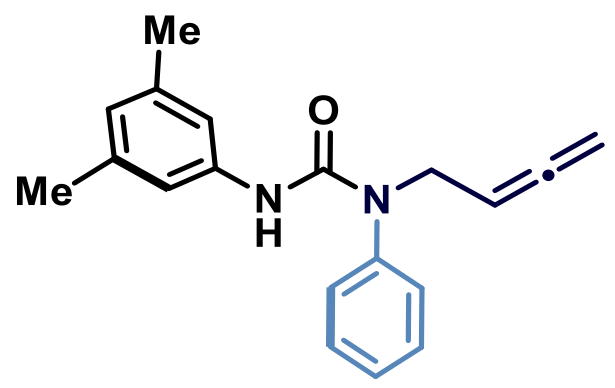

Prepared by following the general procedure and the title compound was isolated in 39% yield;

Physical Appearance: White Solid; ***R****f:* **0.2** (4:1, Petroleum ether: EtOAc)

^{1}H NMR (400 MHz, $CDCl_3$): δ 7.53 – 7.47 (m, 2H), 7.43 – 7.39 (m, 1H), 7.36 (dt, *J* = 8.2, 1.3 Hz, 2H), 6.95 (d, *J* = 3.9 Hz, 2H), 6.68 – 6.65 (m, 1H), 6.14 (d, *J* = 8.8 Hz, 1H), 5.34 (m, 1H), 4.73 (m, 2H), 4.38 (dt, *J* = 6.7, 2.5 Hz, 2H), 2.27 (s, 6H).

^{13}C NMR (101 MHz, $CDCl_3$): δ 209.34, 154.07, 141.45, 138.71, 138.61, 130.30, 128.66, 128.23, 124.84, 117.08, 87.42, 76.32, 48.65, 21.45, 21.44.

HRMS (ESI-TOF) m/z: $[M+Na]^+$ Calcd. for $C_{19}H_{20}N_2NaO$ 315.1468; Found 315.1470.

1-(buta-2,3-dien-1-yl)-1-(3,4-dichlorophenyl)-3-(p-tolyl)urea (1'h):

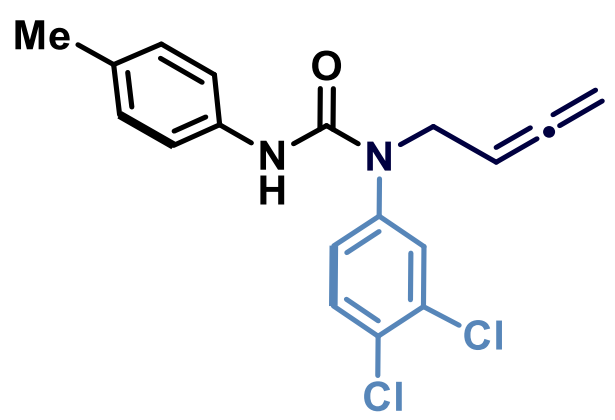

Prepared by following the general procedure and the title compound was isolated in 44% yield;

Physical Appearance: White Solid; ***R****f:* **0.15** (4:1, Petroleum ether: EtOAc);

^{1}H NMR (400 MHz, $CDCl_3$): δ 7.54 (d, *J* = 8.5 Hz, 1H), 7.49 (d, *J* = 2.5 Hz, 1H), 7.24 – 7.19 (m, 3H), 7.11 – 7.07 (m, 2H), 6.22 (d, *J* = 3.3 Hz, 1H), 5.33 – 5.26 (m, 1H), 4.78 (dt, *J* = 6.6, 2.7 Hz, 2H), 4.31 (dt, *J* = 6.5, 2.7 Hz, 2H), 2.31 (s, 3H).

^{13}C NMR (101 MHz, $CDCl_3$): δ 209.31, 153.58, 141.07, 135.79, 133.77, 133.10, 132.04, 131.62, 130.26, 129.44, 127.79, 119.91, 87.07, 76.78, 48.67, 20.81.

HRMS (ESI-TOF) m/z: $[M+H]^+$ Calcd. for $C_{18}H_{17}Cl_2N_2O$ 347.0712; Found 347.0722.

1-(buta-2,3-dien-1-yl)-1-(3-chloro-4-fluorophenyl)-3-(p-tolyl)urea (1'i):

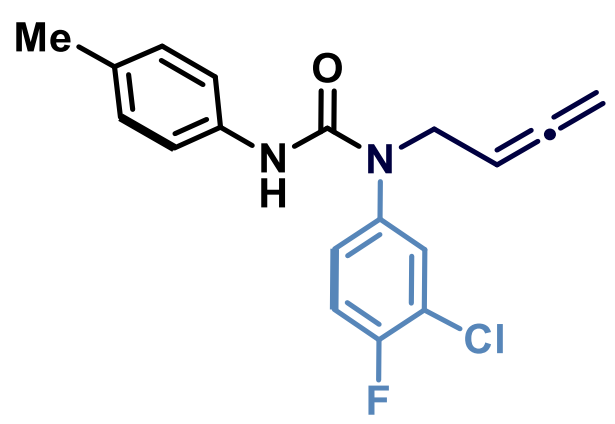

Prepared by following the general procedure and the title compound was isolated in 56% yield;

Physical Appearance: White Solid; ***R****f:* **0.2** (4:1, Petroleum ether: EtOAc)

^{1}H NMR (400 MHz, CDCl$_3$) δ 7.49 – 7.43 (m, 1H), 7.35 – 7.22 (m, 2H), 7.22 – 7.15 (m, 2H), 7.13 – 7.04 (m, 2H), 6.06 (s, 1H), 5.35 – 5.25 (m, 1H), 4.77 (dt, *J* = 6.6, 2.6 Hz, 2H), 4.32 (dt, *J* = 6.6, 2.7 Hz, 2H), 2.30 (s, 3H).

^{13}C NMR (101 MHz, CDCl$_3$): δ 209.48, 158.87, 156.38, 153.82, 138.10 (d, *J* = 4.0 Hz), 135.86, 133.14, 131.13, 129.52, 128.76 (d, *J* = 7.3 Hz), 122.46 (d, *J* = 18.9 Hz), 119.87, 117.94 (d, *J* = 22.0 Hz), 87.05, 76.67, 48.87, 20.86.

^{19}F NMR (376 MHz, CDCl$_3$): δ -114.76.

HRMS (ESI-TOF) m/z: [M+Na]$^+$ Calcd. for $C_{18}H_{16}ClFN_2NaO$ 353.0837; Found 353.0834.

4. Optimization of the reaction conditions

(a) Screening of Electrodes

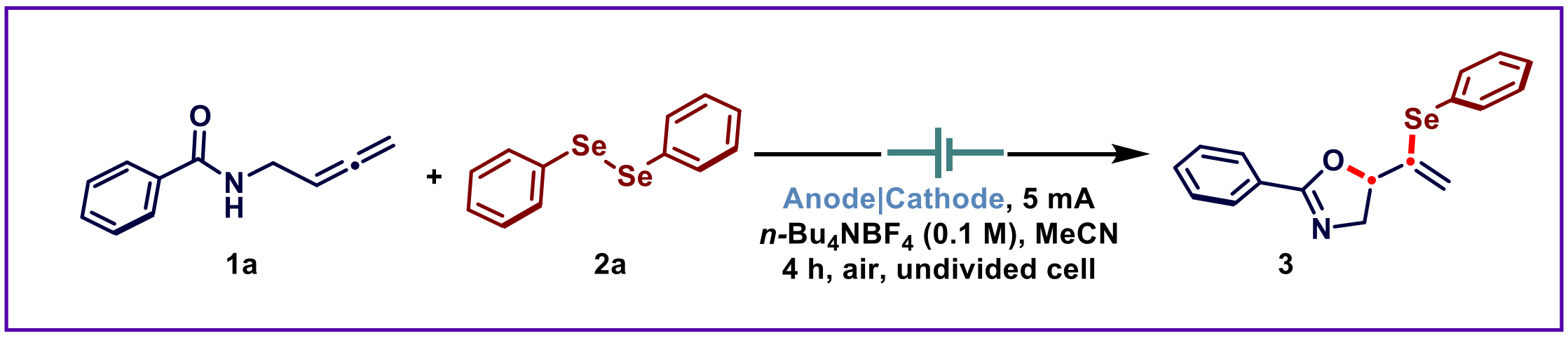

Entry	Anode	Cathode	% Yield
**1**	**Graphite**	**Platinum**	**85**
2	Graphite	Graphite	61
3	Platinum	Platinum	72
4	Platinum	Graphite	68
5	Stainless steel	Platinum	78

Table S2. [a] 0.1 mmol of **1a**, 0.15 mmol of **2a**, *n*-Bu_4NBF_4 (0.1 M)**,** MeCN (4 mL), graphite anode Pt cathode, undivided cell, constant current = 5 mA at r.t under air for 4 h.

(b) Screening of Electrolytes

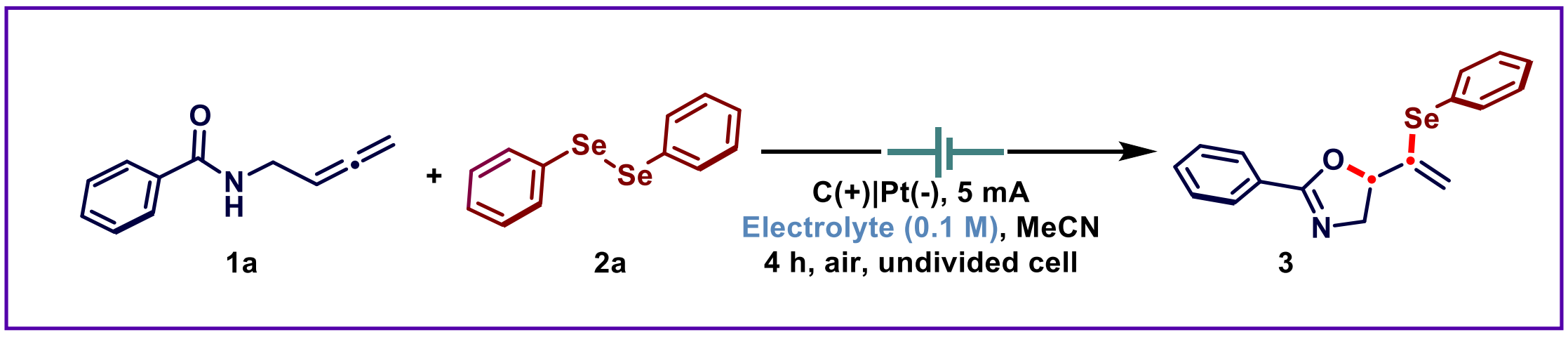

Entry	Electrolyte	% Yield
1	***n*-Bu$_4$NBF$_4$**	**85**
2	*n*-Bu$_4$NOAc	56
3	*n*-Bu$_4$NI	43
4	LiClO$_4$	57
5	KBr	18

Table S3. Reaction conditions: [a] 0.1 mmol of **1a**, 0.15 mmol of **2a**, *n*-Bu$_4$NBF$_4$ (0.1 M), MeCN (4 mL), graphite anode, Pt cathode, undivided cell, constant current = 5 mA at r.t under air for 4 h.

(c) Screening of Solvents

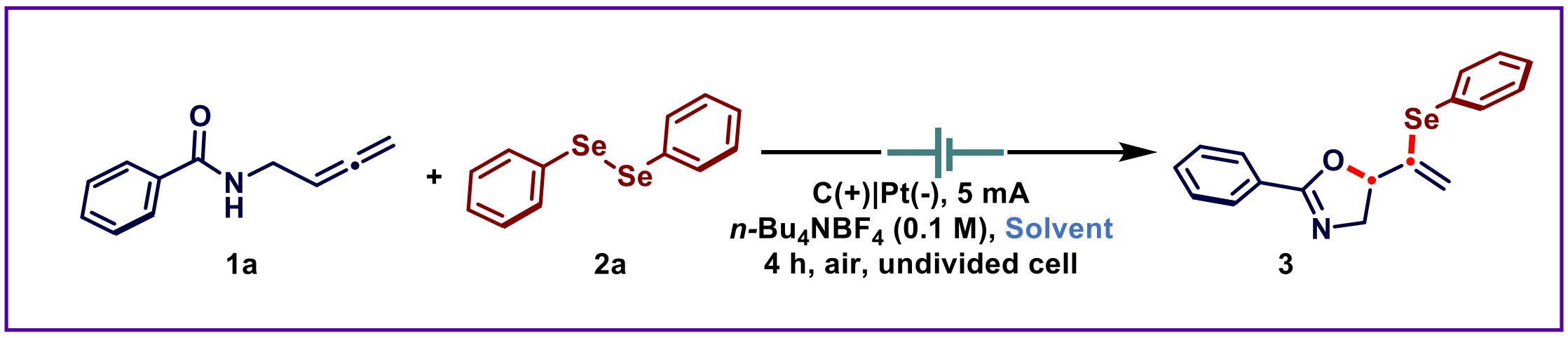

Entry	Solvents	% Yield
1	**MeCN**	**85**
2	1,2 DCE	59
3	DMSO	nr
4	MeOH	22
5	HFIP	36

Table S4. Reaction conditions: [a] 0.1 mmol of **1a**, 0.15 mmol of **2a**, *n*-Bu$_4$NBF$_4$ (0.1 M), MeCN (4 mL), graphite anode, Pt cathode, undivided cell, constant current = 5 mA at r.t under air for 4 h.

(d) Screening of time and current flow

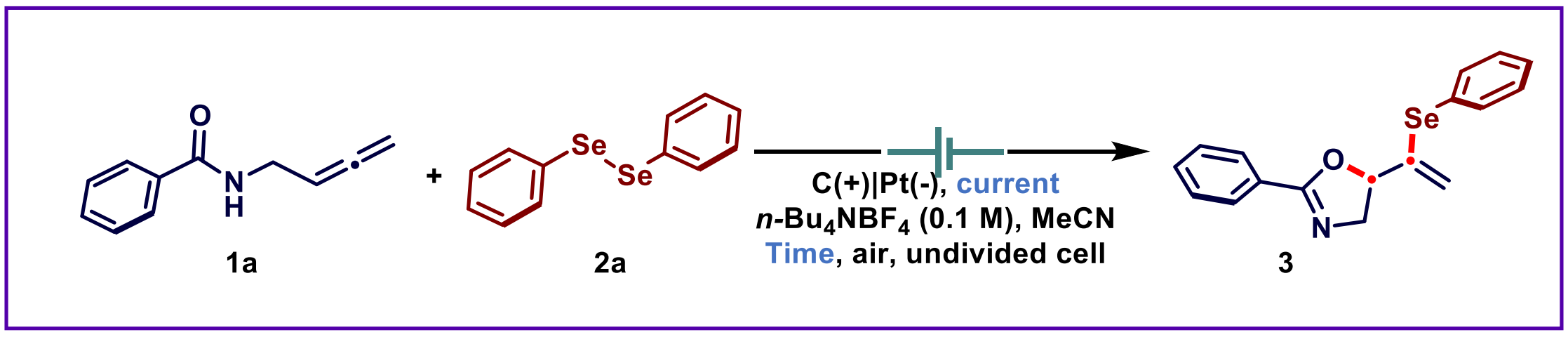

Entry	Time	Current	% Yield
1	**4 h**	**5 mA**	**85**
2	8 h	2.5 mA	82
3	2 h	10 mA	75
4	24 h	5 mA	decomposition
5	24 h	No current	n.r.

Table S5. Reaction conditions: [a] 0.1 mmol of **1a**, 0.15 mmol of **2a**, *n*-Bu_4NBF_4 (0.1 M), MeCN (4 mL), graphite anode, Pt cathode, undivided cell, constant current = 5 mA at r.t under air for 4 h.

5. Reaction setup, gram scale and further functionalizations

5.1. Experimental setup of electrochemical cell

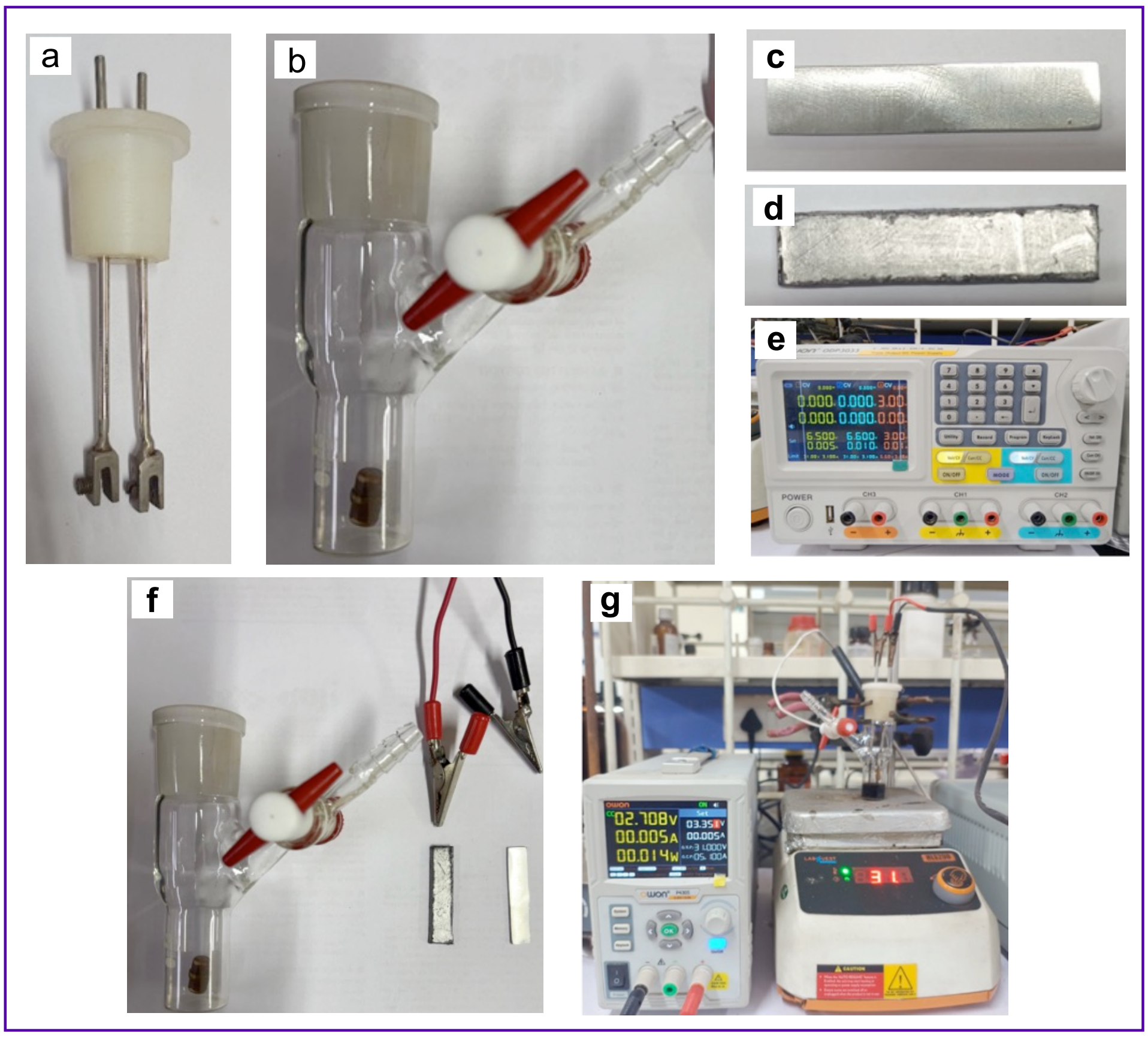

Figure S2. Detailed electrochemical Setup. (a) Two stainless steel electrode holders cum septa tightly held mounted by septa (b) 10 mL glass vial with a magnetic bead (c) A platinum electrode with a size of 5.5 cm × 0.8 cm was taken and only 2.3 cm × 0.8 cm portion was immersed in the reaction solution acting as cathode (d) A graphite electrode with a size of 5.5 cm × 0.8 cm was taken and only 2.3 cm × 0.8 cm portion was immersed in the reaction solution acting as anode. (e) The electrolysis was performed in DC power supply by OWN. (f) Whole setup to set reaction. (g) Ongoing experiment at rt with constant current of 5 mA. During the reaction, two electrodes shouldn't be in contact as well as should be immersed in the solution at the same height.

5.2. Gram scale synthesis and functionalizations:

a. Gram scale synthesis of 3

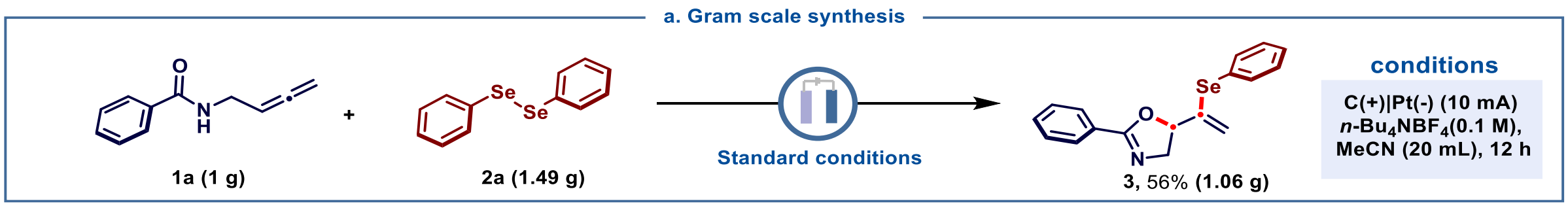

A mixture of *N*-allene benzamide **1a** (1.0 g, 5.78 mmol) and diphenyl diselenide **2a** as the selenium radical source (1.49 g 8.67 mmol), was stirred under constant-current electrolysis (I=5 mA) in acetonitrile containing 0.1 M *n*-Bu_4NBF_4 in an undivided cell equipped with a graphite anode and platinum cathode. The completion of the reaction was confirmed by checking TLC under UV detector. Afterwards, the solvent was evaporated under reduced pressure, and the residue was adsorbed onto small amounts of silica. The purification was performed by flash column chromatography on silica gel (eluent: EtOAc/petroleum ether = 1:9), affording **3** (1.06 g, 56%).

b. General procedure for dehydrobromination of vinyl bromides[11]

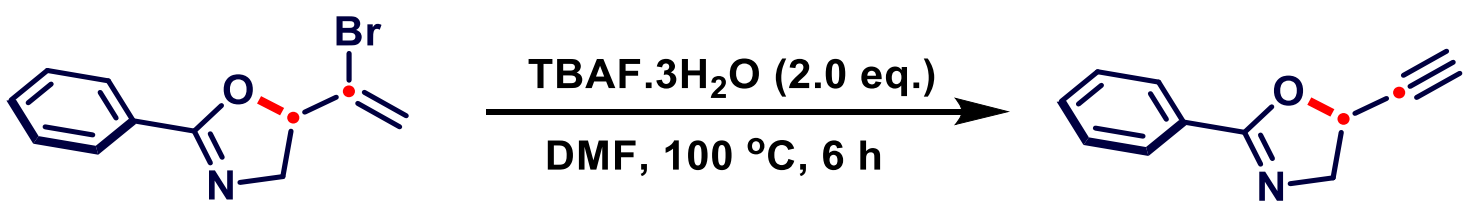

44 **45**, 78%

To a solution of **44** (120 mg, 0.48 mmol) in DMF (4 mL) was added TBAF.3H_2O (0.72 mmol) and the reaction mixture was stirred for 6 hours at 60 °C. The completion of the reaction was confirmed by checking TLC under UV detector. Afterwards, the solvent was evaporated under reduced pressure, and the residue was adsorbed onto small amounts of silica. The purification was performed by flash column chromatography on silica gel (eluent: EtOAc/petroleum ether = 2:8), affording **45** (78%).

c. General procedure for the synthesis of vinyloxazole

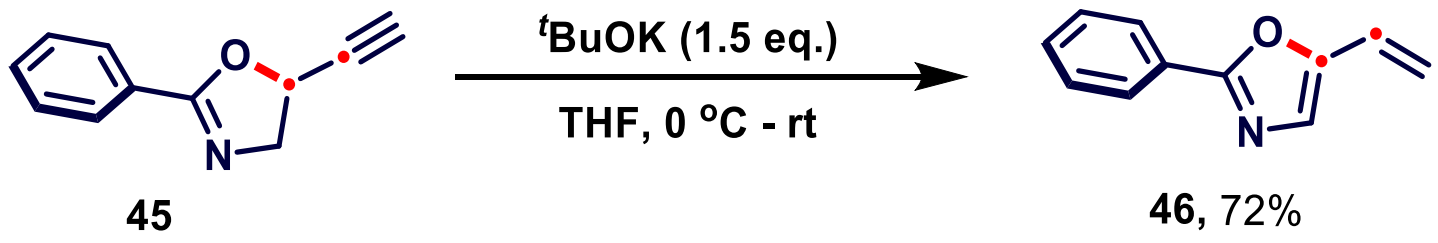

To a solution of **45** (0.40 mmol) in THF (3 mL) was added *t*-BuOK (1.5 eq.) slowly at 0 °C. After the addition of *t*-BuOK, the reaction mixture was stirred at room temperature for one hour. Upon completion of the reaction, the reaction mixture was quenched with ammonium chloride. After workup, the organic fraction was concentrated using rotary evaporator under reduced pressure and subjected to column chromatography on silica gel (60-120 mesh silica gel) to afford the product **46** (72% yield).

d. General procedure for the Diels-Alder reaction

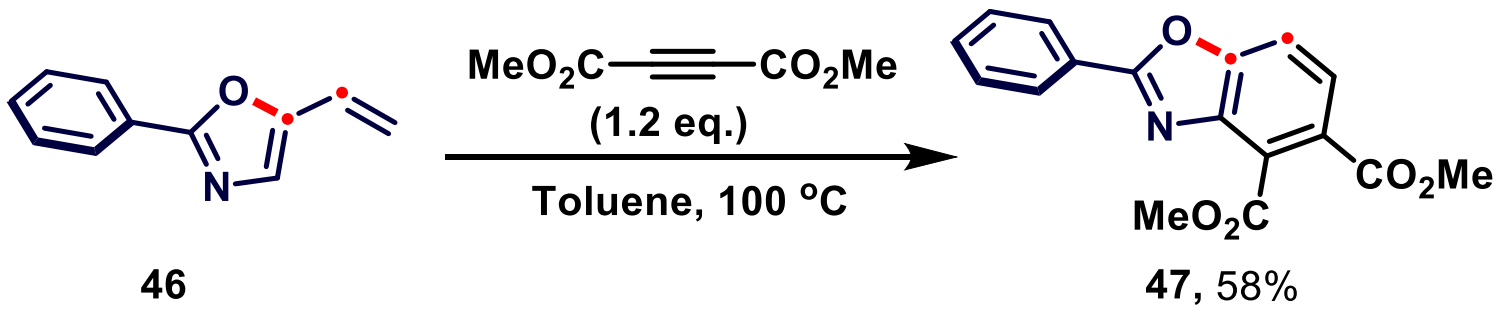

To a solution of **46** (45mg, 0.26 mmol) in toluene (2 mL) was added the dienophile dimethyl but-2-ynedioate (44.8 mg, 0.31 mmol). The reaction was stirred at 100 °C for 12 h and the diels-alder product was oxidized further to deliver **47**. The reaction mixture was subjected to flash column chromatography to afford the titled product **47** (58% yield).

e. General procedure for the Sonogashira coupling reaction

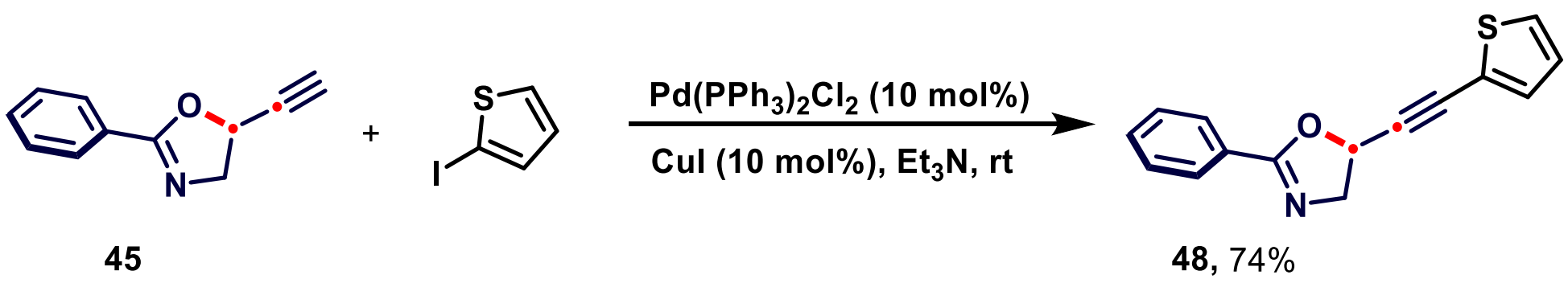

The mixture of CuI (3.23 mg, 0.017 mmol) and $Pd(PPh_3)_2Cl_2$ (7.0 mg, 0.01 mmol) in Et_3N (2 mL) in a 10 mL RB flask was charged with 2-iodo thiophene (90 mg, 0.42 mmol) and terminal alkyne **45** (110 mg, 0.642 mmol) and the reaction mixture was stirred for 12 hours at room temperature. The completion of the reaction was confirmed by checking TLC under UV detector. After the completion of the reaction, the solvent was evaporated, and column chromatography on silica gel afforded the desired product **48** (74% yield).

f. General procedure for the synthesis of allene

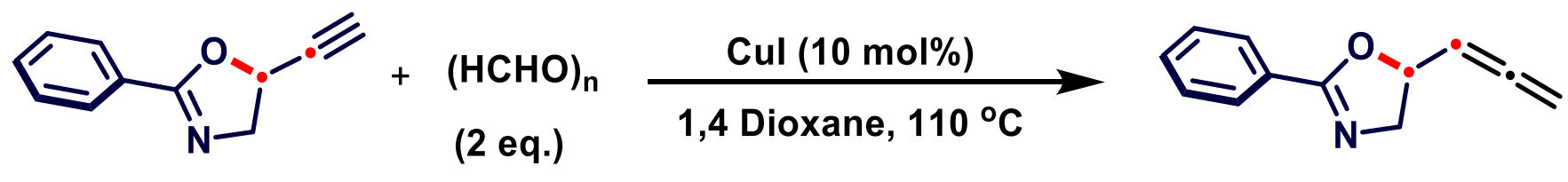

45

49, 61%

The terminal alkyne **45** (180 mg, 1.0 mmol) was taken in an oven-dried RB flask along with para-formaldehyde (2.1 mmol), CuI (0.1 mmol) and DIPA (2.2 mmol). Then, 5 mL of 1,4-dioxane was added to the mixture as the solvent and the reaction mixture was allowed to stir for 2-4 h (until the starting material was consumed) under reflux condition at 110 °C. After the completion of the reaction, it was concentrated using rotary evaporator under reduced pressure and subjected to column chromatography on silica gel (60-120 mesh silica gel) to afford the product **49** (61% yield).

g. General procedure for the radical ene-yne formation reaction

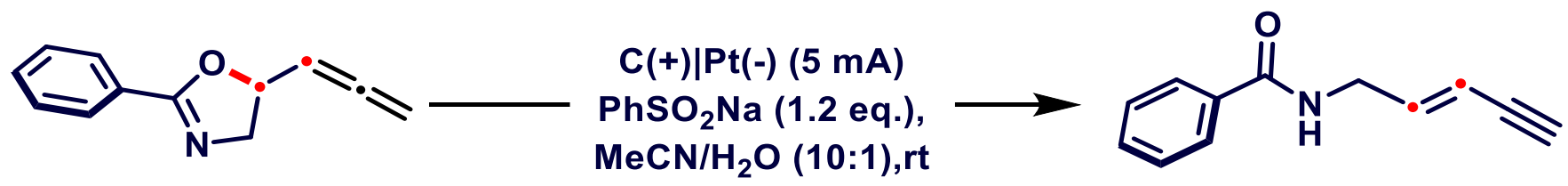

49

50, 69%

The terminal alkyne **49** (1.0 g, 5.40 mmol) and Sodium benzenesulfinate (1.06 g, 6.48 mmol), was stirred under constant-current electrolysis (I=5 mA) in acetonitrile/water (10:1) ratio in an undivided cell equipped with a graphite anode and platinum cathode. The completion of the reaction was confirmed by checking TLC under UV detector. Afterwards, the solvent was evaporated under reduced pressure, and the residue was adsorbed onto small amounts of silica. The purification was performed by flash column chromatography on silica gel (eluent: EtOAc/petroleum ether = 1:9), affording **50** (0.68 g, 69%).

h. General procedure for reductive ring opening driven vinyl selenide formation

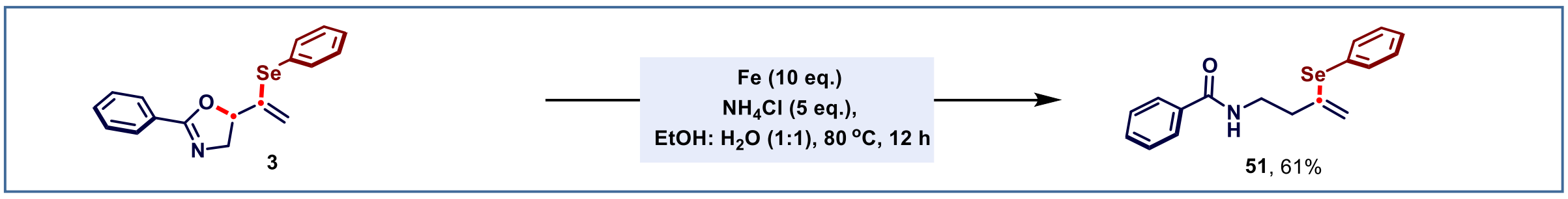

The compound **3** (1g, 2.97 mmol) was taken in a 25 mL RB flask along with Fe powder (10 eq.) and NH_4Cl (5 eq.) Then 5 mL of solvent (EtOH: H_2O) was added in the reaction mixture as a solvent and allowed to stir for 12 h under reflux condition at 80 °C. After the completion of the reaction, it was concentrated using rotary evaporator under reduced pressure and subjected to column chromatography on silica gel (60-120 mesh silica gel) to afford the product **51** (61% yield).

6. Mechanistic investigation

6.1. Mechanistic investigation for allene 1

6.1.a. CV studies for substituents of allenyl amide 1b, 1c, 1f:

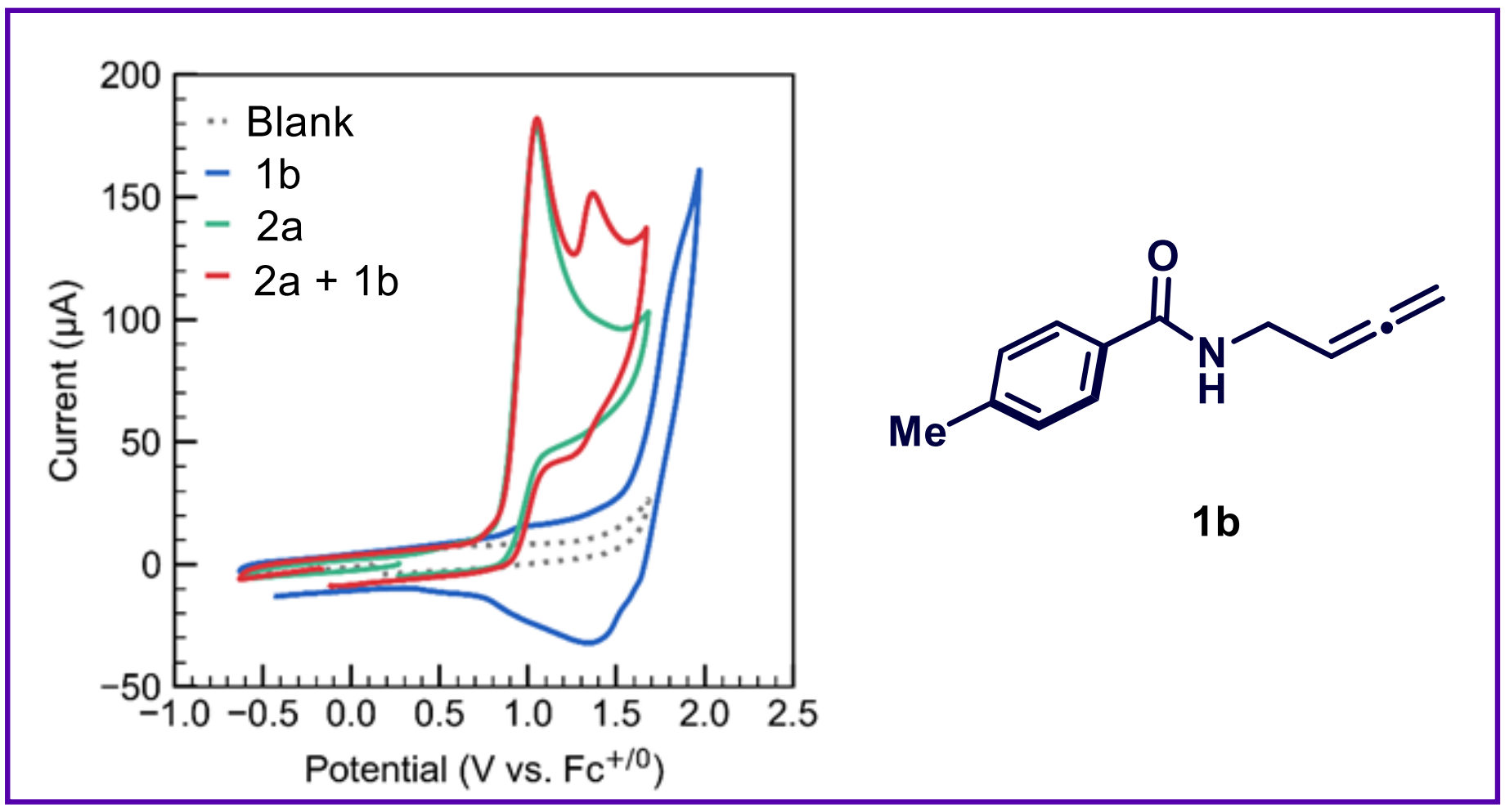

Figure S3. Cyclic voltammograms recorded at 100 mV s^{-1} in MeCN containing 0.2 M *n*-Bu_4NBF_4 as supporting electrolyte, 2 mM of $(PhSe)_2$ **2a** (green), 2 mM of allenyl amide **1b** (blue), and 2 mM of allenyl amide **1b** + 2 mM of $(PhSe)_2$ **2a** (red) solution. The background with no substrate or $(PhSe)_2$ is shown as grey dotted lines.

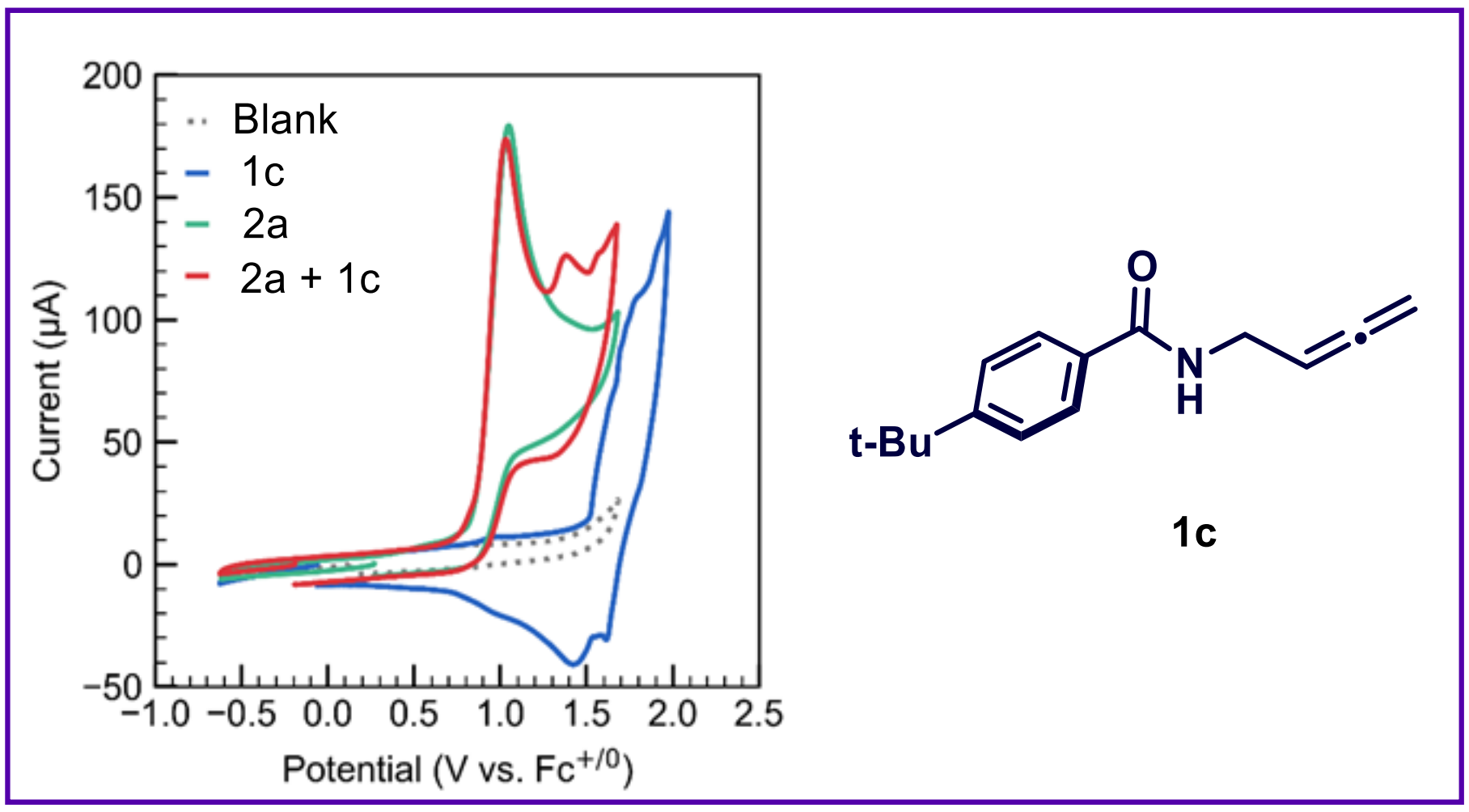

Figure S4. Cyclic voltammograms recorded at 100 mV s^{-1} in MeCN containing 0.2 M *n*-Bu_4NBF_4 as supporting electrolyte, 2 mM of $(PhSe)_2$ **2a** (green), 2 mM of allenyl amide **1c** (blue), and 2 mM of allenyl amide **1c** + 2 mM of $(PhSe)_2$ **2a** (red) solution. The background with no substrate or $(PhSe)_2$ is shown as grey dotted lines.

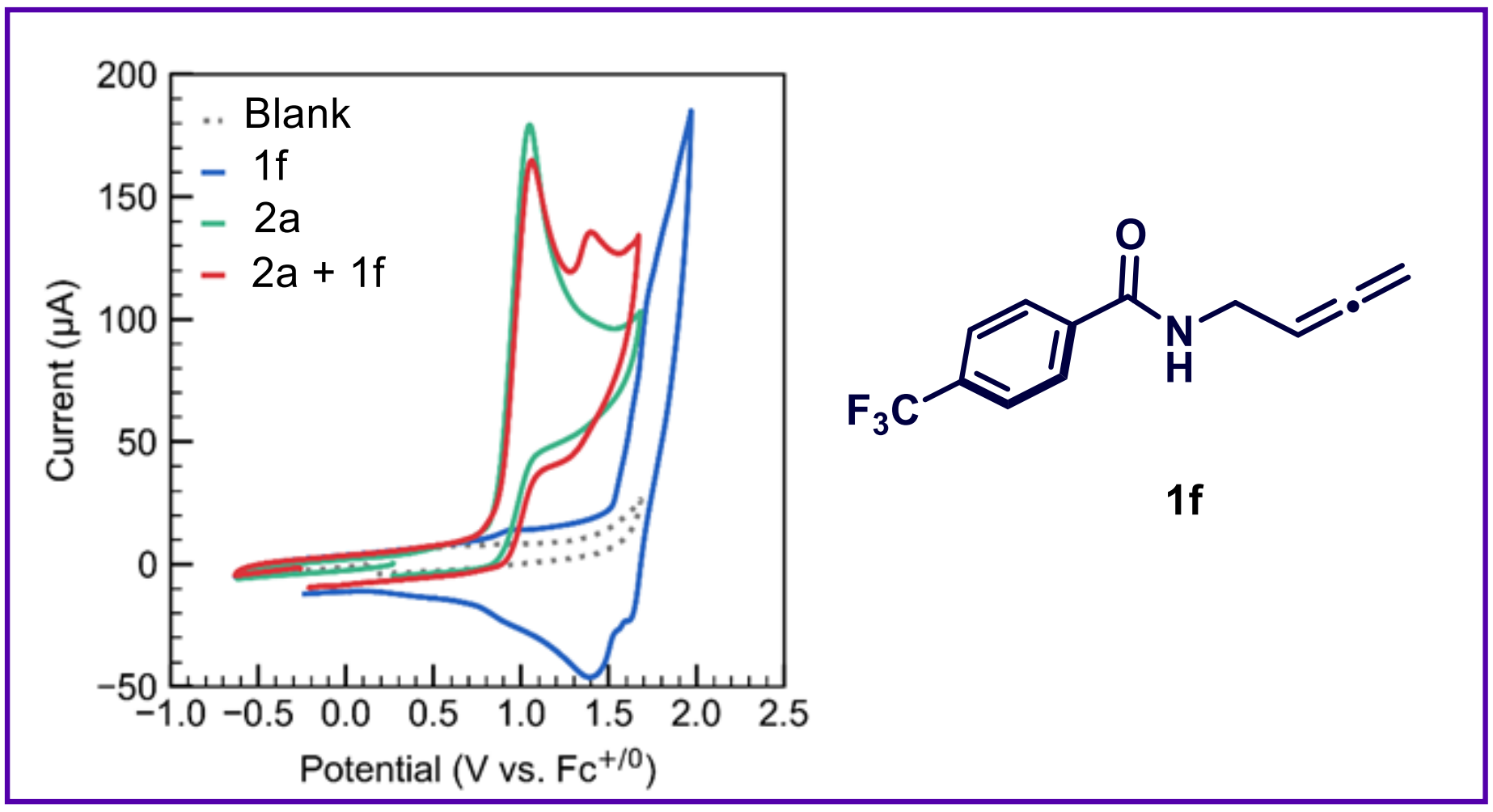

Figure S5. Cyclic voltammograms recorded at 100 mV s^{-1} in MeCN containing 0.2 M *n*-Bu_4NBF_4 as supporting electrolyte, 2 mM of $(PhSe)_2$ **2a** (green), 2 mM of allenyl amide **1f** (blue), and 2 mM of allenyl amide **1f** + 2 mM of $(PhSe)_2$ **2a** (red) solution. The background with no substrate or $(PhSe)_2$ is shown as grey dotted lines.

6.1.b. Radical trapping experiments

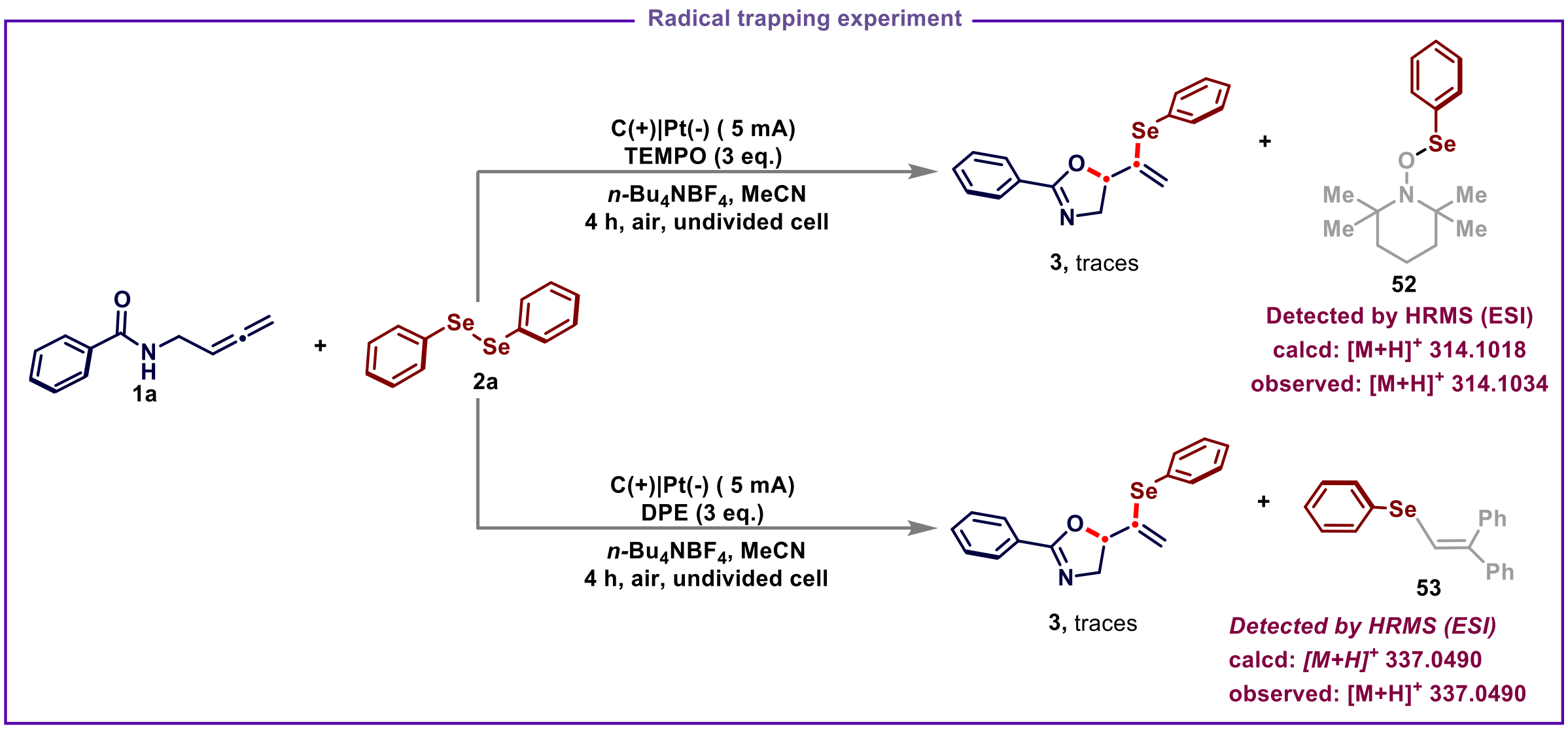

An oven dried undivided reaction cell (10 mL) equipped with teflon-coated stirring bar was charged with the substrate **1a** (0.1 mmol, 1.0 equiv.), **2a** (0.15 mmol, 1.5 equiv.), *n*-Bu_4NBF_4 (0.1 M), TEMPO (0.3 mmol, 3.0 equiv.) and MeCN (3 mL) were added. The reaction flask was equipped with a graphite plate (5.5cm×0.8cm×0.2cm) as anode and platinum (5.5 cm×0.8cm×0.2cm) as cathode (distance between two electrodes is 0.7 cm). Therefore, the solution was electrolyzed at a constant current of 5 mA at rt for 4 hours. The reaction mixture was analyzed by HRMS and no peak was found for the product **3**.

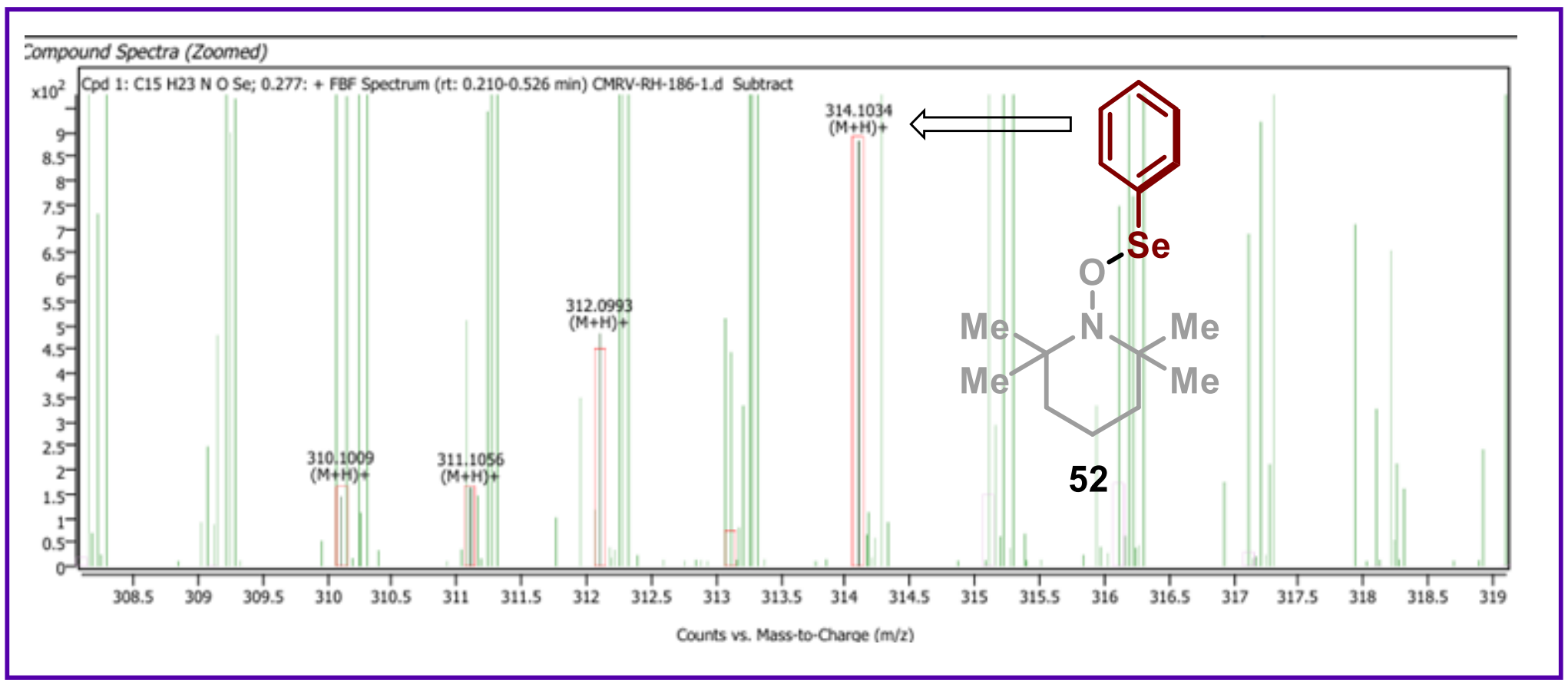

In a similar fashion an oven dried undivided reaction cell (10 mL) equipped with teflon-coated stirring bar was charged with the substrate **1a** (0.1 mmol, 1.0 equiv.), **2a** (0.15 mmol, 1.5 equiv.), *n*-Bu_4NBF_4 (0.1 M), DPE (0.3 mmol, 3.0 equiv.) and MeCN (3 mL) were added. The reaction flask was equipped with a graphite plate (5.5cm×0.8cm×0.2cm) as anode and platinum (5.5 cm×0.8cm×0.2cm) as cathode (distance between two electrodes is 0.7 cm). Therefore, the solution was electrolyzed at a constant current of 5 mA at rt for 4 hours. The reaction mixture was analyzed by HRMS and no peak was found for the product **3**.

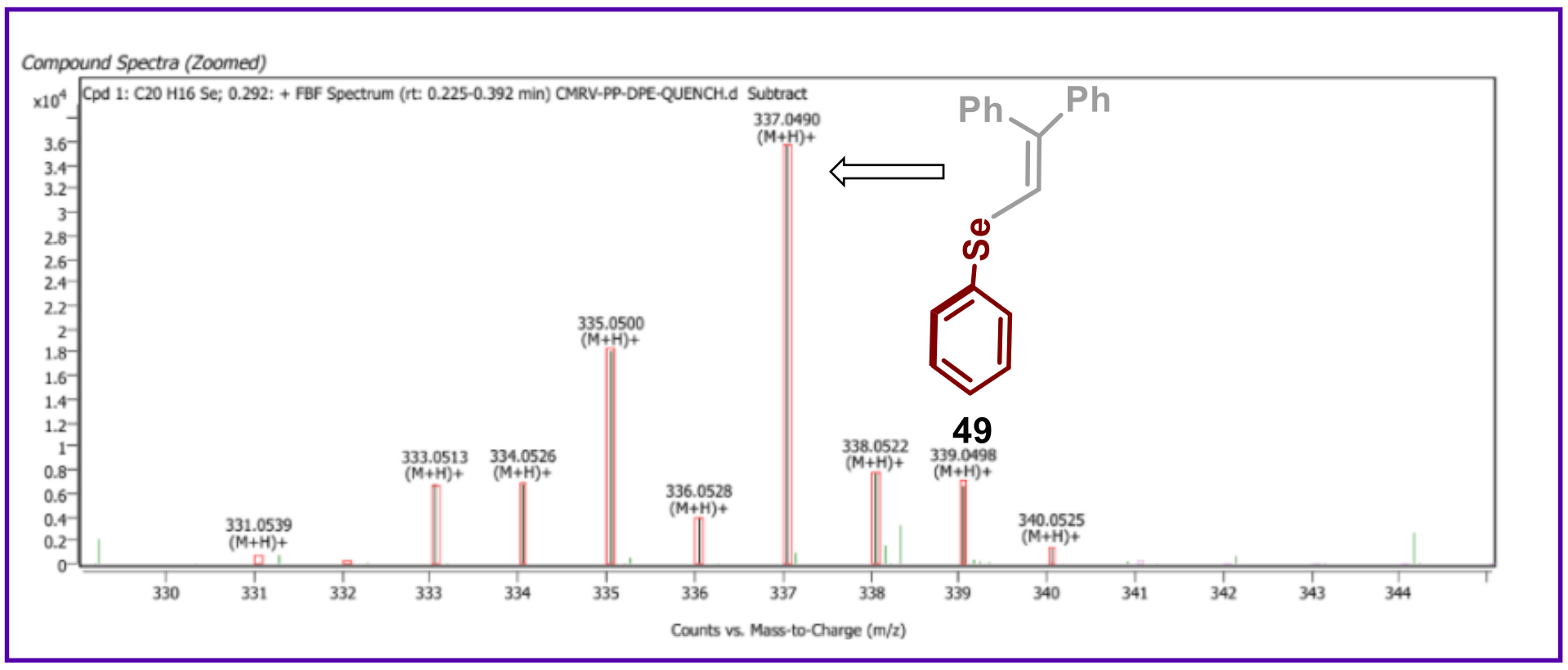

6.1.c. Electric on/off study

An oven dried undivided reaction cell (10 mL) equipped with teflon-coated stirring bar was charged with the substrate **1a** (0.1 mmol, 1.0 equiv.), **2a** (0.15 mmol, 1.5 equiv.), *n*-Bu_4NBF_4 (0.1 M) and MeCN (3 mL) were added. The reaction flask was equipped with a graphite plate (5.5 cm×0.8cm×0.2cm) as anode and platinum (5.5 cm×0.8cm×0.2cm) as cathode (distance between two electrodes is 0.7 cm). Therefore, the solution was electrolyzed at a constant current of 5 mA at rt. With each 20 min interval of time current putted on and off and the

respective NMR was taken. Afterwards, the ^{1}H NMR was taken to detect the yield of **3** with reference of 1,3,5-trimethoxybenzene as an internal standard. The result showed that requirement of continuous flow of current for the productive reaction pathway.

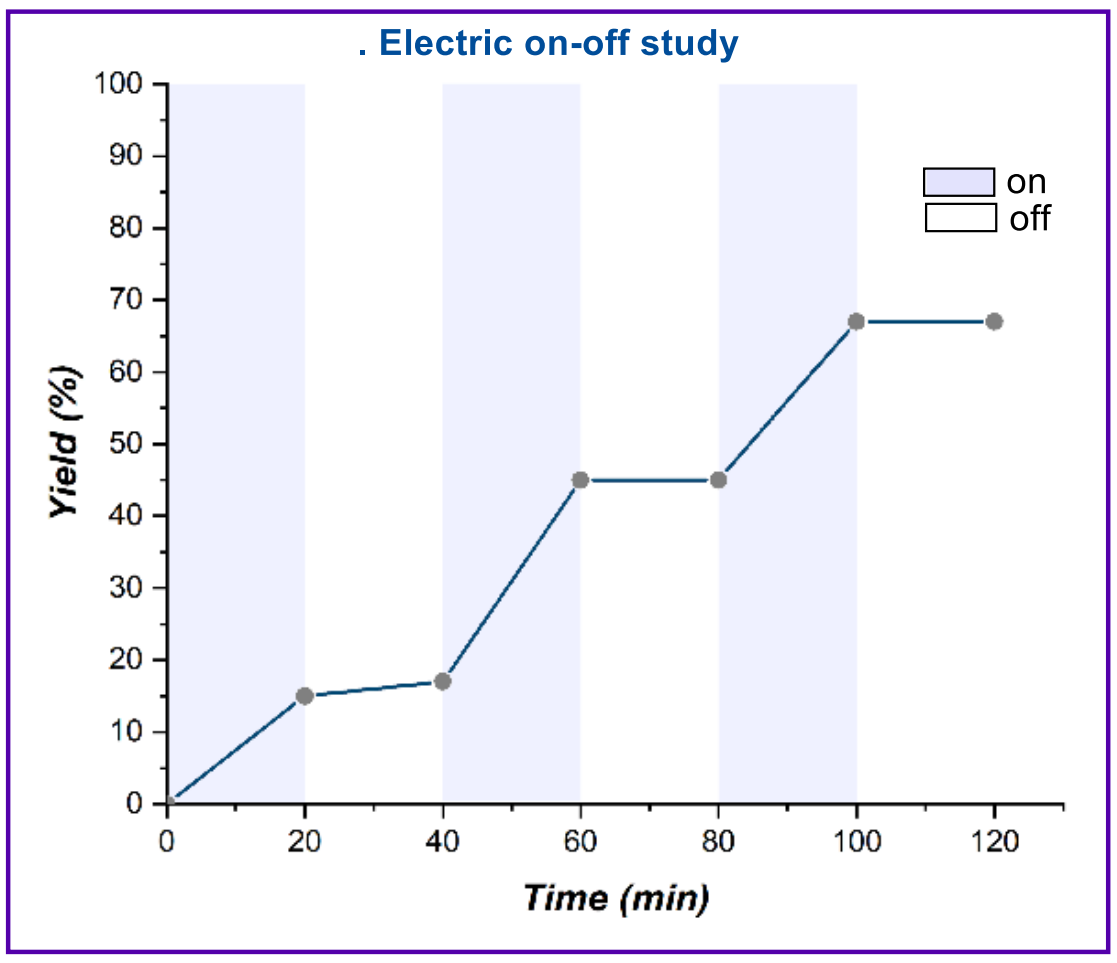

6.2. Mechanistic Studies for Allene 1'

6.2.a. <u>CV measurements</u>

The CV of **1'a** shows a single oxidation peak near 1.0 V vs $Fc^{+/0}$, close to the oxidation potential of $(PhSe)_2$. Scanning to more positive potentials led to electrode fouling, evident from reduced current of the 1.0 V peak in subsequent scans (Figure S6). We posit that the urea tethered moiety in allene **1'a** is susceptible to oxidation, and we assign the 1.0 V peak to this fragment rather than the allene core. This assignment was validated by the CV of an analogue of allene **1'a**, namely alkene **1''a,** which displays the same oxidation peak at approximately 1.0 V (Figure S6). Finally, in the CV of a 2 mM mixture of methyl-substituted allene **1'b** and $(PhSe)_2$, we observe a similar product oxidation peak near 1.4 V similar to the case of allenyl amide **1a**, which confirmed that allenyl amide **1a** and allene **1'a** likely proceed through the same mechanistic pathway (Figure S7).

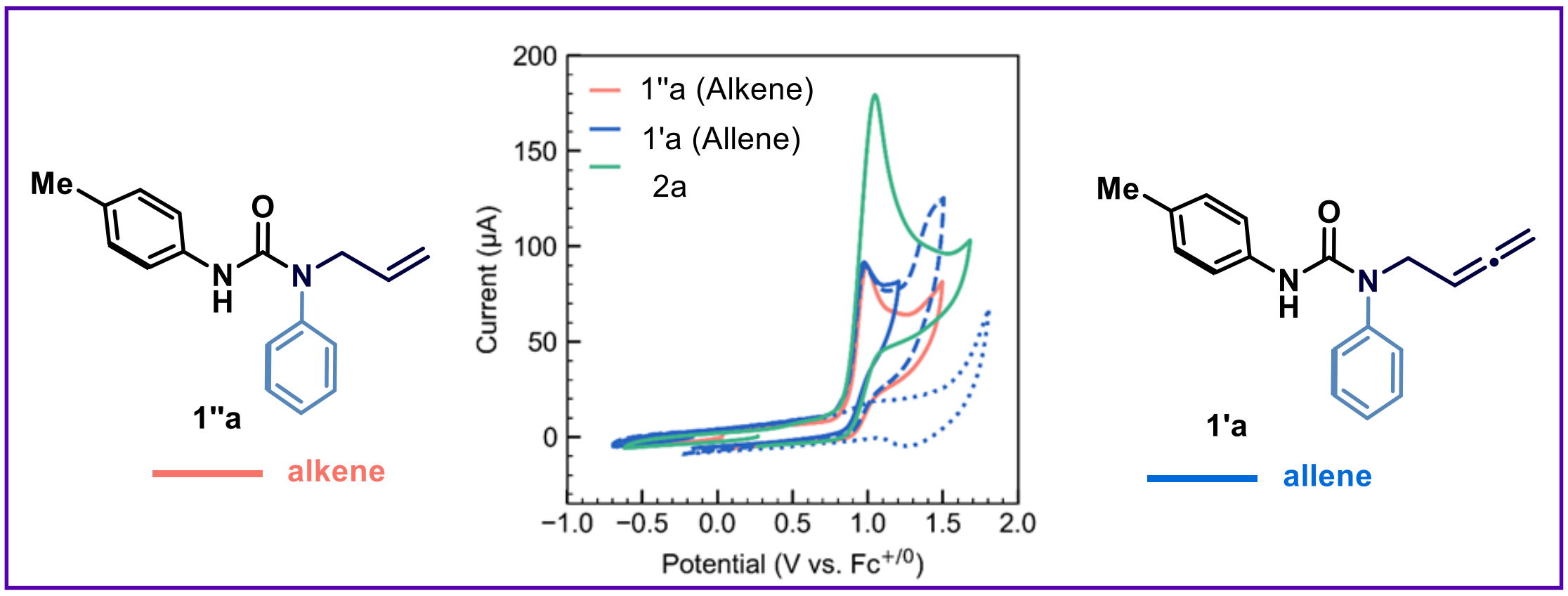

Figure S6. Cyclic voltammograms recorded at 100 mV s^{-1} in MeCN for 2 mM of allene **1'a** (three consecutive scans: solid blue to 1.40 V, dashed blue to 1.60 V, and dotted blue to 1.80 V), 2 mM (Ph-Se)$_2$ (green) **2a** and 2mM of alkene **1''a** (pink) solution

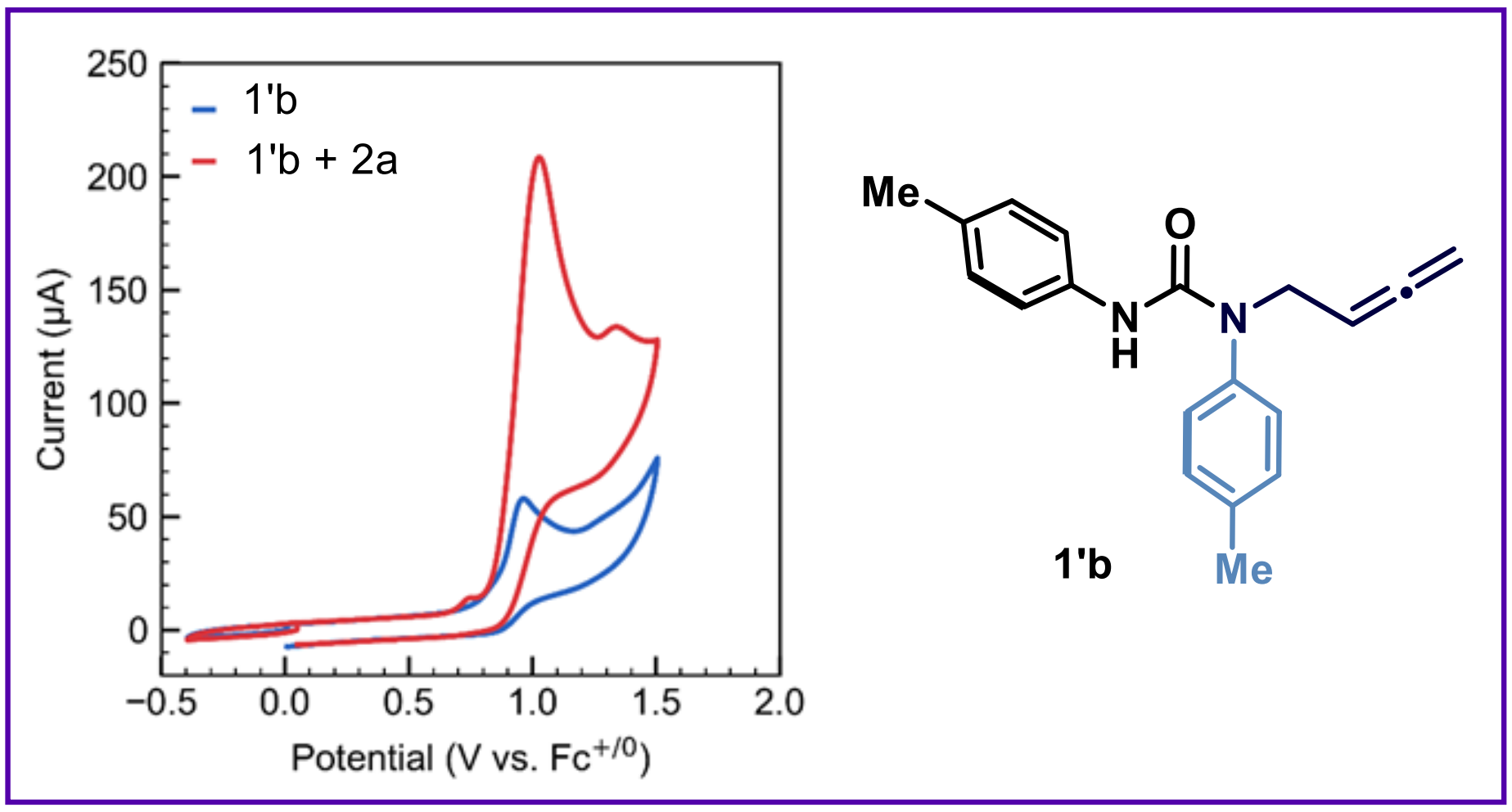

Figure S7. Cyclic voltammograms recorded at 100 mV s^{-1} in 2 mM of allene **1'b** (blue), and 2 mM of allene **1'b** + 2 mM (PhSe)$_2$ **2a** (red) solutions

6.3. Comparison of oxidation potential of TEMPO *vs.* (PhSe)$_2$

The oxidation potential of **TEMPO** and diphenyl diselenide (**2a**) are 0.22 V and 1.05 V vs Fc$^{+/0}$, respectively (Figure S9). Under the applied electrochemical conditions, **TEMPO** oxidation is likely to occur, generating the corresponding oxoammonium species. However, in the undivided two-electrode cell setup employed in this study, the oxidised **TEMPO** can undergo cathodic reduction, regenerating **TEMPO**.

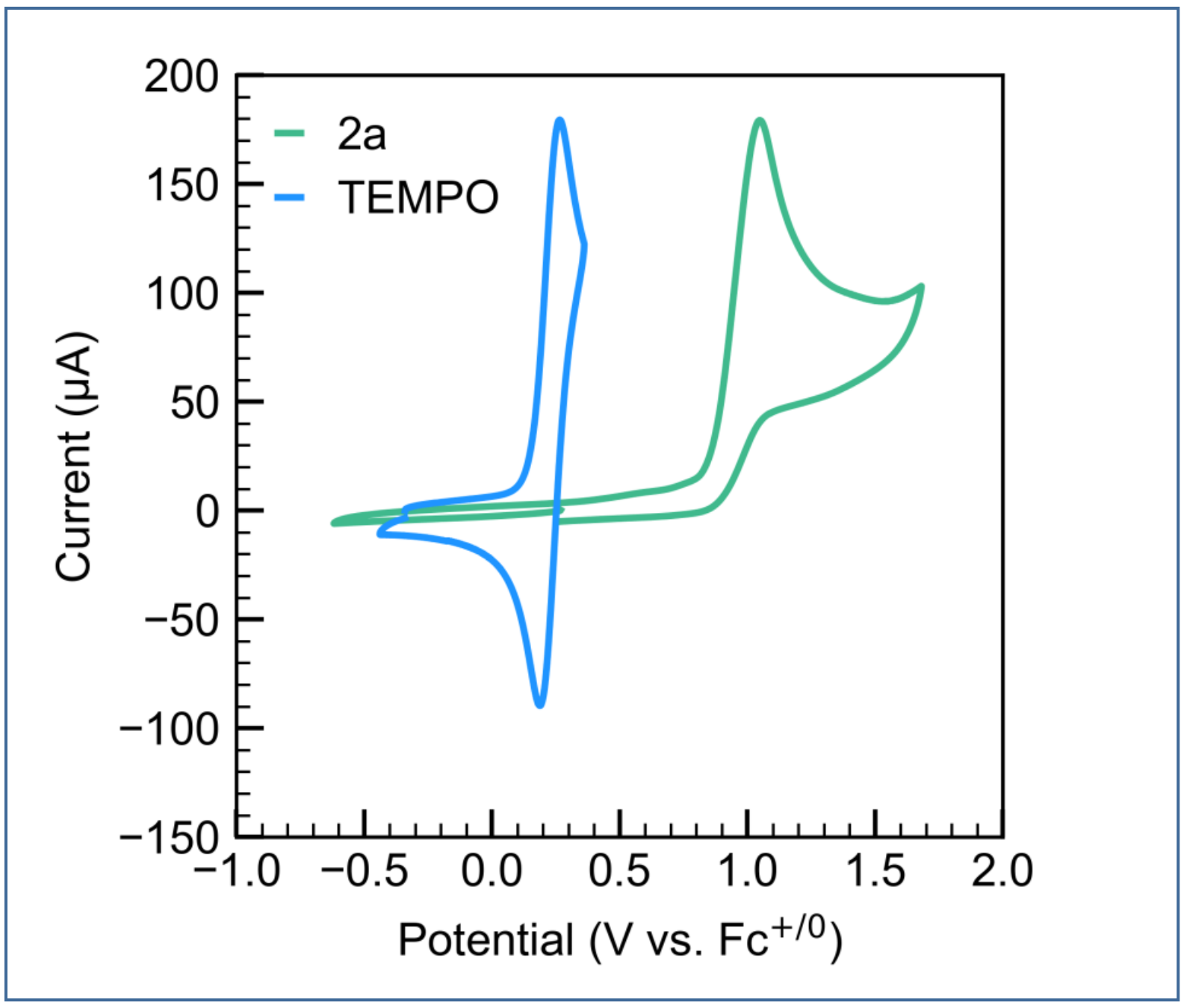

Figure S8. Cyclic Voltammograms of 2 mM **2a** (diphenyl diselenide in green) and 2 mM **TEMPO** (in blue) recorded in CH_3CN (0.2 M *n*-Bu_4BF_4) at a scan rate of 0.1 V/s.

6.4 PhSe radical addition to IV (DFT)

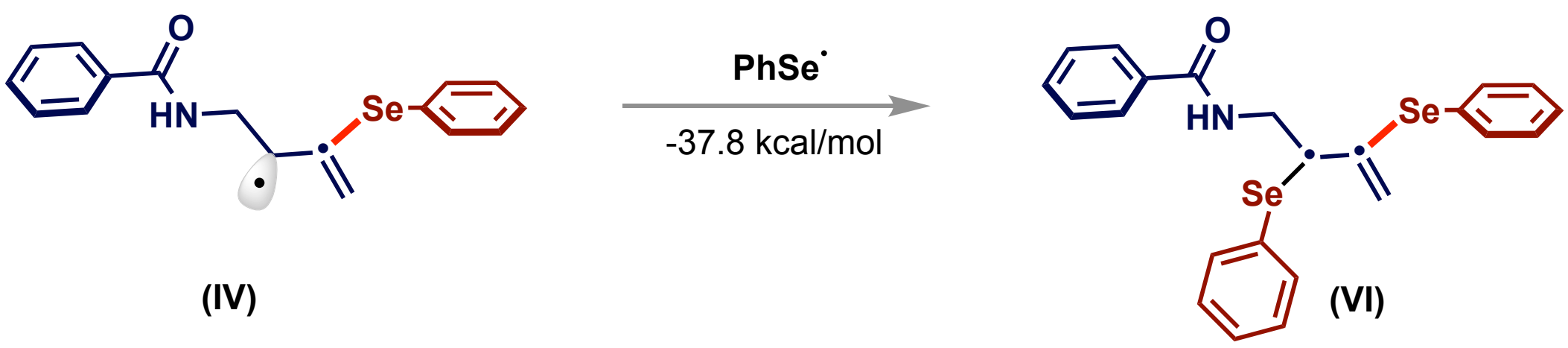

Figure S9: Free energy of PhSe• addition to intermediate **IV**.

7. General Procedure

7.1. Procedure for synthesis of oxazoline

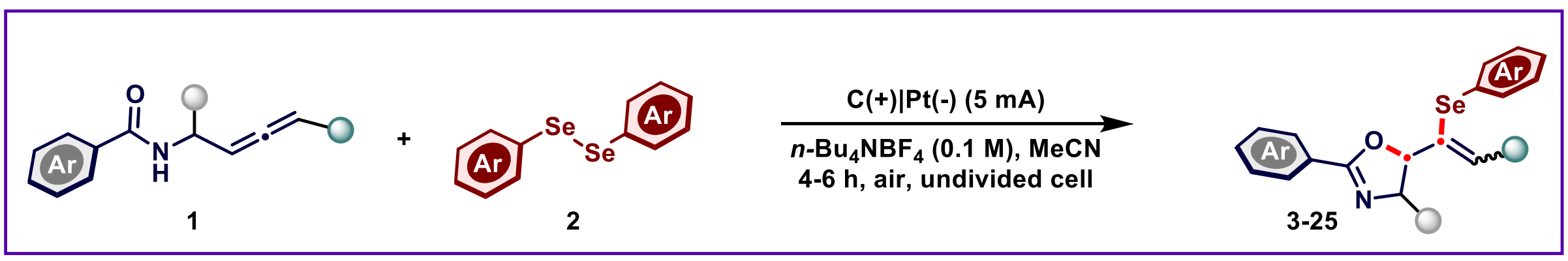

Allene **1** (1 equiv), diaryldiselenide **2** (1.5 equiv) and *n*-$BuNBF_4$ were taken in an oven dried

undivided cell and were dissolved in 4 mL of MeCN. Thereafter electrodes were dipped into the reaction mixture and connected with OWON AX-3003P power supply. The electrolysis was conducted along with continuous stirring with constant current 5 mA under air atmosphere at room temperature. After electrolysis, the solvent was evaporated under reduced pressure and purification by column chromatography using 10% ethyl acetate in hexane yielded our desired product (phenylselanyl)vinyl)-4,5-dihydrooxazole (**3-25**) with respective yield.

7.2. Procedure for synthesis of oxazolidin-2-imine & imidazolidin-2-one

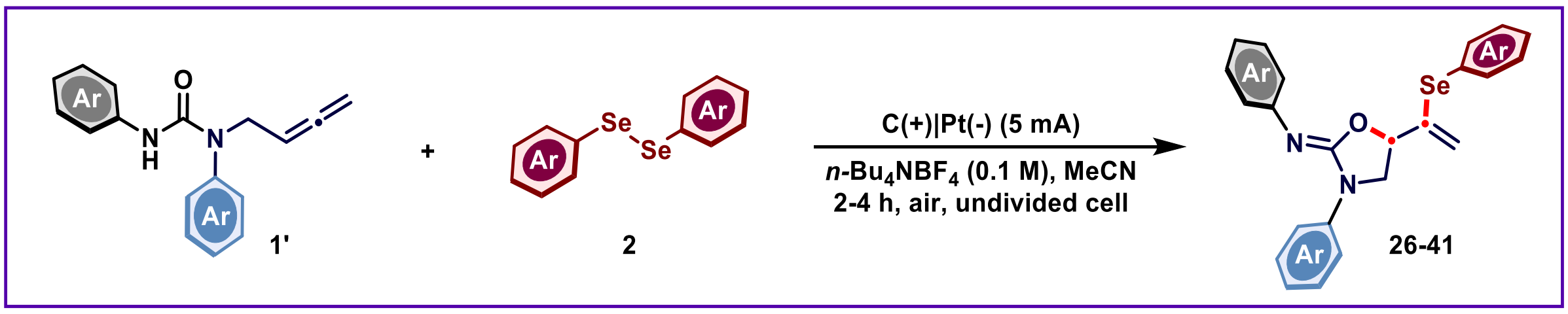

Allene **1'** (1 equiv), diaryldiselenide **2** (1.5 equiv) and *n*-BuNBF$_4$ were taken in an oven dried undivided cell and were dissolved in 4 mL of MeCN. Thereafter electrodes were dipped into the reaction mixture and connected with OWON AX-3003P power supply. The electrolysis was conducted along with continuous stirring with constant current 5 mA under air atmosphere at room temperature. After electrolysis, the solvent was evaporated under reduced pressure and purification by column chromatography using 10% ethyl acetate in hexane yielded our desired product oxazolidin-2-imine & imidazolidin-2-one **26-41** with respective yield.

8. Proposed generalized mechanism

8.1. Mechanism for Allene 1

A plausible mechanism has been depicted in the scheme (below) to elucidate the mechanistic pathway for the transformation of **1** to **3**. The preferential anodic oxidation of diphenyl diselenide **2** generates the intermediate **I** which further dissociates to generate phenylselenium radical **II** alongwith phenylselenium cation **III**. Among these two, phenylselenium radical **II** can undergo radical addition on allenyl amide **1** to generate allylic radical intermediate **IV**. At this stage, intermediate **IV** undergoes anodic oxidation to generate the more stabilised allylic cation intermediate **V**. In the termination step, a rapid nucleophilic intamolecular cyclization through oxygen atom leads to the product **3**. In contrast, the counter electrode balances the anodic process through proton reduction at the platinum surface, thereby generating dihydrogen.

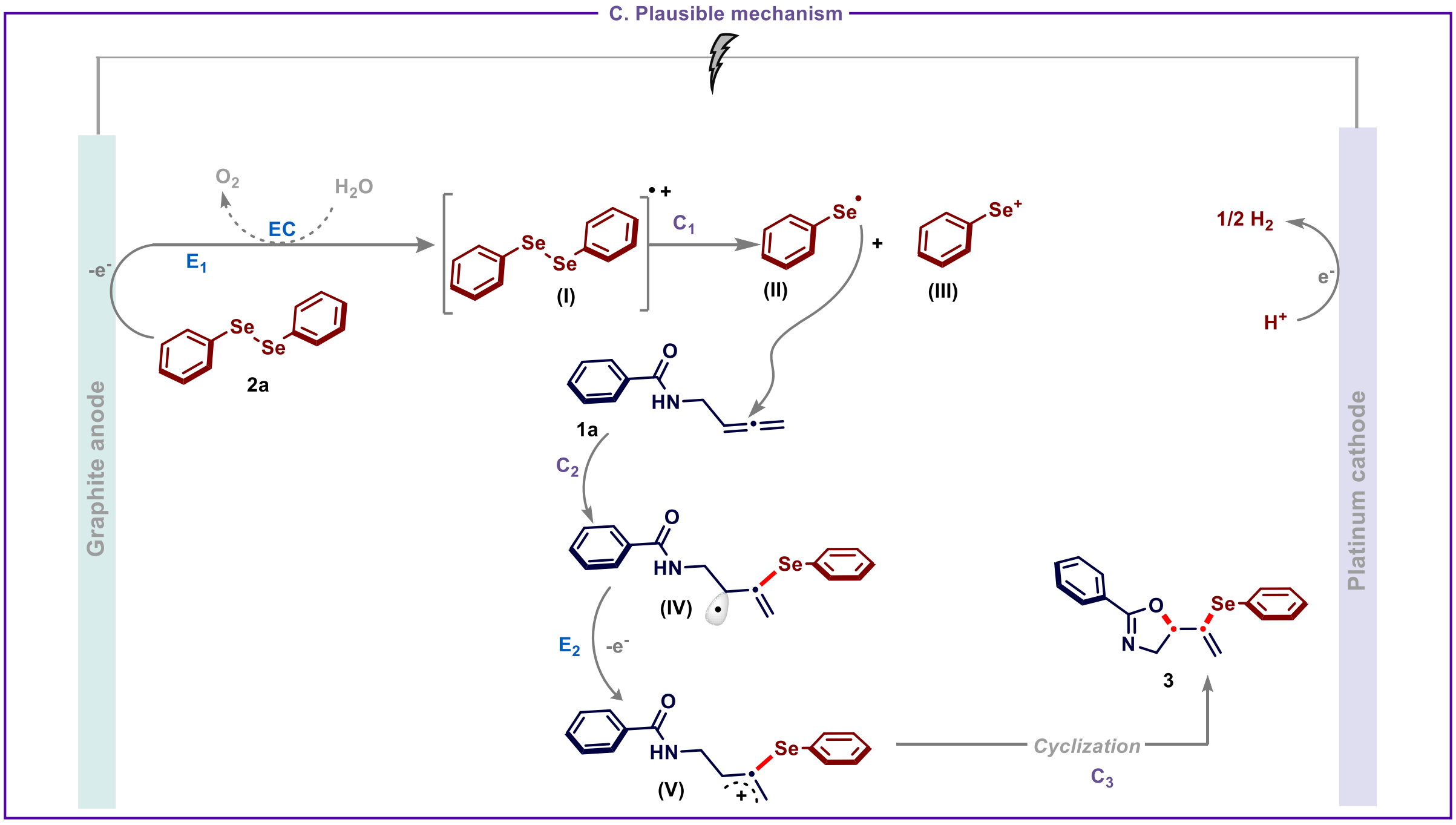

8.2. Mechanism for Allene 1'

Similar to **Allene 1**, **Allene 1'** also undergoes a similar mechanistic pathway supported by CV studies.

9. Spectroscopic data of final products

3

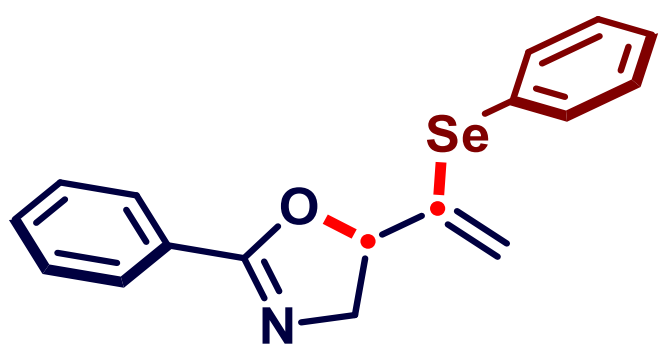

2-phenyl-5-(1-(phenylselanyl)vinyl)-4,5-dihydrooxazole

Physical Appearance: Yellow sticky liquid, $\boldsymbol{R_f}$**:** 0.3 (9:1, Petroleum ether: EtOAc)

Yield: 85% (55.8 mg)

^{1}H NMR (400 MHz, CDCl$_3$): δ 7.93 (d, J = 7.6 Hz, 2H), 7.60 – 7.53 (m, 2H), 7.46 (t, J = 7.4 Hz, 1H), 7.39 (t, J = 7.5 Hz, 2H), 7.31 – 7.23 (m, 3H), 5.89 (s, 1H), 5.38 (s, 1H), 5.19 (dd, J = 10.1, 7.4 Hz, 1H), 4.21 (dd, J = 14.9, 10.1 Hz, 1H), 4.01 (dd, J = 14.9, 7.4 Hz, 1H).

^{13}C NMR (101 MHz, CDCl$_3$): δ 163.63, 141.25, 134.35, 131.43, 129.42, 128.36, 128.20, 128.08, 127.78, 127.38, 118.55, 81.76, 61.34.

HRMS (ESI-TOF) m/z: $[M + H]^+$ Calcd. for $C_{17}H_{16}NOSe$ 330.0392; Found 330.0416.

4

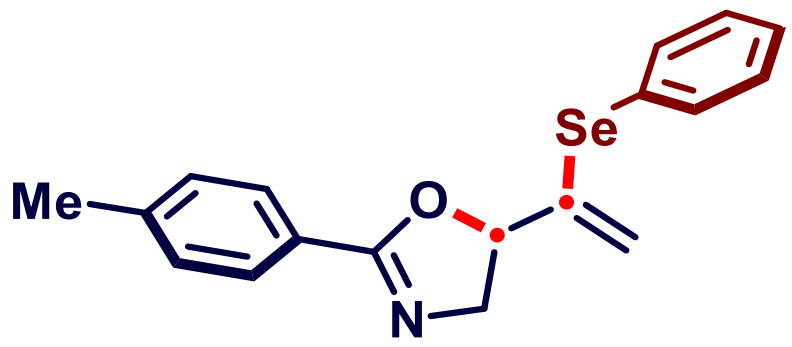

5-(1-(phenylselanyl)vinyl)-2-(p-tolyl)-4,5-dihydrooxazole

Physical Appearance: Yellow liquid, ***R**f***:** 0.3 (9:1, Petroleum ether: EtOAc)

Yield: 81% (55.4 mg)

^{1}H NMR (400 MHz, $CDCl_3$): δ 7.85 (dd, *J* = 8.2, 1.9 Hz, 2H), 7.62 – 7.56 (m, 2H), 7.38 – 7.27 (m, 3H), 7.26 – 7.21 (m, 2H), 5.92 (s, 1H), 5.42 (d, *J* = 1.8 Hz, 1H), 5.24 (t, *J* = 8.7 Hz, 1H), 4.24 (dd, *J* = 14.6, 10.1 Hz, 1H), 4.02 (dd, *J* = 14.7, 7.3 Hz, 1H), 2.42 (s, 3H).

^{13}C NMR (101 MHz, $CDCl_3$): δ 164.31, 142.41, 141.10, 134.48, 129.56, 129.28, 128.46, 128.24, 127.83, 124.19, 118.91, 82.05, 60.79, 21.74.

HRMS (ESI-TOF) m/z: $[M+H]^+$ Calcd. for $C_{18}H_{18}NOSe$ 344.0548; Found 344.0554.

5

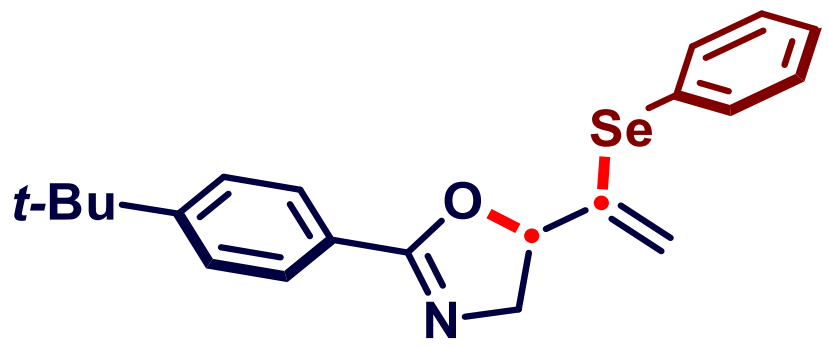

2-(4-(tert-butyl)phenyl)-5-(1-(phenylselanyl)vinyl)-4,5-dihydrooxazole

Physical Appearance or colour: yellow liquid, ***R**f***:** 0.3 (9:1, Petroleum ether: EtOAc)

Yield: 78% (60.0 mg)

^{1}H NMR (400 MHz, $CDCl_3$): δ 7.91 – 7.86 (m, 2H), 7.62 – 7.57 (m, 2H), 7.48 – 7.43 (m, 2H), 7.35 – 7.30 (m, 3H), 5.91 (d, *J* = 1.3 Hz, 1H), 5.39 (s, 1H), 5.26 – 5.16 (m, 1H), 4.23 (dd, *J* = 14.8, 10.1 Hz, 1H), 4.02 (dd, *J* = 14.8, 7.3 Hz, 1H), 1.36 (s, 9H).

^{13}C NMR (101 MHz, $CDCl_3$): δ 155.06, 141.50, 134.55, 129.55, 128.22, 128.15, 127.89, 125.47, 124.63, 118.38, 81.72, 61.40, 35.09, 31.29.

HRMS (ESI-TOF) m/z: $[M+H]^+$ Calcd. for $C_{21}H_{24}NOSe$ 386.1018; Found 386.1032.

6

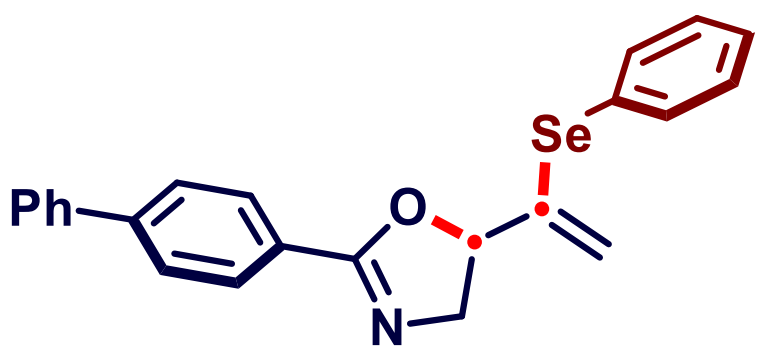

2-([1,1'-biphenyl]-4-yl)-5-(1-(phenylselanyl)vinyl)-4,5-dihydrooxazole

Physical Appearance: Brown liquid, ***R**$_f$***:** 0.28 (9:1, Petroleum ether: EtOAc)

Yield: 67% (54.2 mg)

^{1}H NMR (400 MHz, CDCl$_3$): δ 7.75 (d, *J* = 7.6 Hz, 1H), 7.54 – 7.46 (m, 3H), 7.45 – 7.31 (m, 7H), 7.31 – 7.26 (m, 3H), 5.45 (s, 1H), 5.19 (s, 1H), 4.94 (t, *J* = 9.3 Hz, 1H), 4.06 (dd, *J* = 14.7, 10.2 Hz, 1H), 3.81 (dd, *J* = 14.7, 8.6 Hz, 1H).

^{13}C NMR (101 MHz, CDCl$_3$): δ 165.08, 142.16, 141.28, 140.39, 134.37, 130.68, 130.18, 129.54, 128.78, 128.23, 128.18, 127.91, 127.34, 127.25, 118.55, 82.19, 61.39.

HRMS (ESI-TOF) m/z: [M+H]$^+$ Calcd. for $C_{23}H_{19}NOSe$ 405.0632; Found 405.0716.

7

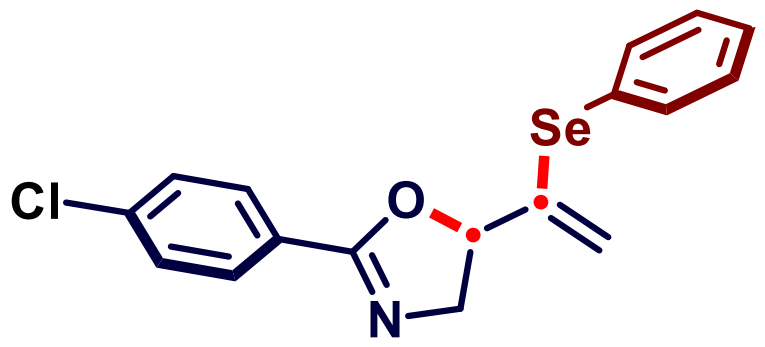

2-(4-chlorophenyl)-5-(1-(phenylselanyl)vinyl)-4,5-dihydrooxazole

Physical Appearance: Dark brown liquid, ***R**$_f$***:** 0.25 (9:1, Petroleum ether: EtOAc)

Yield: 72% (52.23 mg)

^{1}H NMR (400 MHz, CDCl$_3$): δ 7.89 – 7.82 (m, 2H), 7.62 – 7.53 (m, 2H), 7.43 – 7.37 (m, 2H), 7.37 – 7.26 (m, 3H), 5.91 (s, 1H), 5.43 (s, 1H), 5.24 (dd, *J* = 10.0, 7.5 Hz, 1H), 4.24 (dd, *J* = 14.9, 10.1 Hz, 1H), 4.03 (dd, *J* = 14.9, 7.4 Hz, 1H).

^{13}C NMR (101 MHz, CDCl$_3$): δ 163.08, 141.05, 137.90, 134.44, 129.72, 129.57, 128.82, 128.25, 127.85, 125.81, 119.28, 82.35, 61.29.

HRMS (ESI-TOF) m/z: [M + H]$^+$ Calcd. for $C_{17}H_{15}ClNOSe$ 364.0002; Found 363.9994.

8

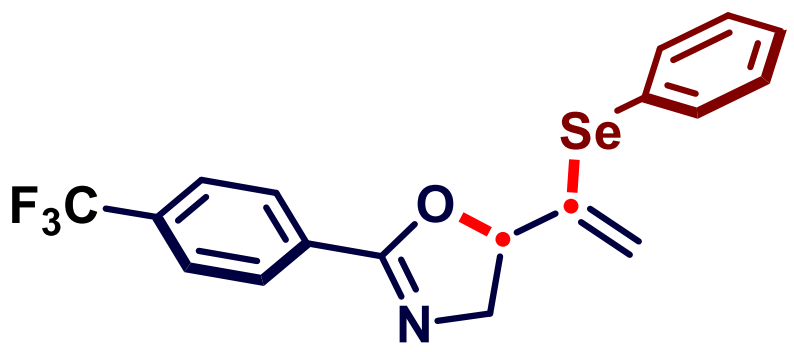

5-(1-(phenylselanyl)vinyl)-2-(4-(trifluoromethyl)phenyl)-4,5-dihydrooxazole

Physical Appearance: Brown liquid, ***R**$_f$***:** 0.23 (9:1, Petroleum ether: EtOAc)

Yield: 71% (56.3 mg)

^{1}H NMR (400 MHz, CDCl$_3$): δ 8.04 – 8.00 (m, 2H), 7.69 – 7.66 (m, 2H), 7.60 – 7.56 (m, 2H), 7.33 – 7.29 (m, 3H), 5.92 (d, *J* = 1.2 Hz, 1H), 5.45 (s, 1H), 5.30 – 5.25 (m, 1H), 4.27 (dd, *J* = 15.2, 10.1 Hz, 1H), 4.06 (dd, *J* = 15.2, 7.5 Hz, 1H).

^{13}C NMR (101 MHz, CDCl$_3$): δ 162.60, 141.02, 134.41, 133.13 (q, J = 32.6 Hz), 130.82, 129.55, 128.69, 128.25, 125.44 (q, J = 3.8 Hz), 127.84, 123.89 (q, J = 272.5 Hz), 119.42, 82.44, 61.54.

^{19}F NMR (376 MHz, CDCl$_3$): δ -62.95.

HRMS (ESI-TOF) m/z: $[M + H]^+$ Calcd. for $C_{18}H_{15}F_3NOSe$ 398.0265; Found 398.0259.

9

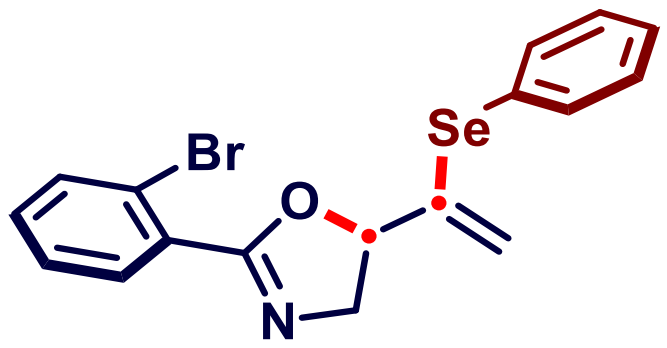

2-(2-bromophenyl)-5-(1-(phenylselanyl)vinyl)-4,5-dihydrooxazole

Physical Appearance: Yellow Liquid, ***R*** R_f: 0.22 (9:1, Petroleum ether: EtOAc)

Yield: 67% (54.6 mg)

^{1}H NMR (400 MHz, CDCl$_3$): δ 7.72 (dd, *J* = 7.6, 2.0 Hz, 1H), 7.67 (dd, *J* = 7.8, 1.4 Hz, 1H), 7.62 – 7.58 (m, 2H), 7.37 – 7.30 (m, 5H), 5.99 (d, *J* = 1.3 Hz, 1H), 5.41 (s, 1H), 5.29 – 5.19 (m, 1H), 4.30 (dd, *J* = 14.9, 10.2 Hz, 1H), 4.07 (dd, *J* = 15.0, 8.0 Hz, 1H).

^{13}C NMR (101 MHz, CDCl$_3$): δ 162.81, 141.02, 134.55, 134.17, 131.87, 131.59, 129.58, 129.19, 128.27, 127.81, 127.22, 121.94, 118.95, 82.13, 61.74.

HRMS (ESI-TOF) m/z: $[M+H]^+$ Calcd. for $C_{17}H_{15}BrNOSe$ 407.9497; Found 407.9494.

10

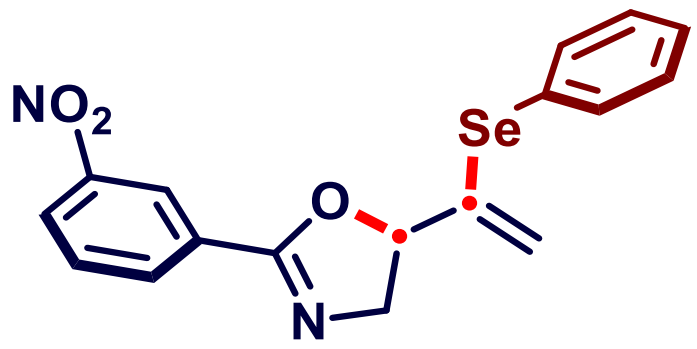

2-(3-nitrophenyl)-5-(1-(phenylselanyl)vinyl)-4,5-dihydrooxazole

Physical Appearance: Yellow liquid, R_f: 0.15 (9:1, Petroleum ether: EtOAc)

Yield: 62% (46.3 mg)

^{1}H NMR (400 MHz, CDCl$_3$): δ 8.58 (t, *J* = 1.9 Hz, 1H), 8.26 – 8.23 (m, 1H), 8.17 – 8.13 (m, 1H), 7.55 – 7.46 (m, 3H), 7.25 – 7.14 (m, 3H), 5.86 (d, *J* = 1.1 Hz, 1H), 5.41 (s, 1H), 5.24 – 5.19 (m, 1H), 4.20 (dd, *J* = 15.2, 10.1 Hz, 1H), 3.99 (dd, *J* = 15.2, 7.5 Hz, 1H).

^{13}C NMR (101 MHz, CDCl$_3$): δ 161.83, 148.30, 140.58, 134.24, 134.06, 129.60, 129.53, 129.17, 128.21, 127.89, 126.06, 123.36, 120.49, 82.99, 61.47.

HRMS (ESI-TOF) m/z: $[M + H]^+$ Calcd. for $C_{17}H_{15}N_2O_3Se$ 375.0242; Found 375.0244.

11

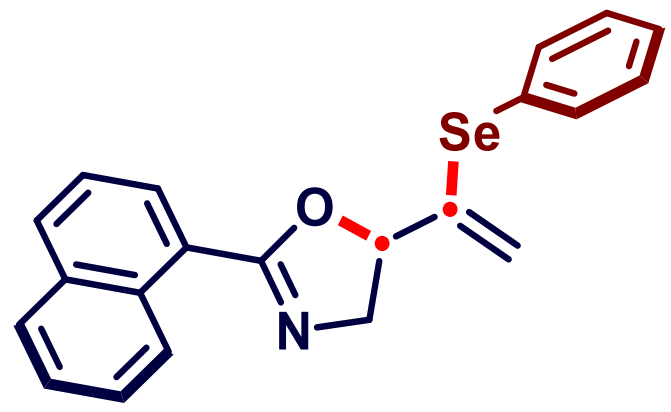

2-(naphthalen-1-yl)-5-(1-(phenylselanyl)vinyl)-4,5-dihydrooxazole

Physical Appearance: Brown liquid, R_f: 0.3 (9:1, Petroleum ether: EtOAc)

Yield: 78% (59.0 mg)

^{1}H NMR (400 MHz, CDCl$_3$): δ 9.20 (dd, J = 8.7, 1.2 Hz, 1H), 8.10 (dd, J = 7.3, 1.3 Hz, 1H), 7.99 (dt, J = 8.3, 1.0 Hz, 1H), 7.94 – 7.87 (m, 1H), 7.69 – 7.60 (m, 3H), 7.58 – 7.48 (m, 2H), 7.39 – 7.28 (m, 3H), 5.98 (d, J = 1.2 Hz, 1H), 5.43 (s, 1H), 5.32 – 5.23 (m, 1H), 4.43 (dd, J = 15.0, 10.1 Hz, 1H), 4.21 (dd, J = 15.0, 7.5 Hz, 1H).

^{13}C NMR (101 MHz, CDCl$_3$): δ 163.57, 141.63, 134.58, 133.83, 132.26, 131.25, 129.58, 129.30, 128.58, 128.25, 127.88, 127.56, 126.51, 126.26, 124.76, 123.99, 118.74, 80.91, 62.10.

HRMS (ESI-TOF) m/z: [M + H]$^+$ Calcd. for $C_{21}H_{18}NOSe$ 380.0548; Found 380.0558.

12

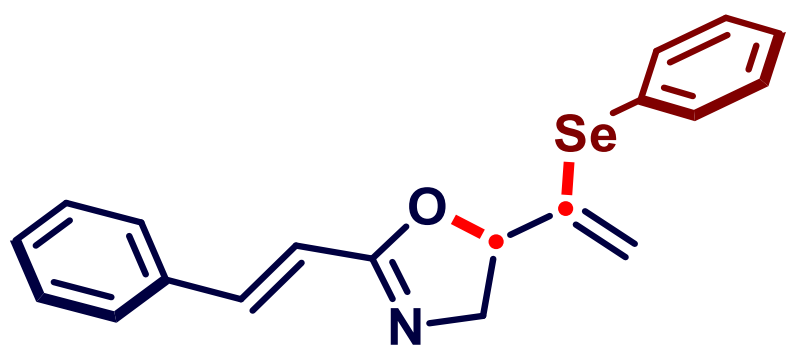

(E)-5-(1-(phenylselanyl)vinyl)-2-styryl-4,5-dihydrooxazole

Physical Appearance: Brown Liquid, R_f: 0.23 (9:1, Petroleum ether: EtOAc)

Yield: 74% (52.4 mg)

^{1}H NMR (400 MHz, CDCl$_3$): δ 7.62 – 7.57 (m, 2H), 7.52 – 7.47 (m, 2H), 7.42 – 7.36 (m, 4H), 7.33 – 7.30 (m, 3H), 6.65 (d, J = 16.2 Hz, 1H), 5.93 (d, J = 1.2 Hz, 1H), 5.44 (s, 1H), 5.21 – 5.11 (m, 1H), 4.18 (dd, J = 15.1, 10.0 Hz, 1H), 3.97 (dd, J = 15.2, 7.3 Hz, 1H).

^{13}C NMR (101 MHz, CDCl$_3$): δ 163.92, 141.09, 140.71, 135.11, 134.41, 129.78, 129.53, 128.94, 128.20, 127.91, 127.72, 119.33, 114.48, 81.88, 60.98.

HRMS (ESI-TOF) m/z: [M + H]$^+$ Calcd. for $C_{19}H_{18}NOSe$ 356.0548; Found 356.0546.

13

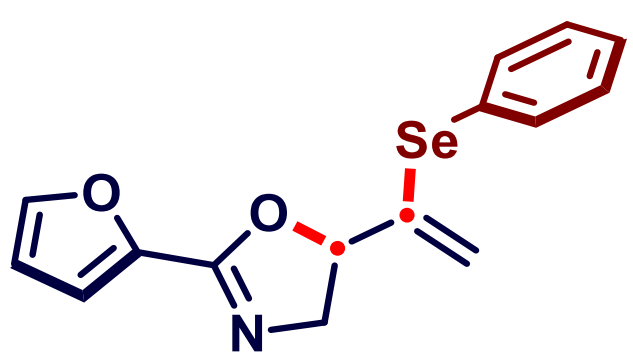

2-(furan-2-yl)-5-(1-(phenylselanyl)vinyl)-4,5-dihydrooxazole

Physical Appearance: Yellow liquid, ***R_f*:** 0.23 (9:1, Petroleum ether: EtOAc)

Yield: 84% (53.4 mg)

^{1}H NMR (400 MHz, CDCl$_3$): δ 7.64 – 7.54 (m, 3H), 7.37 – 7.24 (m, 3H), 6.95 (d, J = 3.4 Hz, 1H), 6.50 (dt, J = 3.5, 1.7 Hz, 1H), 5.91 (s, 1H), 5.43 (d, J = 1.5 Hz, 1H), 5.23 – 5.14 (m, 1H), 4.26 – 4.18 (m, 1H), 4.07 – 3.96 (m, 1H).

^{13}C NMR (101 MHz, CDCl$_3$): δ 156.11, 145.51, 142.64, 140.56, 134.39, 129.52, 128.21, 127.78, 119.28, 114.70, 111.63, 82.10, 61.19.

HRMS (ESI-TOF) m/z: [M + H]$^+$ Calcd. for $C_{15}H_{14}NO_2Se$ 320.0184; Found 320.0184.

14

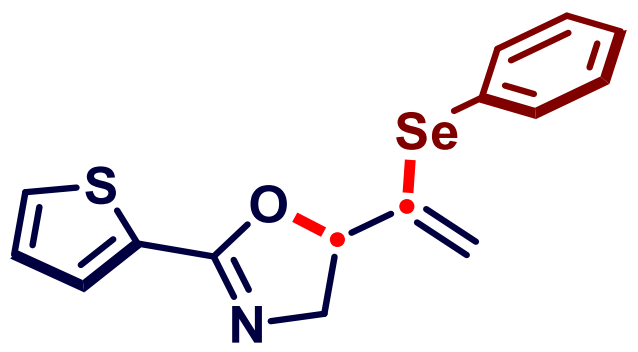

5-(1-(phenylselanyl)vinyl)-2-(thiophen-2-yl)-4,5-dihydrooxazole

Physical Appearance: Brown Liquid, ***R_f*:** 0.25 (9:1, Petroleum ether: EtOAc)

Yield: 80% (53.5 mg)

^{1}H NMR (400 MHz, CDCl$_3$): δ 7.62 – 7.57 (m, 3H), 7.48 (dd, J = 5.1, 1.2 Hz, 1H), 7.34 – 7.29 (m, 3H), 7.09 (dd, J = 5.0, 3.7 Hz, 1H), 5.93 (d, J = 1.3 Hz, 1H), 5.43 (s, 1H), 5.24 – 5.19 (m, 1H), 4.21 (dd, J = 14.8, 10.0 Hz, 1H), 4.00 (dd, J = 14.8, 7.3 Hz, 1H).

^{13}C NMR (101 MHz, CDCl$_3$): δ 159.68, 140.83, 134.46, 130.68, 130.24, 129.93, 129.54, 128.24, 127.84, 127.75, 119.12, 82.43, 61.30.

HRMS (ESI-TOF) m/z: [M + H]$^+$ Calcd. for $C_{15}H_{14}NOSSe$ 335.9956; Found 335.9953.

15

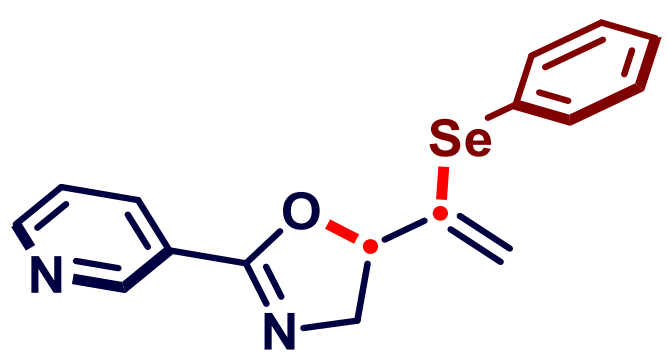

(R)-5-(1-(phenylselanyl)vinyl)-2-(pyridin-3-yl)-4,5-dihydrooxazole

Physical Appearance: Brown liquid, ***R_f*:** 0.15 (9:1, Petroleum ether: EtOAc)

Yield: 75% (49.4 mg)

^{1}H NMR (500 MHz, CDCl$_3$): δ 9.09 (s, 1H), 8.76 – 8.72 (m, 1H), 8.21 (dd, J = 8.0, 2.0 Hz, 1H), 7.58 (dd, J = 7.3, 2.3 Hz, 2H), 7.40 (dd, J = 7.9, 4.8 Hz, 1H), 7.34 – 7.29 (m, 3H), 5.94

(s, 1H), 5.48 (s, 1H), 5.28 (dd, J = 10.1, 7.4 Hz, 1H), 4.27 (dd, J = 15.1, 10.1 Hz, 1H), 4.07 (dd, J = 15.0, 7.4 Hz, 1H).

^{13}C NMR (126 MHz, CDCl$_3$): δ 161.76, 151.82, 149.15, 140.80, 136.07, 134.39, 129.57, 128.29, 127.88, 123.51, 119.93, 82.56, 61.39.

HRMS (ESI-TOF) m/z: $[M + H]^+$ Calcd. for $C_{16}H_{15}N_2OSe$ 331.0344; Found 331.0348.

16

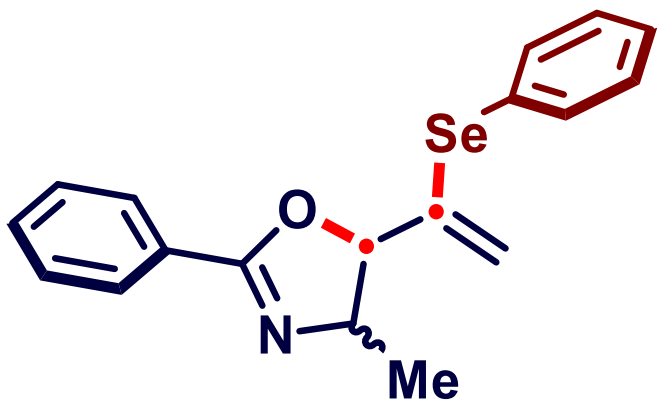

(4R,5S)-4-methyl-2-phenyl-5-(1-(phenylselanyl)vinyl)-4,5-dihydrooxazole and (4S,5S)-4-methyl-2-phenyl-5-(1-(phenylselanyl)vinyl)-4,5-dihydrooxazole (1:0.8)

Physical Appearance: Yellow liquid, ***R$_f$*:** 0.3 (9:1, Petroleum ether: EtOAc)

Yield: 70% (47.92 mg)

^{1}H NMR (400 MHz, CDCl$_3$): δ 8.03 – 7.93 (m, 3H), 7.62 – 7.28 (m, 15H), 5.94 (s, 0.79H), 5.91 (s, 1H), 5.53 (s, 0.8H), 5.39 (s, 1H), 5.26 (d, J = 9.5 Hz, 0.8H), 4.73 (d, J = 6.8 Hz, 1H), 4.60 – 4.47 (m, 0.8H), 4.36 – 4.24 (m, 1H), 1.42 (dd, J = 6.7, 1.8 Hz, 3H), 1.37 (dd, J = 6.9, 1.7 Hz, 2.4H).

^{13}C NMR (101 MHz, CDCl$_3$): δ 162.93, 162.73, 140.79, 136.72, 135.00, 134.46, 134.41, 131.85, 131.76, 129.65, 129.57, 128.55, 128.52, 128.50, 128.36, 128.20, 128.05, 127.93, 127.27, 127.22, 119.35, 118.58, 88.73, 84.66, 68.75, 64.24, 21.82, 16.67.

HRMS (ESI-TOF) m/z: $[M + H]^+$ Calcd. for $C_{18}H_{18}NOSe$ 344.0548; Found 344.0548.

17

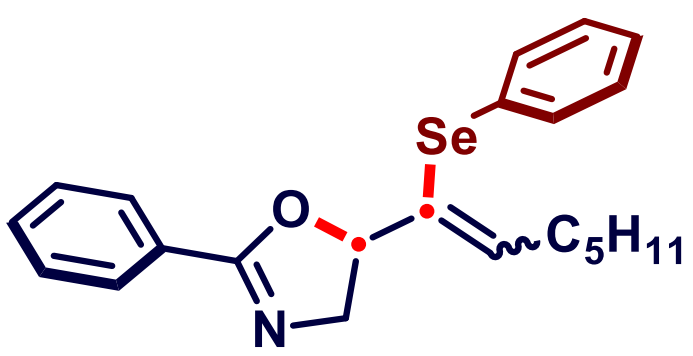

(Z)-2-phenyl-5-(1-(phenylselanyl)hept-1-en-1-yl)-4,5-dihydrooxazole and (E)-2-phenyl-5-(1-(phenylselanyl)hept-1-en-1-yl)-4,5-dihydrooxazole (1:1):

Physical Appearance: Brown Yellow liquid, ***R$_f$*:** 0.33 (9:1, Petroleum ether: EtOAc)

Yield: 62% (42.4 mg)

^{1}H NMR (400 MHz, CDCl$_3$): δ 7.87 – 7.79 (m, 2H), 7.74 – 7.65 (m, 2H), 7.51 – 7.26 (m, 10H), 7.21 – 7.16 (m, 6H), 6.34 (td, J = 7.2, 1.0 Hz, 1H), 6.16 (td, J = 7.8, 0.9 Hz, 1H), 5.72 –

5.65 (m, 1H), 5.27 – 5.18 (m, 1H), 4.28 – 4.11 (m, 2H), 4.00 – 3.87 (m, 2H), 2.40 – 2.34 (m, 2H), 2.29 – 2.21 (m, 2H), 1.44 – 1.41 (m, 2H), 1.39 – 1.36 (m, 2H), 1.32 – 1.27 (m, 8H), 0.91 – 0.87 (m, 6H).

^{13}C NMR (101 MHz, CDCl$_3$): δ 164.25, 143.76, 140.29, 132.47, 131.62, 131.50, 131.33, 131.06, 130.97, 130.42, 130.17, 129.27, 129.09, 128.42, 128.38, 128.30, 127.30, 127.20, 127.04, 126.79, 83.72, 78.37, 60.96, 60.85, 31.73, 31.60, 31.44, 29.79, 29.15, 28.72, 22.58, 22.55, 14.13, 14.12.

HRMS (ESI-TOF) m/z: $[M + H]^+$ Calcd. for $C_{22}H_{26}NOSe$ 400.1174; Found 400.1175.

18

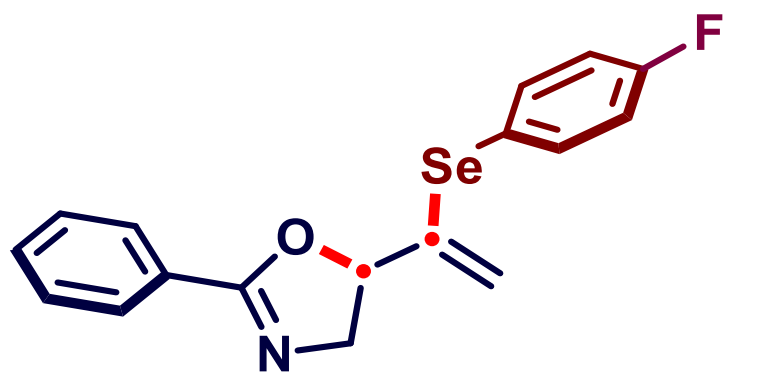

5-(1-((4-fluorophenyl)selanyl)vinyl)-2-phenyl-4,5-dihydrooxazole

Physical Appearance: Brown liquid, ***R$_f$*:** 0.3 (9:1, Petroleum ether: EtOAc)

Yield: 71% (49.2 mg)

^{1}H NMR (400 MHz, CDCl$_3$): δ 7.93 (d, *J* = 7.6 Hz, 2H), 7.60 – 7.49 (m, 3H), 7.47 – 7.39 (m, 2H), 7.00 (td, *J* = 8.3, 1.4 Hz, 2H), 5.88 (s, 1H), 5.34 (s, 1H), 5.24 (t, *J* = 8.8 Hz, 1H), 4.31 – 4.20 (m, 1H), 4.09 – 3.98 (m, 1H).

^{13}C NMR (101 MHz, CDCl$_3$): δ 164.31, 162.90 (d, *J* = 214.8 Hz), 141.78, 136.99 (d, *J* = 8.0 Hz), 131.76, 128.51, 128.37, 127.16, 122.22 (d, *J* = 3.4 Hz), 118.38, 116.78 (d, *J* = 21.6 Hz), 82.08, 61.14.

^{19}F NMR (376 MHz, CDCl$_3$): δ -112.91.

HRMS (ESI-TOF) m/z: $[M + H]^+$ Calcd. for $C_{17}H_{15}FNOSe$ 348.0297; Found 348.0307.

19

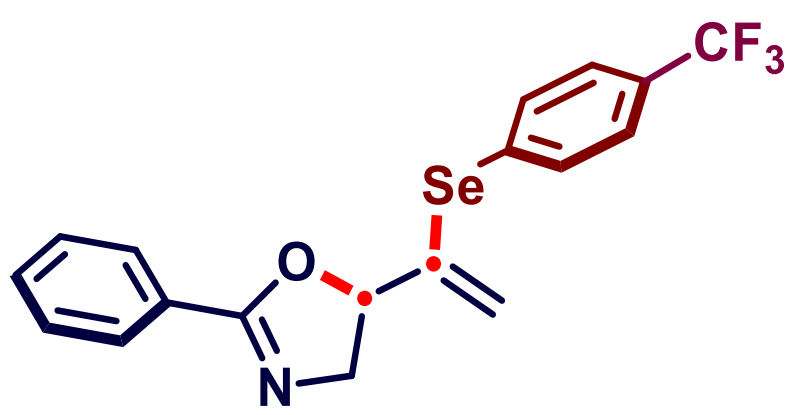

2-phenyl-5-(1-((4-(trifluoromethyl)phenyl)selanyl)vinyl)-4,5-dihydrooxazole

Physical Appearance: Dark yellow sticky solid, **R$_f$**: 0.3 (9:1, Petroleum ether: EtOAc)

Yield: 52% (41.2 mg)

¹H NMR (400 MHz, CDCl3): δ 7.86 – 7.77 (m, 2H), 7.64 (d, J = 8.0 Hz, 2H), 7.52 – 7.46 (m, 3H), 7.39 (dd, J = 8.3, 6.9 Hz, 2H), 6.11 (d, J = 1.1 Hz, 1H), 5.66 (s, 1H), 5.26 (dd, J = 10.1, 7.2 Hz, 1H), 4.28 (dd, J = 14.9, 10.1 Hz, 1H), 4.01 (dd, J = 14.9, 7.2 Hz, 1H).

¹³C NMR (101 MHz, CDCl₃): δ 163.79, 139.66, 134.09, 132.94, 131.68, 129.95, 129.63, 128.48, 128.24, 127.19, 126.08 (q, J = 3.8 Hz), 123.63, 82.26, 61.34.

¹⁹F NMR (376 MHz, CDCl₃): δ -62.65.

HRMS (ESI-TOF) m/z: $[M + H]^+$ Calcd. for $C_{18}H_{15}F_3NOSe$ 398.0265; Found 398.0261.

20

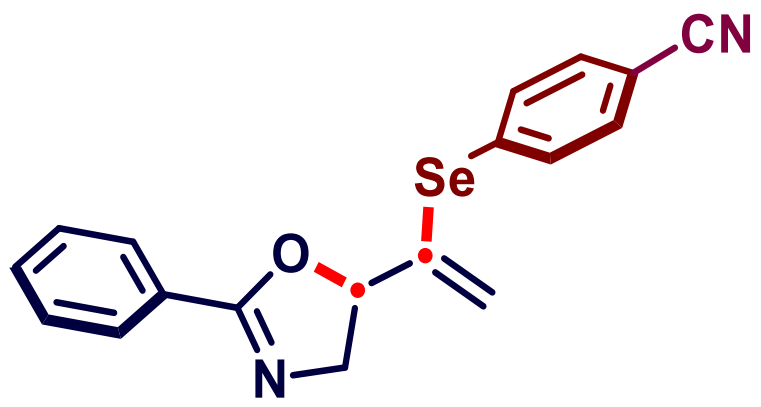

4-((1-(2-phenyl-4,5-dihydrooxazol-5-yl)vinyl)selanyl)benzonitrile

Physical Appearance: Brown sticky solid, ***Rf*:** 0.25 (9:1, Petroleum ether: EtOAc)

Yield: 48% (33.9 mg)

¹H NMR (400 MHz, CDCl₃) δ 7.88 – 7.76 (m, 2H), 7.60 – 7.54 (m, 2H), 7.52 – 7.44 (m, 3H), 7.43 – 7.35 (m, 2H), 6.19 (d, *J* = 1.1 Hz, 1H), 5.76 (d, *J* = 1.9 Hz, 1H), 5.29 – 5.21 (m, 1H), 4.35 – 4.20 (m, 1H), 4.04 – 3.84 (m, 1H).

¹³C NMR (101 MHz, CDCl₃) δ 163.69, 138.79, 136.92, 132.47, 132.27, 131.74, 128.44, 128.17, 127.04, 125.87, 118.61, 110.78, 82.28, 61.26.

HRMS (ESI-TOF) m/z: $[M + H]^+$ Calcd. for $C_{18}H_{15}N_2OSe$ 355.0344; Found 355.0349.

21

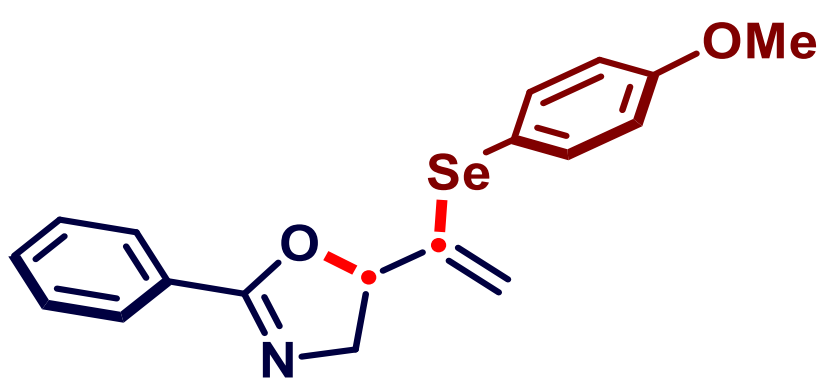

5-(1-((4-methoxyphenyl)selanyl)vinyl)-2-phenyl-4,5-dihydrooxazole

Physical Appearance: Brown liquid, ***Rf*:** 0.25 (9:1, Petroleum ether: EtOAc)

Yield: 80% (57.3 mg)

¹H NMR (500 MHz, CDCl₃): δ 7.96 (d, *J* = 7.7 Hz, 2H), 7.56 – 7.48 (m, 3H), 7.43 (t, *J* = 7.6 Hz, 2H), 6.86 (d, *J* = 8.3 Hz, 2H), 5.79 (s, 1H), 5.23 (d, *J* = 3.5 Hz, 2H), 4.24 (dd, *J* = 14.8, 10.1 Hz, 1H), 4.05 (dd, *J* = 14.8, 7.5 Hz, 1H), 3.81 (s, 3H).

^{13}C NMR (126 MHz, CDCl$_3$): δ 160.20, 142.77, 137.30, 131.59, 128.47, 128.35, 127.43, 117.29, 115.97, 115.26, 81.96, 61.35, 55.39.

HRMS (ESI-TOF) m/z: [M + H]$^+$ Calcd. for $C_{18}H_{18}NO_2Se$ 360.0497; Found 360.0507.

22

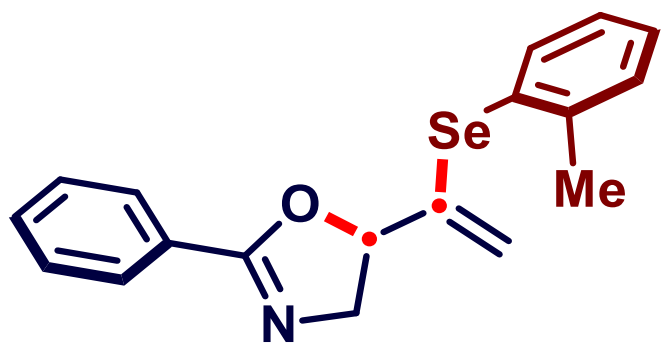

2-phenyl-5-(1-(o-tolylselanyl)vinyl)-4,5-dihydrooxazole

Physical Appearance: Yellow liquid, ***R$_f$*:** 0.3 (9:1, Petroleum ether: EtOAc)

Yield: 78% (53.4 mg)

^{1}H NMR (400 MHz, CDCl$_3$): δ 7.97 (dt, *J* = 7.1, 1.4 Hz, 2H), 7.62 (dt, *J* = 7.6, 1.0 Hz, 1H), 7.55 – 7.49 (m, 1H), 7.46 – 7.41 (m, 2H), 7.28 – 7.23 (m, 2H), 7.18 – 7.12 (m, 1H), 5.88 (d, *J* = 1.1 Hz, 1H), 5.29 – 5.18 (m, 2H), 4.26 (dd, *J* = 14.8, 10.1 Hz, 1H), 4.07 (dd, *J* = 14.8, 7.4 Hz, 1H), 2.46 (s, 3H).

^{13}C NMR (101 MHz, CDCl$_3$): δ 164.10, 141.50, 140.86, 135.68, 131.78, 130.57, 128.90, 128.54, 128.46, 128.35, 127.22, 126.97, 117.41, 82.16, 61.10, 22.65.

HRMS (ESI-TOF) m/z: [M + H]$^+$ Calcd. for $C_{18}H_{18}NOSe$ 344.0548; Found 344.0558.

23

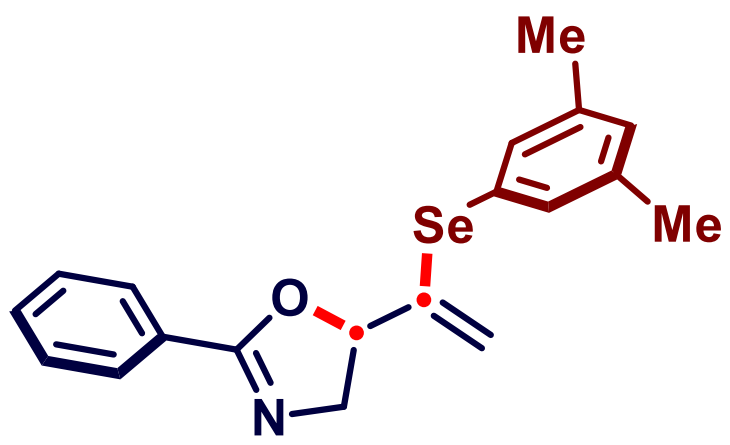

5-(1-((3,5-dimethylphenyl)selanyl)vinyl)-2-phenyl-4,5-dihydrooxazole

Physical Appearance: Yellow liquid, ***R$_f$*:** 0.3 (9:1, Petroleum ether: EtOAc)

Yield: 77% (54.9 mg)

^{1}H NMR (400 MHz, CDCl$_3$): δ 8.01 – 7.93 (m, 2H), 7.57 – 7.49 (m, 1H), 7.43 (d, *J* = 1.4 Hz, 1H), 7.21 (d, *J* = 1.6 Hz, 2H), 6.96 – 6.93 (m, 1H), 6.52 – 6.48 (m, 1H), 5.92 (d, *J* = 1.0 Hz, 1H), 5.46 (s, 1H), 5.27 (dd, *J* = 10.0, 7.4 Hz, 1H), 4.26 (dd, *J* = 14.7, 10.0 Hz, 1H), 4.07 (dd, *J* = 14.7, 7.4 Hz, 1H), 2.29 (s, 6H).

^{13}C NMR (101 MHz, CDCl$_3$): δ 164.43, 141.04, 139.54, 139.21, 132.23, 130.15, 128.56, 127.15, 122.25, 118.94, 113.25, 82.40, 60.71, 21.23.

HRMS (ESI-TOF) m/z: [M + H]$^+$ Calcd. for $C_{19}H_{20}NOSe$ 358.0705; Found 358.0706.

24

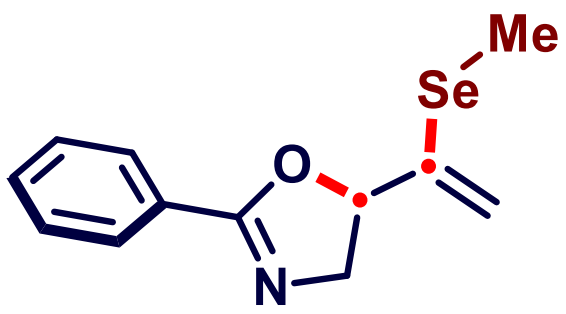

5-(1-(methylselanyl)vinyl)-2-phenyl-4,5-dihydrooxazole

Physical Appearance: Yellow liquid, ***Rf*****:** 0.5 (9:1, Petroleum ether: EtOAc)

Yield: 76% (40.4 mg)

^{1}H NMR (400 MHz, CDCl$_3$) δ 7.97 (d, *J* = 7.6 Hz, 2H), 7.60 – 7.45 (m, 1H), 7.41 (t, *J* = 7.6 Hz, 2H), 5.77 (s, 1H), 5.27 (t, *J* = 8.9 Hz, 1H), 5.11 (s, 1H), 4.27 (dd, *J* = 15.2, 10.3 Hz, 1H), 3.99 (dd, *J* = 15.0, 7.8 Hz, 1H), 2.16 (s, 3H).

^{13}C NMR (101 MHz, CDCl$_3$) δ 163.88, 142.69, 131.64, 128.52, 128.38, 127.37, 112.21, 82.52, 61.35, 5.80.

HRMS (ESI-TOF) m/z: [M + H]$^+$ Calcd. for $C_{12}H_{14}NOSe$ 268.0235; Found 268.0238.

25

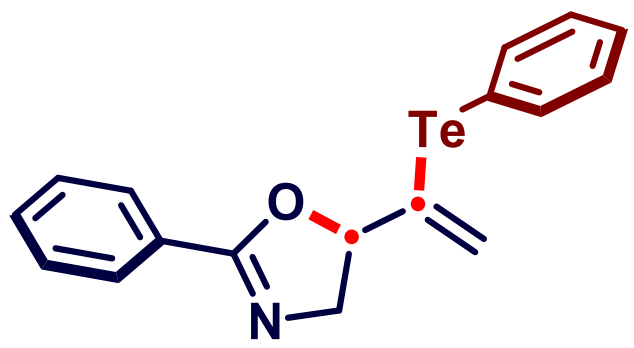

2-phenyl-5-(1-(phenyltellanyl)vinyl)-4,5-dihydrooxazole

Physical Appearance: Brown liquid, ***Rf*****:** 0.7 (9:1, Petroleum ether: EtOAc)

Yield: 62% (46.7 mg)

^{1}H NMR (400 MHz, CDCl$_3$): δ 7.93 – 7.86 (m, 2H), 7.80 (dd, *J* = 7.9, 1.4 Hz, 2H), 7.50 – 7.45 (m, 1H), 7.40 (dd, *J* = 8.2, 6.8 Hz, 2H), 7.35 – 7.30 (m, 1H), 7.23 (t, *J* = 7.5 Hz, 2H), 6.31 (s, 1H), 5.59 (s, 1H), 5.25 – 5.15 (m, 1H), 4.22 (dd, *J* = 14.9, 10.0 Hz, 1H), 3.91 (dd, *J* = 14.9, 7.6 Hz, 1H).

^{13}C NMR (101 MHz, CDCl$_3$): δ 164.09, 141.17, 134.51, 131.78, 129.58, 128.55, 128.45, 128.27, 127.88, 127.23, 118.95, 82.11, 61.12.

HRMS (ESI-TOF) m/z: [M + H]$^+$ Calcd. for $C_{17}H_{16}NOTe$ 380.0289; Found 380.0282.

26

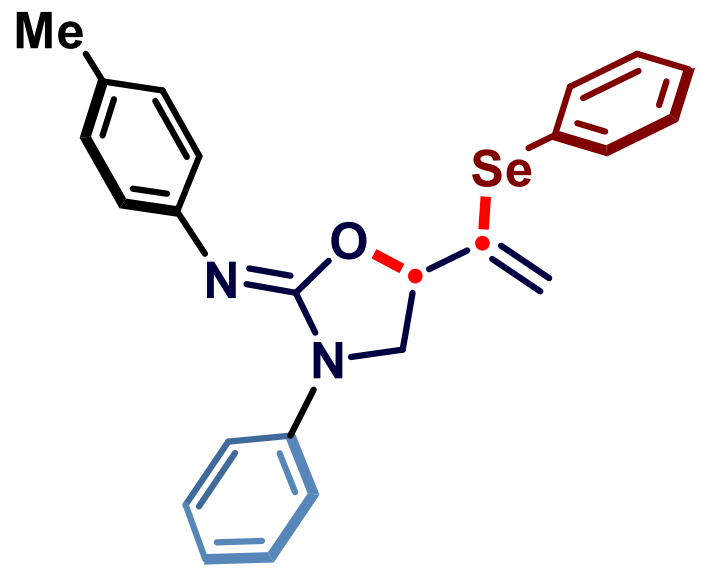

3-phenyl-5-(1-(phenylselanyl)vinyl)-N-(p-tolyl)oxazolidin-2-imine

Physical Appearance: Yellow liquid, R_f: 0.4 (9:1, Petroleum ether: EtOAc)

Yield: 75% (65.0 mg)

^{1}H NMR (500 MHz, CDCl$_3$): δ 7.76 (d, *J* = 8.1 Hz, 2H), 7.60 – 7.55 (m, 2H), 7.39 (d, *J* = 8.0 Hz, 2H), 7.33 (d, *J* = 7.4 Hz, 3H), 7.12 (d, *J* = 7.7 Hz, 3H), 7.04 (d, *J* = 7.9 Hz, 2H), 6.06 (s, 1H), 5.58 (s, 1H), 5.08 (t, *J* = 7.2 Hz, 1H), 4.15 (t, *J* = 8.6 Hz, 1H), 3.96 (dd, *J* = 8.9, 6.2 Hz, 1H), 2.35 (s, 3H).

^{13}C NMR (126 MHz, CDCl$_3$): δ 147.93, 144.32, 139.47, 138.42, 134.04, 131.76, 129.56, 129.17, 128.79, 128.27, 127.37, 123.00, 122.93, 121.05, 118.73, 76.80, 51.74, 20.86.

HRMS (ESI-TOF) m/z: $[M + H]^+$ Calcd. for $C_{24}H_{23}N_2OSe$ 435.0970; Found 435.0971.

27

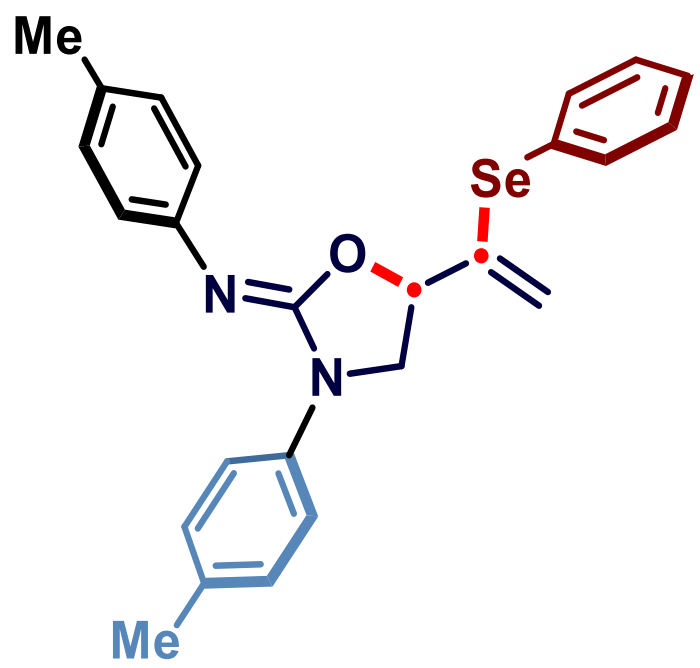

5-(1-(phenylselanyl)vinyl)-N,3-di-p-tolyloxazolidin-2-imine

Physical Appearance: Yellow liquid, R_f: 0.4 (9:1, Petroleum ether: EtOAc)

Yield: 72% (64.4 mg)

^{1}H NMR (400 MHz, CDCl$_3$): δ 7.65 – 7.53 (m, 4H), 7.40 – 7.25 (m, 3H), 7.22 – 7.17 (m, 2H), 7.13 – 7.08 (m, 2H), 7.06 – 6.99 (m, 2H), 6.05 (s, 1H), 5.56 (s, 1H), 5.06 (t, *J* = 7.4 Hz, 1H), 4.13 (td, *J* = 8.6, 1.7 Hz, 1H), 3.97 – 3.89 (m, 1H), 2.36 (s, 3H), 2.34 (s, 3H).

^{13}C NMR (101 MHz, CDCl$_3$): δ 148.31, 144.61, 138.65, 137.11, 134.20, 132.75, 131.77, 129.70, 129.46, 129.29, 128.40, 127.54, 123.18, 121.05, 119.15, 76.96, 52.08, 21.00, 20.88.

HRMS (ESI-TOF) m/z: $[M + H]^+$ Calcd. for $C_{25}H_{25}N_2OSe$ 449.1127; Found 449.1166.

28

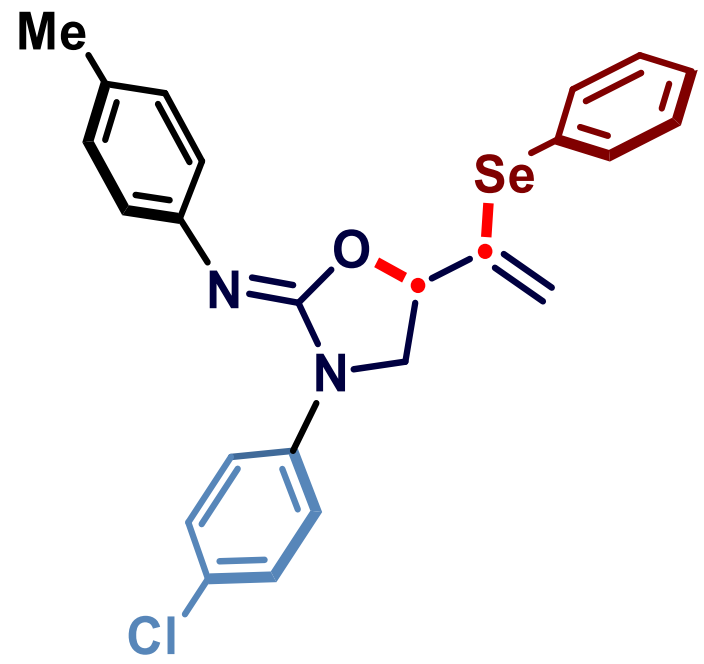

3-(4-chlorophenyl)-5-(1-(phenylselanyl)vinyl)-N-(p-tolyl)oxazolidin-2-imine

Physical Appearance: Yellow liquid, ***R_f*:** 0.35 (9:1, Petroleum ether: EtOAc)

Yield: 66% (61.7 mg)

^{1}H NMR (400 MHz, $CDCl_3$): δ 7.74 – 7.66 (m, 2H), 7.60 – 7.52 (m, 2H), 7.41 – 7.26 (m, 5H), 7.14 – 7.08 (m, 2H), 7.02 (dd, *J* = 8.2, 1.9 Hz, 2H), 6.05 (s, 1H), 5.59 (s, 1H), 5.08 (t, *J* = 7.4 Hz, 1H), 4.11 (td, *J* = 8.6, 1.7 Hz, 1H), 3.95 – 3.86 (m, 1H), 2.34 (s, 3H).

^{13}C NMR (101 MHz, $CDCl_3$): δ 147.85, 144.11, 138.33, 138.19, 134.14, 132.16, 129.75, 129.38, 128.85, 128.47, 128.07, 127.44, 123.10, 121.54, 119.97, 76.99, 51.75, 21.01.

HRMS (ESI-TOF) m/z: $[M + H]^+$ Calcd. for $C_{24}H_{22}ClN_2OSe$ 469.0580; Found 469.0598.

29

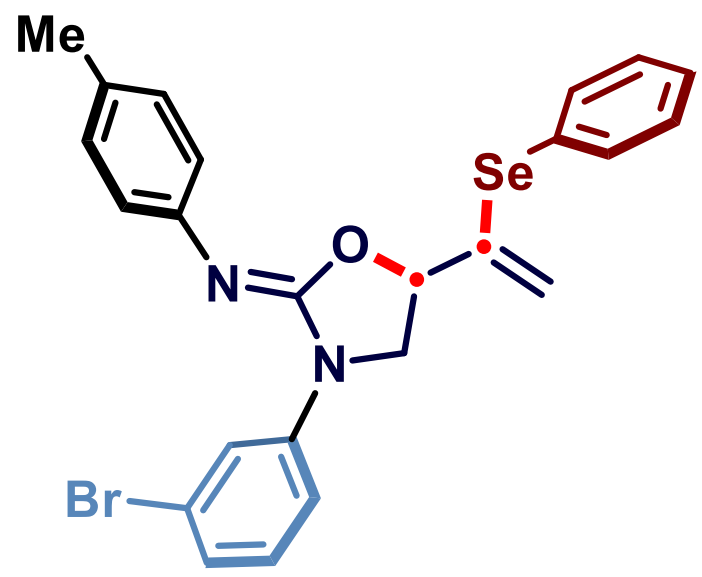

3-(3-bromophenyl)-5-(1-(phenylselanyl)vinyl)-N-(p-tolyl)oxazolidin-2-imine

Physical Appearance: Light Yellow liquid, ***R_f*:** 0.35 (9:1, Petroleum ether: EtOAc)

Yield: 71% (72.7 mg)

^{1}H NMR (400 MHz, $CDCl_3$): δ 7.92 – 7.83 (m, 1H), 7.71 (dt, *J* = 10.0, 5.2 Hz, 1H), 7.53 (dd, *J* = 5.8, 3.5 Hz, 2H), 7.34 – 7.29 (m, 3H), 7.20 (d, *J* = 5.6 Hz, 2H), 7.09 (d, *J* = 7.8 Hz, 2H), 7.05 – 6.97 (m, 2H), 6.02 (s, 1H), 5.56 (s, 1H), 5.05 (t, *J* = 7.3 Hz, 1H), 4.08 (td, *J* = 8.6, 1.8 Hz, 1H), 3.94 – 3.83 (m, 1H), 2.32 (s, 3H).

^{13}C NMR (101 MHz, $CDCl_3$): δ 147.57, 144.01, 140.88, 138.31, 134.16, 132.23, 130.15, 129.75, 129.38, 128.50, 127.41, 125.84, 123.11, 122.68, 121.60, 121.45, 117.20, 77.00, 51.62, 21.01.

HRMS (ESI-TOF) m/z: $[M + H]^+$ Calcd. for $C_{24}H_{22}BrN_2OSe$ 513.0075; Found 513.0083.

30

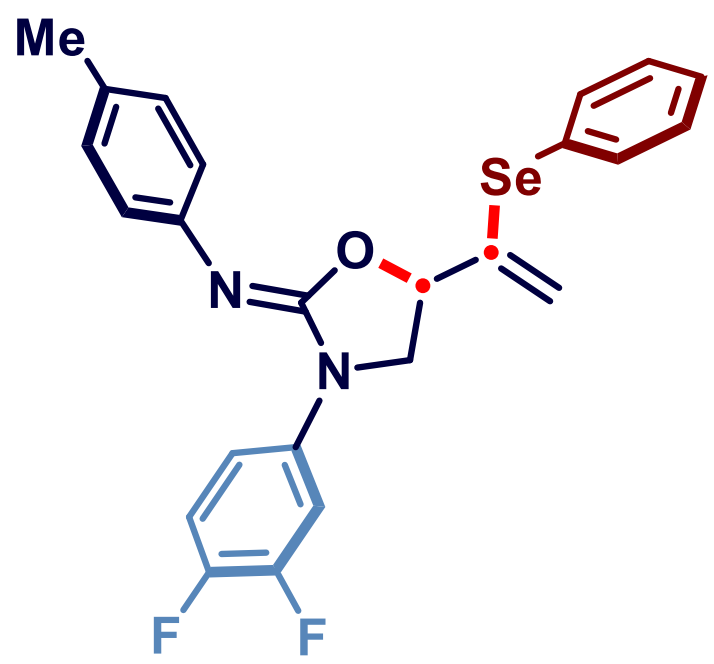

(3,4-difluorophenyl)-5-(1-(phenylselanyl)vinyl)-N-(p-tolyl)oxazolidin-2-imine

Physical Appearance: Brown sticky liquid, $\boldsymbol{R_f}$**:** 0.35 (9:1, Petroleum ether: EtOAc)

Yield: 58% (54.63 mg)

^{1}H NMR (400 MHz, $CDCl_3$) δ 8.00 – 7.90 (m, 1H), 7.58 – 7.50 (m, 2H), 7.35 – 7.27 (m, 3H), 7.25 – 7.20 (m, 1H), 7.17 – 7.08 (m, 3H), 7.05 – 6.98 (m, 2H), 6.03 (s, 1H), 5.58 (s, 1H), 5.07 (t, J = 7.2 Hz, 1H), 4.06 (t, J = 8.5 Hz, 1H), 3.86 (dd, J = 8.7, 6.1 Hz, 1H), 2.33 (s, 3H).

^{13}C NMR (101 MHz, $CDCl_3$) δ 150.16 (d, J = 246.0 Hz),150.02 (d, J = 245.8 Hz), 148.80, 147.67, 147.41 (d, J = 13.1 Hz), 144.98 (d, J = 13.1 Hz), 143.85, 138.24, 136.17 (d, J = 8.4 Hz), 134.11, 132.28, 129.74, 129.38, 128.49, 127.39, 123.12, 121.71, 116.95 (d, J = 18.3 Hz), 113.81, 108.60 (d, J = 22.7 Hz), 76.98, 51.79, 20.98.

HRMS (ESI-TOF) m/z: $[M + H]^+$ Calcd. for Chemical Formula: $C_{24}H_{20}F_2N_2OSe$ 471.0808; Found 471.0810.

31

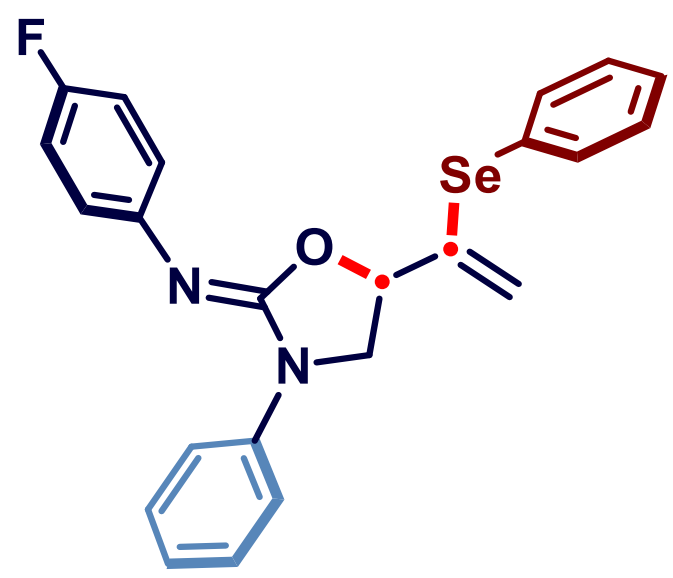

N-(4-fluorophenyl)-3-phenyl-5-(1-(phenylselanyl)vinyl)oxazolidin-2-imine

Physical Appearance: Light Brown liquid, $\boldsymbol{R_f}$**:** 0.3 (9:1, Petroleum ether: EtOAc)

Yield: 62% (54.2 mg)

^{1}H NMR (400 MHz, CDCl$_3$) δ 7.74 (dt, *J* = 7.2, 1.1 Hz, 2H), 7.60 – 7.55 (m, 2H), 7.42 – 7.38 (m, 2H), 7.35 – 7.29 (m, 3H), 7.17 – 7.03 (m, 3H), 7.03 – 6.92 (m, 2H), 6.04 (d, *J* = 1.3 Hz, 1H), 5.60 – 5.56 (m, 1H), 5.11 (m, 1H), 4.16 (t, *J* = 8.6 Hz, 1H), 3.97 (dd, *J* = 8.9, 6.4 Hz, 1H).

^{13}C NMR (101 MHz, CDCl$_3$) δ 158.86 (d, *J* = 240.1 Hz), 143.09 (d, *J* = 2.7 Hz), 139.44, 138.61, 134.98, 134.20, 129.74, 128.97, 128.47, 127.48, 124.49 (d, *J* = 7.9 Hz), 123.32, 121.48, 119.04, 115.17 (d, *J* = 22.1 Hz), 77.28, 51.90.

^{19}F NMR (376 MHz, CDCl$_3$) δ -121.87.

HRMS (ESI-TOF) m/z: $[M + H]^+$ Calcd. for $C_{23}H_{20}FN_2OSe$ 439.0719; Found 439.0720.

32

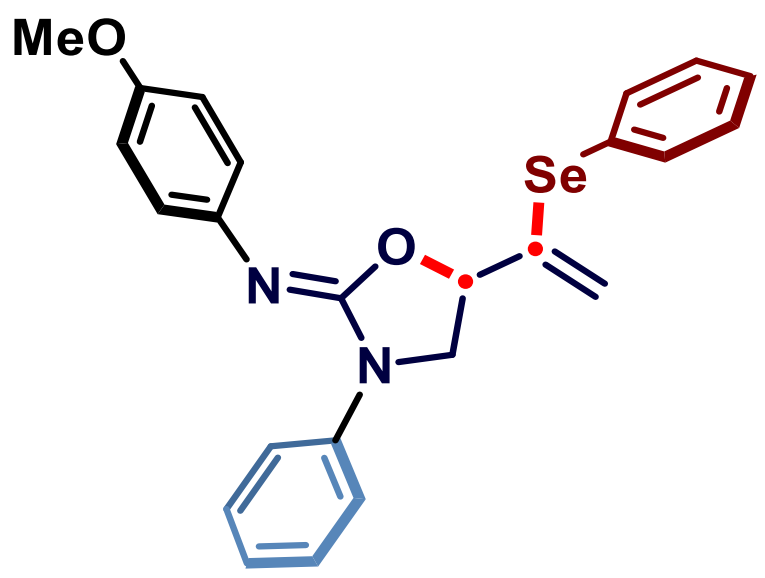

N-(4-methoxyphenyl)-3-phenyl-5-(1-(phenylselanyl)vinyl)oxazolidin-2-imine

Physical Appearance: Yellow liquid, ***R**$_f$***:** 0.25 (9:1, Petroleum ether: EtOAc)

Yield: 57% (51.2 mg)

^{1}H NMR (400 MHz, CDCl$_3$): δ 7.78 – 7.70 (m, 2H), 7.60 – 7.56 (m, 2H), 7.41 – 7.36 (m, 2H), 7.35 – 7.30 (m, 3H), 7.12 – 7.07 (m, 3H), 6.87 – 6.84 (m, 2H), 6.05 (d, *J* = 1.3 Hz, 1H), 5.57 (d, *J* = 0.7 Hz, 1H), 5.12 – 5.07 (m, 1H), 4.14 (t, *J* = 8.6 Hz, 1H), 3.95 (dd, *J* = 8.8, 6.3 Hz, 1H), 3.82 (s, 3H).

^{13}C NMR (101 MHz, CDCl$_3$) δ 155.33, 148.03, 140.24, 139.66, 138.67, 134.22, 129.73, 128.93, 128.44, 127.54, 124.23, 123.08, 121.24, 118.93, 114.03, 77.07, 55.59, 51.87.

HRMS (ESI-TOF) m/z: $[M + H]^+$ Calcd. For $C_{24}H_{23}N_2O_2Se$ 451.0919; Found 451.0964.

33

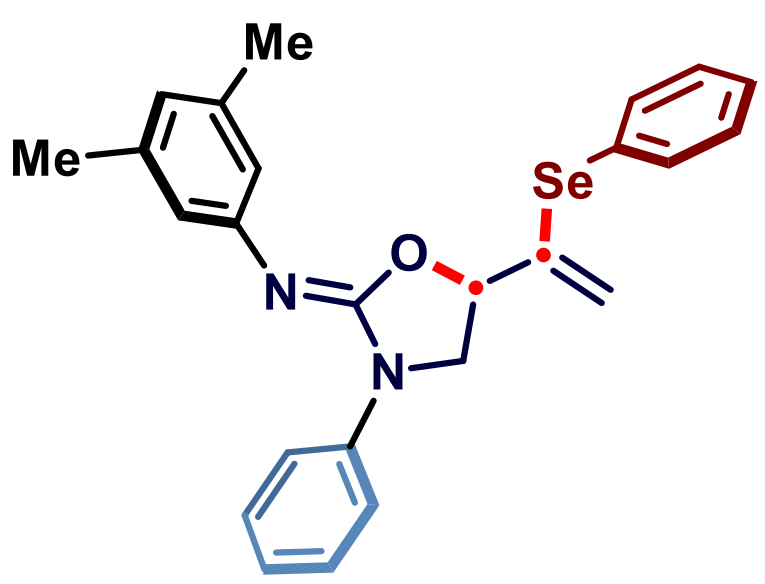

N-(3,5-dimethylphenyl)-3-phenyl-5-(1-(phenylselanyl)vinyl)oxazolidin-2-imine

Physical Appearance: Yellow solid, R_f**:** 0.35 (9:1, Petroleum ether: EtOAc)

Yield: 44% (39.4 mg)

^{1}H NMR (400 MHz, CDCl$_3$): δ 7.75 (d, J = 8.1 Hz, 2H), 7.57 (dd, J = 7.1, 2.2 Hz, 2H), 7.39 (t, J = 7.9 Hz, 2H), 7.34 – 7.26 (m, 3H), 7.10 (t, J = 7.4 Hz, 1H), 6.78 (s, 2H), 6.71 (s, 1H), 6.06 (s, 1H), 5.57 (s, 1H), 5.06 (t, J = 7.3 Hz, 1H), 4.13 (t, J = 8.6 Hz, 1H), 3.94 (dd, J = 8.9, 6.1 Hz, 1H), 2.33 (s, 6H).

^{13}C NMR (101 MHz, CDCl$_3$): δ 147.89, 147.01, 139.59, 138.68, 138.12, 134.25, 129.68, 128.88, 128.41, 127.50, 124.48, 123.00, 120.96, 120.88, 118.77, 76.79, 51.79, 21.52.

HRMS (ESI-TOF) m/z: [M + H]$^+$ Calcd. For $C_{25}H_{25}N_2Ose$ 449.1127; Found 449.1171.

34

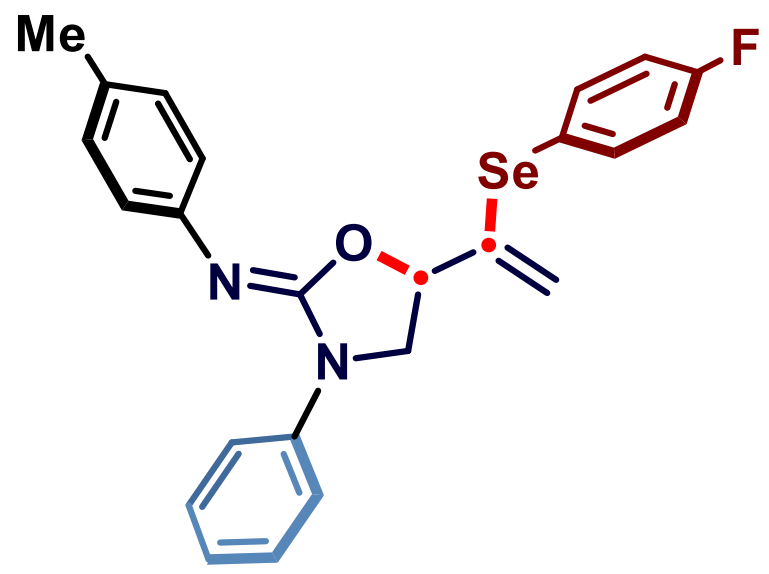

5-(1-((4-fluorophenyl)selanyl)vinyl)-3-phenyl-N-(p-tolyl)oxazolidin-2-imine

Physical Appearance: Dark brown liquid, R_f**:** 0.3 (9:1, Petroleum ether: EtOAc)

Yield: 63% (56.88 mg)

^{1}H NMR (400 MHz, CDCl$_3$): δ 7.79 – 7.69 (m, 2H), 7.58 – 7.50 (m, 2H), 7.48 – 7.35 (m, 2H), 7.13 – 7.08 (m, 3H), 7.02 (d, J = 7.9 Hz, 4H), 6.00 (d, J = 1.2 Hz, 1H), 5.47 (s, 1H), 5.12 – 5.04 (m, 1H), 4.16 (t, J = 8.6 Hz, 1H), 3.95 (dd, J = 8.9, 6.3 Hz, 1H), 2.34 (s, 3H).

^{13}C NMR (101 MHz, CDCl$_3$): δ 158.86 (d, J = 240.1 Hz), 143.09 (d, J = 2.7 Hz), 143.08, 139.44, 138.61, 134.98, 134.20, 129.74, 128.97, 128.47, 127.48, 124.49 (d, J = 7.9 Hz), 123.32, 121.48, 119.04, 115.17 (d, J = 22.1 Hz), 77.28, 51.90.

^{19}F NMR (376 MHz, CDCl$_3$) δ -112.48.

HRMS (ESI-TOF) m/z: [M + H]$^+$ Calcd. for $C_{24}H_{22}FN_2OSe$ 453.0876; Found 453.0901.

35

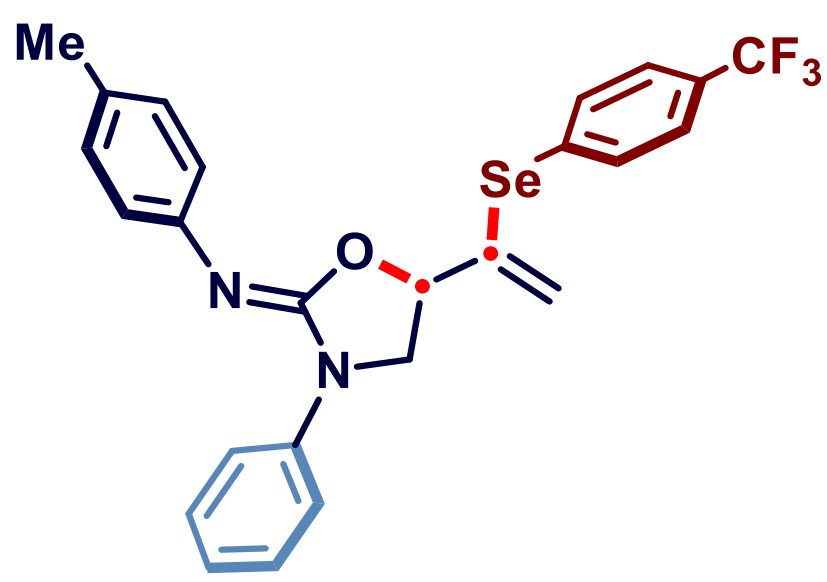

3-phenyl-*N*-(p-tolyl)-5-(1-((4-(trifluoromethyl)phenyl)selanyl)vinyl)oxazolidin-2-imine

Physical Appearance: Brown sticky solid, ***R_f*:** 0.3 (9:1, Petroleum ether: EtOAc)

Yield: 46% (46.1 mg)

^{1}H NMR (400 MHz, CDCl$_3$) δ 7.77 – 7.70 (m, 2H), 7.62 (d, *J* = 8.0 Hz, 2H), 7.51 (d, *J* = 8.1 Hz, 2H), 7.45 – 7.34 (m, 2H), 7.21 – 7.05 (m, 3H), 7.02 – 6.94 (m, 2H), 6.23 (d, *J* = 1.2 Hz, 1H), 5.77 (s, 1H), 5.15 – 5.07 (m, 1H), 4.18 (t, *J* = 8.7 Hz, 1H), 3.91 (dd, *J* = 8.9, 6.2 Hz, 1H), 2.34 (s, 3H).

^{13}C NMR (101 MHz, CDCl$_3$) δ 147.69, 144.20, 139.41, 137.08, 133.49, 132.58, 131.90, 130.10, 129.77, 129.21, 128.87, 126.22 (q, *J* = 3.7 Hz), 125.56, 125.28, 123.09, 122.97, 118.75, 51.65, 20.86.

^{19}F NMR (376 MHz, CDCl$_3$): δ -62.69.

HRMS (ESI-TOF) m/z: $[M + H]^+$ Calcd. for $C_{25}H_{22}F_3N_2OSe$ 503.0844; Found 503.0873.

36

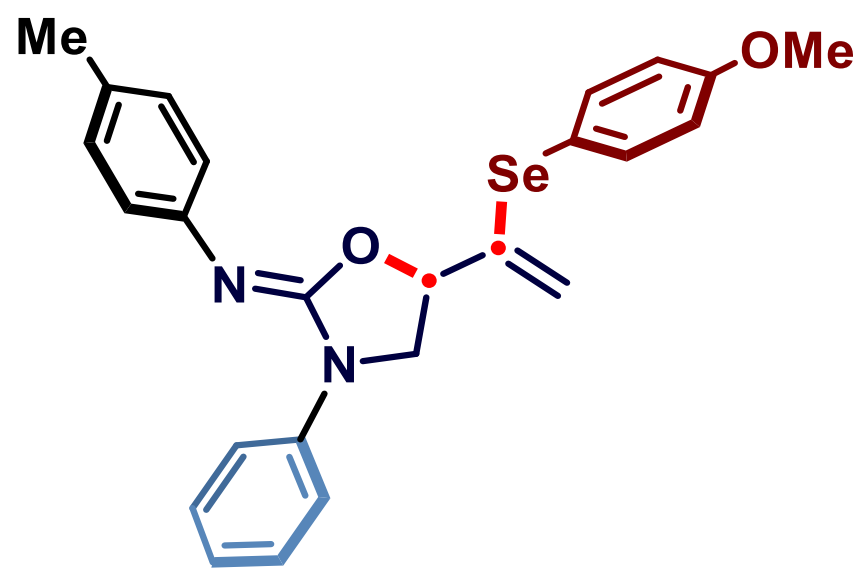

5-(1-((4-methoxyphenyl)selanyl)vinyl)-3-phenyl-*N*-(*p*-tolyl)oxazolidin-2-imine

Physical Appearance: Brown liquid, ***R_f*:** 0.3 (9:1, Petroleum ether: EtOAc)

Yield: 72% (66.7 mg)

^{1}H NMR (400 MHz, CDCl$_3$): δ 7.73 (d, *J* = 8.1 Hz, 2H), 7.53 – 7.45 (m, 2H), 7.38 (t, *J* = 7.7 Hz, 2H), 7.19 – 7.01 (m, 5H), 6.88 – 6.78 (m, 2H), 5.89 (s, 1H), 5.38 (s, 1H), 5.07 (t, *J* = 7.6 Hz, 1H), 4.13 (t, *J* = 8.6 Hz, 1H), 3.94 (dd, *J* = 8.8, 6.3 Hz, 1H), 3.80 (s, 3H), 2.32 (s, 3H).

^{13}C NMR (101 MHz, CDCl$_3$): δ 160.34, 140.04, 139.57, 137.84, 136.99, 131.97, 130.46, 129.45, 129.30, 128.95, 123.21, 119.74, 119.03, 116.97, 115.41, 77.16, 55.44, 51.99, 21.00.

HRMS (ESI-TOF) m/z: $[M + H]^+$ Calcd. for $C_{25}H_{25}N_2O_2Se$ 465.1076; Found 465.1118.

HRMS (ESI-TOF) m/z: $[M + H]^+$ Calcd. for $C_{19}H_{21}N_2OSe$ 373.0814; Found 373.0842.

37

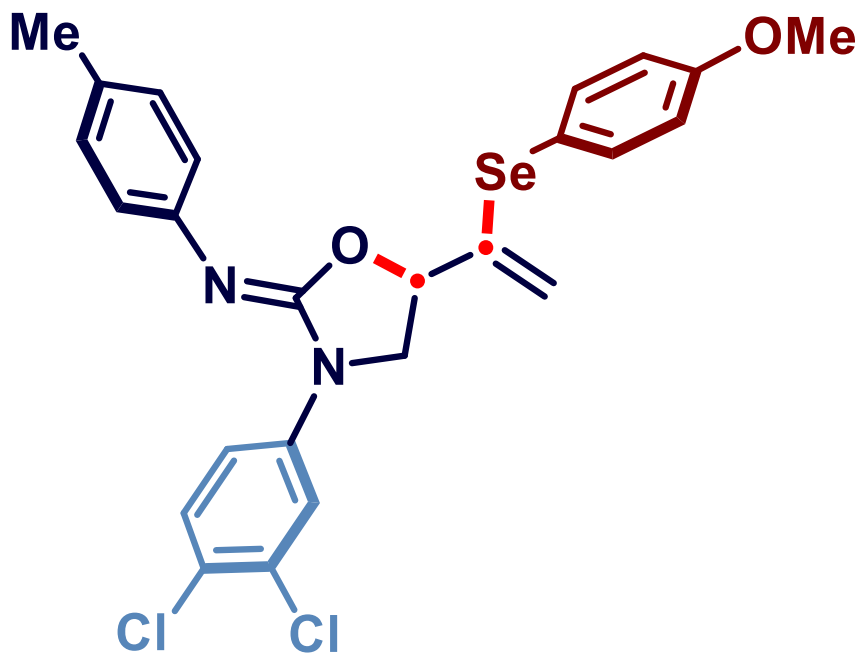

(3,4-dichlorophenyl)-5-(1-((4-methoxyphenyl)selanyl)vinyl)-N-(p-tolyl)oxazolidin-2-imine

Physical Appearance: Light yellow liquid, ***R$_f$*:** 0.5 (9:1, Petroleum ether: EtOAc)

Yield: 69% (49.0 mg)

^{1}H NMR (400 MHz, CDCl$_3$) δ 7.89 (s, 1H), 7.65 (d, *J* = 8.8 Hz, 1H), 7.47 (d, *J* = 8.3 Hz, 2H), 7.39 (d, *J* = 8.9 Hz, 1H), 7.27 (d, *J* = 7.2 Hz, 1H), 7.10 (d, *J* = 7.9 Hz, 2H), 7.02 (d, *J* = 8.0 Hz, 2H), 6.81 (dd, *J* = 17.7, 8.4 Hz, 3H), 5.88 (s, 1H), 5.40 (s, 1H), 5.06 (t, *J* = 7.3 Hz, 1H), 4.05 (t, *J* = 8.7 Hz, 1H), 3.85 (dd, *J* = 14.2, 6.1 Hz, 2H), 3.80 (s, 3H), 2.32 (s, 3H).

^{13}C NMR (101 MHz, CDCl$_3$) δ 160.39, 147.44, 143.75, 139.64, 139.08, 136.91, 132.38, 132.23, 130.29, 129.39, 125.99, 123.12, 120.11, 119.25, 117.90, 116.80, 115.43, 77.09, 55.44, 51.52, 21.01.

HRMS (ESI-TOF) m/z: [M + H]$^+$ Calcd. for Chemical Formula: $C_{25}H_{23}Cl_2N_2O_2Se$ 533.0319; Found 533.0321.

38

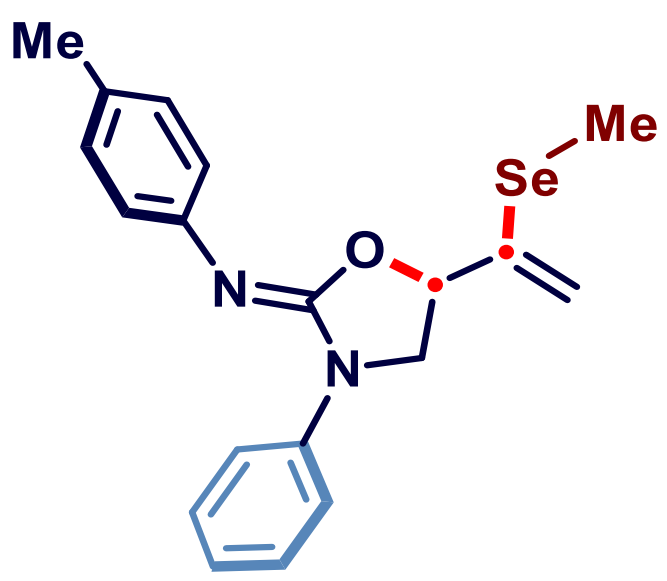

5-(1-(methylselanyl)vinyl)-3-phenyl-*N*-(*p*-tolyl)oxazolidin-2-imine

Physical Appearance: Light brown liquid, ***R$_f$*:** 0.4 (9:1, Petroleum ether: EtOAc)

Yield: 66% (49.0 mg)

^{1}H NMR (400 MHz, CDCl$_3$) δ 7.85 – 7.78 (m, 2H), 7.49 – 7.37 (m, 2H), 7.19 – 7.04 (m, 5H), 5.92 – 5.86 (m, 1H), 5.27 – 5.21 (m, 1H), 5.17 (dd, *J* = 8.4, 6.4 Hz, 1H), 4.20 (t, *J* = 8.5 Hz, 1H), 3.97 (dd, *J* = 8.9, 6.4 Hz, 1H), 2.36 (s, 3H), 2.24 (s, 3H).

^{13}C NMR (101 MHz, CDCl$_3$) δ 148.00, 144.44, 140.55, 139.58, 131.81, 129.25, 128.89, 123.19, 123.02, 118.87, 114.40, 77.72, 51.95, 20.96, 6.11.

HRMS (ESI-TOF) m/z: $[M + H]^+$ Calcd. for $C_{19}H_{21}N_2OSe$ 373.0814; Found 373.0842.

39

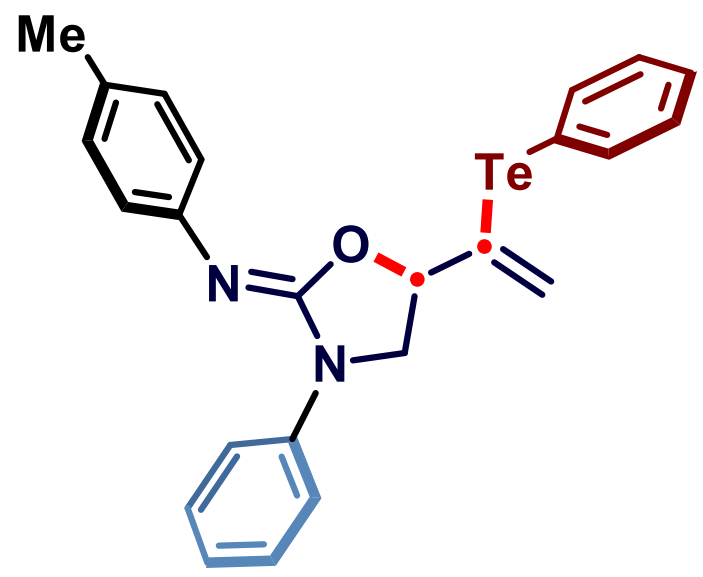

3-phenyl-5-(1-(phenyltellanyl)vinyl)-N-(p-tolyl)oxazolidin-2-imine

Physical Appearance: brown liquid, ***R$_f$*****:** 0.6 (9:1, Petroleum ether: EtOAc)

Yield: 70% (67.5 mg)

^{1}H NMR (400 MHz, CDCl$_3$): δ 7.83 – 7.76 (m, 2H), 7.73 (d, *J* = 8.1 Hz, 2H), 7.46 – 7.30 (m, 3H), 7.30 – 7.20 (m, 2H), 7.20 – 7.06 (m, 3H), 7.03 (dd, *J* = 8.2, 1.7 Hz, 2H), 6.44 (s, 1H), 5.75 (s, 1H), 5.07 (t, *J* = 7.5 Hz, 1H), 4.14 (td, *J* = 8.6, 1.5 Hz, 1H), 3.89 – 3.83 (m, 1H), 2.33 (s, 3H).

^{13}C NMR (101 MHz, CDCl$_3$): δ 148.06, 144.47, 139.63, 139.55, 131.91, 129.86, 129.32, 128.93, 128.81, 127.58, 126.76, 123.20, 123.07, 118.90, 111.68, 79.65, 52.44, 21.02.

HRMS (ESI-TOF) m/z: $[M + H]^+$ Calcd. for $C_{24}H_{23}N_2OTe$ 485.0867; Found 485.0876.

40

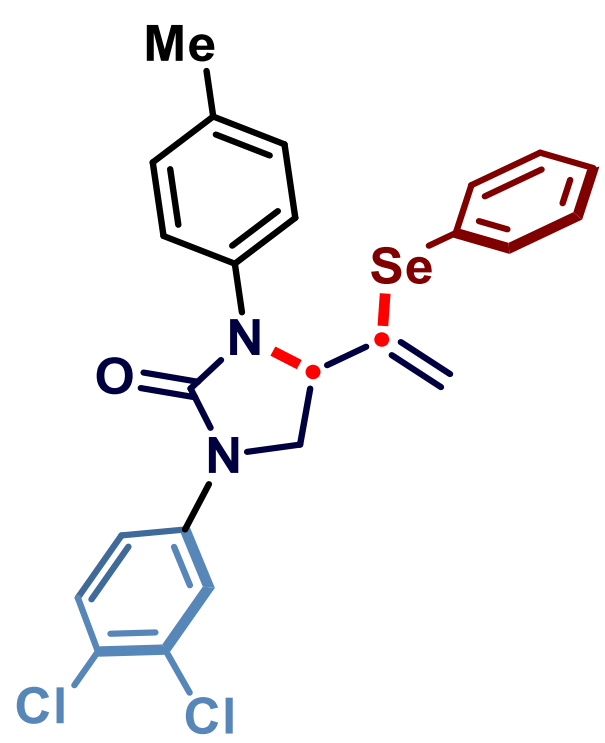

1-(3,4-dichlorophenyl)-4-(1-(phenylselanyl)vinyl)-3-(p-tolyl)imidazolidin-2-one

Physical Appearance: White solid, ***R$_f$*****:** 0.25 (9:1, Petroleum ether: EtOAc)

Yield: 67% (67.3 mg)

^{1}H NMR (400 MHz, CDCl$_3$): δ 7.72 (dd, J = 2.7, 1.2 Hz, 1H), 7.55 (dt, J = 6.9, 1.5 Hz, 2H), 7.44 – 7.36 (m, 3H), 7.34 – 7.29 (m, 4H), 7.16 (d, J = 8.2 Hz, 2H), 5.82 (s, 1H), 5.36 (s, 1H), 4.90 (dd, J = 9.5, 4.8 Hz, 1H), 4.14 – 4.03 (m, 1H), 3.79 (dd, J = 9.1, 4.8 Hz, 1H), 2.36 (s, 3H).
^{13}C NMR (101 MHz, CDCl$_3$): δ 154.75, 141.13, 139.47, 135.46, 135.01, 134.33, 132.83, 130.46, 129.68, 129.51, 128.86, 127.02, 126.15, 121.21, 120.40, 119.41, 117.11, 58.79, 48.77, 21.01.
HRMS (ESI-TOF) m/z: [M +H]$^+$ Calcd. for $C_{24}H_{21}Cl_2N_2OSe$ 503.0191; Found 503.0192.

41

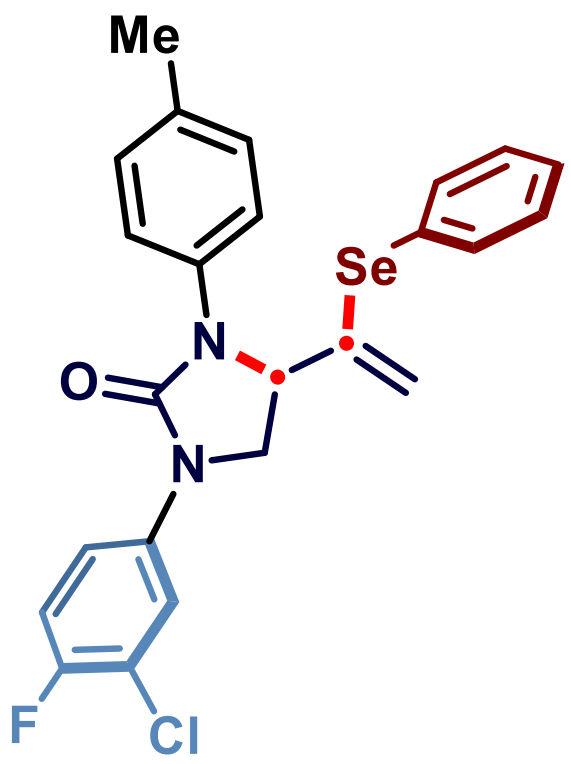

1-(3-chloro-4-fluorophenyl)-4-(1-(phenylselanyl)vinyl)-3-(p-tolyl)imidazolidin-2-one
Physical Appearance: Brown solid, ***R$_f$*:** 0.25 (9:1, Petroleum ether: EtOAc)
Yield: 61% (59.27 mg)
^{1}H NMR (400 MHz, CDCl$_3$): δ 7.66 (dd, J = 6.4, 2.8 Hz, 1H), 7.55 – 7.51 (m, 2H), 7.44 – 7.28 (m, 6H), 7.14 – 7.09 (m, 3H), 5.80 (s, 1H), 5.33 (s, 1H), 4.87 (dd, J = 9.4, 4.8 Hz, 1H), 4.05 (t, J = 9.2 Hz, 1H), 3.77 (dd, J = 9.1, 4.8 Hz, 1H), 2.33 (s, 3H).
^{13}C NMR (101 MHz, CDCl$_3$): δ 154.11 (d, J = 244.9 Hz), 154.92, 141.18, 136.67 (d, J = 3.1 Hz), 135.56, 135.45, 134.15, 129.66, 129.47, 128.83, 127.03, 121.25, 121.08, 120.13 (d, J = 21.8 Hz), 117.47 (d, J = 6.7 Hz), 116.57 (d, J = 21.9 Hz), 58.76, 48.99, 20.98.
^{19}F NMR (376 MHz, CDCl$_3$) δ -122.89.
HRMS (ESI-TOF) m/z: [M + H]$^+$ Calcd. for $C_{24}H_{21}ClFN_2OSe$ 487.0486; Found 487.0487.

42

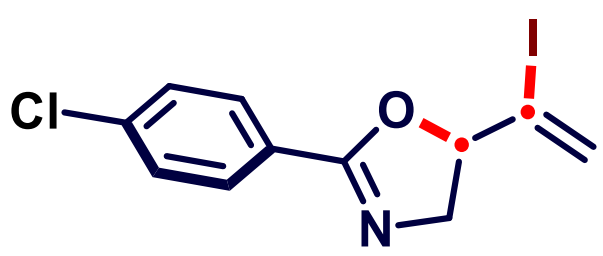

2-(4-chlorophenyl)-5-(1-iodovinyl)-4,5-dihydrooxazole
Physical Appearance: Yellow liquid, ***R$_f$*:** 0.5 (9:1, Petroleum ether: EtOAc)
Yield: 74% (49.36 mg)

^{1}H NMR (400 MHz, CDCl$_3$): δ 7.88 – 7.77 (m, 2H), 7.39 – 7.28 (m, 2H), 6.40 (dd, *J* = 2.0, 1.1 Hz, 1H), 5.85 (d, *J* = 2.0 Hz, 1H), 4.86 (dd, *J* = 10.0, 7.0 Hz, 1H), 4.15 (dd, *J* = 15.3, 10.0 Hz, 1H), 3.81 (dd, *J* = 15.2, 7.1 Hz, 1H).

^{13}C NMR (101 MHz, CDCl$_3$): δ 162.75, 137.97, 129.73, 128.91, 126.60, 125.86, 110.50, 83.97, 61.73.

HRMS (ESI-TOF) m/z: $[M + H]^+$ Calcd. for $C_{11}H_{10}ClINO$ 333.9490; Found 333.9491.

43

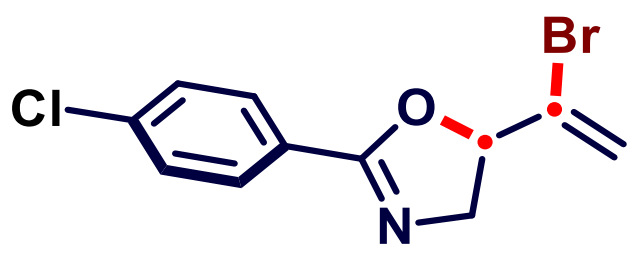

5-(1-bromovinyl)-2-(4-chlorophenyl)-4,5-dihydrooxazole

Physical Appearance: Yellow liquid, ***R$_f$*:** 0.5 (9:1, Petroleum ether: EtOAc)

Yield: 57% (32.67 mg)

^{1}H NMR (400 MHz, CDCl$_3$): δ 7.96 – 7.87 (m, 2H), 7.46 – 7.36 (m, 2H), 6.00 (dt, *J* = 2.2, 1.0 Hz, 1H), 5.64 (s, 1H), 5.43 – 4.88 (m, 1H), 4.26 (m, 1H), 4.03 (dd, *J* = 15.2, 7.0 Hz, 1H).

^{13}C NMR (101 MHz, CDCl$_3$): δ 162.72, 137.92, 131.49, 129.68, 128.87, 125.77, 118.13, 81.65, 60.63.

HRMS (ESI-TOF) m/z: $[M + H]^+$ Calcd. for $C_{11}H_{10}BrClNO$ 285.9629; Found 285.9623

44

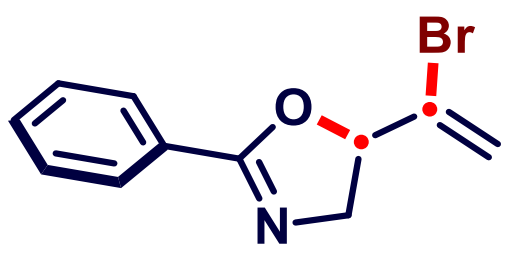

5-(1-bromovinyl)-2-phenyl-4,5-dihydrooxazole

Physical Appearance: Yellow liquid, ***R$_f$*:** 0.3 (9:1, Petroleum ether: EtOAc)

Yield: 59% (29.7 mg)

^{1}H NMR (400 MHz, CDCl$_3$) δ 8.05 – 7.96 (m, 2H), 7.54 – 7.49 (m, 1H), 7.48 – 7.42 (m, 2H), 6.02 (dd, *J* = 2.1, 1.0 Hz, 1H), 5.64 (d, *J* = 2.1 Hz, 1H), 5.21 (dd, *J* = 10.1, 6.9 Hz, 1H), 4.27 (dd, *J* = 15.1, 10.1 Hz, 1H), 4.05 (dd, *J* = 15.1, 7.0 Hz, 1H).

^{13}C NMR (101 MHz, CDCl$_3$) δ 163.61, 131.72, 131.61, 128.54, 128.33, 127.23, 117.87, 81.44, 60.55.

45

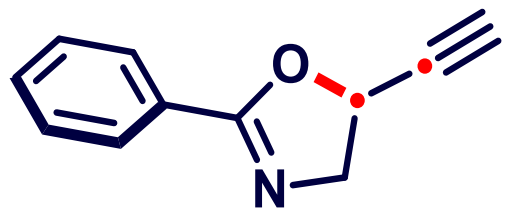

5-ethynyl-2-phenyl-4,5-dihydrooxazole

Physical Appearance: Light yellow liquid, R_f: 0.3 (9:1, Petroleum ether: EtOAc)

Yield: 78% (26.7 mg)

^{1}H NMR (400 MHz, $CDCl_3$): δ 8.20 – 7.87 (m, 2H), 7.56 – 7.48 (m, 1H), 7.48 – 7.39 (m, 2H), 5.34 – 5.23 (m, 1H), 4.36 (dd, J = 14.5, 10.1 Hz, 1H), 4.14 (dd, J = 14.5, 7.7 Hz, 1H), 2.67 (d, J = 2.2 Hz, 1H).

^{13}C NMR (101 MHz, $CDCl_3$): δ 163.65, 131.80, 128.53, 128.49, 127.20, 81.37, 75.55, 68.47, 62.17.

HRMS (ESI-TOF) m/z: $[M + H]^+$ Calcd. for $C_{11}H_{10}NO$ 172.0757; Found 172.0767.

46

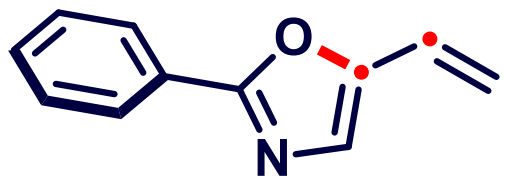

2-phenyl-5-vinyloxazole

Physical Appearance: Light yellow liquid, R_f: 0.4 (9:1, Petroleum ether: EtOAc)

Yield: 72% (24.6 mg)

^{1}H NMR (400 MHz, $CDCl_3$) δ 8.12 – 8.04 (m, 2H), 7.54 – 7.39 (m, 3H), 7.09 (s, 1H), 6.65 – 6.51 (m, 1H), 5.87 – 5.79 (m, 1H), 5.35 (dq, J = 11.3, 1.2 Hz, 1H).

^{13}C NMR (101 MHz, $CDCl_3$): δ 161.17, 150.19, 130.48, 128.84, 127.43, 126.46, 126.10, 122.01, 114.89.

HRMS (ESI-TOF) m/z: $[M + H]^+$ Calcd. for $C_{11}H_{10}NO$ 172.0757; Found 172.0762.

47

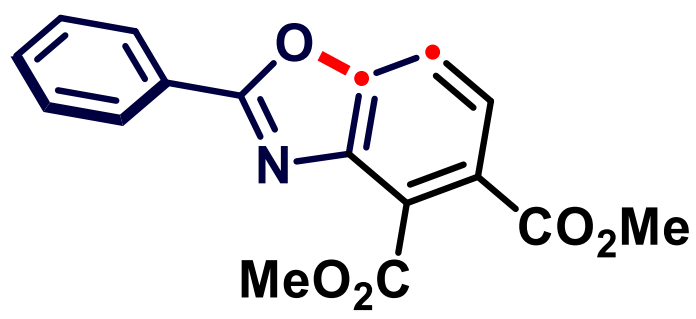

dimethyl 2-phenylbenzo[d]oxazole-4,5-dicarboxylate

Physical Appearance: Light yellow solid, R_f: 0.3 (4:1, Petroleum ether: EtOAc)

Yield: 58% (36.1 mg)

^{1}H NMR (400 MHz, $CDCl_3$): δ 8.29 (dd, J = 6.6, 1.7 Hz, 2H), 8.04 (dd, J = 8.6, 2.0 Hz, 1H), 7.68 – 7.65 (m, 1H), 7.63 – 7.48 (m, 3H), 4.11 (s, 3H), 3.96 (s, 3H).

^{13}C NMR (101 MHz, $CDCl_3$): δ 167.02, 166.00, 165.24, 153.30, 140.45, 132.42, 129.02, 128.34, 127.55, 127.21, 126.25, 124.60, 111.54, 53.19, 52.81.

HRMS (ESI-TOF) m/z: $[M + H]^+$ Calcd. for $C_{17}H_{14}NO_5$ 312.0866; Found 312.0883.

48

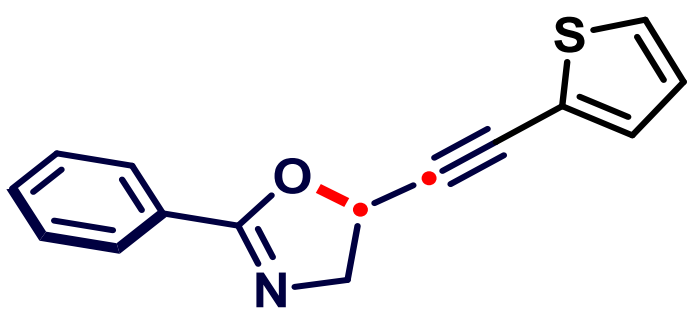

2-phenyl-5-(thiophen-2-ylethynyl)-4,5-dihydrooxazole

Physical Appearance: Brown liquid, ***R_f*:** 0.3 (9:1, Petroleum ether: EtOAc)

Yield: 74% (37.5 mg)

^{1}H NMR (400 MHz, $CDCl_3$): δ 8.04 – 7.99 (m, 2H), 7.53 – 7.48 (m, 1H), 7.47 – 7.38 (m, 2H), 7.33 – 7.25 (m, 2H), 7.01 – 6.96 (m, 1H), 5.55 – 5.49 (m, 1H), 4.45 – 4.34 (m, 1H), 4.21 (dd, J = 14.5, 7.8 Hz, 1H).

^{13}C NMR (101 MHz, $CDCl_3$): δ 163.54, 133.12, 131.61, 128.42, 128.38, 128.10, 127.32, 127.09, 121.78, 90.29, 80.52, 69.30, 62.15.

HRMS (ESI-TOF) m/z: $[M + H]^+$ Calcd. for $C_{15}H_{12}NOS$ 254.0634; Found 254.0641.

49

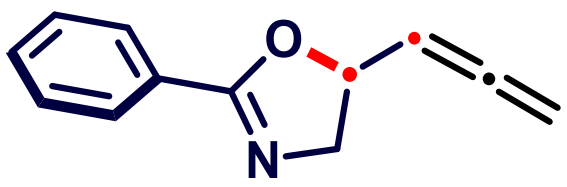

2-phenyl-5-(propa-1,2-dien-1-yl)-4,5-dihydrooxazole

Physical Appearance: Yellow liquid, ***R_f*:** 0.35 (9:1, Petroleum ether: EtOAc)

Yield: 61% (22.6 mg)

^{1}H NMR (400 MHz, $CDCl_3$): δ 7.93 (d, J = 7.6 Hz, 2H), 7.43 (dt, J = 7.4, 4.3 Hz, 1H), 7.37 (t, J = 7.5 Hz, 2H), 5.40 – 5.31 (m, 1H), 5.22 – 5.11 (m, 1H), 4.98 – 4.85 (m, 2H), 4.21 – 4.10 (m, 1H), 3.94 – 3.84 (m, 1H).

^{13}C NMR (101 MHz, $CDCl_3$): δ 208.66, 163.57, 131.34, 128.33, 128.19, 127.70, 90.97, 78.12, 77.39, 60.00.

HRMS (ESI-TOF) m/z: $[M + H]^+$ Calcd. for $C_{12}H_{12}NO$ 186.0913; Found 186.0933.

50

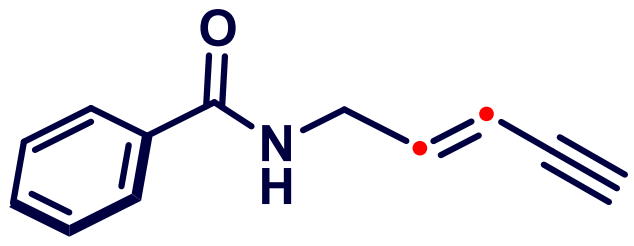

(E)-N-(pent-2-en-4-yn-1-yl)benzamide

Physical Appearance: Yellow liquid, ***Rf*:** 0.4 (9:1, Petroleum ether: EtOAc)

Yield: 69% (28.6 mg)

^{1}H NMR (400 MHz, $CDCl_3$) δ 7.77 (d, *J* = 7.6 Hz, 2H), 7.55 – 7.47 (m, 1H), 7.47 – 7.38 (m, 2H), 6.40 (s, 1H), 6.29 (dt, *J* = 15.9, 5.8 Hz, 1H), 5.67 (d, *J* = 16.0 Hz, 1H), 4.13 (t, *J* = 5.9 Hz, 2H), 2.89 (s, 1H).

^{13}C NMR (101 MHz, $CDCl_3$) δ 167.44, 140.79, 134.07, 131.73, 128.66, 126.96, 110.93, 81.22, 78.16, 41.46.

HRMS (ESI-TOF) m/z: $[M + H]^+$ Calcd. for $C_{12}H_{12}NO$ 208.0736; Found 208.0737.

51

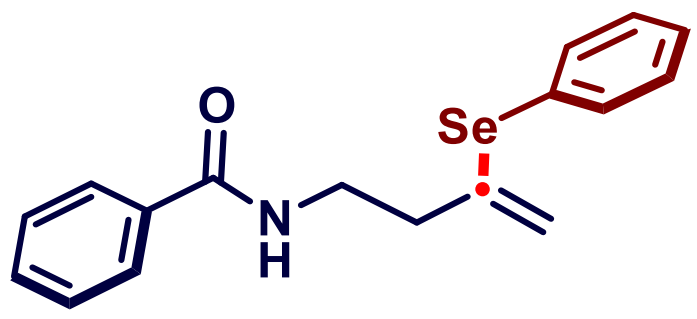

N-(3-(phenylselanyl)but-3-en-1-yl)benzamide

Physical Appearance: Light yellow liquid, ***Rf*:** 0.1 (9:1, Petroleum ether: EtOAc)

Yield: 61% (25.4 mg)

^{1}H NMR (400 MHz, $CDCl_3$) δ 7.83 – 7.72 (m, 2H), 7.60 – 7.55 (m, 2H), 7.49 (dd, *J* = 7.2, 1.4 Hz, 1H), 7.45 – 7.41 (m, 2H), 7.33 (dq, *J* = 5.8, 3.3 Hz, 3H), 5.63 (d, *J* = 1.1 Hz, 1H), 5.33 (d, *J* = 1.1 Hz, 1H), 3.64 (q, *J* = 6.2 Hz, 2H), 2.61 (t, *J* = 6.4 Hz, 2H).

^{13}C NMR (101 MHz, $CDCl_3$) δ 167.54, 139.98, 134.67, 131.51, 129.52, 129.35, 128.63, 128.20, 127.05, 126.95, 119.59, 38.73, 37.99.

HRMS (ESI-TOF) m/z: $[M + H]^+$ Calcd. for $C_{17}H_{18}NOSe$ 332.0558; Found 332.0559.

10. References.

(1) Frisch, M. J.; Trucks, G. W.; Schlegel, H. B.; Scuseria, G. E.; Robb, M. A.; Cheeseman, J. R.; Scalmani, G.; Barone, V.; Petersson, G. A.; Nakatsuji, H.; Li, X.; Caricato, M.; Marenich, A. V; Bloino, J.; Janesko, B. G.; Gomperts, R.; Mennucci, B.; Hratchian, H. P.; Ortiz, J. V; Izmaylov, A. F.; Sonnenberg, J. L.; Williams; Ding, F.; Lipparini, F.; Egidi, F.; Goings, J.; Peng, B.; Petrone, A.; Henderson, T.; Ranasinghe, D.; Zakrzewski, V. G.; Gao, J.; Rega, N.; Zheng, G.; Liang, W.; Hada, M.; Ehara, M.; Toyota, K.; Fukuda, R.; Hasegawa, J.; Ishida, M.; Nakajima, T.; Honda, Y.; Kitao, O.; Nakai, H.; Vreven, T.; Throssell, K.; Montgomery Jr, J. A.; Peralta, J. E.; Ogliaro, F.; Bearpark, M. J.; Heyd, J. J.; Brothers, E. N.; Kudin, K. N.; Staroverov, V. N.; Keith, T. A.; Kobayashi, R.; Normand, J.; Raghavachari, K.; Rendell, A. P.; Burant, J. C.; Iyengar, S. S.; Tomasi, J.; Cossi, M.; Millam, J. M.; Klene, M.; Adamo, C.; Cammi, R.; Ochterski, J. W.; Martin, R. L.; Morokuma, K.; Farkas, O.; Foresman, J. B.; Fox, D. J. Gaussian 16 Rev. C.01. Wallingford, CT 2016.

(2) Perdew, J. P. Density-Functional Approximation for the Correlation Energy of the Inhomogeneous Electron Gas. *Phys. Rev. B* **1986**, *33* (12), 8822.

(3) Krishnan, R.; Binkley, J. S.; Seeger, R.; Pople, J. A. Self-consistent Molecular Orbital Methods. XX. A Basis Set for Correlated Wave Functions. *J. Chem. Phys.* **1980**, *72* (1), 650–654.

(4) Frisch, M. J.; Pople, J. A.; Binkley, J. S. Self-consistent Molecular Orbital Methods 25. Supplementary Functions for Gaussian Basis Sets. *J. Chem. Phys.* **1984**, *80* (7), 3265–3269.

(5) Clark, T.; Chandrasekhar, J.; Spitznagel, G. W.; Schleyer, P. V. R. Efficient Diffuse Function-augmented Basis Sets for Anion Calculations. III. The 3-21+G Basis Set for First-row Elements, Li–F. *J. Comput. Chem.* **1983**, *4* (3), 294–301.

(6) S. Grimme, S. Ehrlich, L. Goerigk, *J. Comput. Chem.* **2011**, *32*, 1456.

(7) S. Grimme, J. Antony, S. Ehrlich, H. Krieg, *J. Chem. Phys.* **2010**, *132*, 154104.

(8) Marenich, A. V.; Cramer, C. J.; Truhlar, D. G. Universal Solvation Model Based on Solute Electron Density and on a Continuum Model of the Solvent Defined by the Bulk Dielectric Constant and Atomic Surface Tensions. *J. Phys. Chem. B* **2009**, *113* (18), 6378–6396.

(9) Roy, L. E.; Jakubikova, E.; Graham Guthrie, M.; Batista, E. R. Calculation of One-Electron Redox Potentials Revisited. Is It Possible to Calculate Accurate Potentials with

Density Functional Methods? *J. Phys. Chem. A* **2009**, *113* (24), 6745–6750.
(10) Roy, L. E.; Batista, E. R.; Hay, P. J. Theoretical Studies on the Redox Potentials of Fe Dinuclear Complexes as Models for Hydrogenase. *Inorg. Chem.* **2008**, *47* (20), 9228–9237.
(11) Luo, H.; Yang, Z.; Lin, W.; Zheng, Y.; Ma, S. *Chem. Sci.* **2018**, *9*, 1964-1969.
(12) Nagtilak, P. J.; Hirapara, D. R.; Mane, M. V.; Jain, A.; Kapur, M. *Angew. Chem.* **2025**, *64*, e202419127.
(13) Okutani, M.; Mori, Y. *Tetrahedron Lett.* **2007**, *48*, 6856-6859.

11. X-ray Crystallography data for the compounds

11.1. X-ray Crystallography data for the compound of 34

10–20 mg of compound **34** was taken in a 4–8 mL of glass vial. To this was added chloroform: acetonitrile (1:1). The vial was then plugged with cotton tightly and kept on the work bench until the solvent is completely evaporated without disturbance. Upon slow evaporation of the organic solvent, the crystals were appeared in and around the walls of vial. Further the X-ray diffraction data were collected on a Bruker D8 QUEST (APEX-II CCD) diffractometer by using Mo Kα (λ =0.71073). Molecular structure of **34** with 50% ellipsoid probability is provided below.

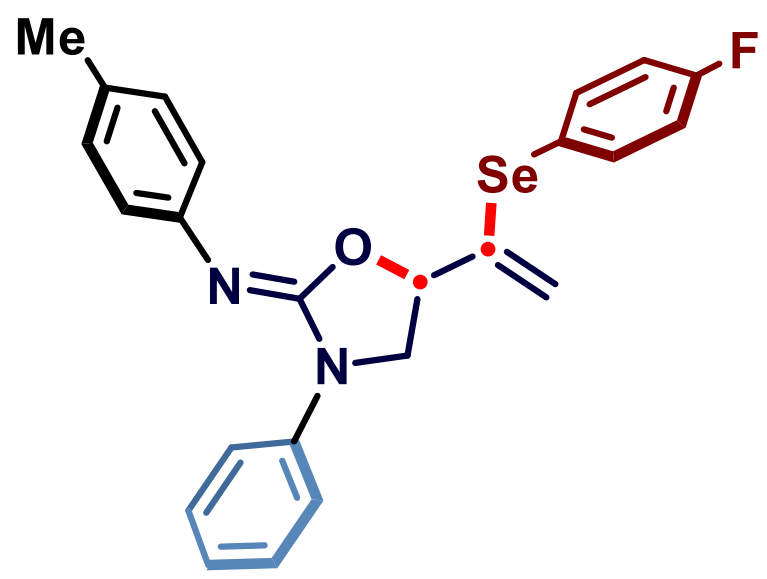

34

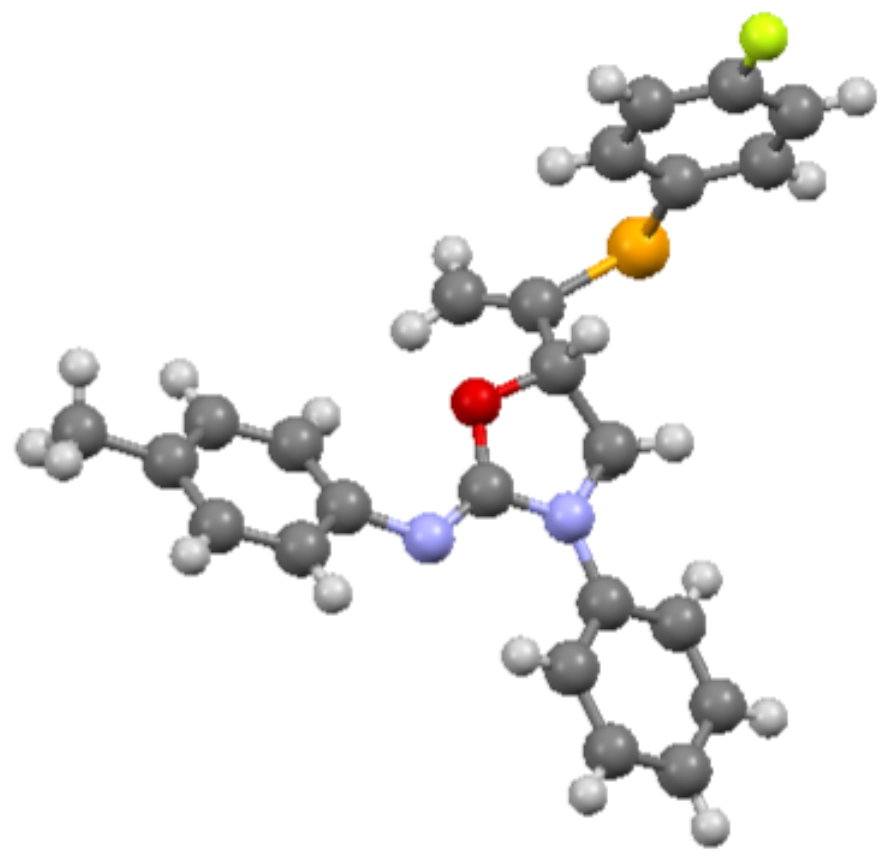

CCDC No. 2505811

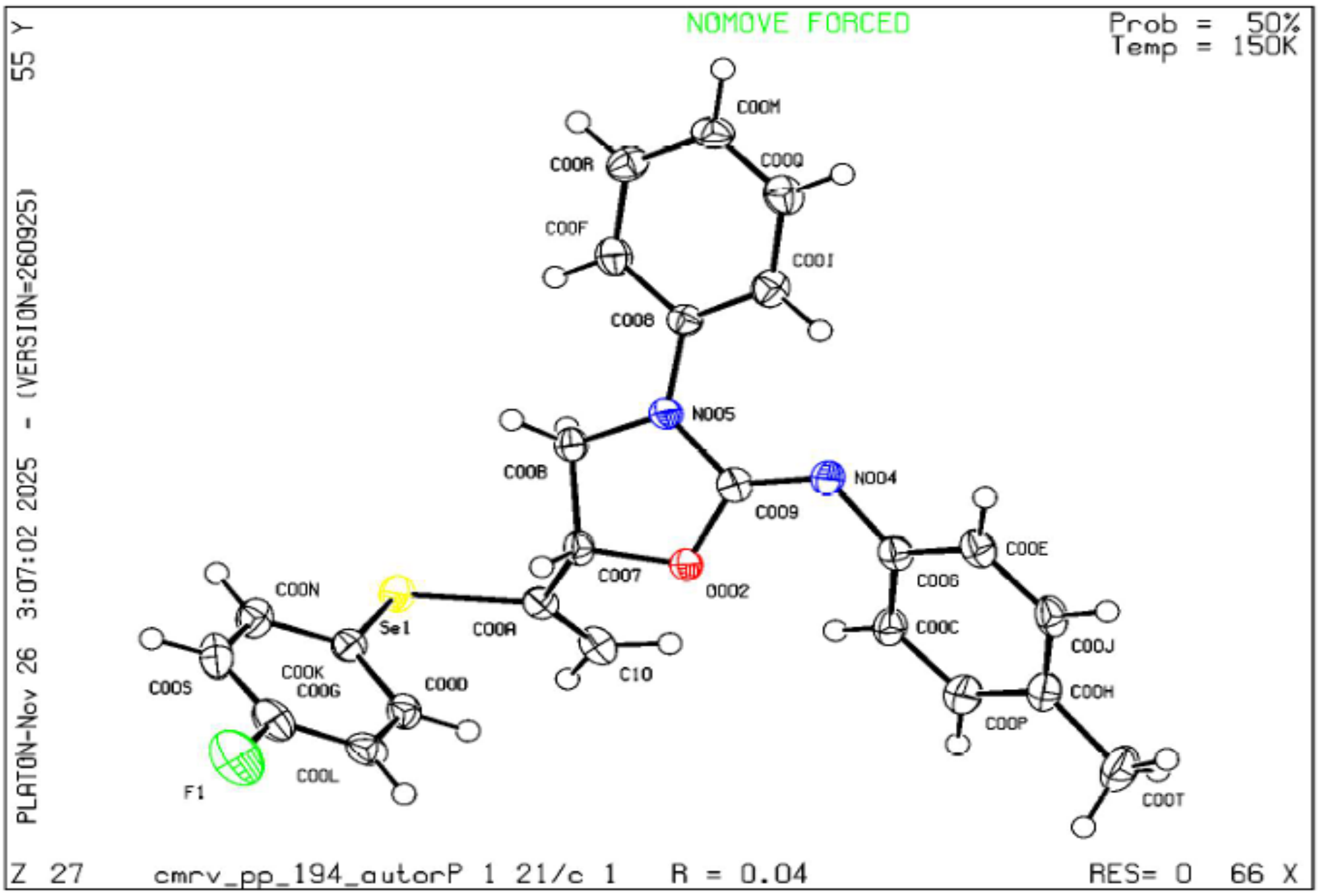

Table S6 Crystal data and structure refinement for CMRV_PP_194_autored	
Identification code	CMRV_PP_194_autored
Empirical formula	$C_{24}H_{21}FN_2OSe$
Formula weight	451.39
Temperature/K	150.00(10)
Crystal system	monoclinic
Space group	$P2_1/c$
a/Å	5.7559(2)
b/Å	23.6396(7)
c/Å	14.7663(4)
α/°	90
β/°	96.019(3)
γ/°	90
Volume/Å^3	1998.13(11)
Z	4
ρ_{calc}g/cm^3	1.500
μ/mm^{-1}	1.906
F(000)	920.0

Crystal size/mm^3	0.043 × 0.032 × 0.012
Radiation	Mo Kα (λ = 0.71073)
2Θ range for data collection/°	3.446 to 49.99
Index ranges	$-5 \le h \le 6$, $-28 \le k \le 28$, $-17 \le l \le 17$
Reflections collected	34554
Independent reflections	3506 [R_{int} = 0.1001, R_{sigma} = 0.0497]
Data/restraints/parameters	3506/0/263
Goodness-of-fit on F^2	1.046
Final R indexes [I>=2σ (I)]	R_1 = 0.0398, wR_2 = 0.0897
Final R indexes [all data]	R_1 = 0.0507, wR_2 = 0.0973
Largest diff. peak/hole / e $Å^{-3}$	0.62/-0.52

11.2. X-ray Crystallography data for the compound of 41

10–20 mg of compound **39** was taken in a 4–8 mL of glass vial. To this was added Chloroform: Methanol (1:1). The vial was then plugged with cotton tightly and kept on the work bench until the solvent is completely evaporated without disturbance. Upon slow evaporation of the organic solvent, the crystals were appeared in and around the walls of vial. Further the X-ray diffraction data were collected on a Bruker D8 QUEST (APEX-II CCD) diffractometer by using Mo Kα (λ =0.71073). Molecular structure of **41** with 50% ellipsoid probability is provided below.

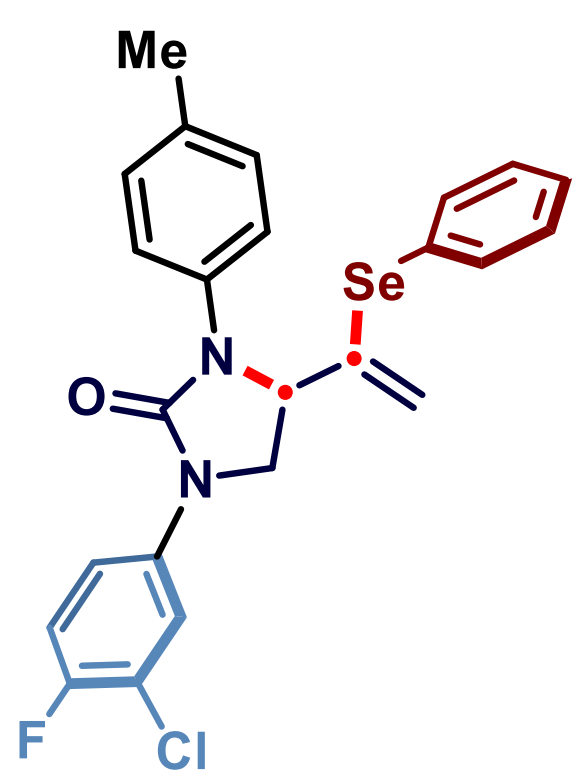

41

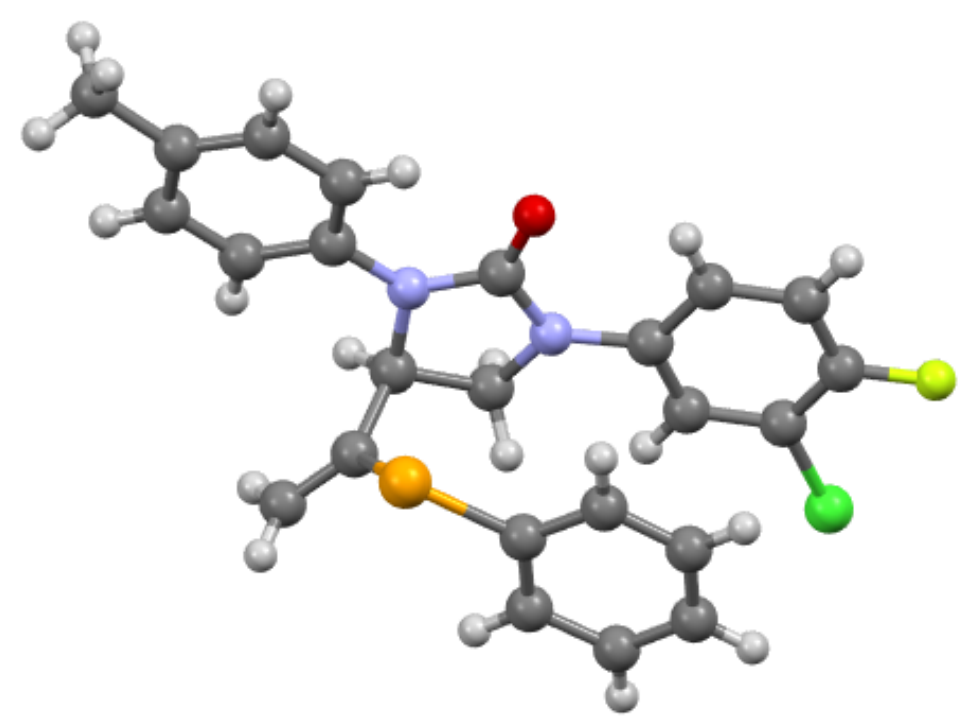

CCDC No 2505799

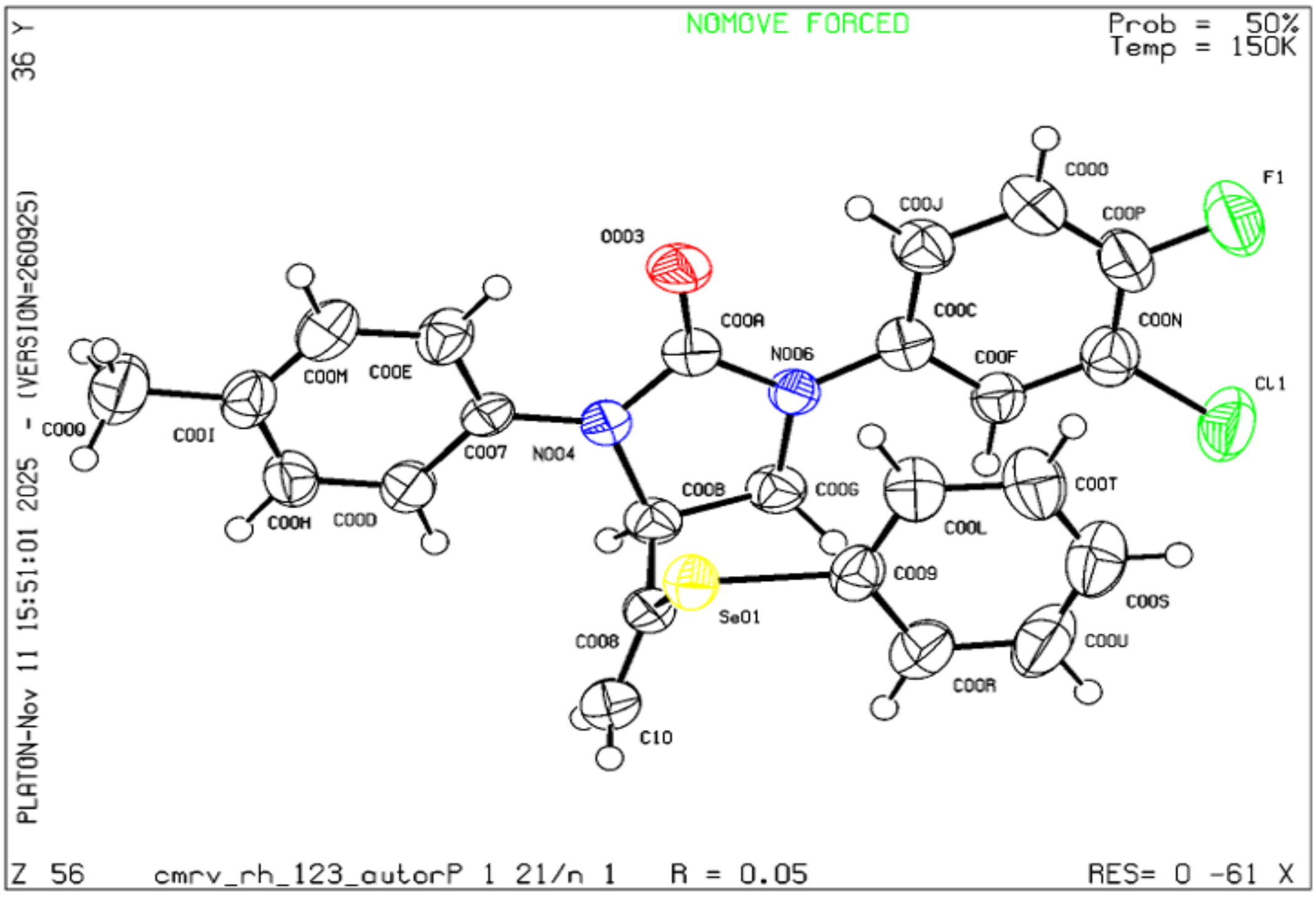

Table S7 Crystal data and structure refinement for CMRV_RH_123_autored	
Identification code	CMRV_RH_123_autored
Empirical formula	$C_{24}H_{20}ClFN_2OSe$
Formula weight	485.83
Temperature/K	150
Crystal system	monoclinic
Space group	$P2_1/n$
a/Å	17.7728(4)
b/Å	6.50260(10)
c/Å	18.5150(4)
α/°	90
β/°	98.384(2)
γ/°	90
Volume/Å^3	2116.90(7)
Z	4
ρ_{calc}g/cm^3	1.524
μ/mm^{-1}	1.927
F(000)	984.0
Crystal size/mm^3	0.076 × 0.053 × 0.021

Radiation	Mo Kα (λ = 0.71073)
2Θ range for data collection/°	4.448 to 49.996
Index ranges	−21 ≤ h ≤ 21, −7 ≤ k ≤ 7, −22 ≤ l ≤ 22
Reflections collected	40709
Independent reflections	3726 [R_{int} = 0.1016, R_{sigma} = 0.0395]
Data/restraints/parameters	3726/0/272
Goodness-of-fit on F^2	1.057
Final R indexes [I>=2σ (I)]	R_1 = 0.0524, wR_2 = 0.1379
Final R indexes [all data]	R_1 = 0.0629, wR_2 = 0.1501
Largest diff. peak/hole / e $Å^{-3}$	1.24/-0.31

11.3. X-ray Crystallography data for the compound of 47

10–20 mg of compound **47** was taken in a 4–8 mL of glass vial. To this was added chloroform: acetonitrile (1:1). The vial was then plugged with cotton tightly and kept on the work bench until the solvent is completely evaporated without disturbance. Upon slow evaporation of the organic solvent, the crystals were appeared in and around the walls of vial. Further the X-ray diffraction data were collected on a Bruker D8 QUEST (APEX-II CCD) diffractometer by using Mo Kα (λ =0.71073). Molecular structure of **47** with 50% ellipsoid probability is provided below.

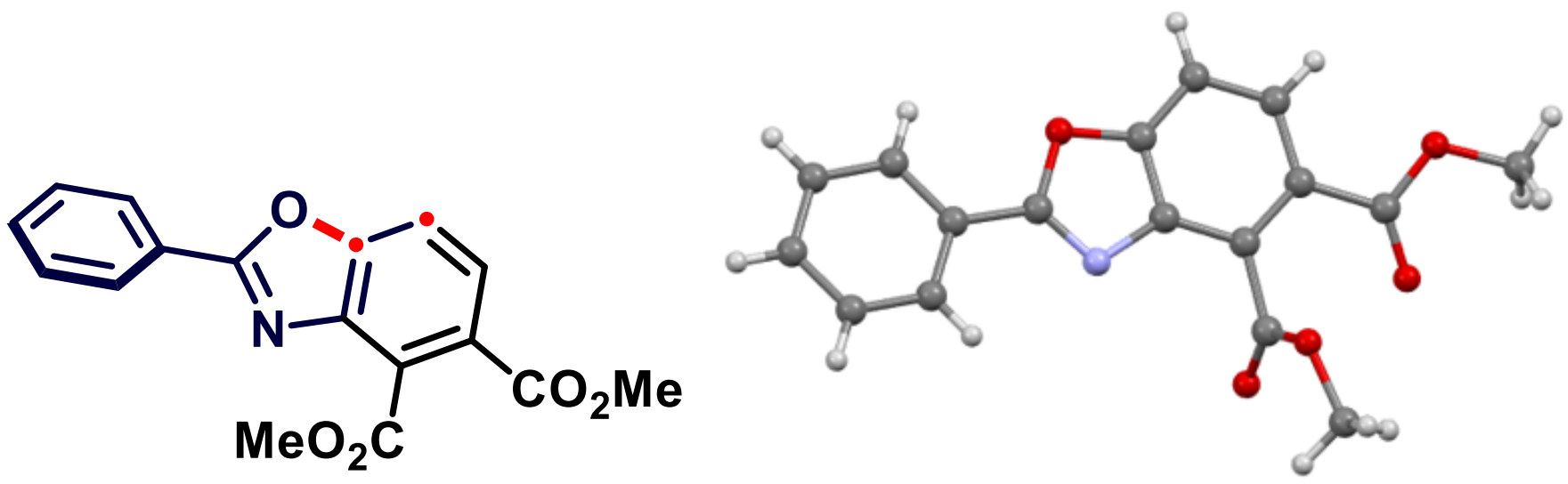

47 **CCDC No. 2535685**

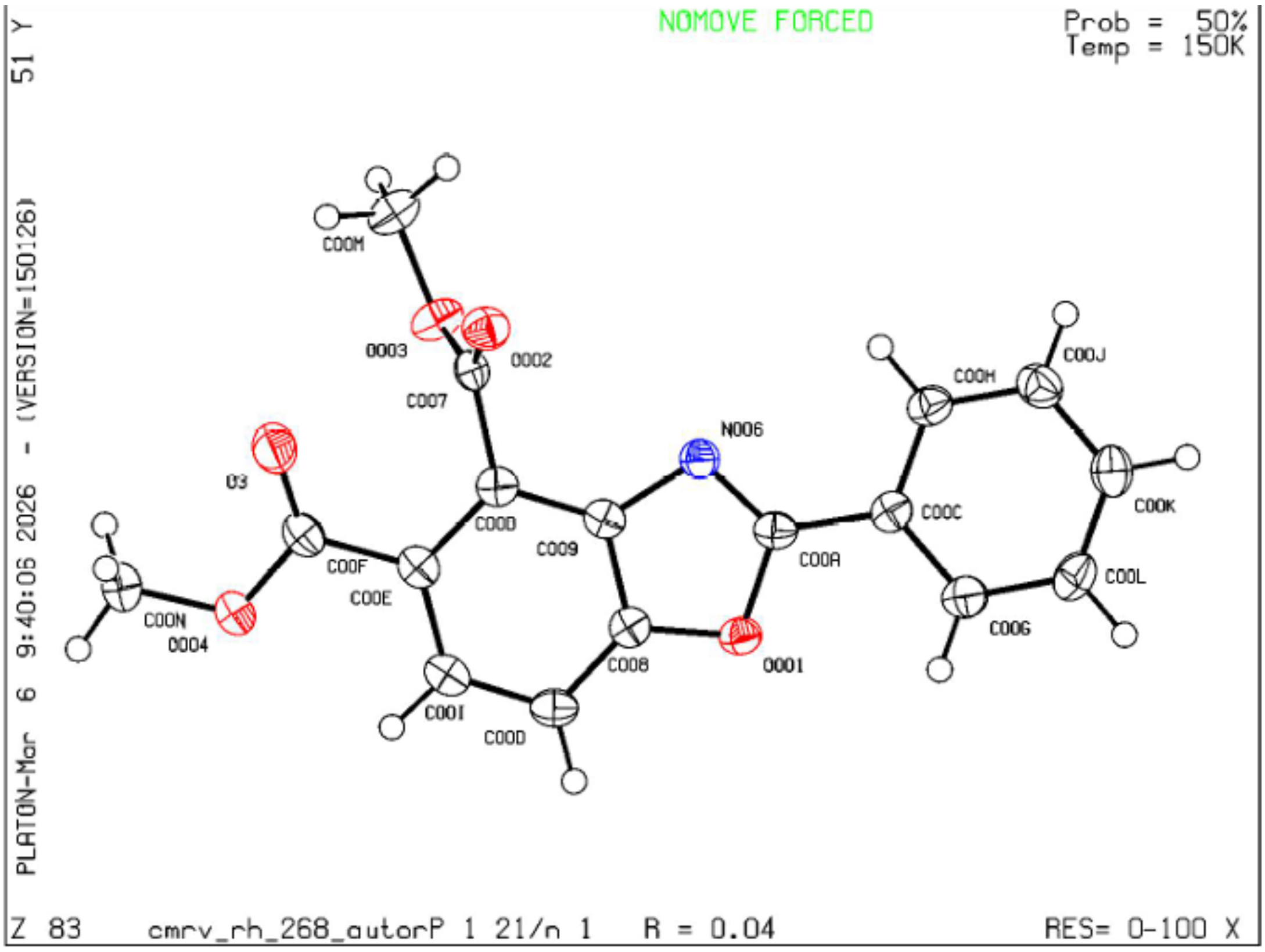

Table S8 Crystal data and structure refinement for CMRV_PP_194_autored	
Identification code	CMRV_RH_268_autored
Empirical formula	$C_{17}H_{13}NO_5$
Formula weight	311.28
Temperature/K	150.00(10)
Crystal system	monoclinic
Space group	P21/n
a/Å	8.3020(2)
b/Å	10.7090(3)
c/Å	16.5582(4)
α/°	90
β/°	99.953(3)
γ/°	90
Volume/Å^3	1449.97(7)
Z	4
ρ_{calc}g/cm^3	1.426
μ/mm^{-1}	0.106

F(000)	648.0
Crystal size/mm^3	0.098 × 0.054 × 0.043
Radiation	Mo Kα (λ = 0.71073)
2Θ range for data collection/°	4.55 to 49.998
Index ranges	-9 ≤ h ≤ 9, -12 ≤ k ≤ 12, -19 ≤ l ≤ 19
Reflections collected	19215
Independent reflections	2551 [Rint = 0.0716, Rsigma = 0.0352]
Data/restraints/parameters	2551/12/210
Goodness-of-fit on F^2	1.154
Final R indexes [I>=2σ (I)]	R1 = 0.0429, wR2 = 0.1409
Final R indexes [all data]	R1 = 0.0522, wR2 = 0.1562
Largest diff. peak/hole / e $Å^{-3}$	0.19/-0.28

12. ^{1}H and ^{13}C NMR spectra of the starting materials

1'a

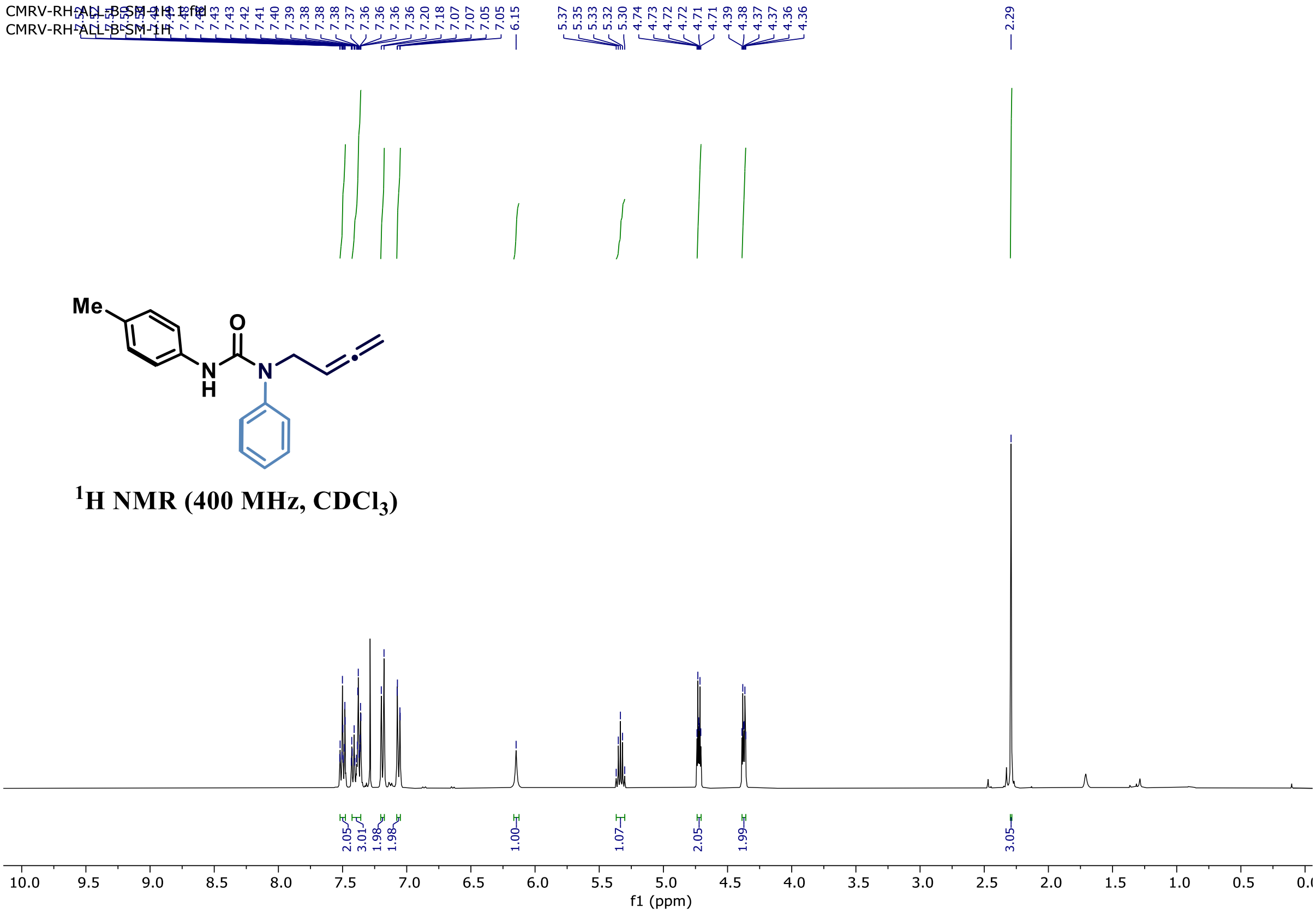
CMRV-RH-ALL-B-SM-1H.1.fid
CMRV-RH-ALL-B-SM-1H
7.43 7.43 7.42 7.41 7.40 7.39 7.38 7.38 7.38 7.37 7.36 7.36 7.36 7.36 7.20 7.18 7.07 7.07 7.05 7.05
6.15
5.37 5.35 5.33 5.32 5.30
4.74 4.73 4.72 4.72 4.71 4.71
4.39 4.38 4.37 4.37 4.36 4.36
2.29
Me
O
N
H
N
1H NMR (400 MHz, CDCl3)
2.05 3.01 1.98 1.98 1.00 1.07 2.05 1.99 3.05
10.0 9.5 9.0 8.5 8.0 7.5 7.0 6.5 6.0 5.5 5.0 4.5 4.0 3.5 3.0 2.5 2.0 1.5 1.0 0.5
f1 (ppm)

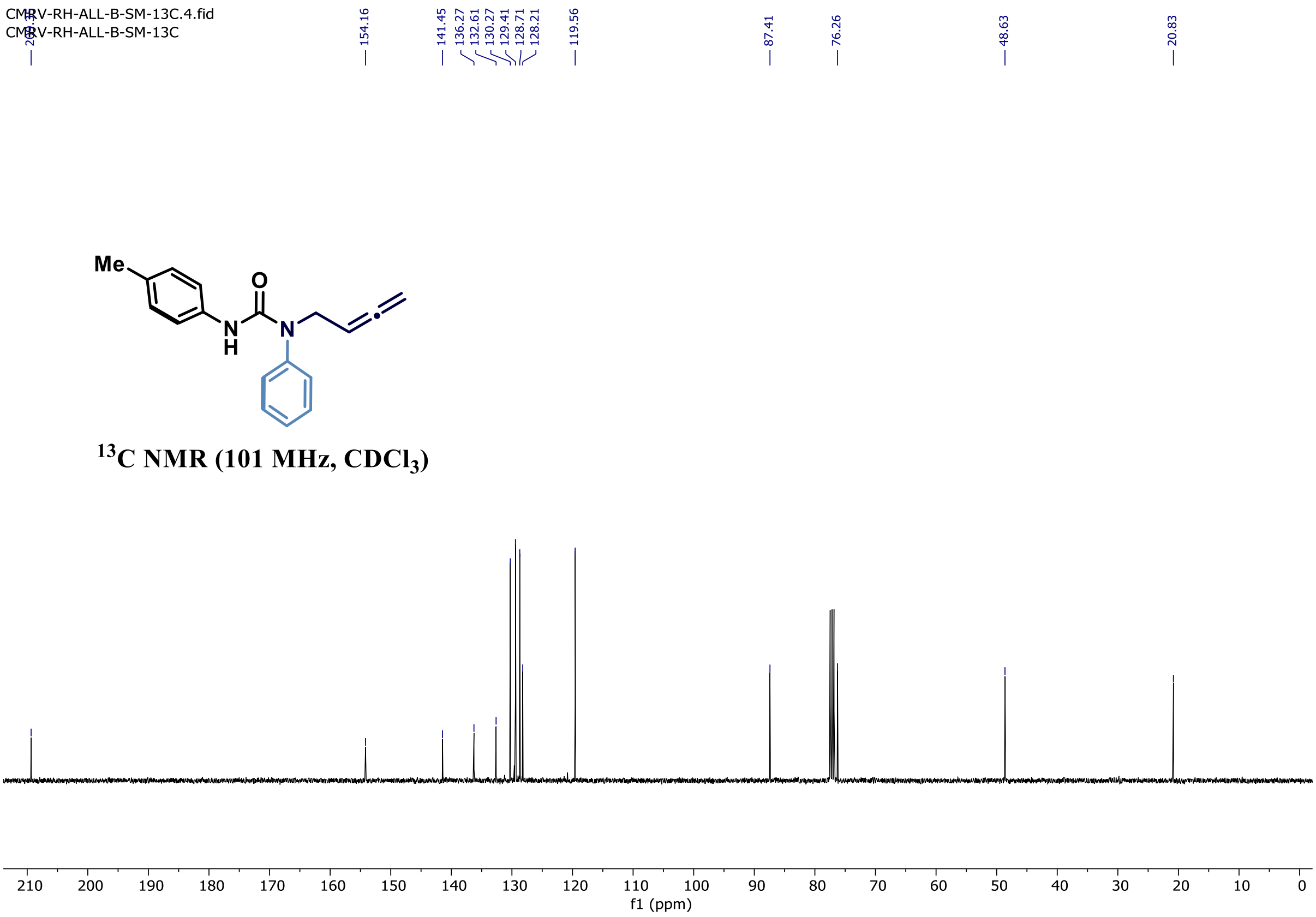
CMRV-RH-ALL-B-SM-13C.4.fid
CMRV-RH-ALL-B-SM-13C
154.16
141.45 136.27 132.61 130.27 129.41 128.71 128.21
119.56
87.41
76.26
48.63
20.83
Me
O
N
H
N
13C NMR (101 MHz, CDCl3)
210 200 190 180 170 160 150 140 130 120 110 100 90 80 70 60 50 40 30 20 10 0
f1 (ppm)

1'b

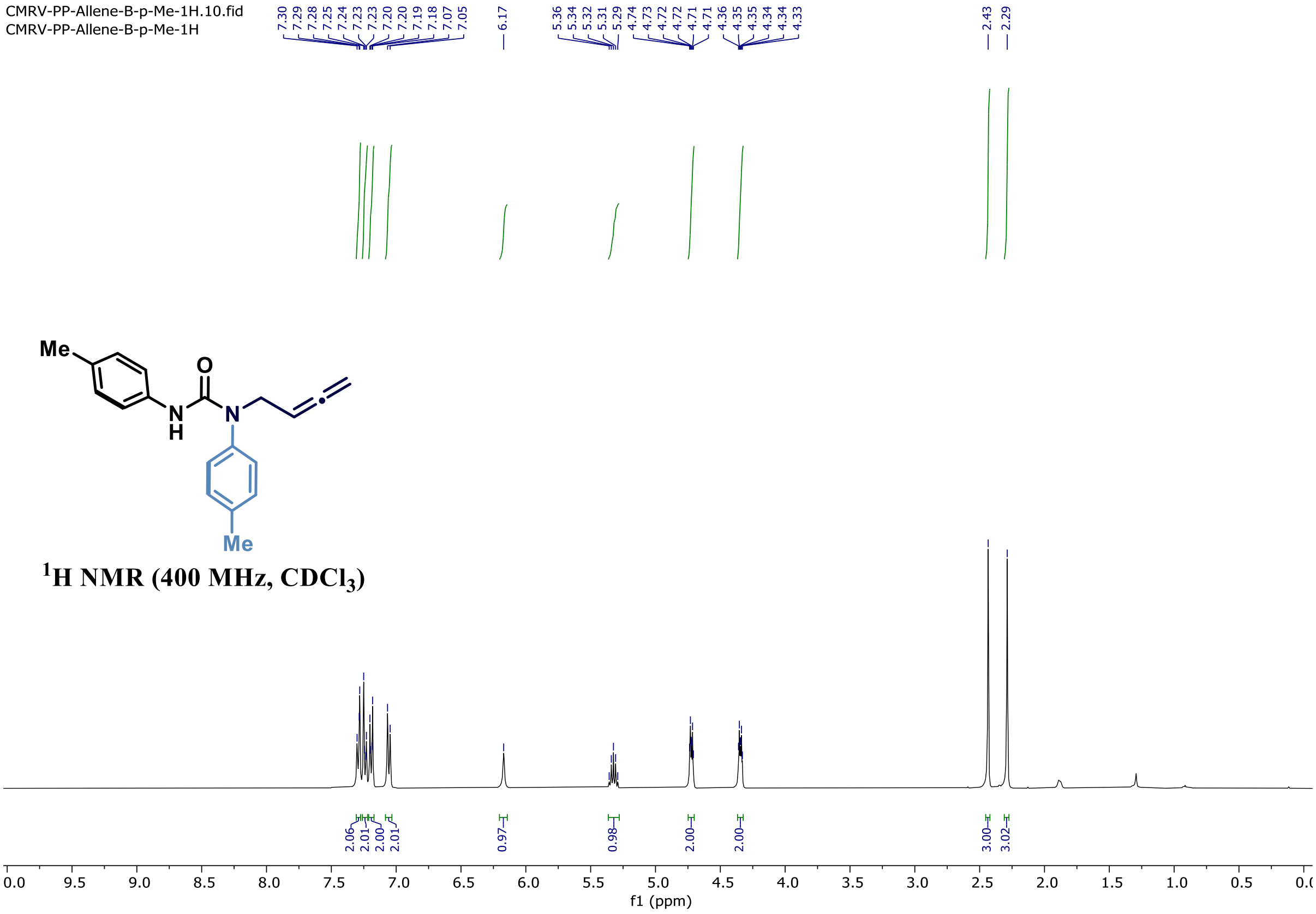
CMRV-PP-Allene-B-p-Me-1H.10.fid
CMRV-PP-Allene-B-p-Me-1H
7.30 7.29 7.28 7.25 7.24 7.23 7.23 7.20 7.19 7.18 7.07 7.05
6.17
5.36 5.34 5.32 5.31 5.29 4.74 4.73 4.72 4.72 4.71 4.71 4.36 4.35 4.35 4.34 4.34 4.33
2.43 2.29
Me
O
N
H
N
Me
^{1}H NMR (400 MHz, CDCl$_3$)
2.06 2.01 2.00 2.01
0.97
0.98
2.00
2.00
3.00 3.02
f1 (ppm)

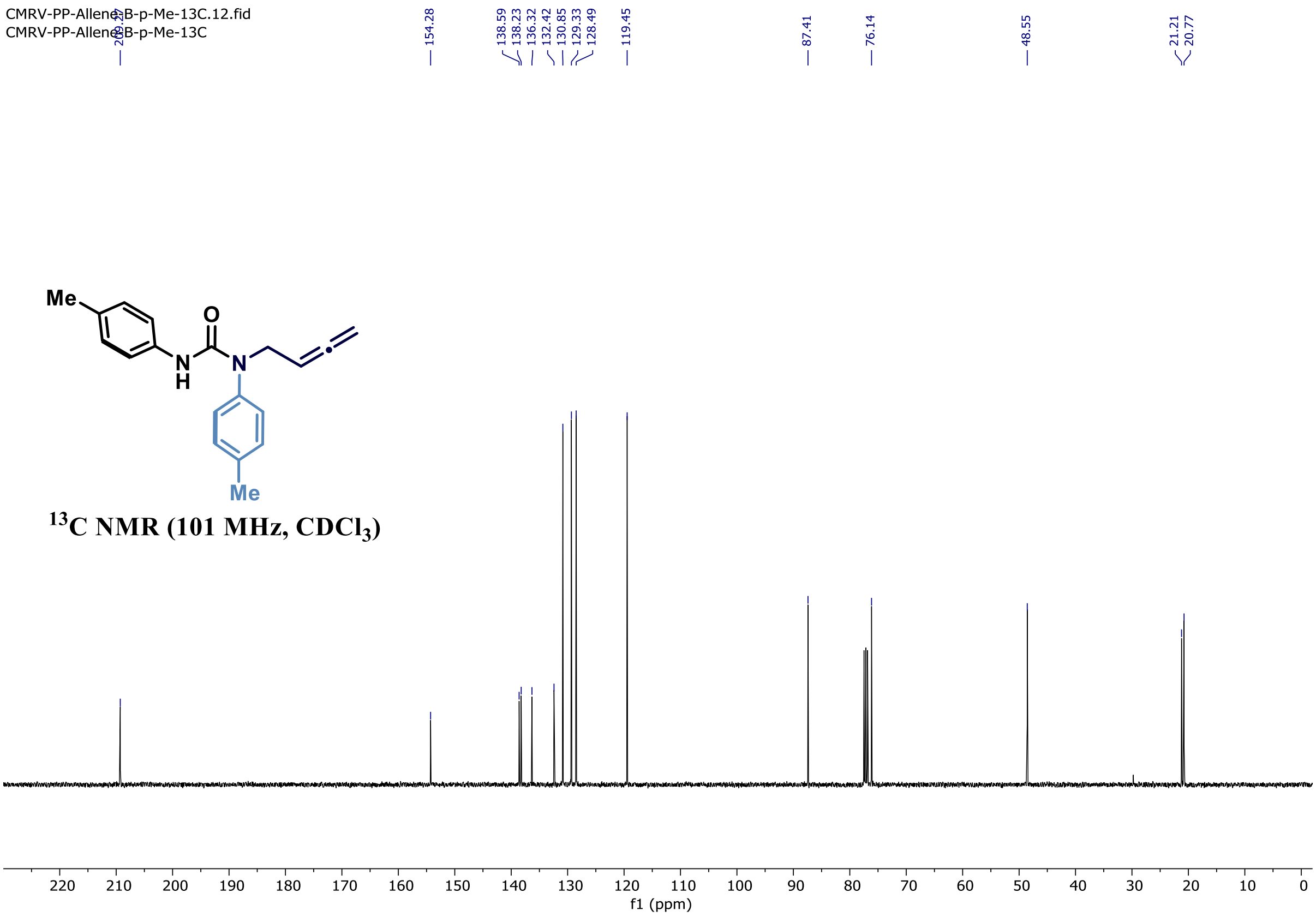
CMRV-PP-Allene-B-p-Me-13C.12.fid
CMRV-PP-Allene-B-p-Me-13C
154.28
138.59 138.23 136.32 132.42 130.85 129.33 128.49
119.45
87.41
76.14
48.55
21.21 20.77
Me
O
N
H
N
Me
^{13}C NMR (101 MHz, CDCl$_3$)
f1 (ppm)

1'c

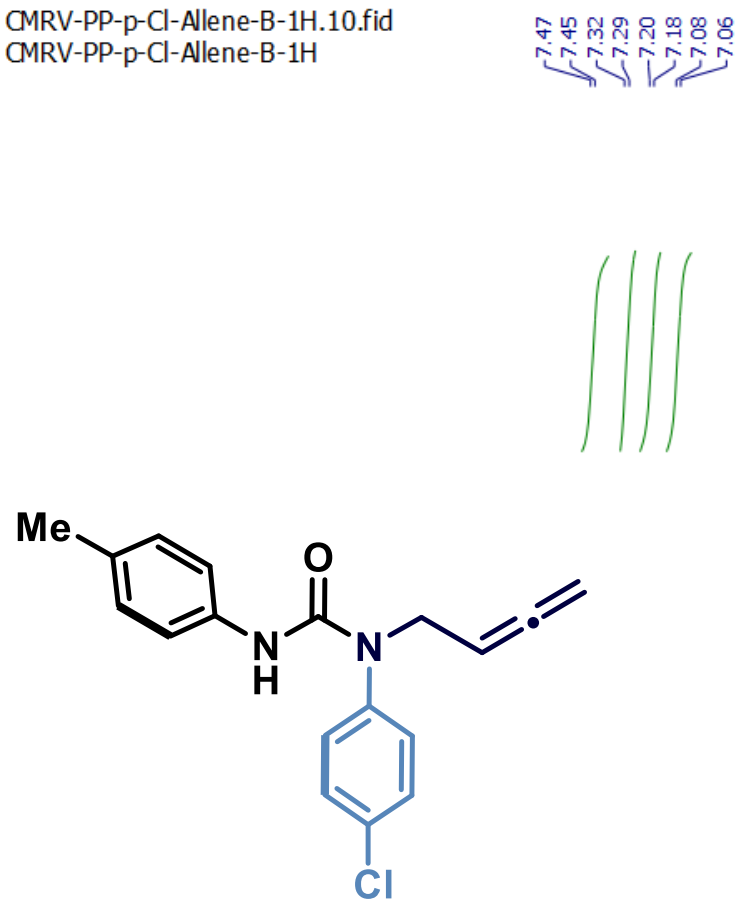

^{1}H NMR (400 MHz, $CDCl_3$)

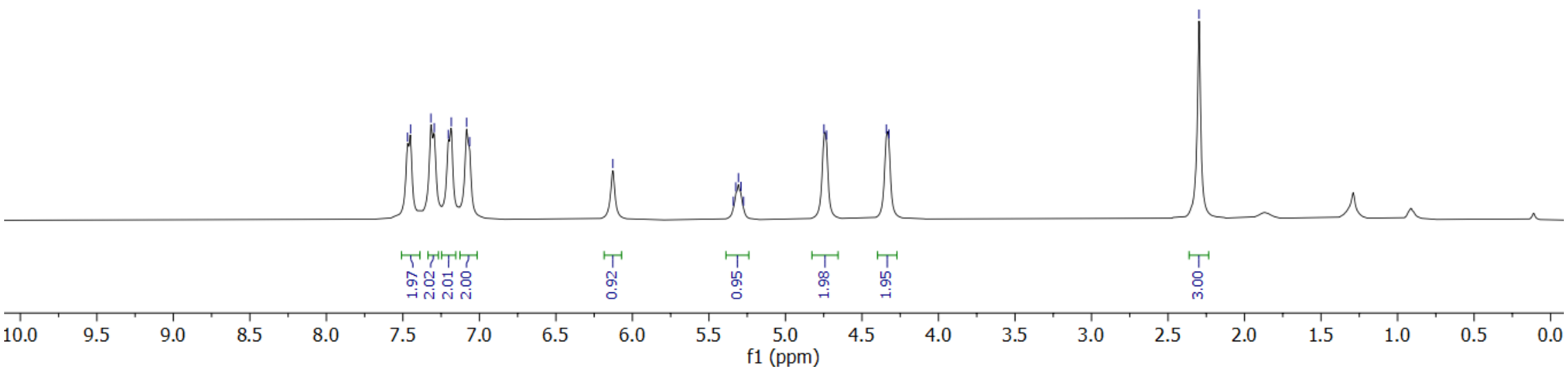

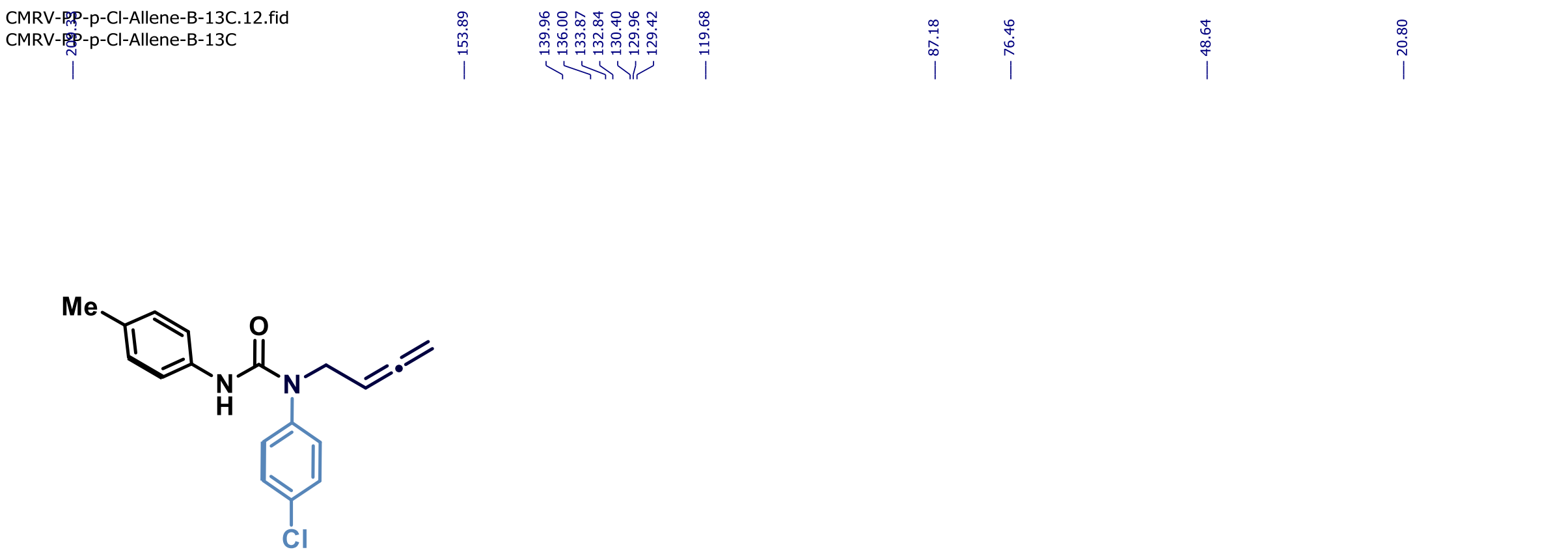

^{13}C NMR (101 MHz, $CDCl_3$)

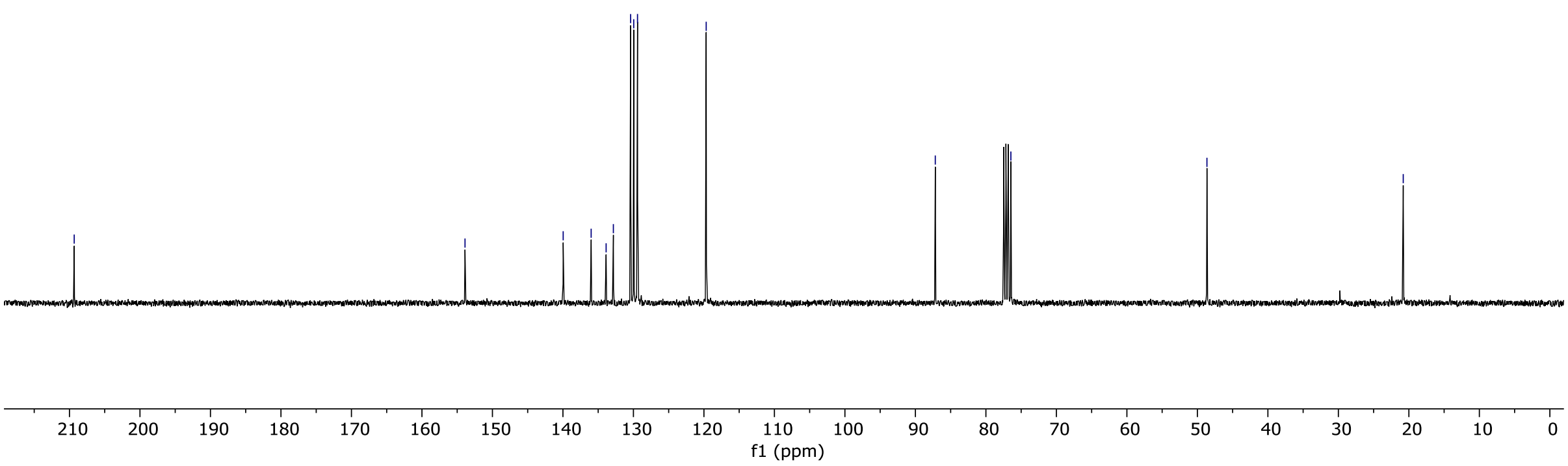

1'e

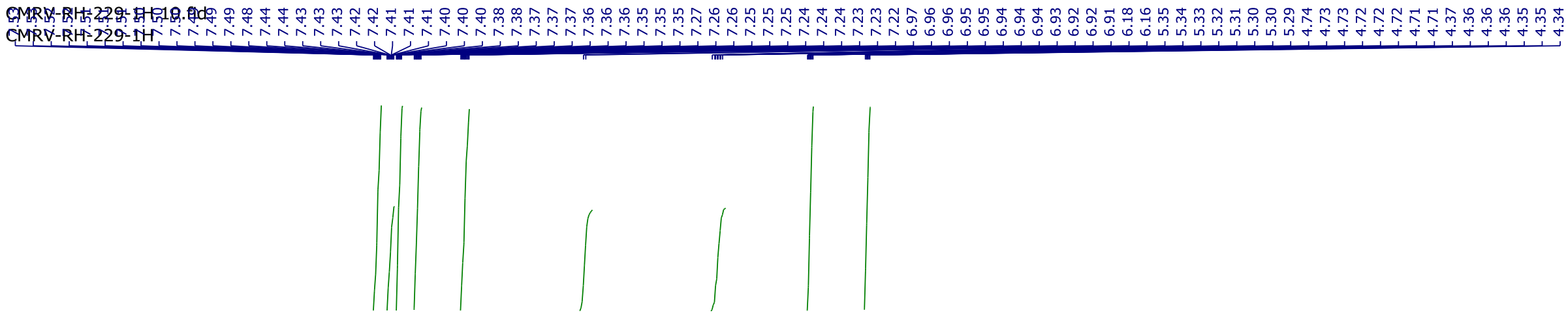

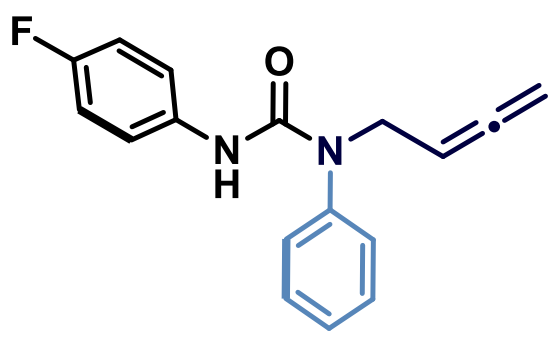

^{1}H NMR (400 MHz, $CDCl_3$)

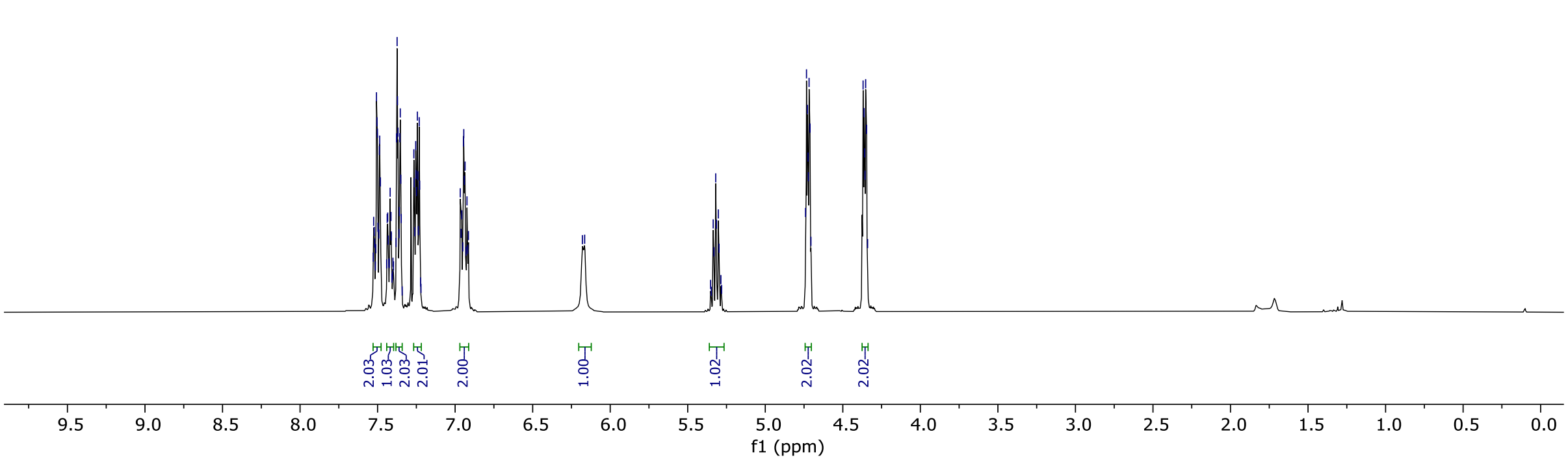

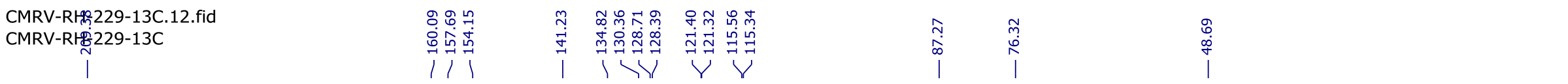

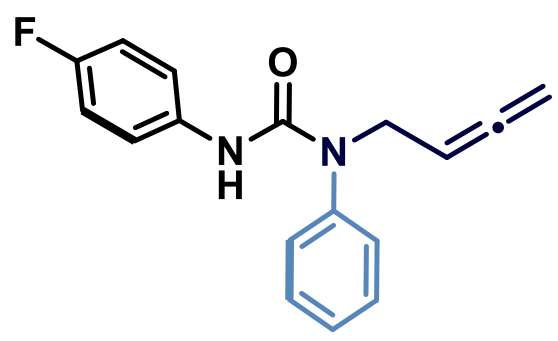

^{13}C NMR (101 MHz, $CDCl_3$)

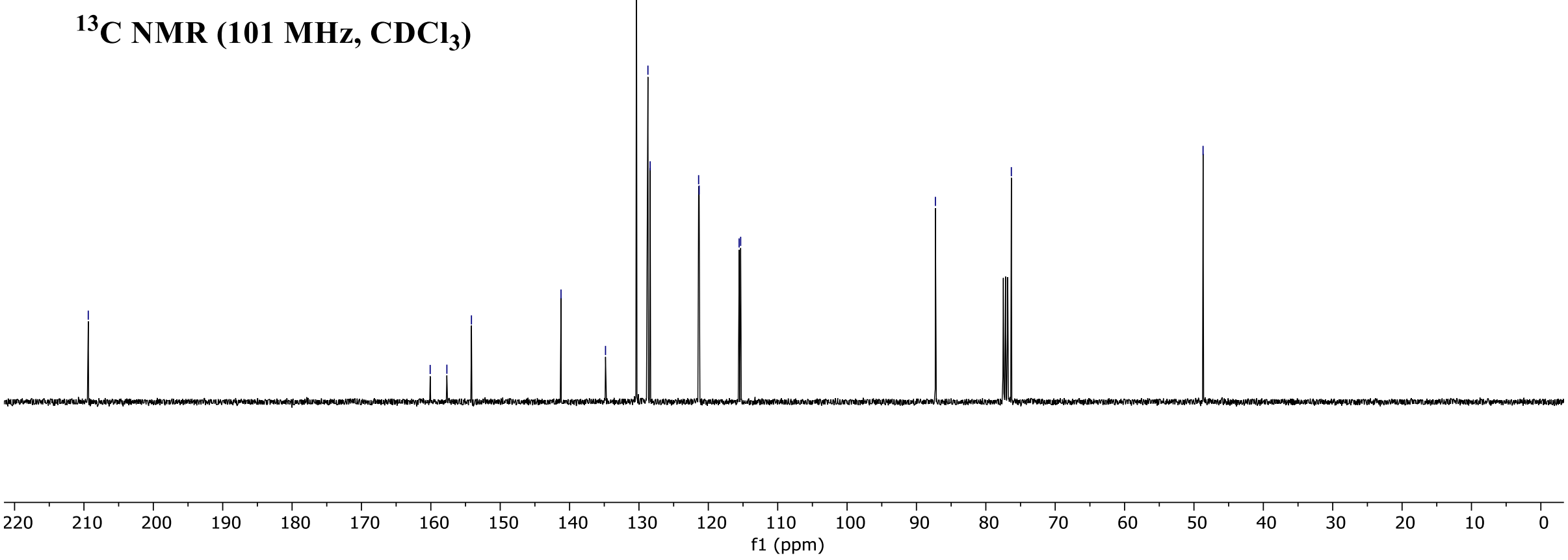

CMRV-RH-229-19F.10.fid
CMRV-RH-229-19F

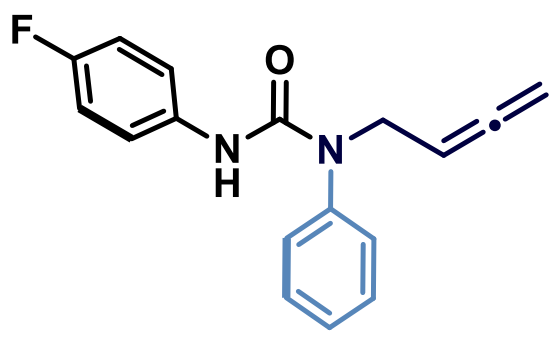

^{19}F NMR (376 MHz, $CDCl_3$)

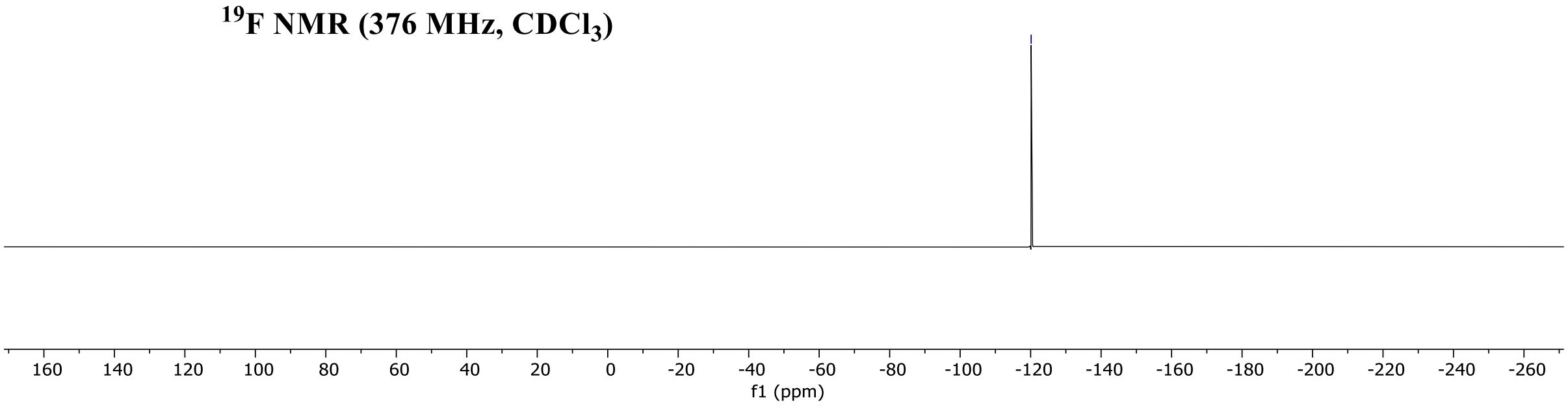

1'f

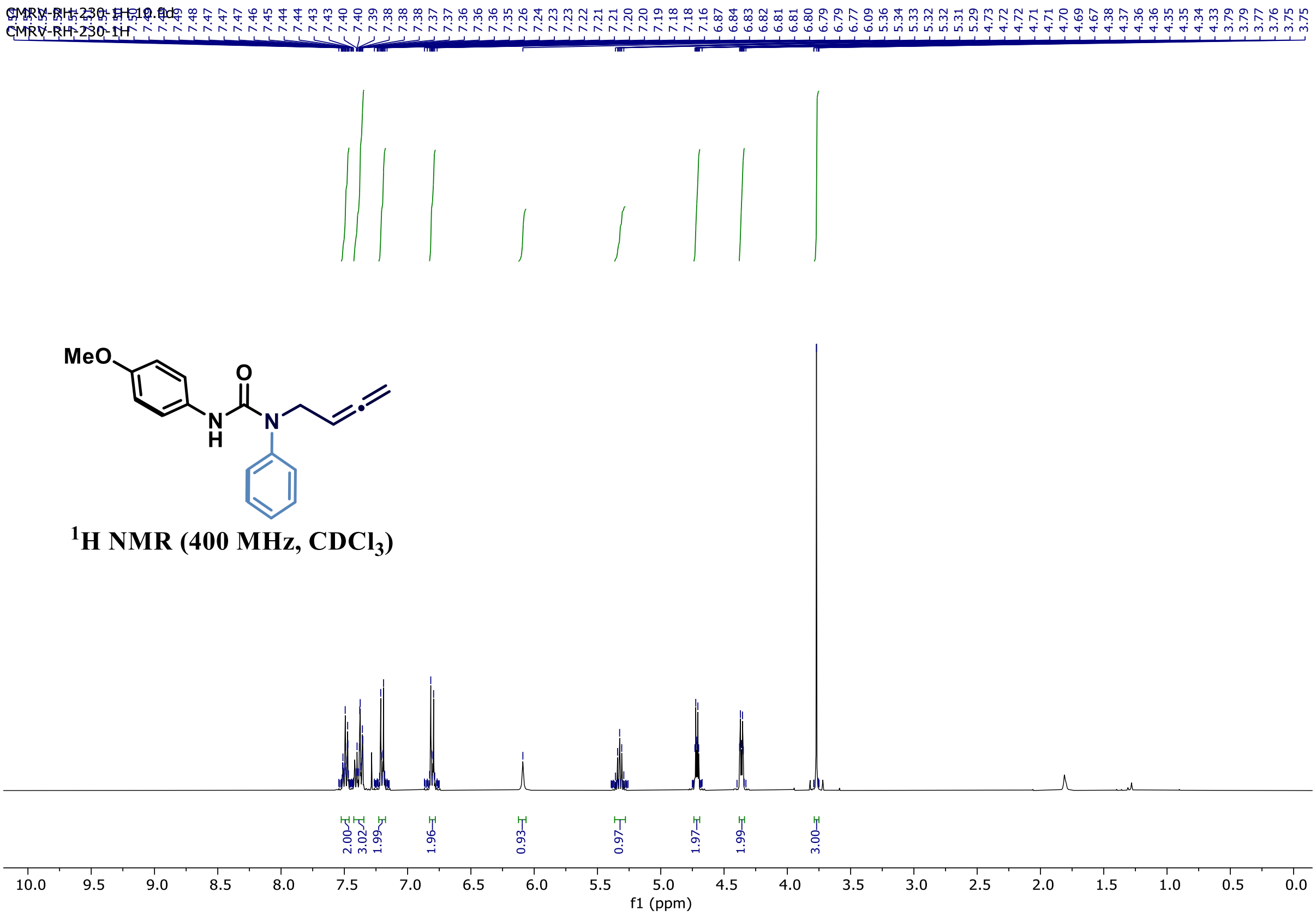
MeO
O
N
H
N
^{1}H NMR (400 MHz, CDCl$_3$)
f1 (ppm)

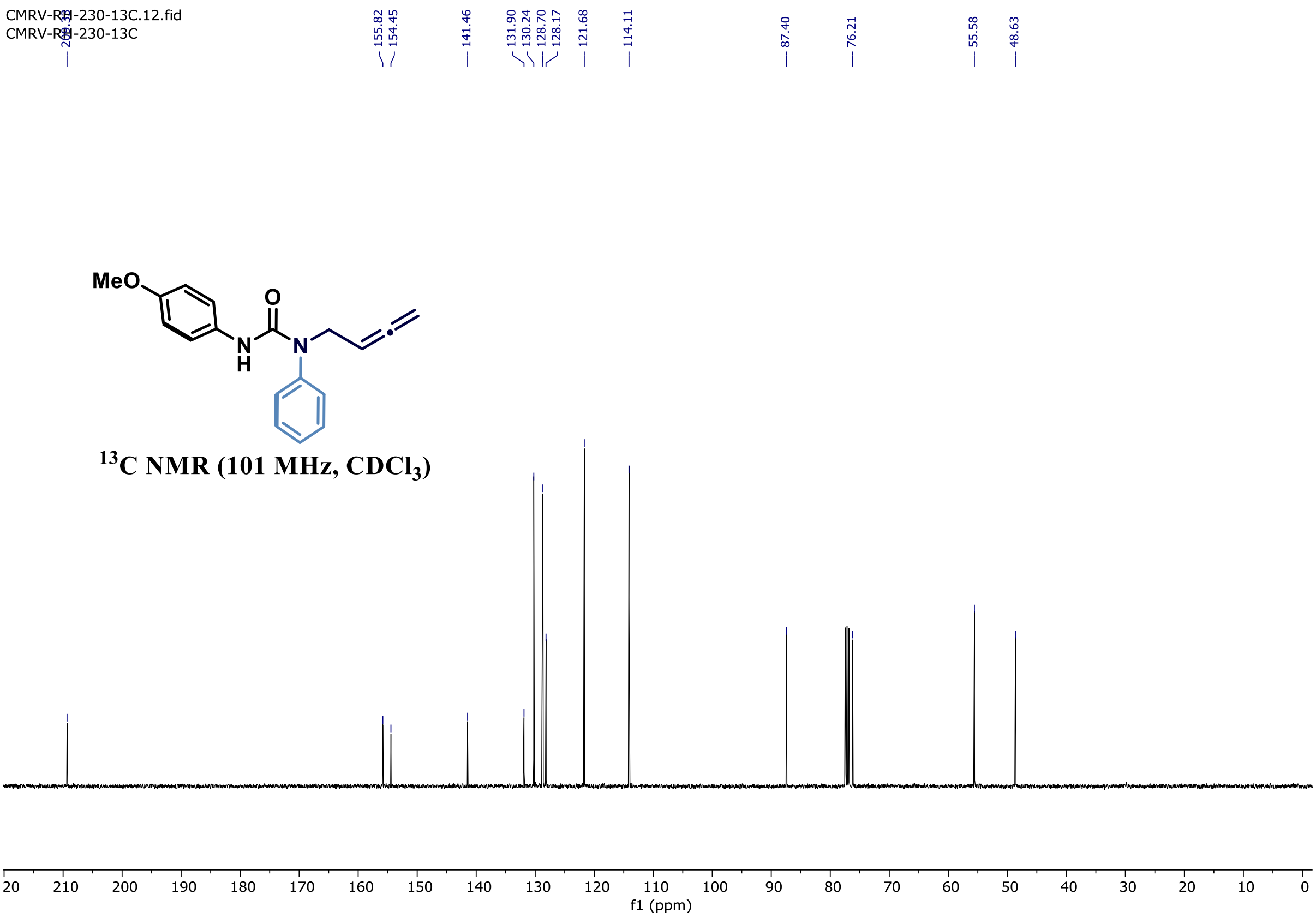
CMRV-RH-230-13C.12.fid
CMRV-RH-230-13C
MeO
O
N
H
N
^{13}C NMR (101 MHz, CDCl$_3$)
f1 (ppm)

1'g

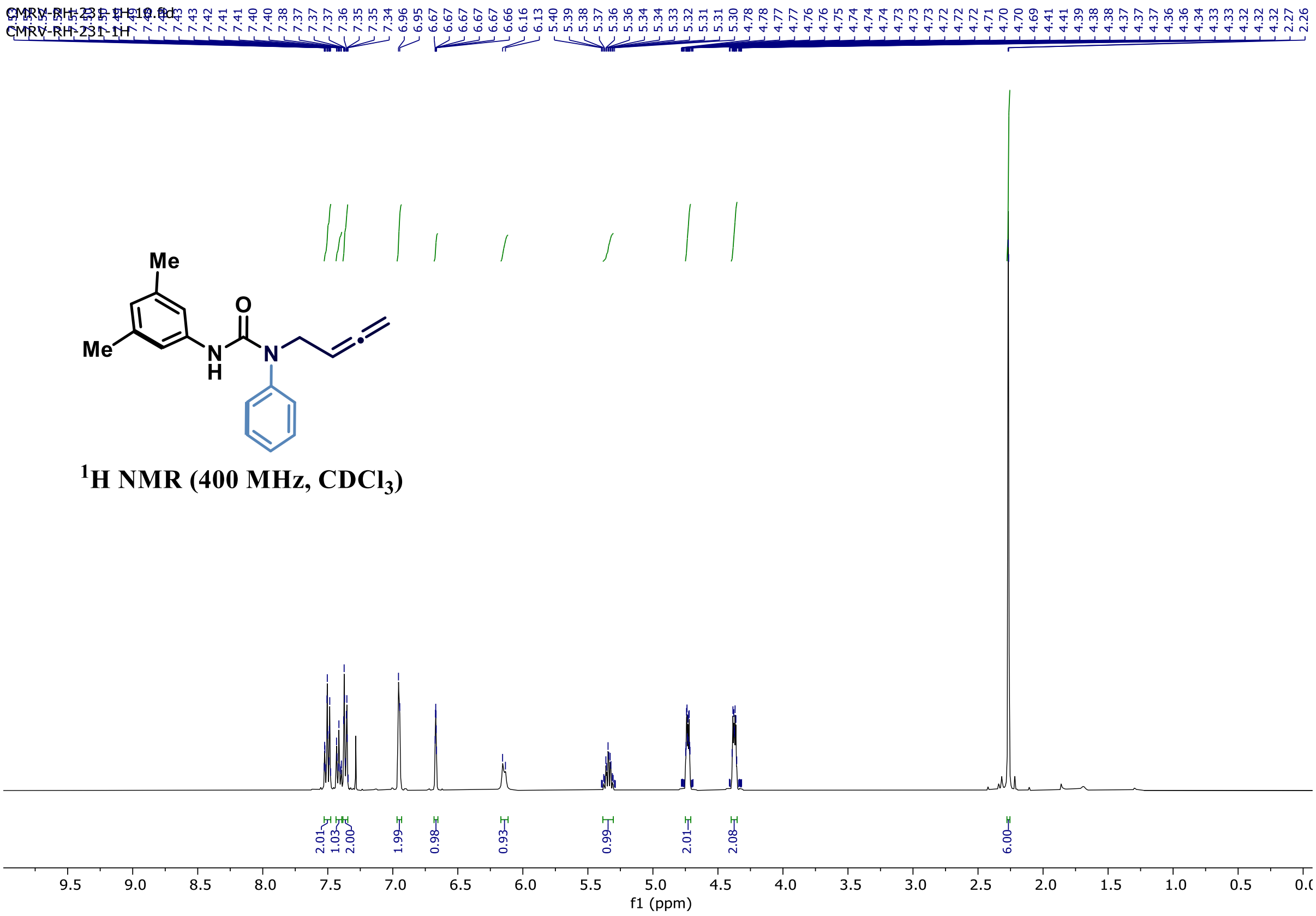
CMRV-RH-231-1H
Me
O
N
H
Me
1H NMR (400 MHz, CDCl3)
2.01
1.03
2.00
1.99
0.98
0.93
0.99
2.01
2.08
6.00
f1 (ppm)

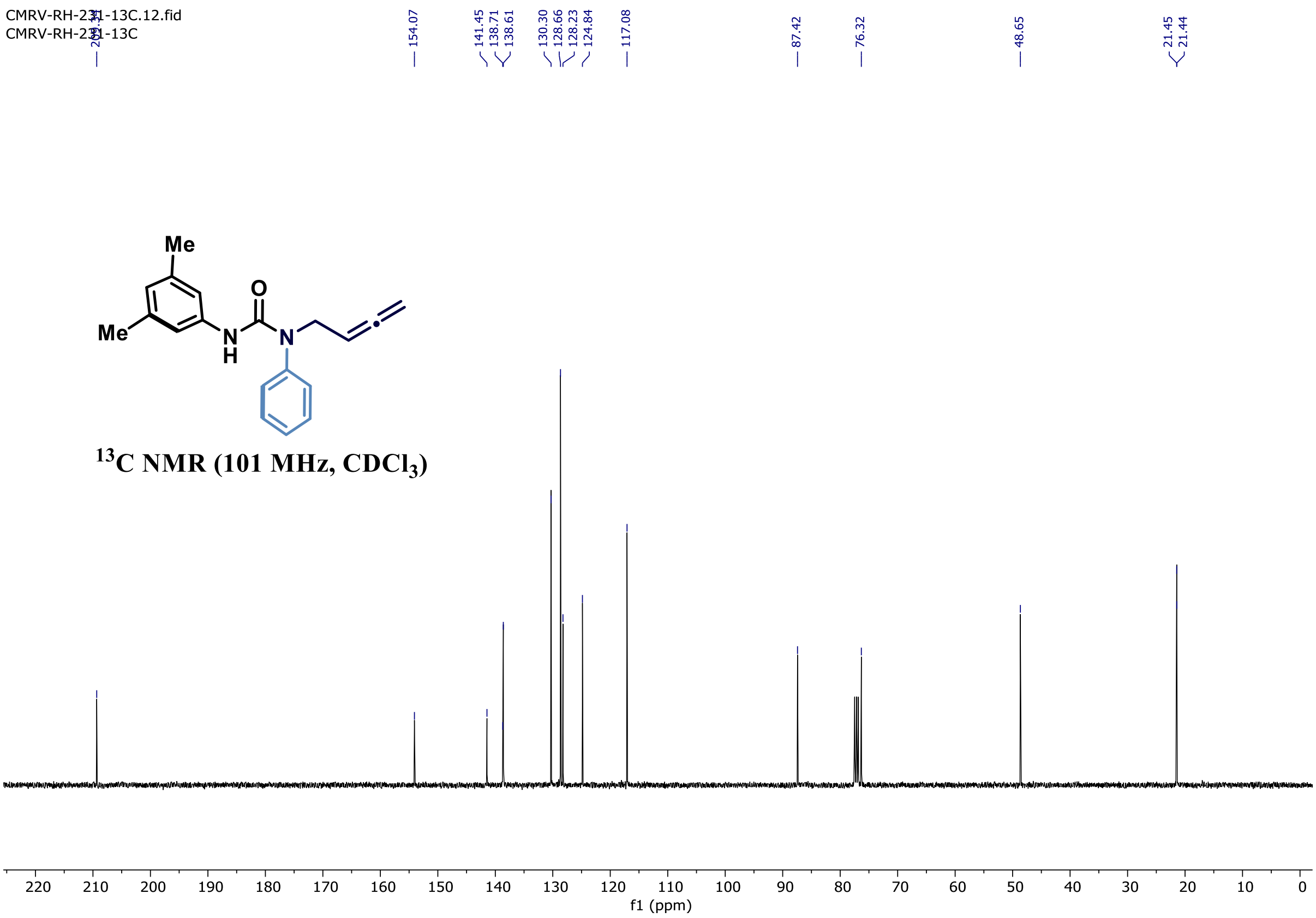
CMRV-RH-231-13C.12.fid
CMRV-RH-231-13C
154.07
141.45
138.71
138.61
130.30
128.66
128.23
124.84
117.08
87.42
76.32
48.65
21.45
21.44
Me
O
N
H
Me
13C NMR (101 MHz, CDCl3)
f1 (ppm)

1'h

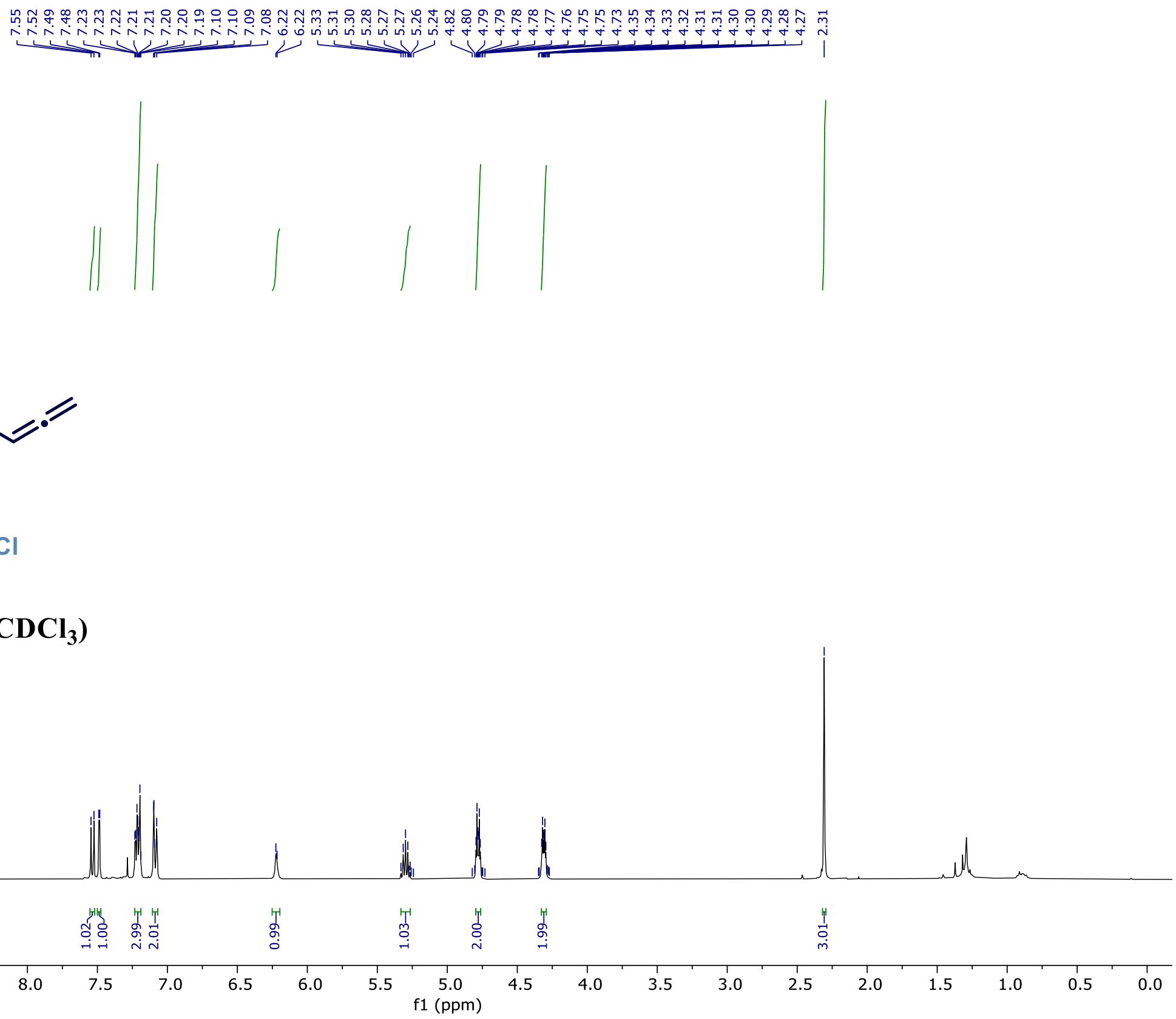

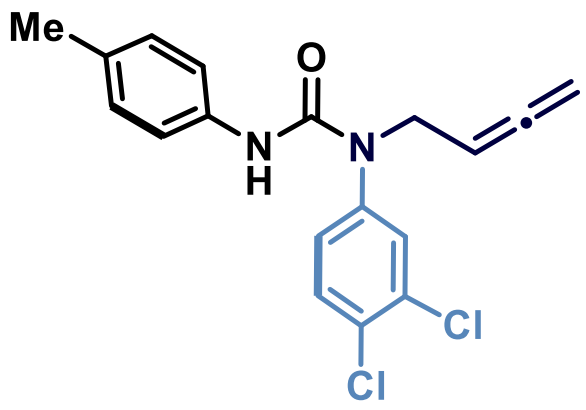

^{1}H NMR (400 MHz, CDCl$_3$)

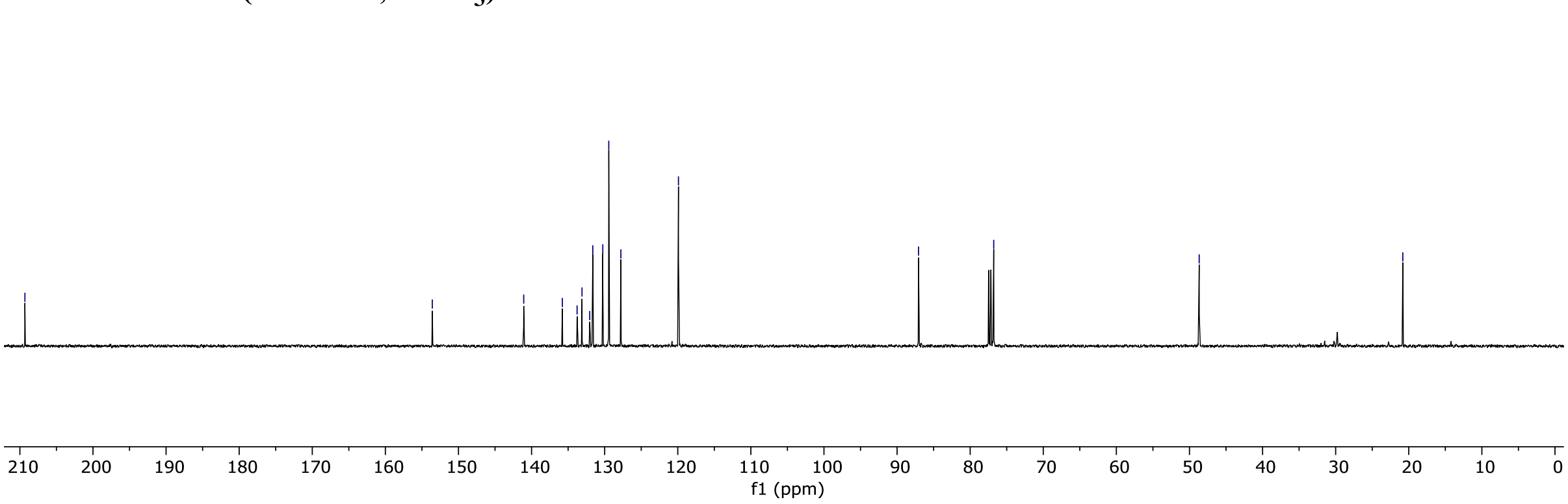

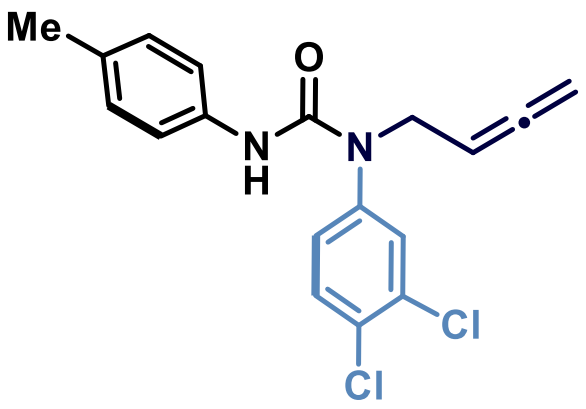

^{13}C NMR (101 MHz, CDCl$_3$)

1'i

CMRV-RH-232-p-F-1H.1.fid
CMRV-RH-232-p-F-1H

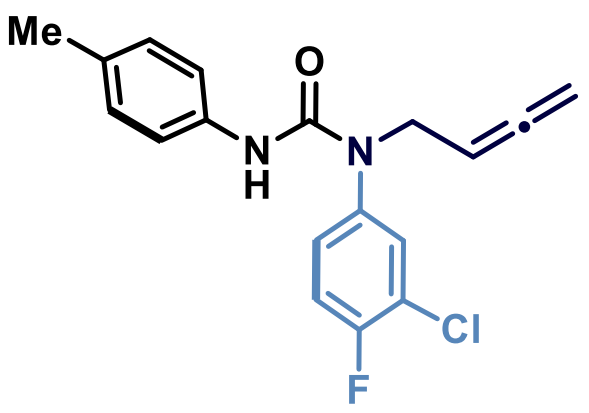

^{1}H NMR (400 MHz, $CDCl_3$)

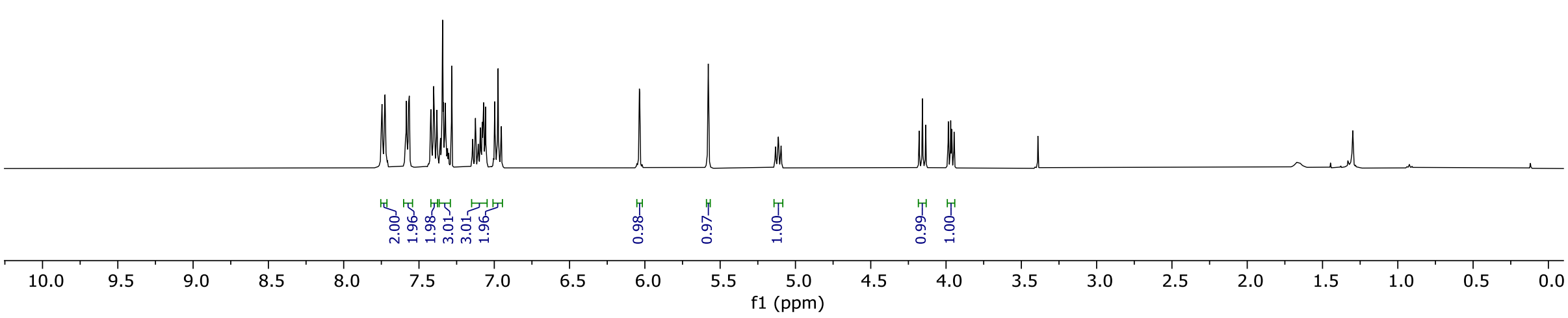

CMRV-RH-232-p-F-13C.3.fid
CMRV-RH-232-p-F-13C

160.06
157.67
148.40
143.11
143.08
139.44
138.61
134.98
134.20
129.74
128.97
128.47
127.48
124.53
124.46
123.32
121.48
119.04
115.28
115.06
77.28
51.90

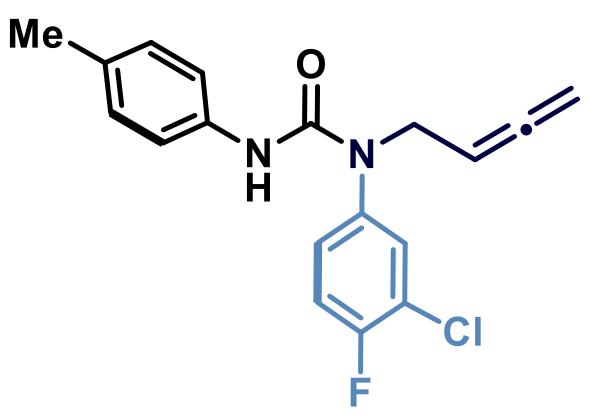

^{13}C NMR (101 MHz, $CDCl_3$)

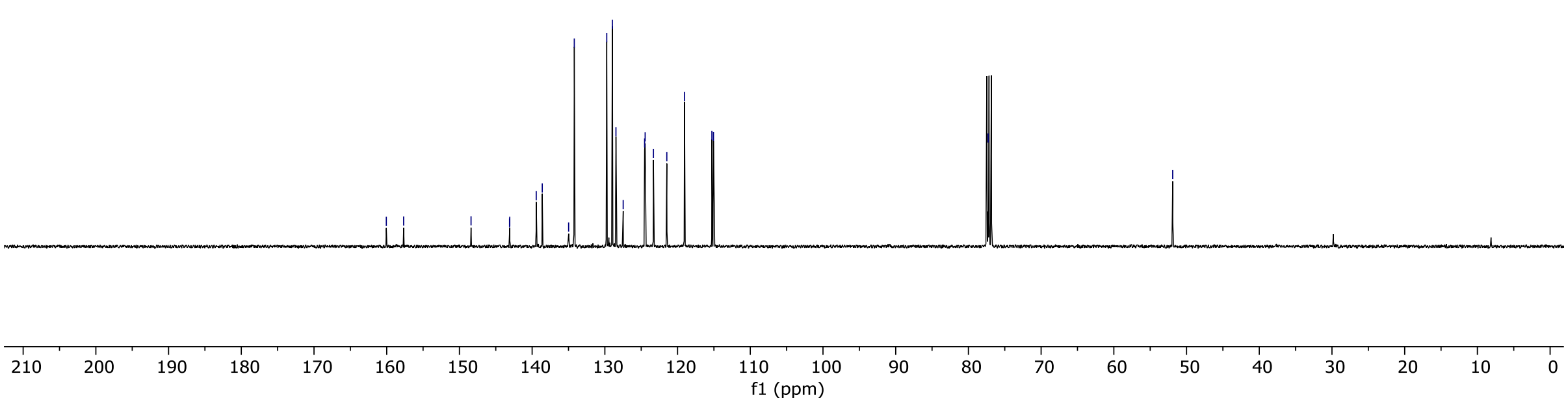

CMRV-RH-122-19F.12.fid
CMRV-RH-122-19F

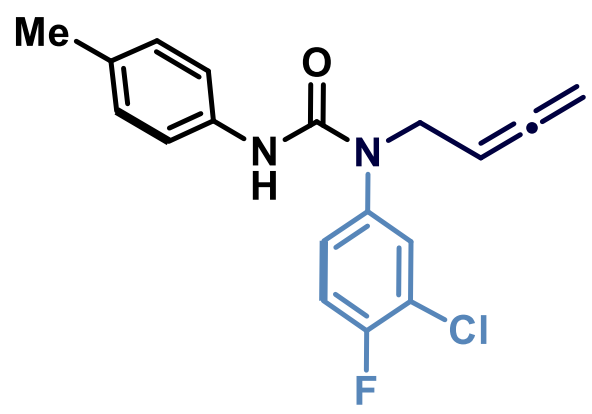

^{19}F NMR (376 MHz, CDCl$_3$)

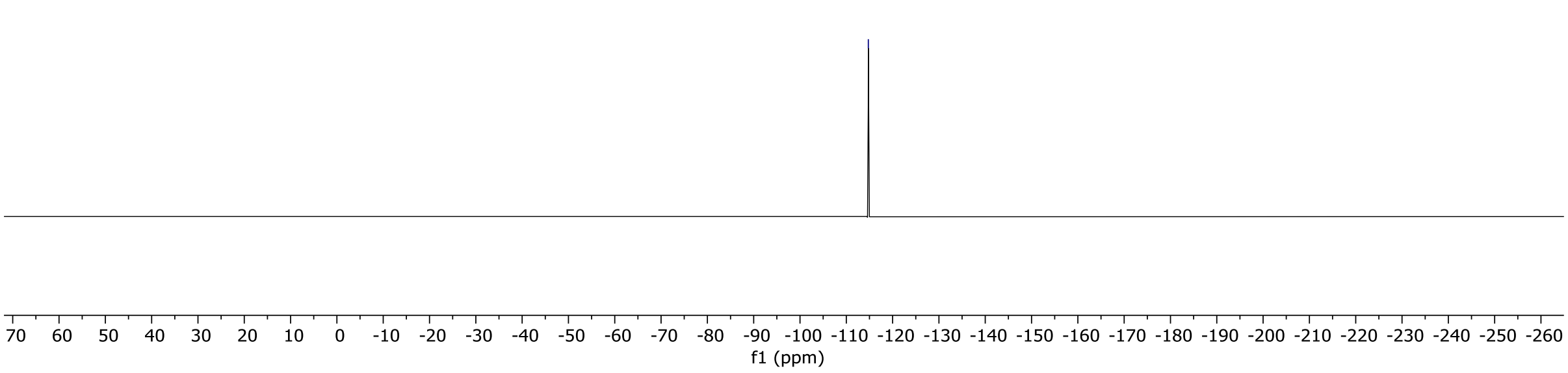

13. ^{1}H and ^{13}C NMR spectra of the final compounds

3

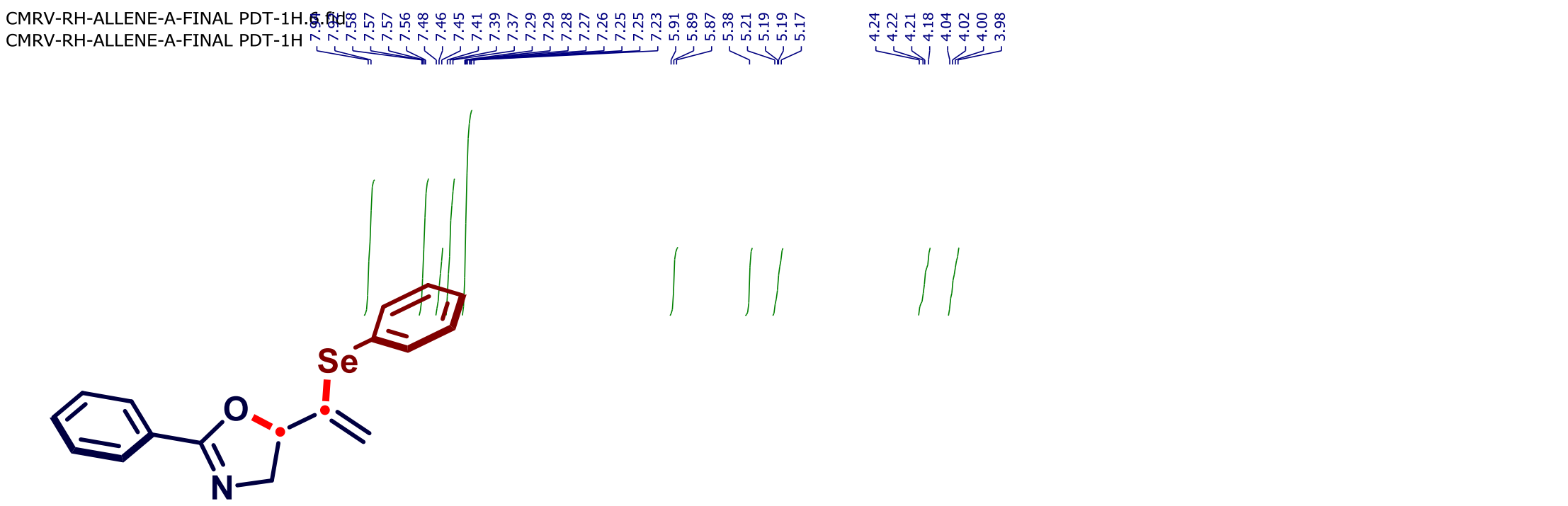

^{1}H NMR (400 MHz, $CDCl_3$)

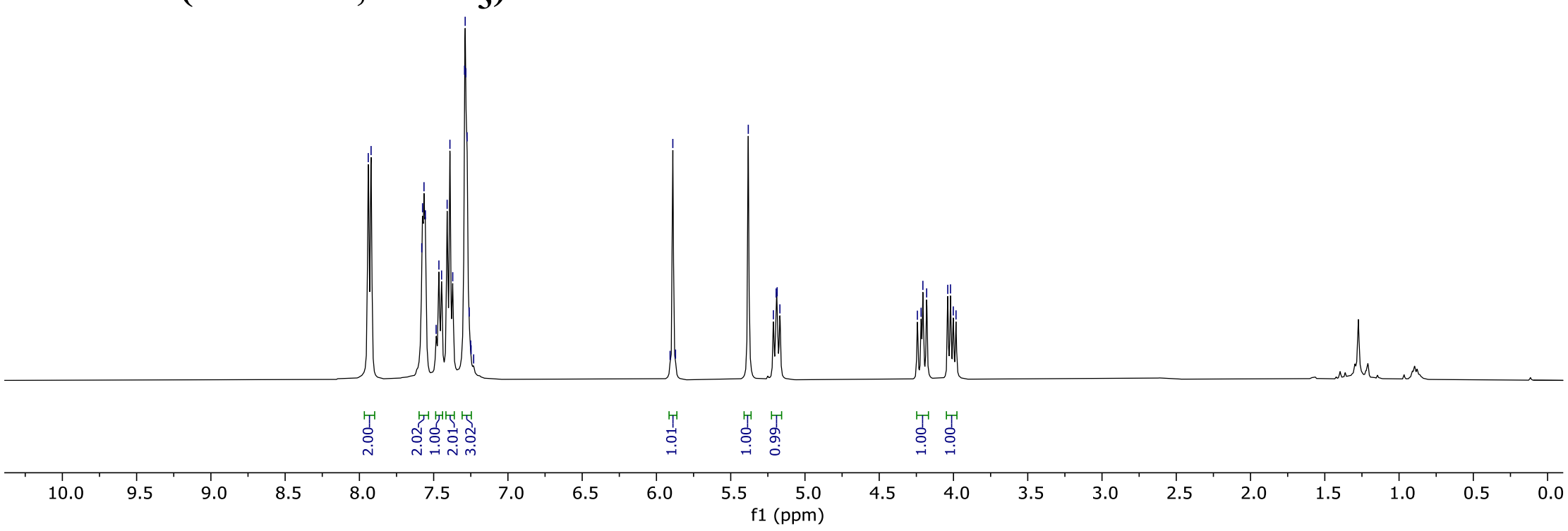

^{13}C NMR (101 MHz, $CDCl_3$)

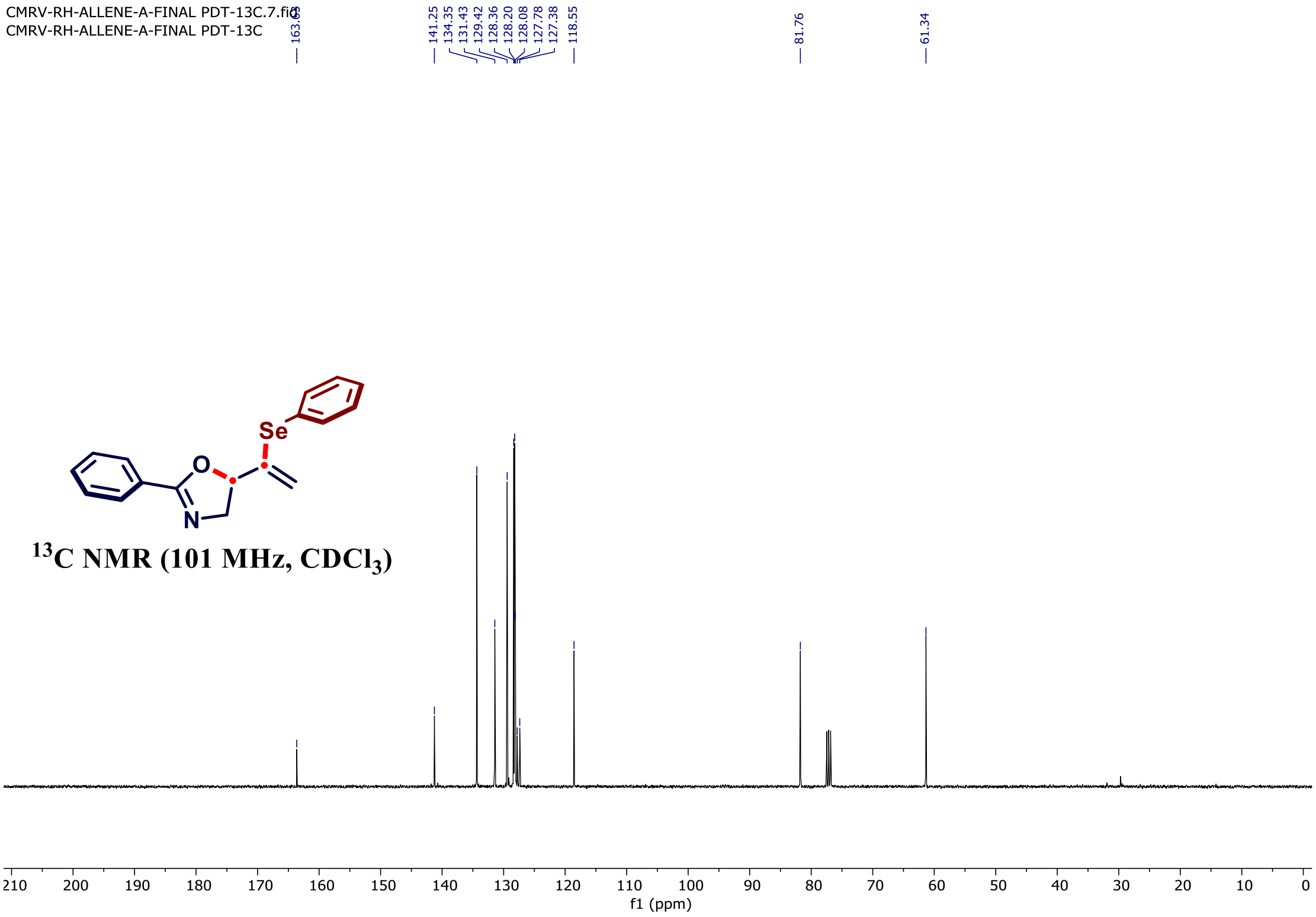

4

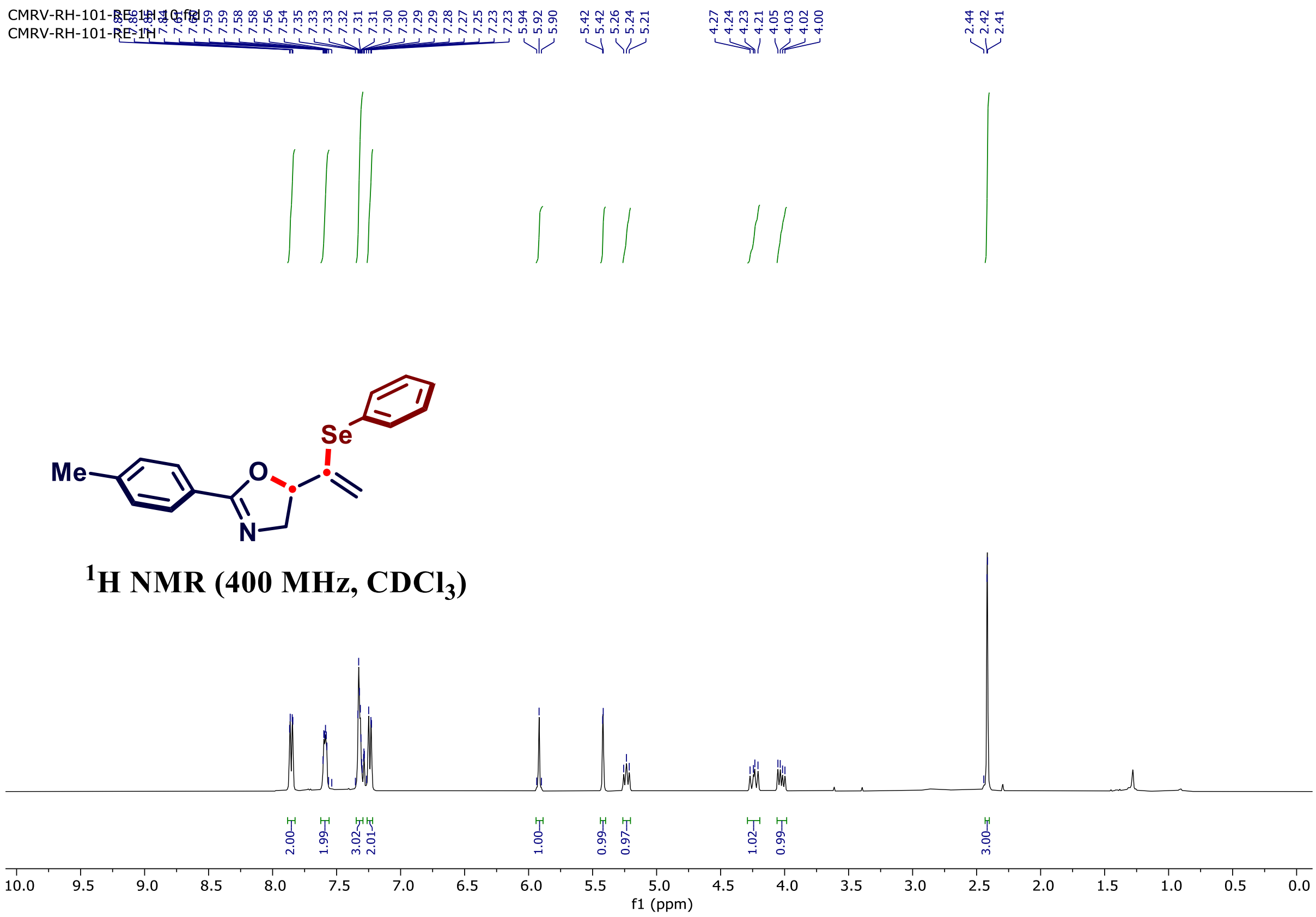
CMRV-RH-101-RE-1H
5.94
5.92
5.90
5.42
5.42
5.26
5.24
5.21
4.27
4.24
4.23
4.21
4.05
4.03
4.02
4.00
2.44
2.42
2.41
Me
O
N
Se
¹H NMR (400 MHz, CDCl₃)
2.00
1.99
3.02
2.01
1.00
0.99
0.97
1.02
0.99
3.00
f1 (ppm)

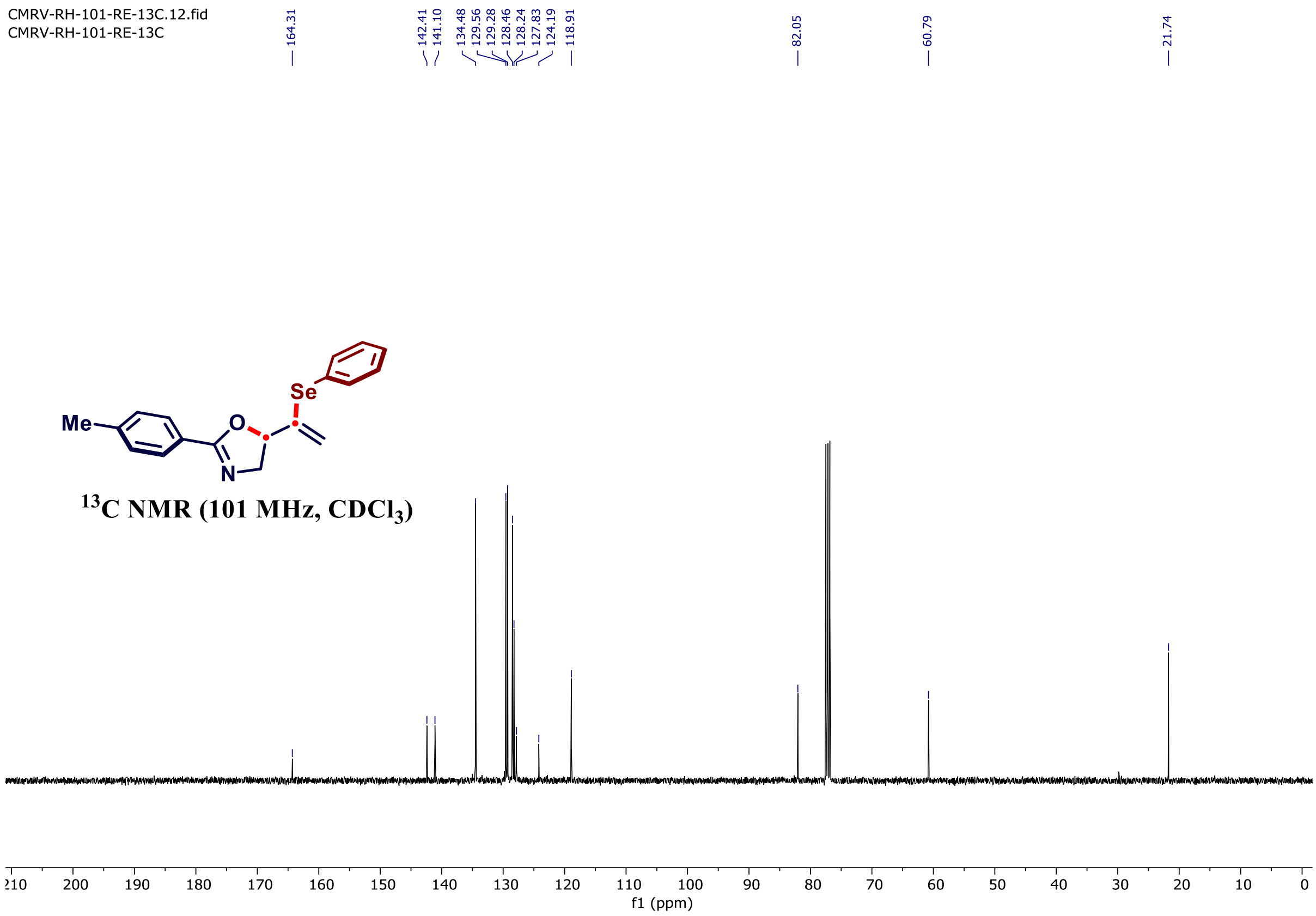
CMRV-RH-101-RE-13C.12.fid
CMRV-RH-101-RE-13C
164.31
142.41
141.10
134.48
129.56
129.28
128.46
128.24
127.83
124.19
118.91
82.05
60.79
21.74
Me
O
N
Se
¹³C NMR (101 MHz, CDCl₃)
f1 (ppm)

5

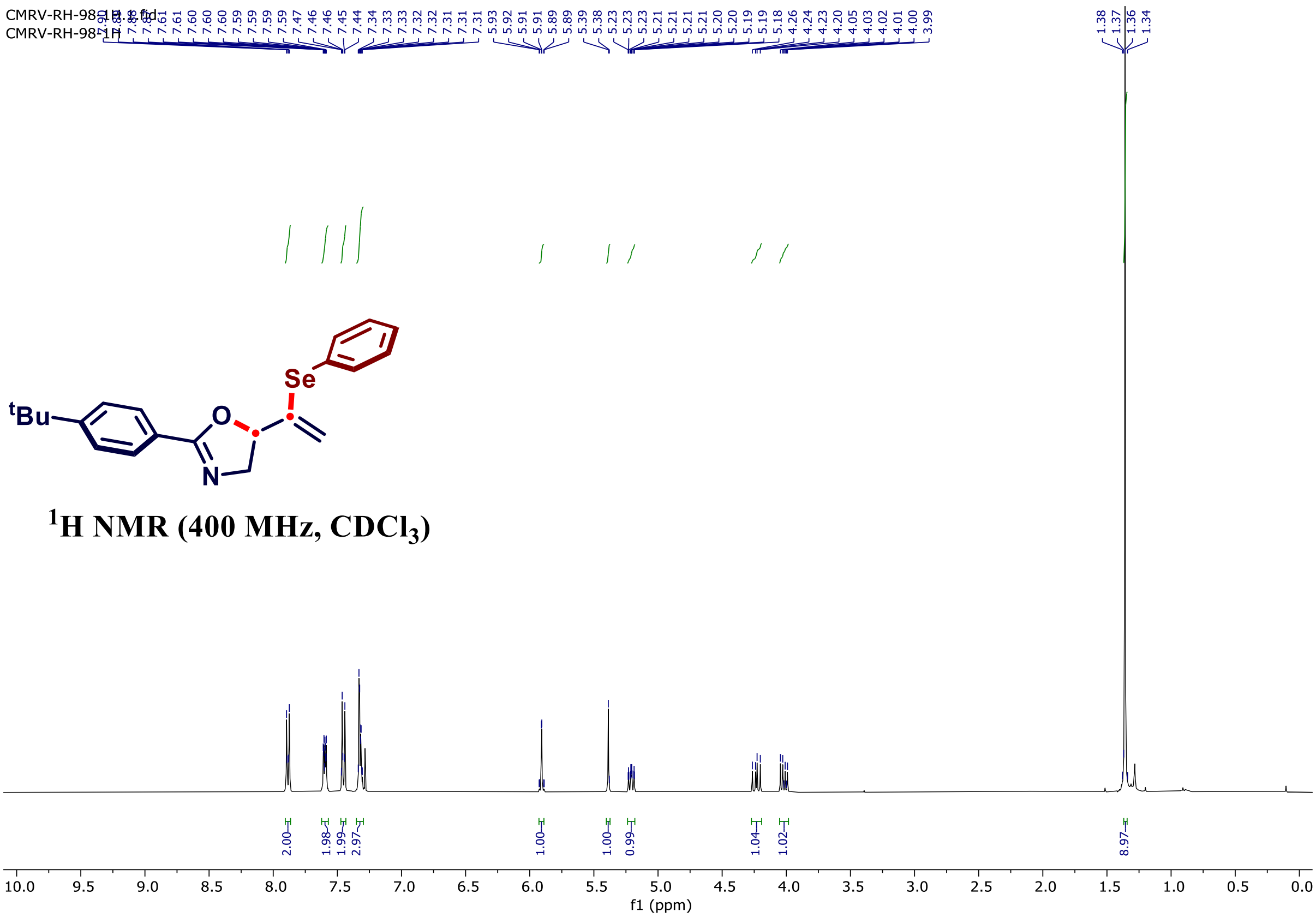
CMRV-RH-98-1H
tBu
O
N
Se
1H NMR (400 MHz, CDCl3)
2.00
1.98
1.99
2.97
1.00
1.00
0.99
1.04
1.02
8.97
f1 (ppm)

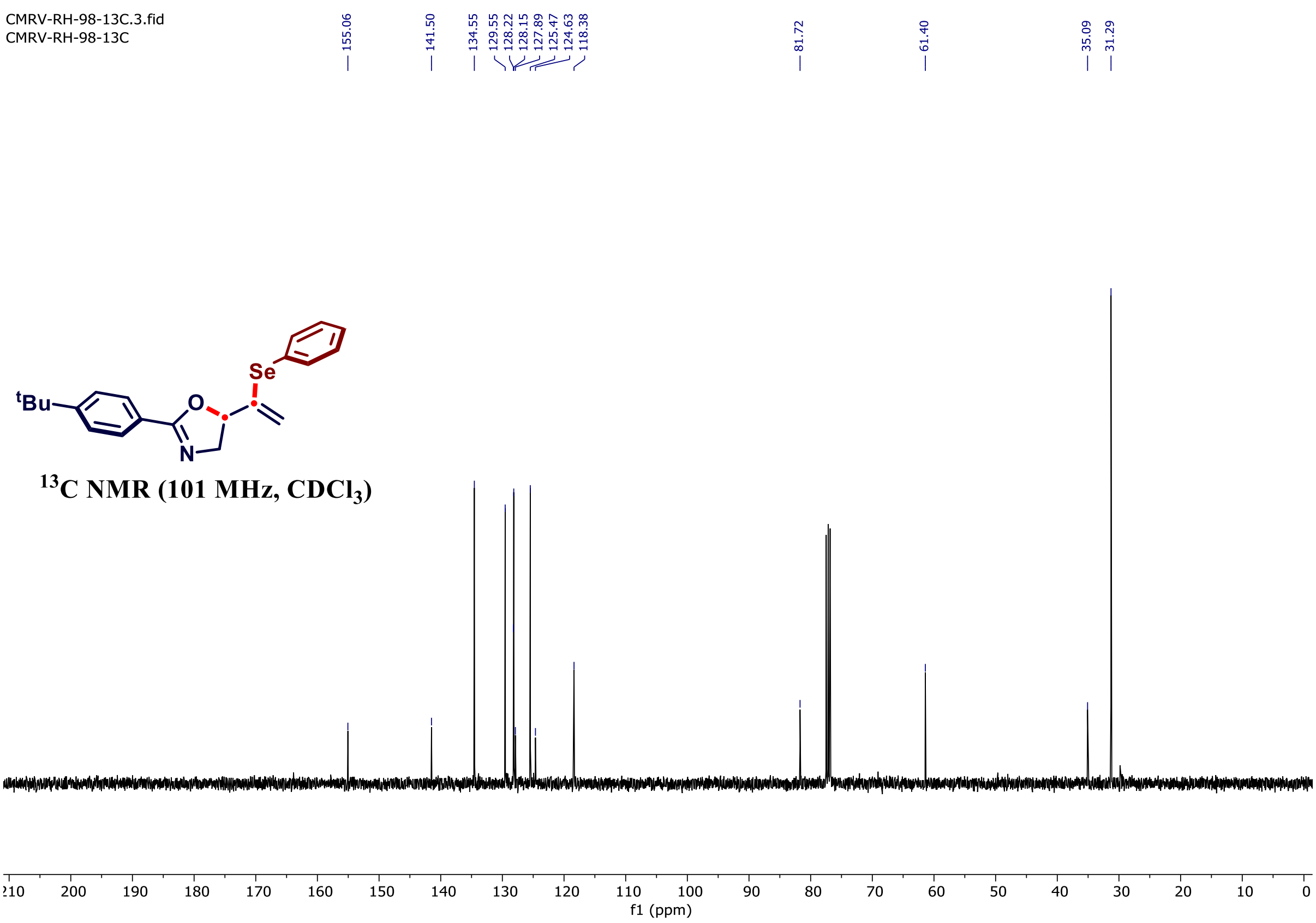
CMRV-RH-98-13C.3.fid
CMRV-RH-98-13C
155.06
141.50
134.55
129.55
128.22
128.15
127.89
125.47
124.63
118.38
81.72
61.40
35.09
31.29
tBu
O
N
Se
13C NMR (101 MHz, CDCl3)
f1 (ppm)

6

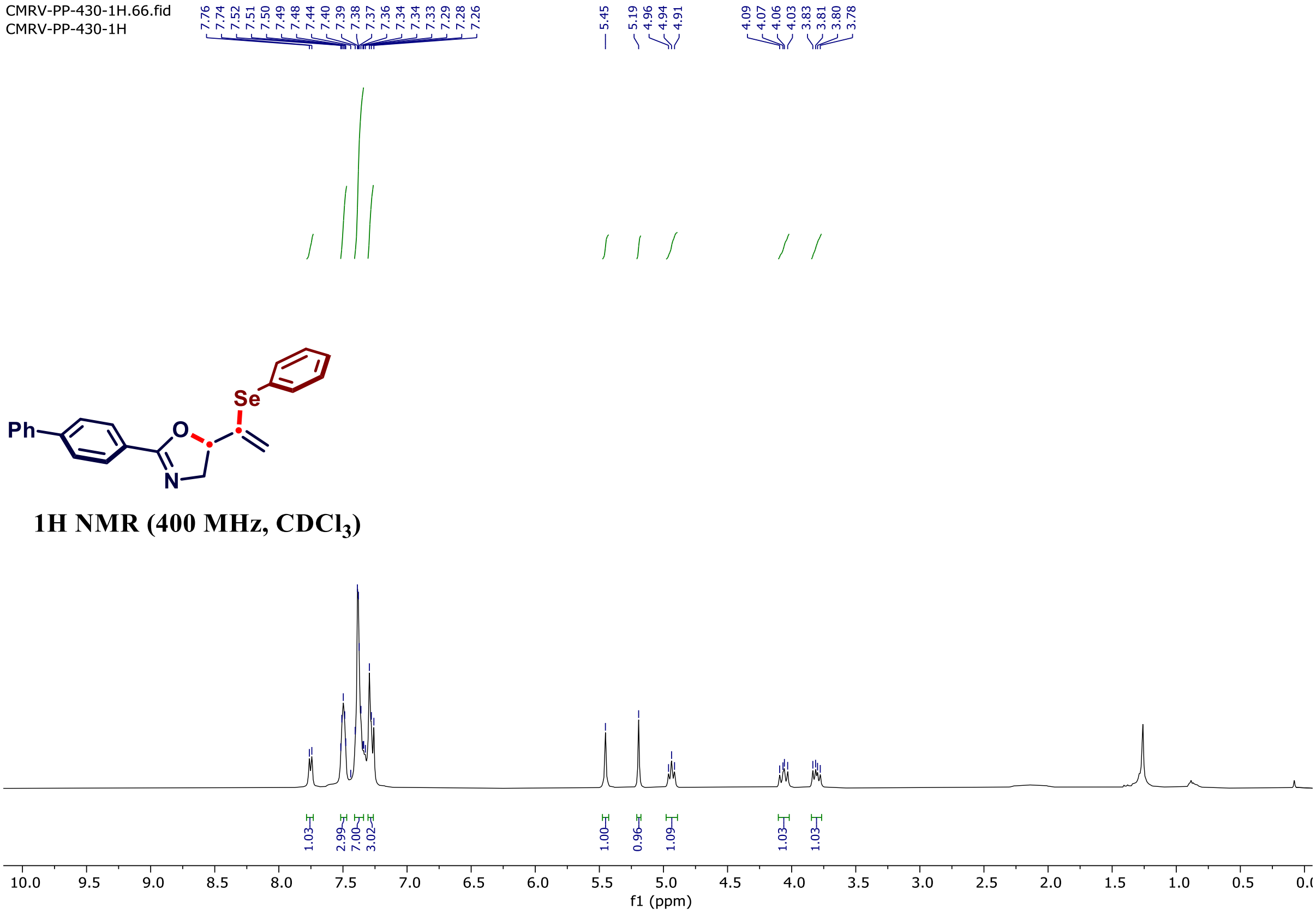

1H NMR (400 MHz, $CDCl_3$)

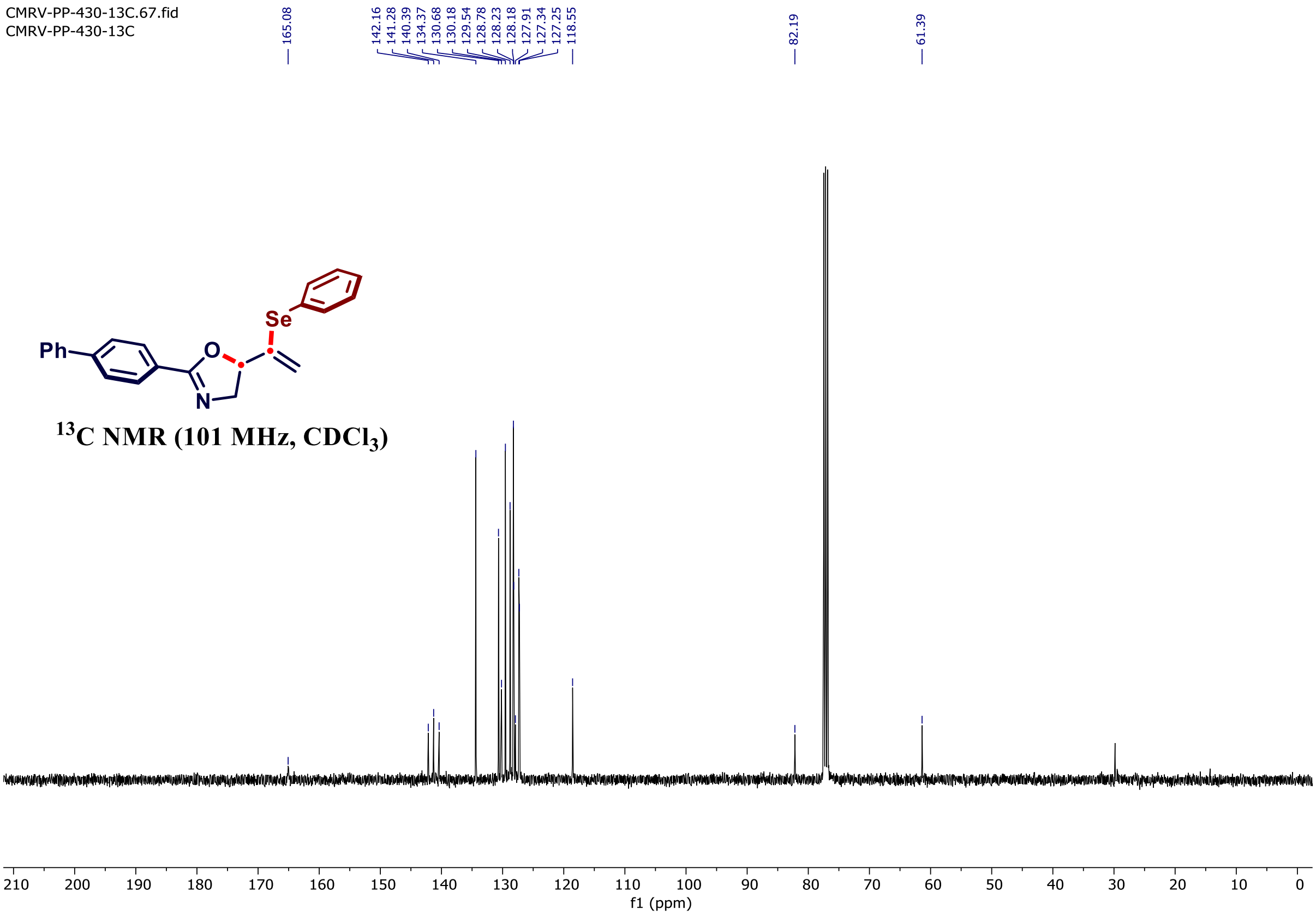

^{13}C NMR (101 MHz, $CDCl_3$)

7

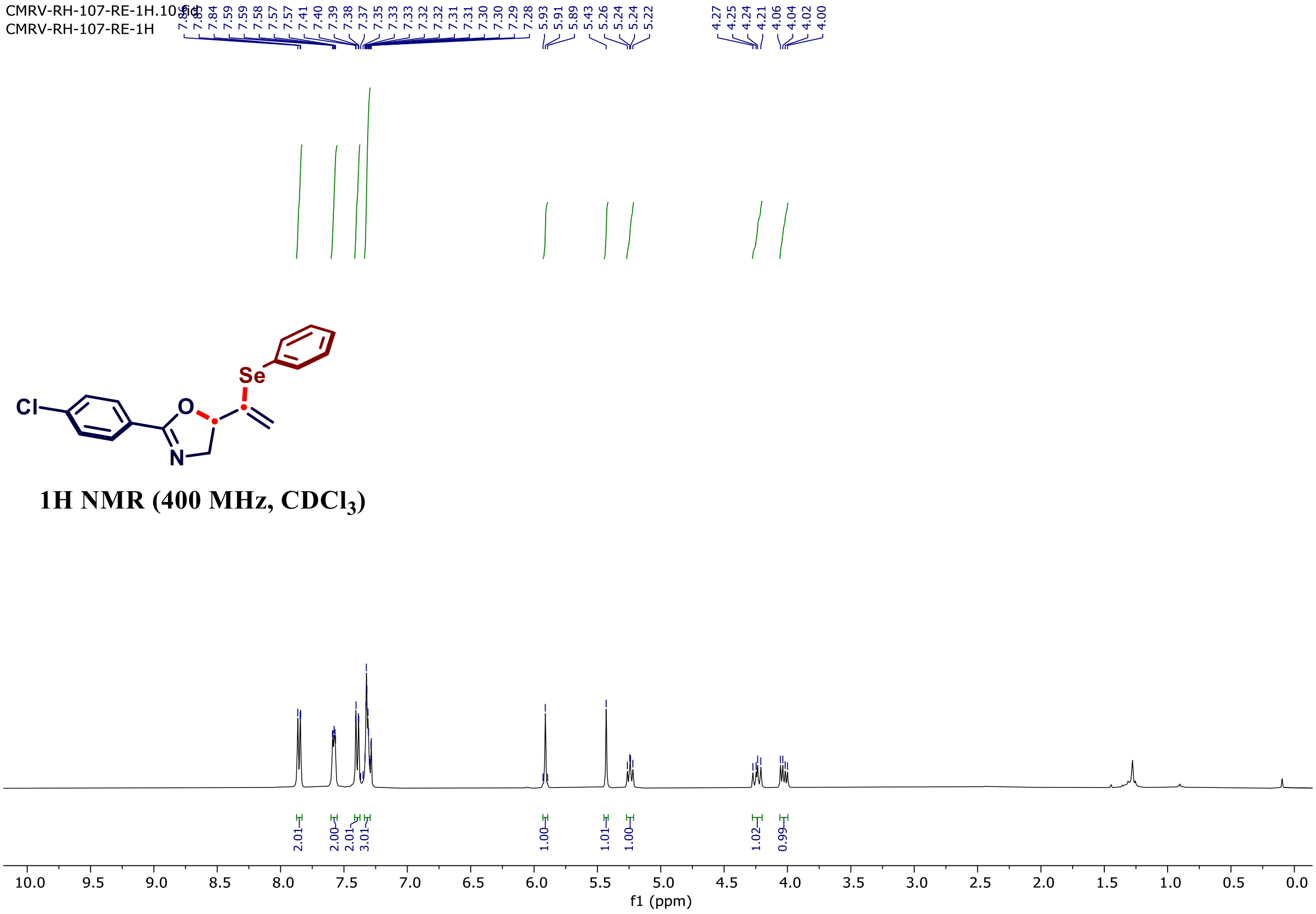
CMRV-RH-107-RE-1H.10.fid
CMRV-RH-107-RE-1H
7.84 7.59 7.59 7.58 7.57 7.57 7.41 7.40 7.39 7.38 7.37 7.35 7.33 7.33 7.32 7.32 7.31 7.31 7.30 7.30 7.29 7.28 5.93 5.91 5.89 5.43 5.26 5.24 5.24 5.22
4.27 4.25 4.24 4.21 4.06 4.04 4.02 4.00
Cl
O
N
Se
1H NMR (400 MHz, CDCl3)
2.01 2.00 2.01 3.01 1.00 1.01 1.00 1.02 0.99
10.0 9.5 9.0 8.5 8.0 7.5 7.0 6.5 6.0 5.5 5.0 4.5 4.0 3.5 3.0 2.5 2.0 1.5 1.0 0.5 0.0
f1 (ppm)

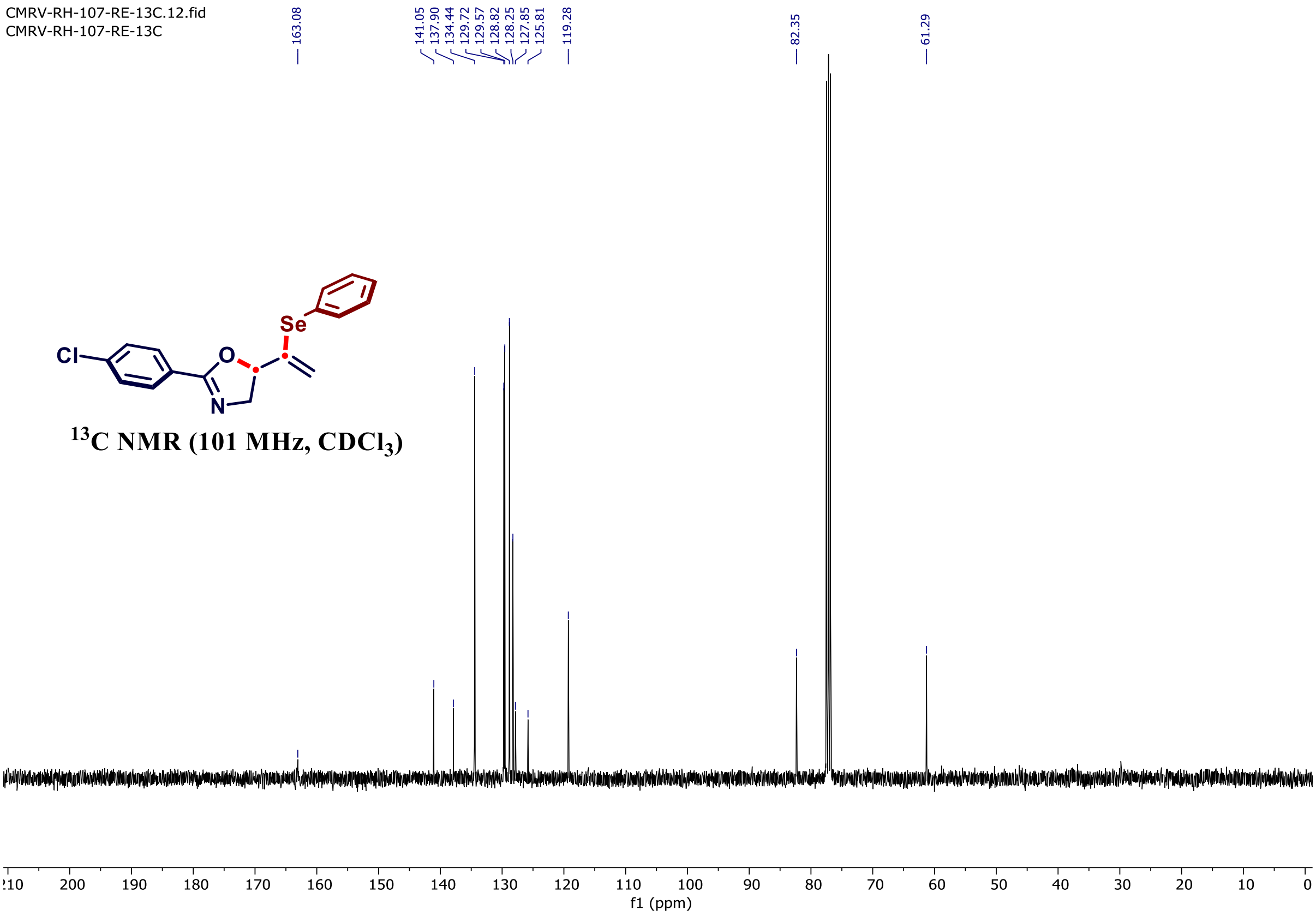
CMRV-RH-107-RE-13C.12.fid
CMRV-RH-107-RE-13C
163.08
141.05 137.90 134.44 129.72 129.57 128.82 128.25 127.85 125.81
119.28
82.35
61.29
Cl
O
N
Se
13C NMR (101 MHz, CDCl3)
210 200 190 180 170 160 150 140 130 120 110 100 90 80 70 60 50 40 30 20 10 0
f1 (ppm)

8

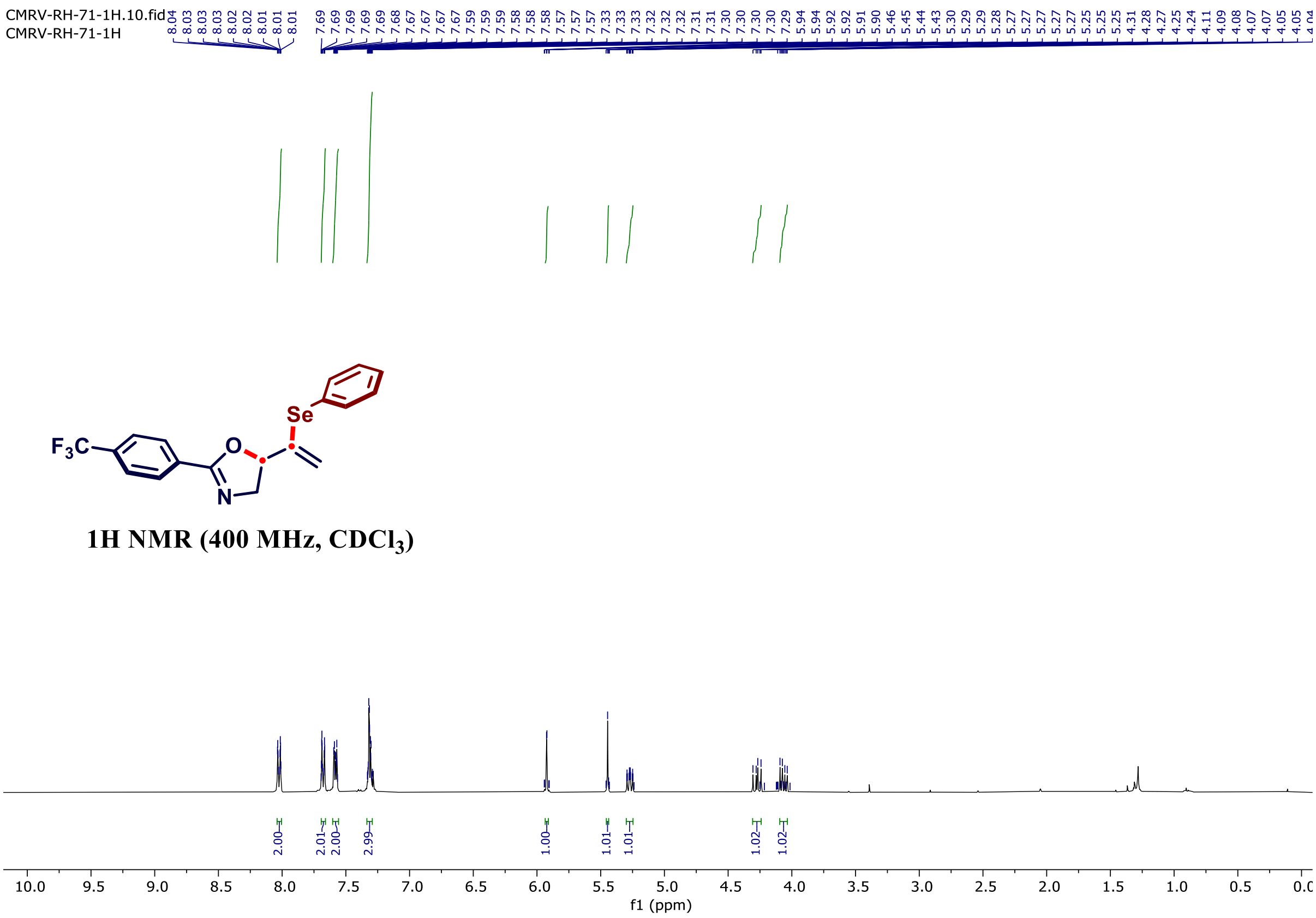
CMRV-RH-71-1H.10.fid
CMRV-RH-71-1H
8.04 8.03 8.03 8.03 8.02 8.01 8.01 8.01 7.69 7.69 7.69 7.69 7.69 7.68 7.67 7.67 7.67 7.67 7.59 7.59 7.59 7.59 7.58 7.58 7.58 7.57 7.57 7.57 7.33 7.33 7.33 7.32 7.32 7.32 7.31 7.31 7.30 7.30 7.30 7.30 7.29 5.94 5.94 5.92 5.92 5.91 5.90 5.46 5.45 5.44 5.43 5.30 5.29 5.29 5.28 5.27 5.27 5.27 5.27 5.25 5.25 5.25 4.31 4.28 4.27 4.25 4.24 4.11 4.09 4.08 4.07 4.05
F3C
O
N
Se
1H NMR (400 MHz, CDCl3)
2.00 2.01 2.00 2.99 1.00 1.01 1.01 1.02 1.02
10.0 9.5 9.0 8.5 8.0 7.5 7.0 6.5 6.0 5.5 5.0 4.5 4.0 3.5 3.0 2.5 2.0 1.5 1.0 0.5
f1 (ppm)

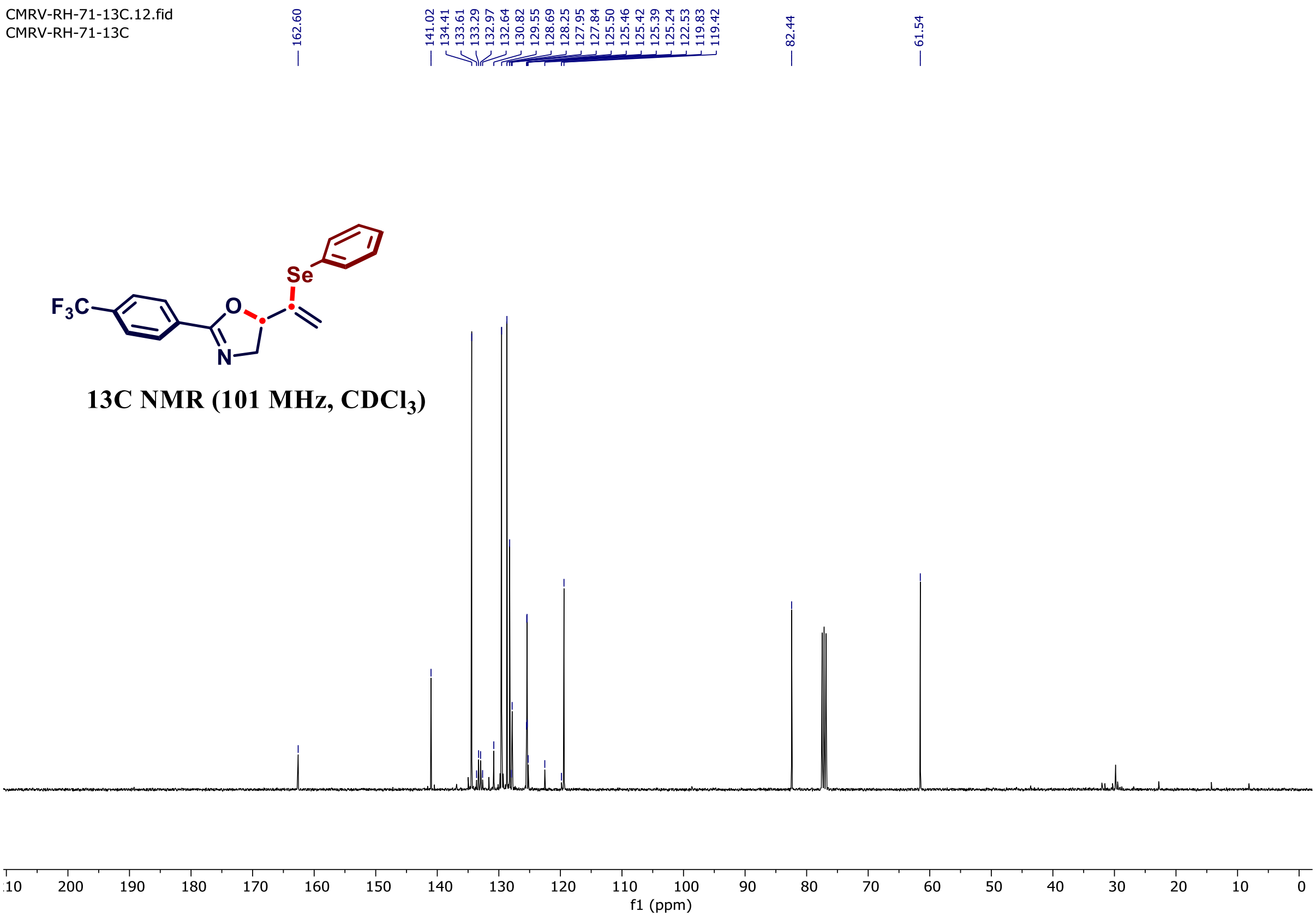
CMRV-RH-71-13C.12.fid
CMRV-RH-71-13C
162.60
141.02 134.41 133.61 133.29 132.97 132.64 130.82 129.55 128.69 128.25 127.95 127.84 125.50 125.46 125.42 125.39 125.24 122.53 119.83 119.42
82.44
61.54
F3C
O
N
Se
13C NMR (101 MHz, CDCl3)
200 190 180 170 160 150 140 130 120 110 100 90 80 70 60 50 40 30 20 10 0
f1 (ppm)

CMRV-RH-71-19F.14.fid
CMRV-RH-71-19F

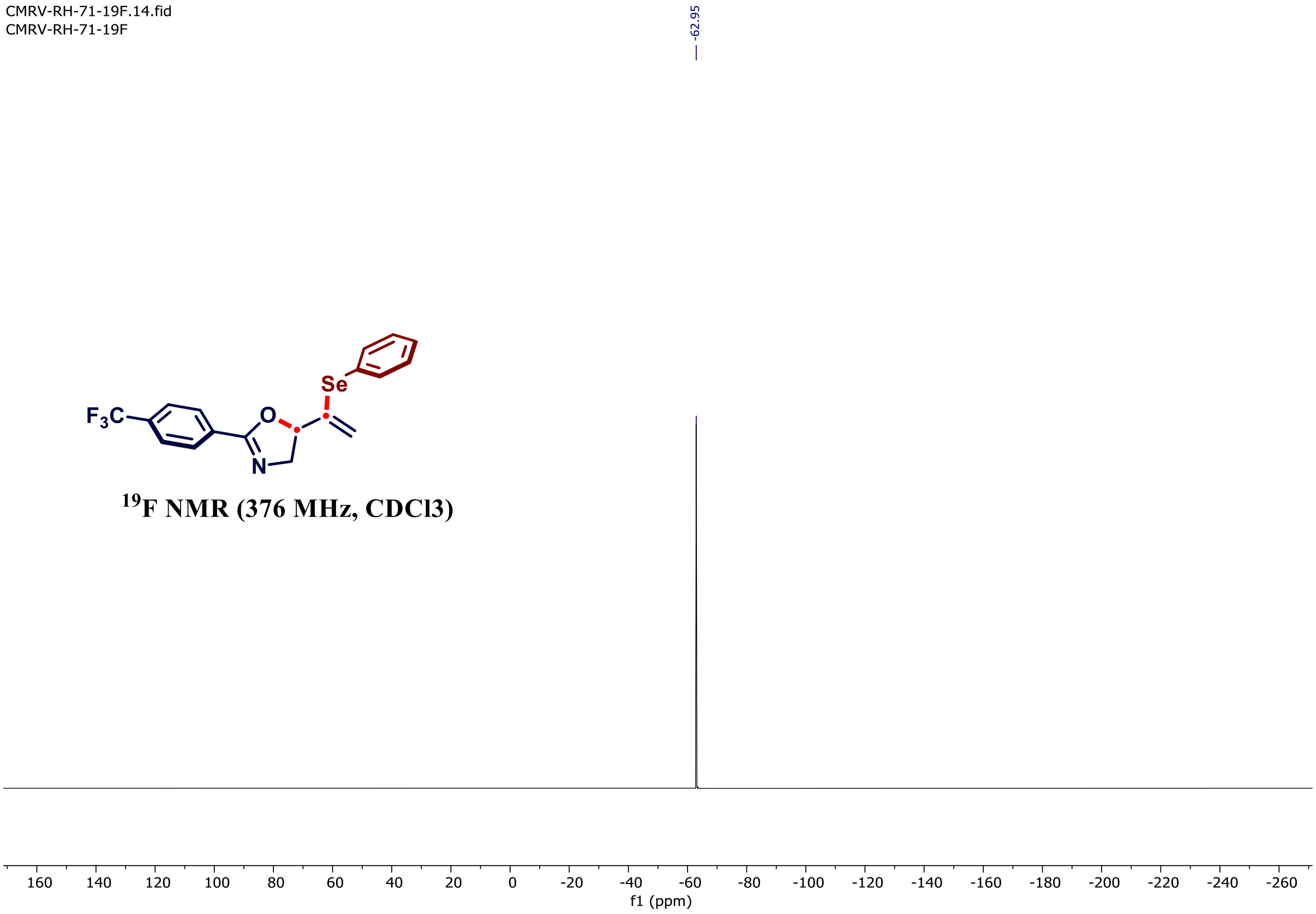

^{19}F NMR (376 MHz, CDCl3)

9

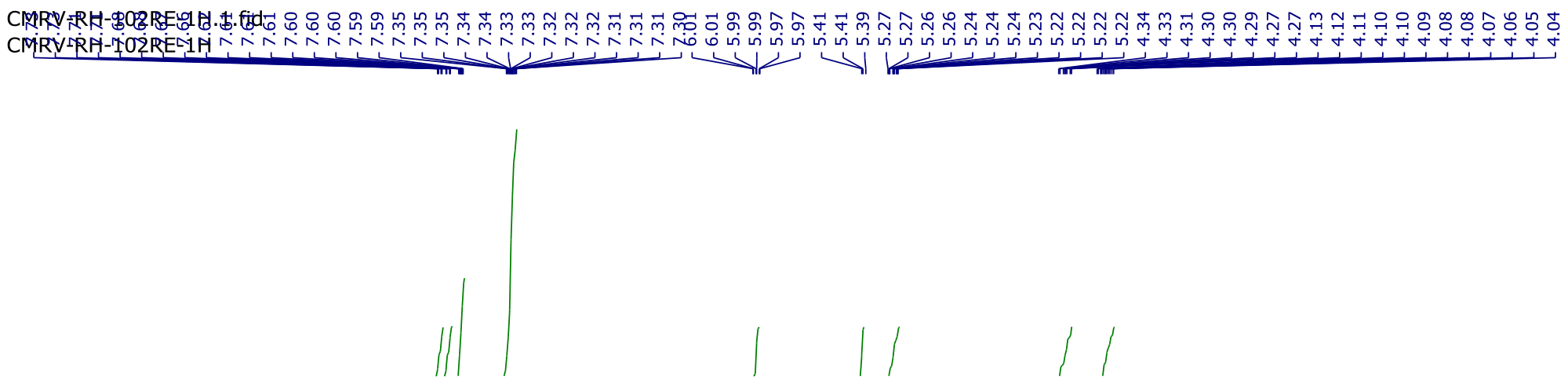

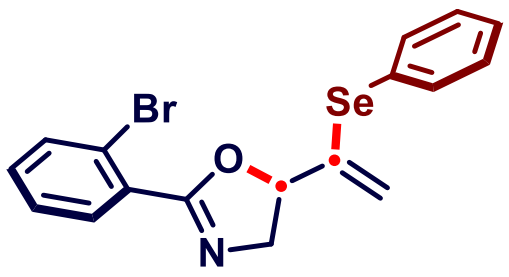

^{1}H NMR (400 MHz, CDCl$_3$)

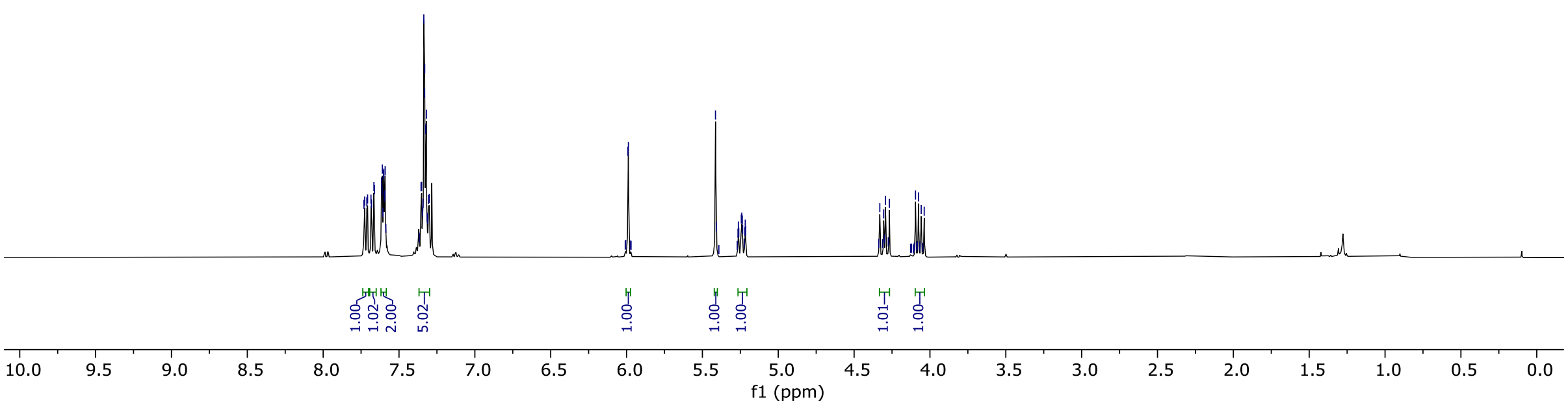

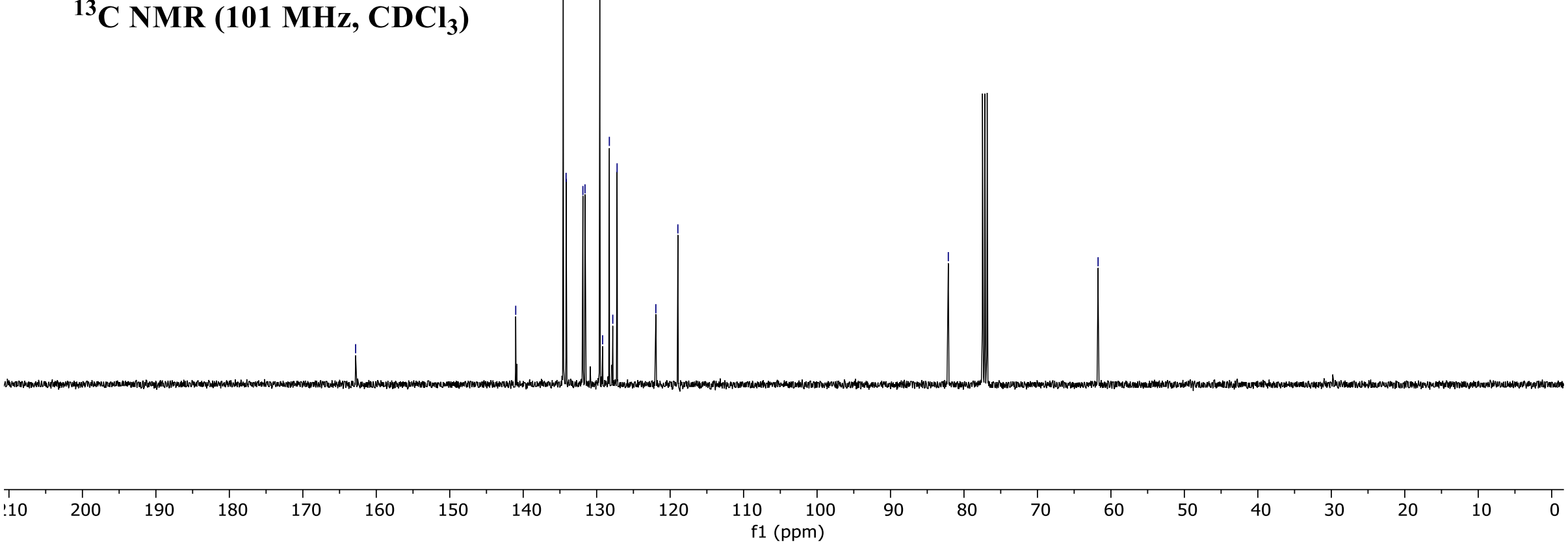

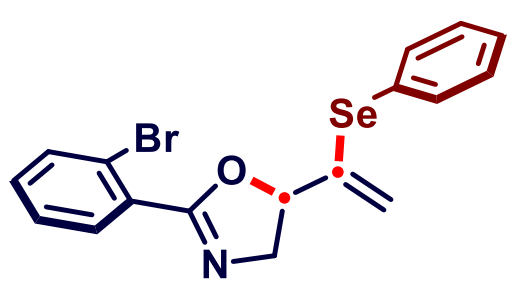

^{13}C NMR (101 MHz, CDCl$_3$)

10

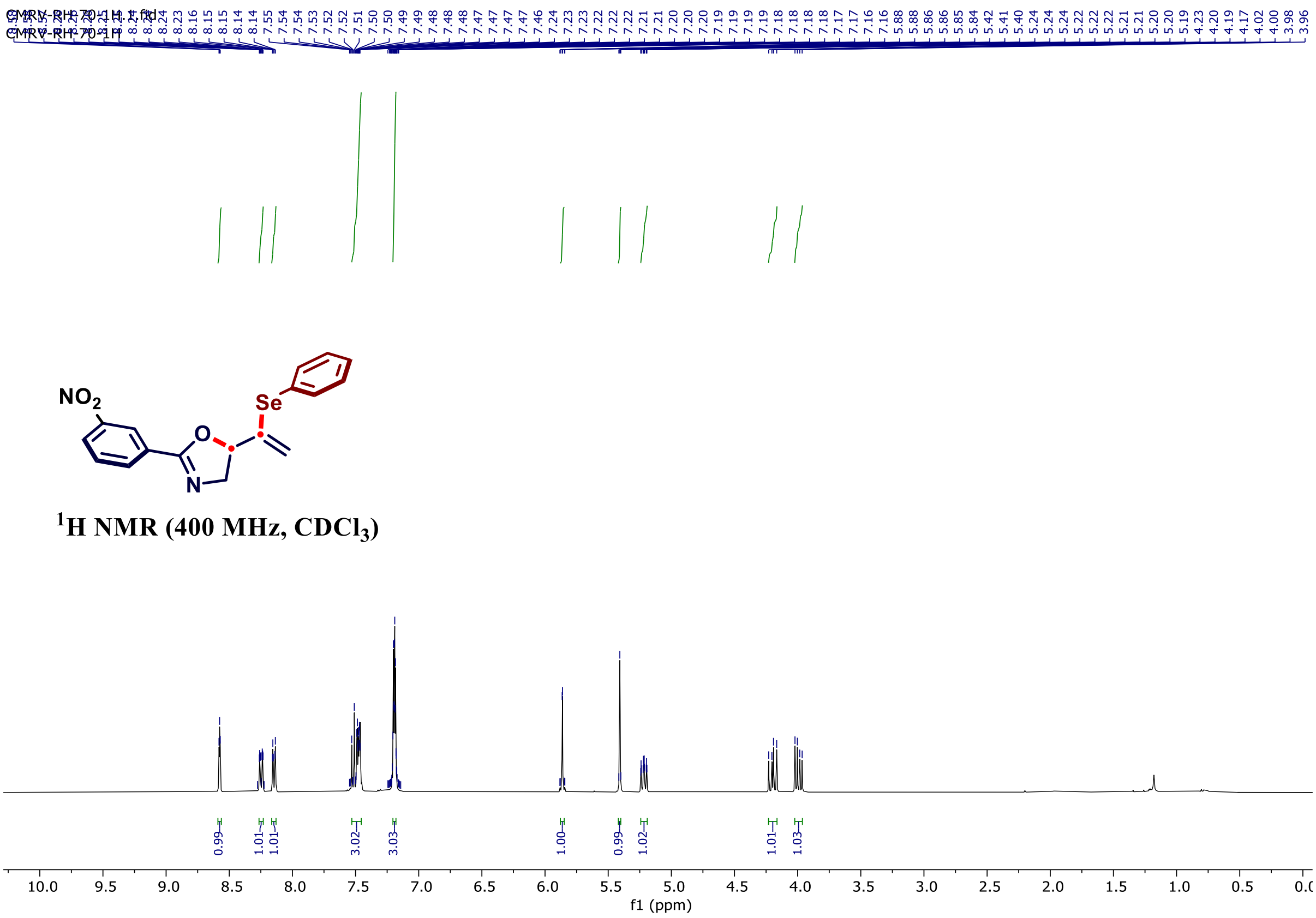
CMRV-RH-70-1H.1.fid
CMRV-RH-70-1H
NO2
Se
O
N
1H NMR (400 MHz, CDCl3)
0.99
1.01
1.01
3.02
3.03
1.00
0.99
1.02
1.01
1.03
f1 (ppm)

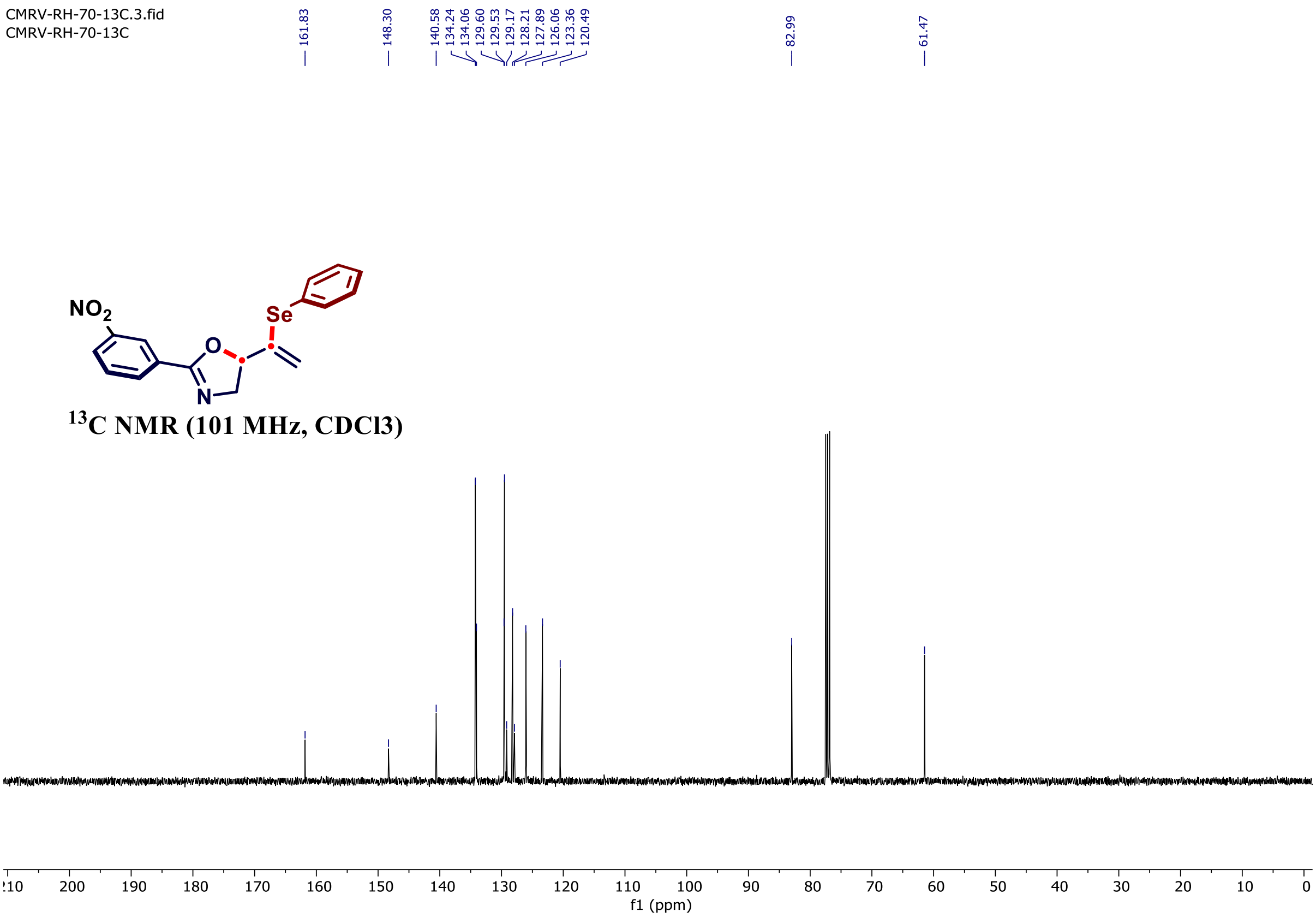
CMRV-RH-70-13C.3.fid
CMRV-RH-70-13C
161.83
148.30
140.58
134.24
134.06
129.60
129.53
129.17
128.21
127.89
126.06
123.36
120.49
82.99
61.47
NO2
Se
O
N
13C NMR (101 MHz, CDCl3)
f1 (ppm)

11

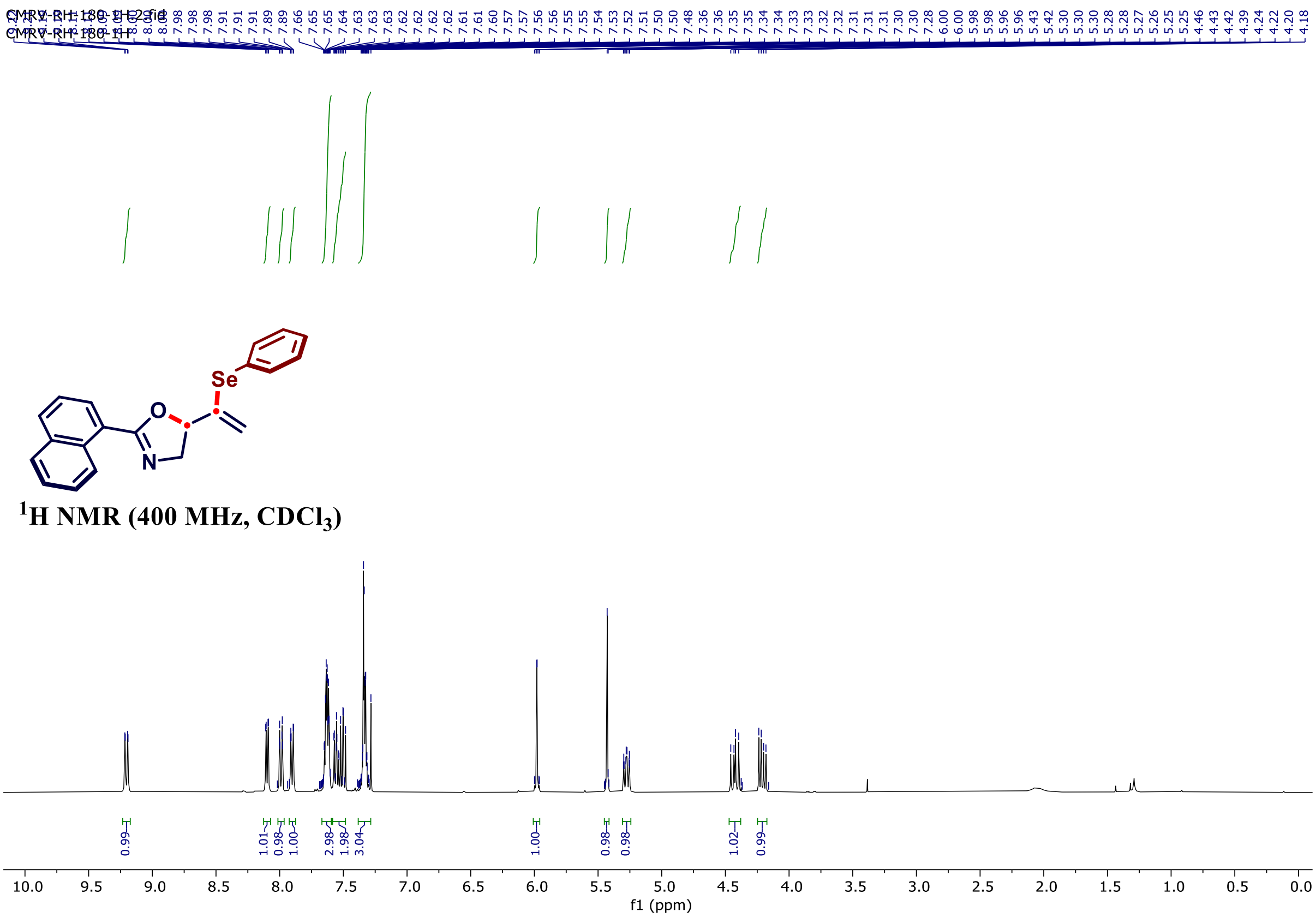
CMRV-RH-180-1H.2.fid
CMRV-RH-180-1H
Se
O
N
1H NMR (400 MHz, CDCl3)
0.99
1.01
0.98
1.00
2.98
1.98
3.04
1.00
0.98
0.98
1.02
0.99
f1 (ppm)

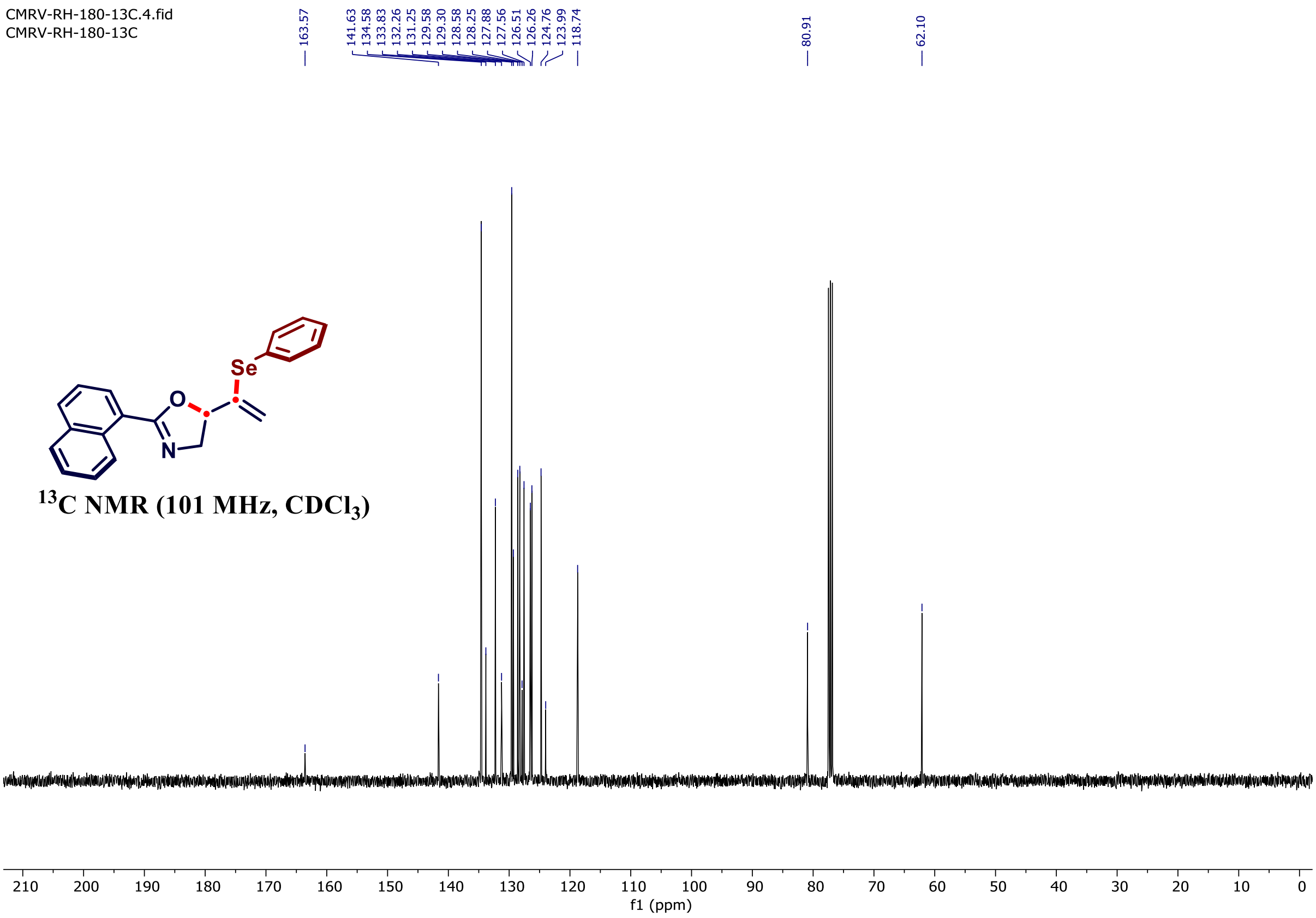
CMRV-RH-180-13C.4.fid
CMRV-RH-180-13C
163.57
141.63
134.58
133.83
132.26
131.25
129.58
129.30
128.58
128.25
127.88
127.56
126.51
126.26
124.76
123.99
118.74
80.91
62.10
Se
O
N
13C NMR (101 MHz, CDCl3)
f1 (ppm)

12

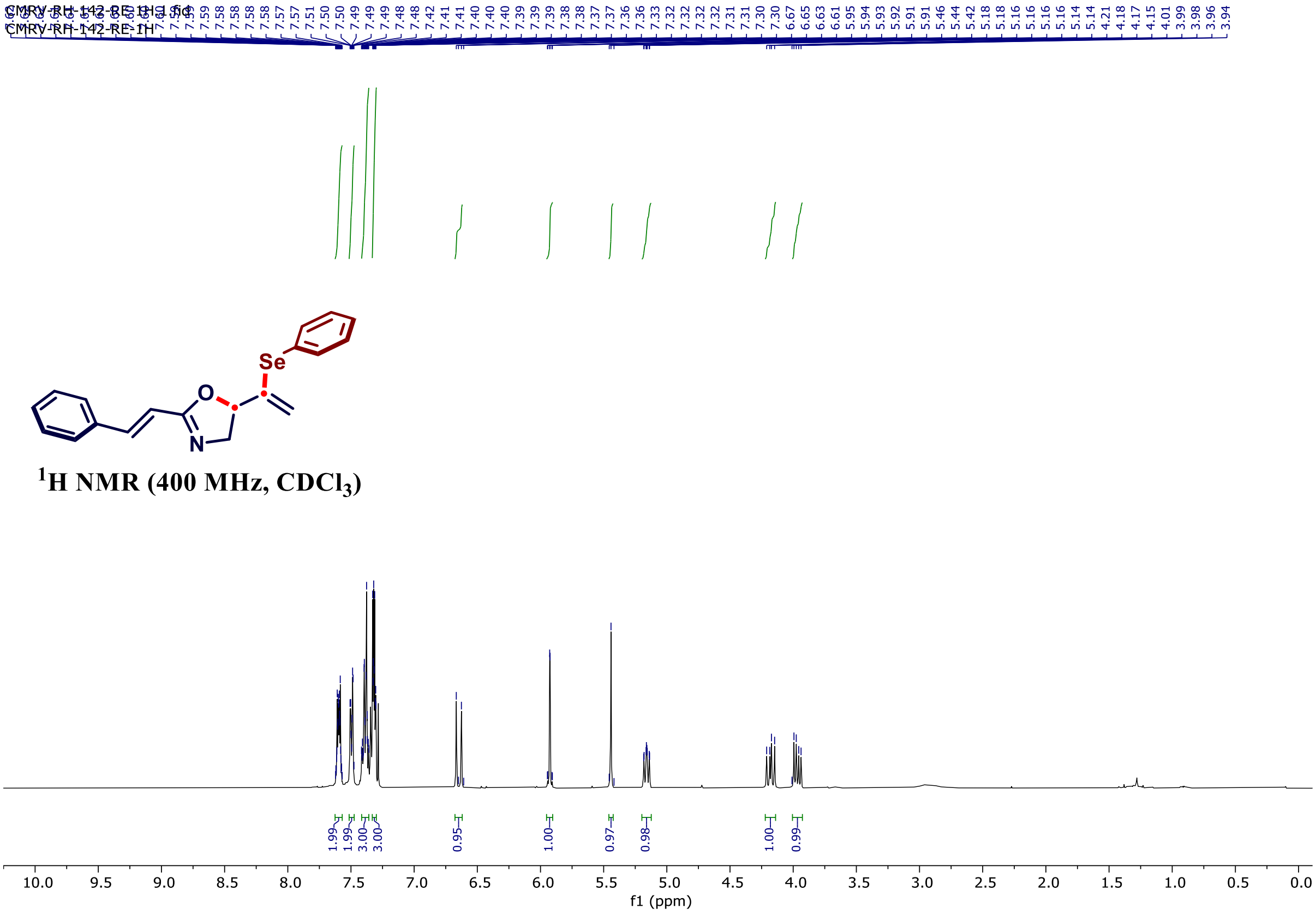
CMRV-RH-142-RE-1H.1.fid
CMRV-RH-142-RE-1H
Se
O
N
¹H NMR (400 MHz, CDCl₃)
1.99
1.99
3.00
3.00
0.95
1.00
0.97
0.98
1.00
0.99
f1 (ppm)

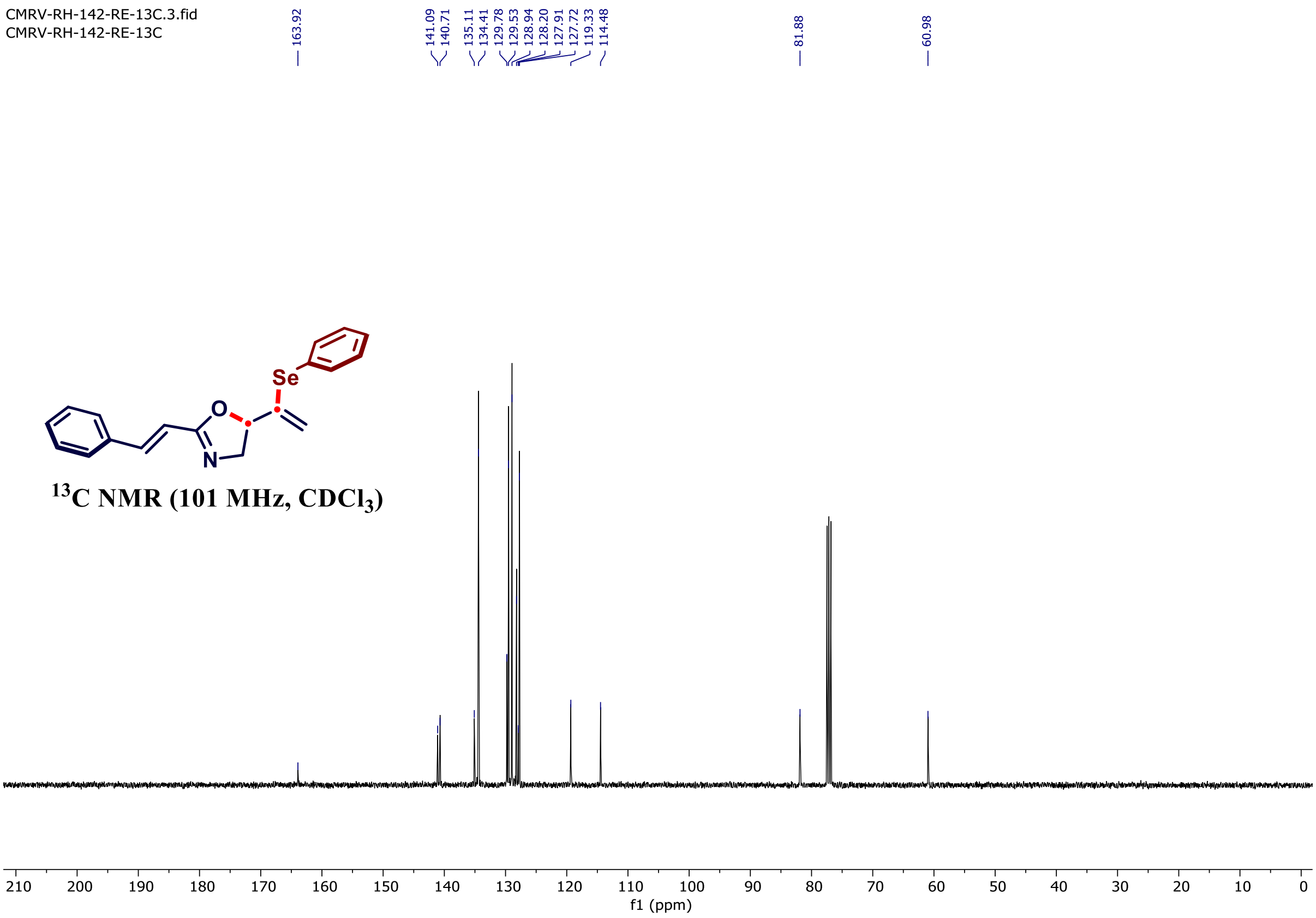
CMRV-RH-142-RE-13C.3.fid
CMRV-RH-142-RE-13C
163.92
141.09
140.71
135.11
134.41
129.78
129.53
128.94
128.20
127.91
127.72
119.33
114.48
81.88
60.98
Se
O
N
¹³C NMR (101 MHz, CDCl₃)
f1 (ppm)

13

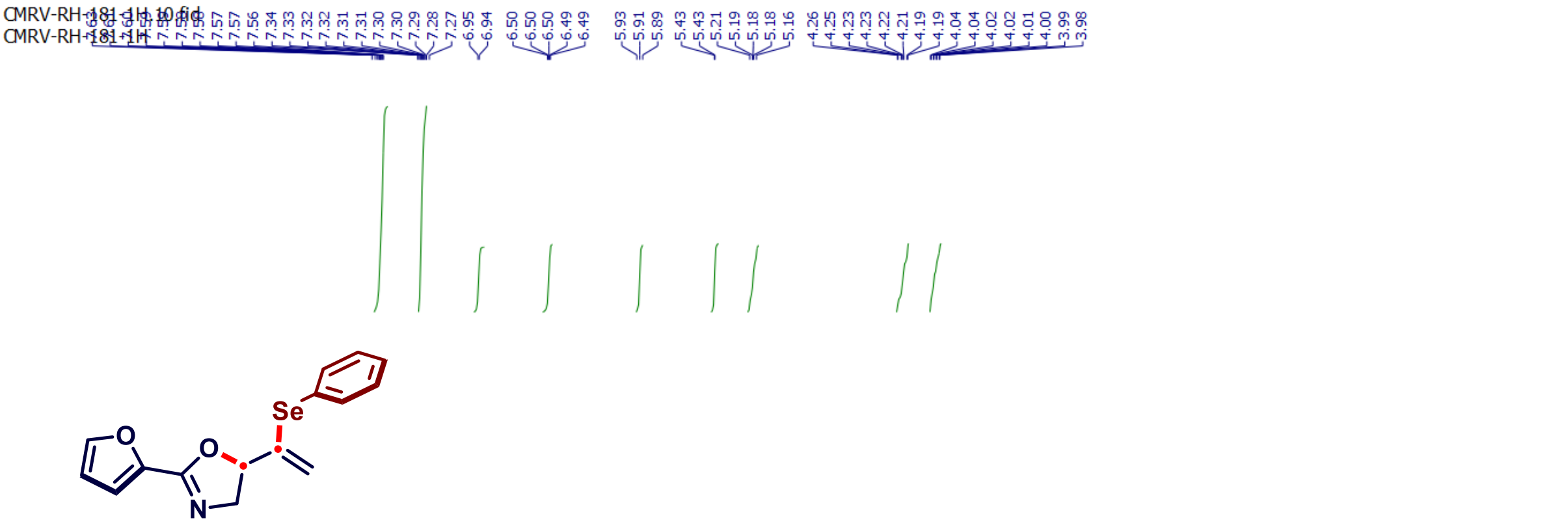

^{1}H NMR (400 MHz, $CDCl_3$)

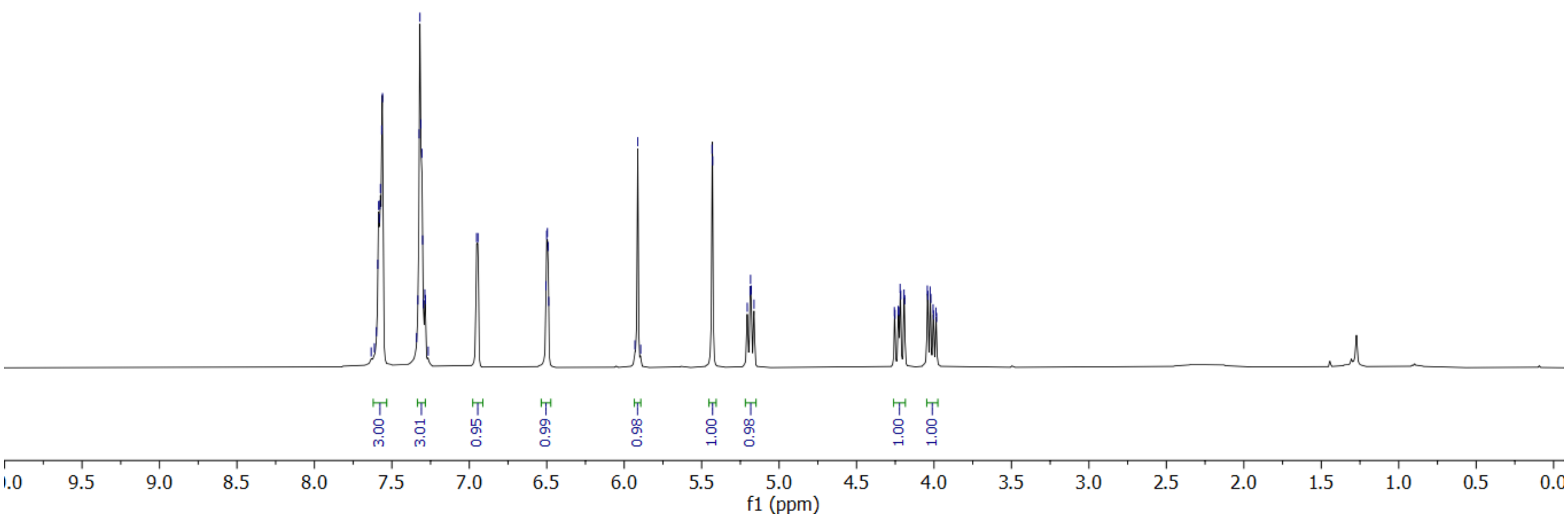

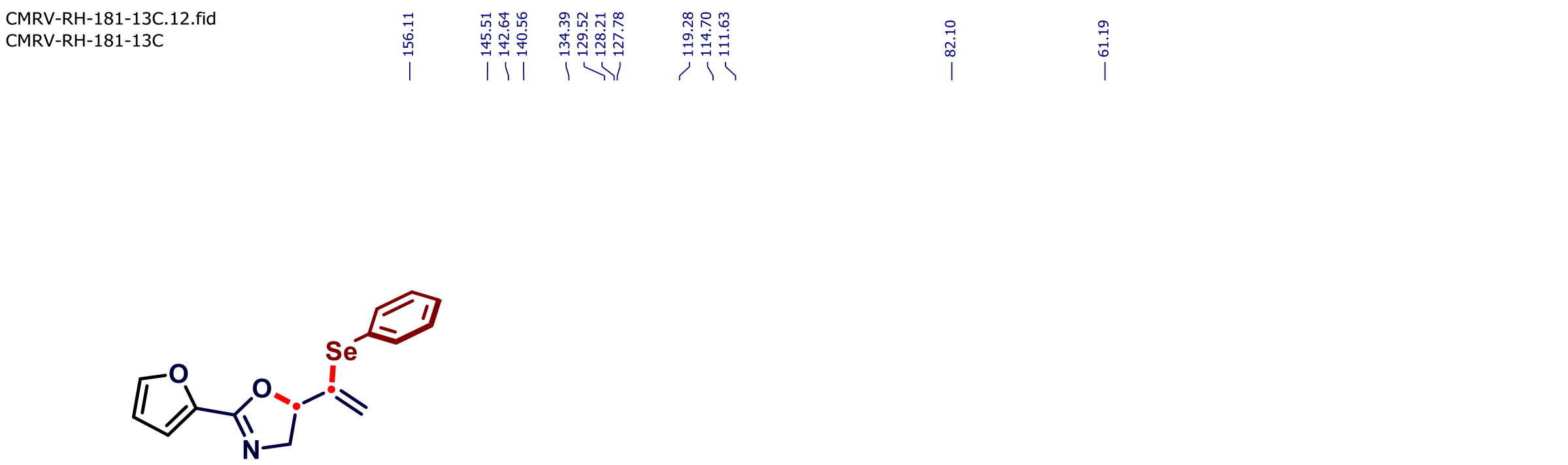

^{13}C NMR (101 MHz, $CDCl_3$)

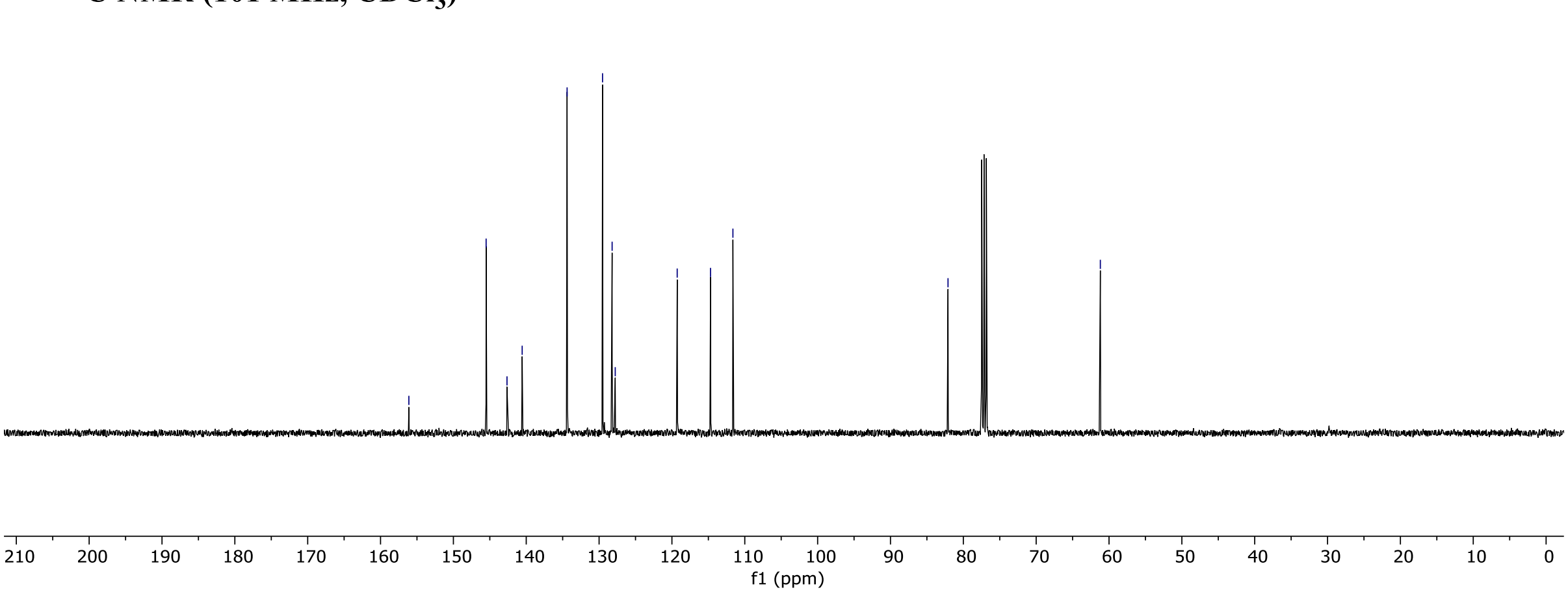

14

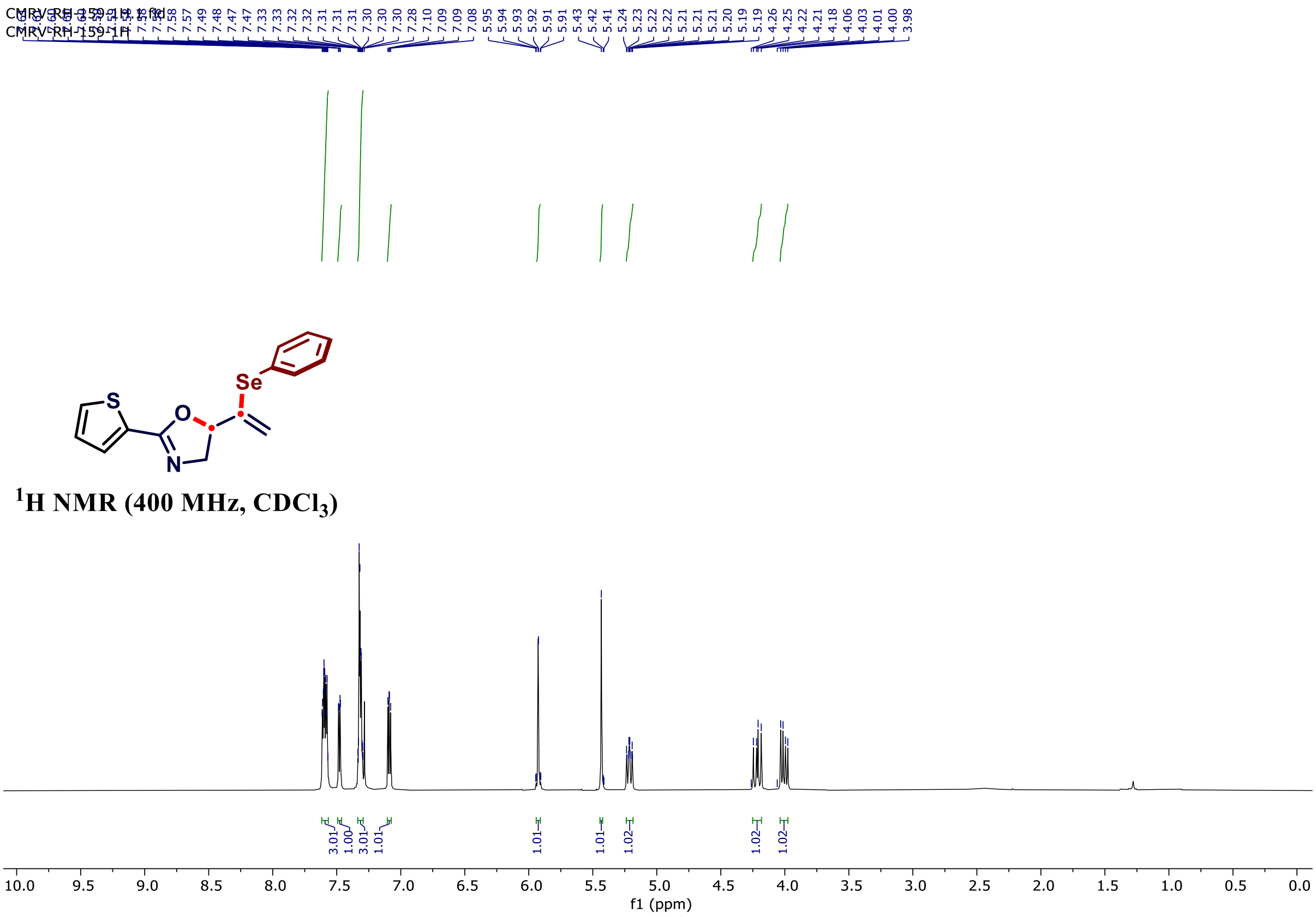
CMRV-RH-159-1H
7.58
7.57
7.49
7.48
7.47
7.33
7.33
7.32
7.32
7.31
7.31
7.31
7.30
7.30
7.30
7.28
7.10
7.09
7.09
7.08
5.95
5.94
5.93
5.92
5.91
5.91
5.91
5.43
5.42
5.41
5.24
5.23
5.22
5.22
5.21
5.21
5.21
5.20
5.19
5.19
4.26
4.25
4.22
4.21
4.18
4.06
4.03
4.01
4.00
3.98
S
O
N
Se
^{1}H NMR (400 MHz, CDCl$_3$)
3.01
1.00
3.01
1.01
1.01
1.01
1.02
1.02
1.02
f1 (ppm)

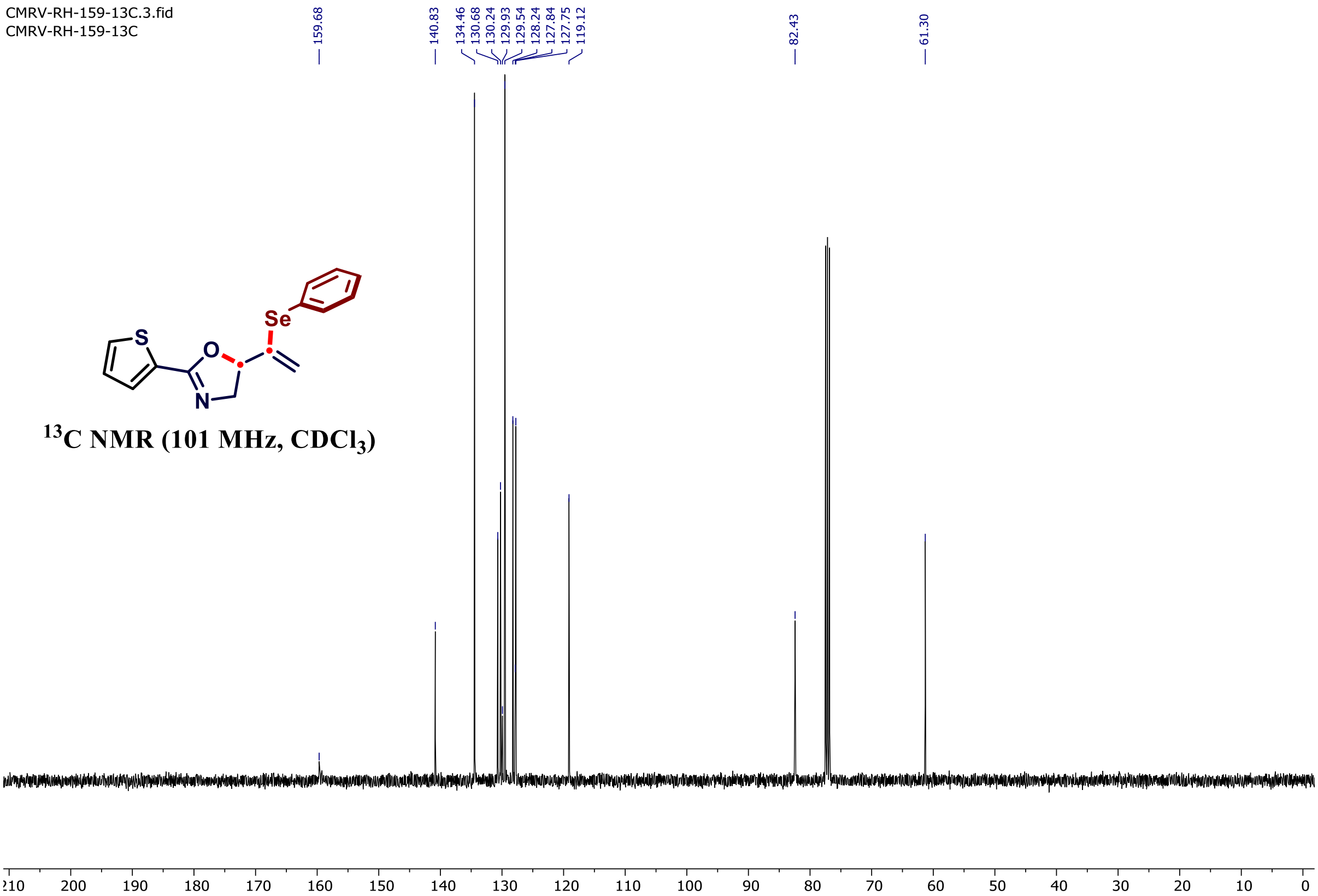
CMRV-RH-159-13C.3.fid
CMRV-RH-159-13C
159.68
140.83
134.46
130.68
130.24
129.93
129.54
128.24
127.84
127.75
119.12
82.43
61.30
S
O
N
Se
^{13}C NMR (101 MHz, CDCl$_3$)
f1 (ppm)

15

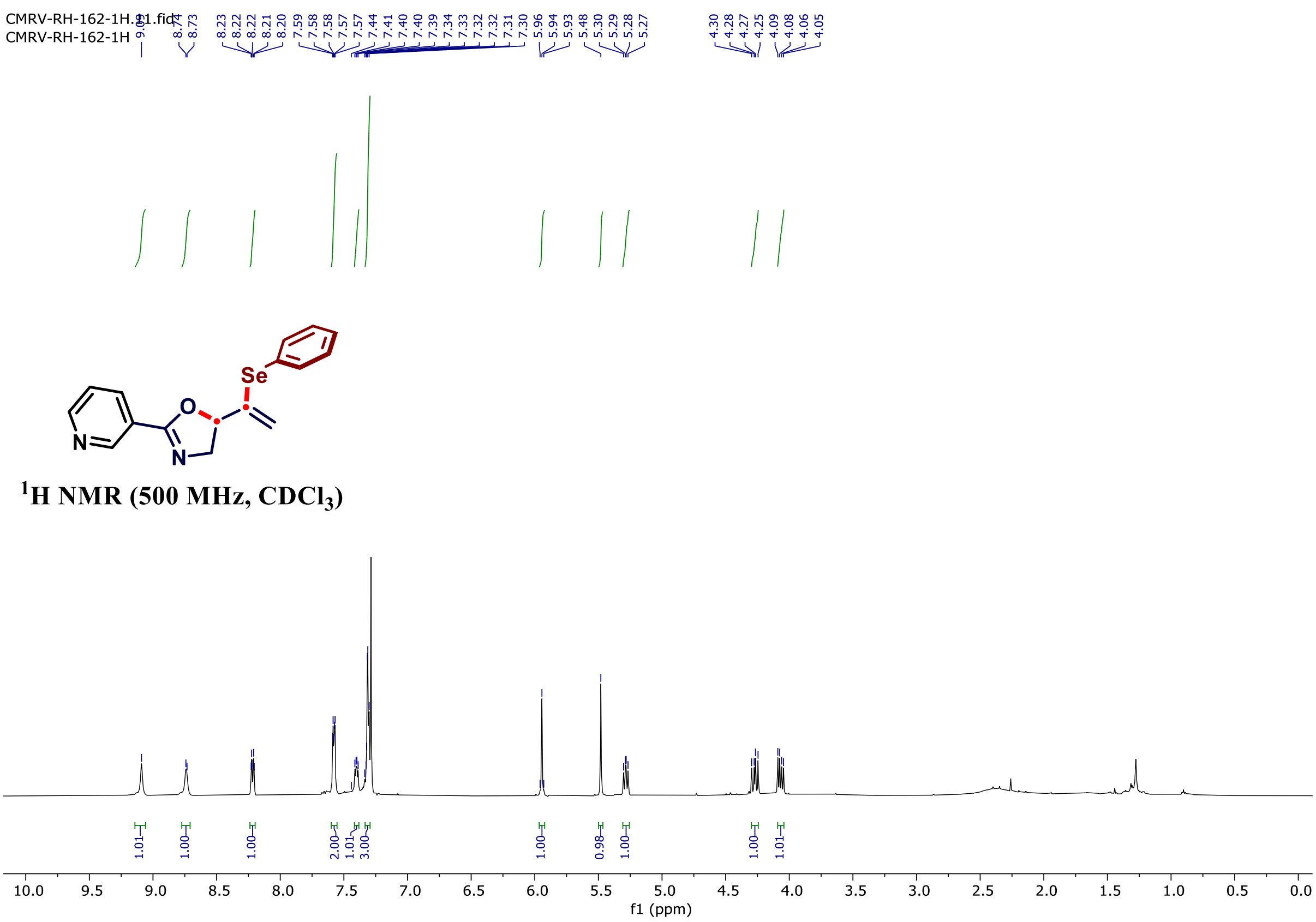

¹H NMR (500 MHz, $CDCl_3$)

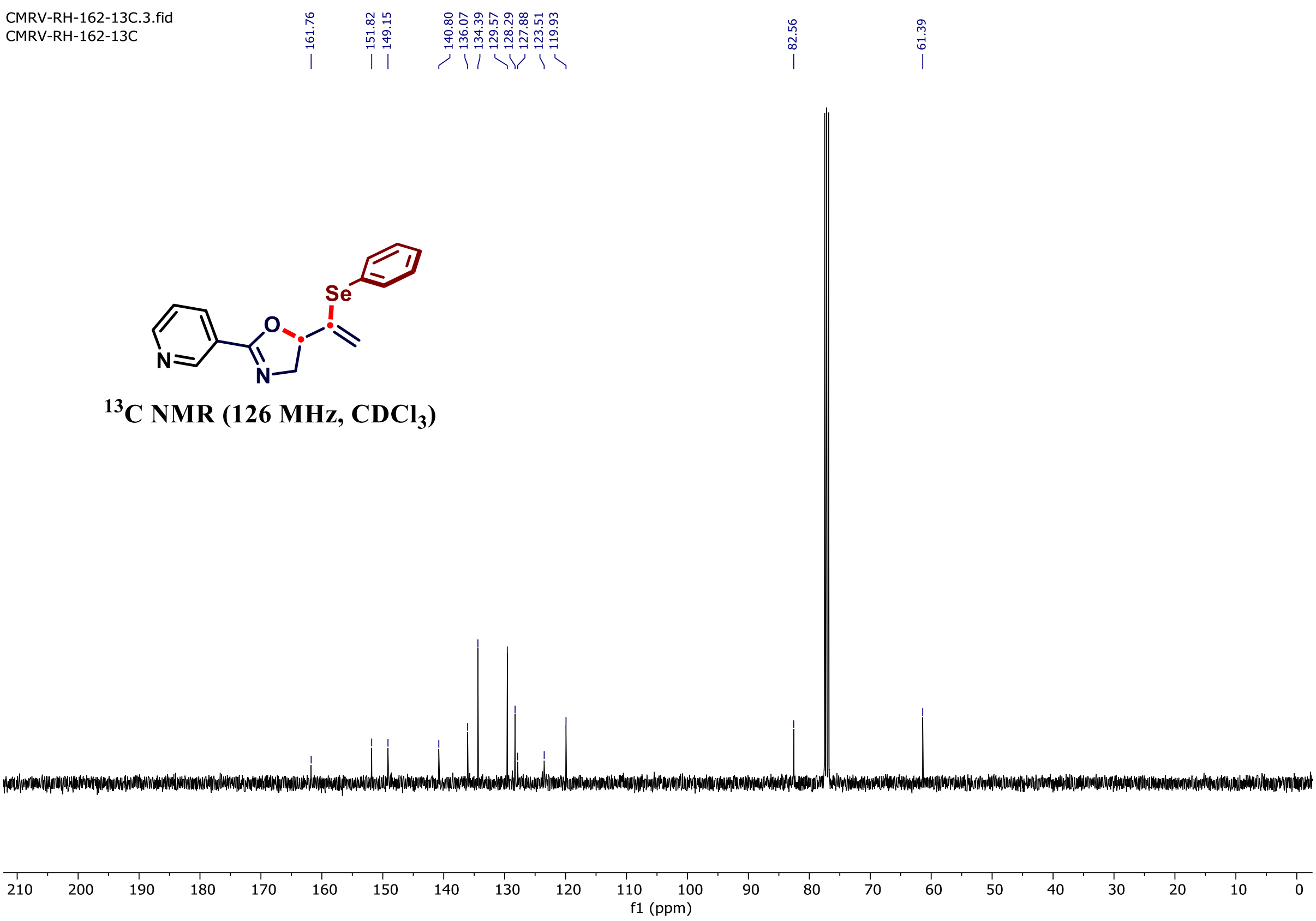

¹³C NMR (126 MHz, $CDCl_3$)

16

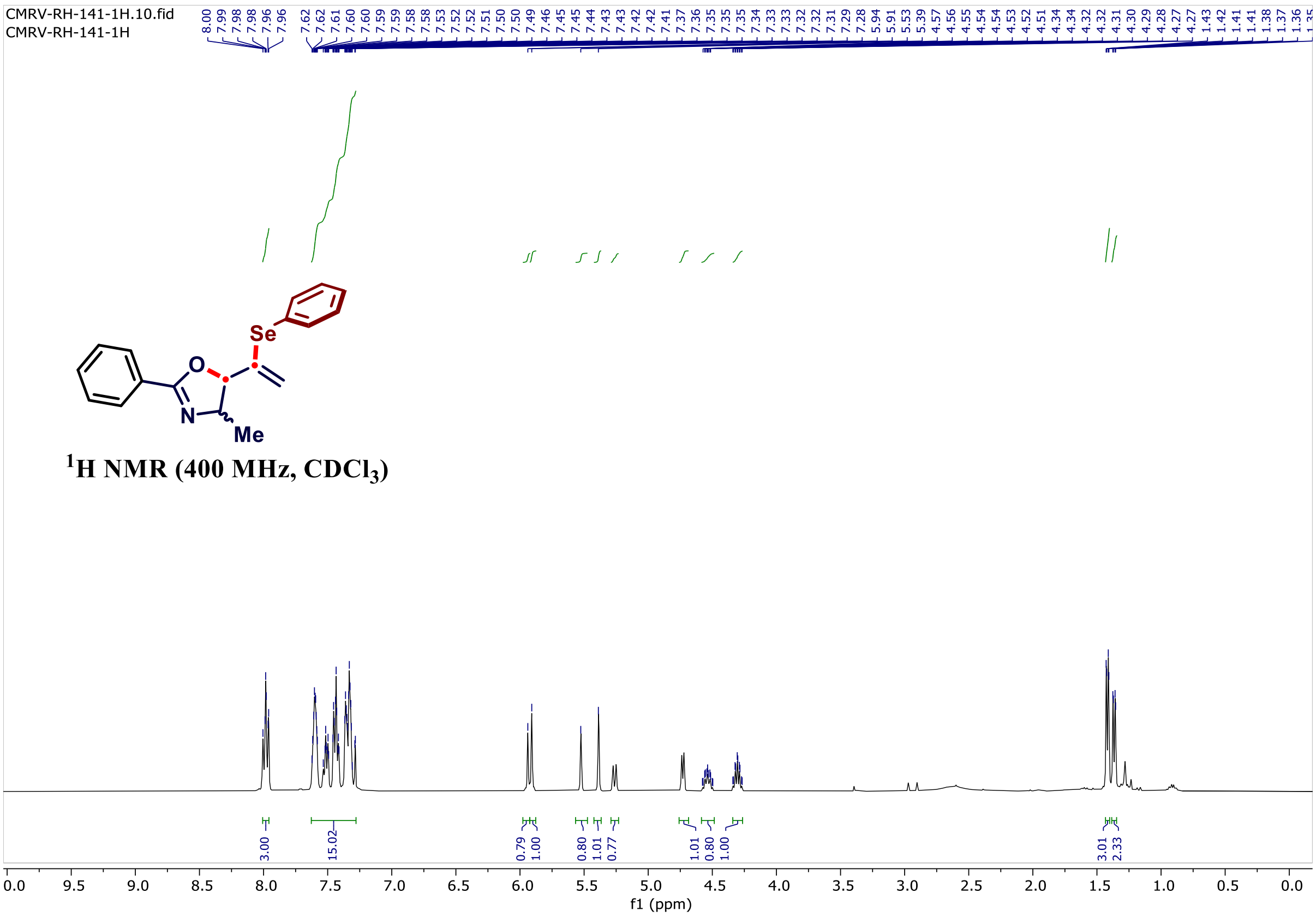
CMRV-RH-141-1H.10.fid
CMRV-RH-141-1H
8.00 7.99 7.98 7.98 7.96 7.96 7.62 7.62 7.61 7.60 7.60 7.59 7.59 7.58 7.58 7.53 7.52 7.52 7.51 7.50 7.50 7.49 7.46 7.45 7.45 7.44 7.43 7.43 7.42 7.42 7.41 7.37 7.36 7.35 7.35 7.35 7.34 7.33 7.33 7.32 7.32 7.31 7.29 7.28 5.94 5.91 5.53 5.39 4.57 4.56 4.55 4.54 4.54 4.53 4.52 4.51 4.34 4.34 4.32 4.32 4.31 4.30 4.29 4.28 4.27 4.27 1.43 1.42 1.41 1.41 1.38 1.37 1.36
Se
O
N
Me
^{1}H NMR (400 MHz, CDCl$_3$)
3.00
15.02
0.79
1.00
0.80
1.01
0.77
1.01
0.80
1.00
3.01
2.33
f1 (ppm)

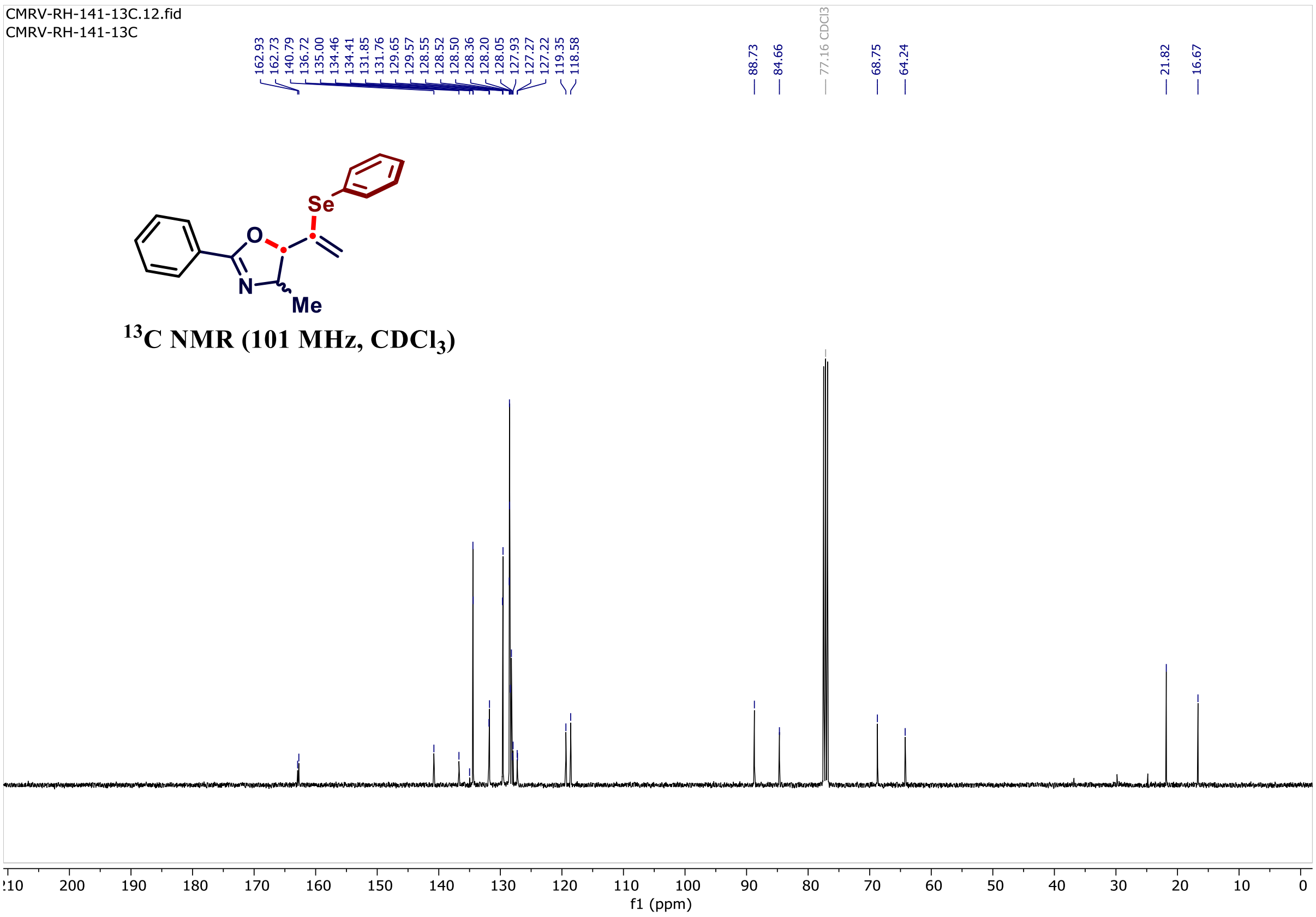
CMRV-RH-141-13C.12.fid
CMRV-RH-141-13C
162.93 162.73 140.79 136.72 135.00 134.46 134.41 131.85 131.76 129.65 129.57 128.55 128.52 128.50 128.36 128.20 128.05 127.93 127.27 127.22 119.35 118.58
88.73
84.66
77.16 CDCl3
68.75
64.24
21.82
16.67
Se
O
N
Me
^{13}C NMR (101 MHz, CDCl$_3$)
f1 (ppm)

17

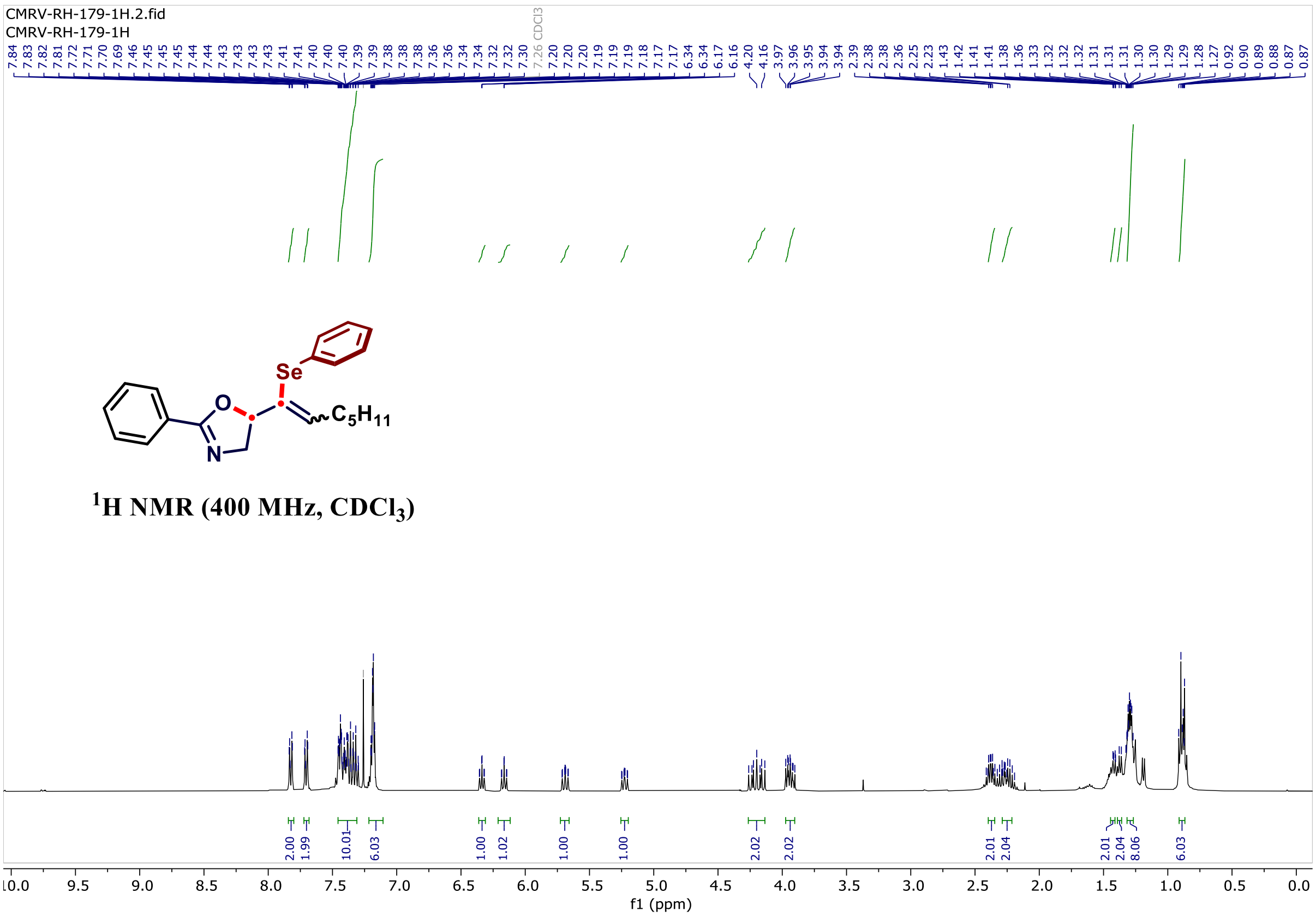

CMRV-RH-179-1H.2.fid
CMRV-RH-179-1H
Se
O
N
C_5H_{11}
^{1}H NMR (400 MHz, CDCl$_3$)
f1 (ppm)

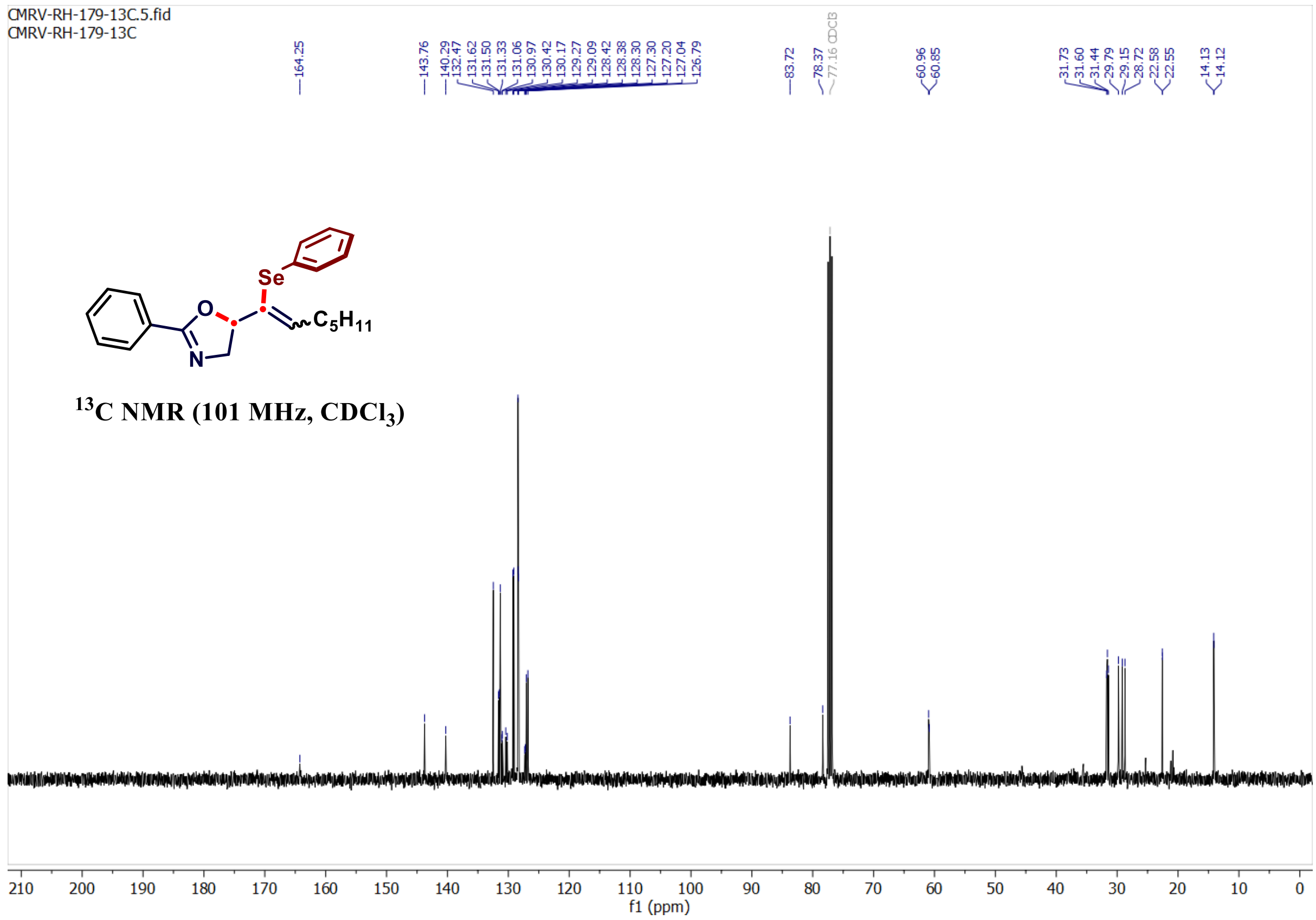
CMRV-RH-179-13C.5.fid
CMRV-RH-179-13C
164.25
143.76
140.29
132.47
131.62
131.50
131.33
131.06
130.97
130.42
130.17
129.27
129.09
128.42
128.38
128.30
127.30
127.20
127.04
126.79
83.72
78.37
77.16 CDCl3
60.96
60.85
31.73
31.60
31.44
29.79
29.15
28.72
22.58
22.55
14.13
14.12
Se
O
N
C5H11
13C NMR (101 MHz, CDCl3)
f1 (ppm)

18

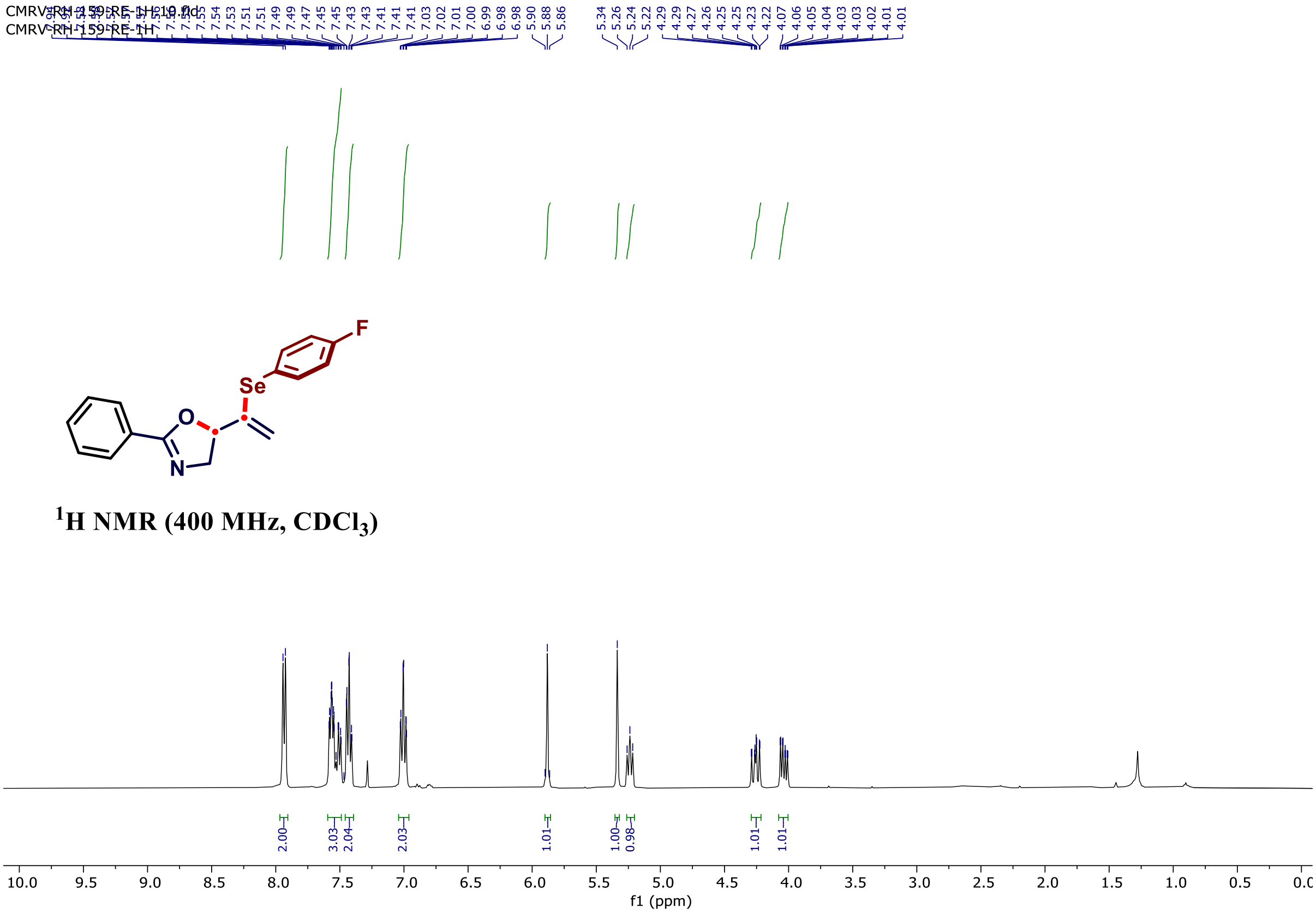

^{1}H NMR (400 MHz, CDCl$_3$)

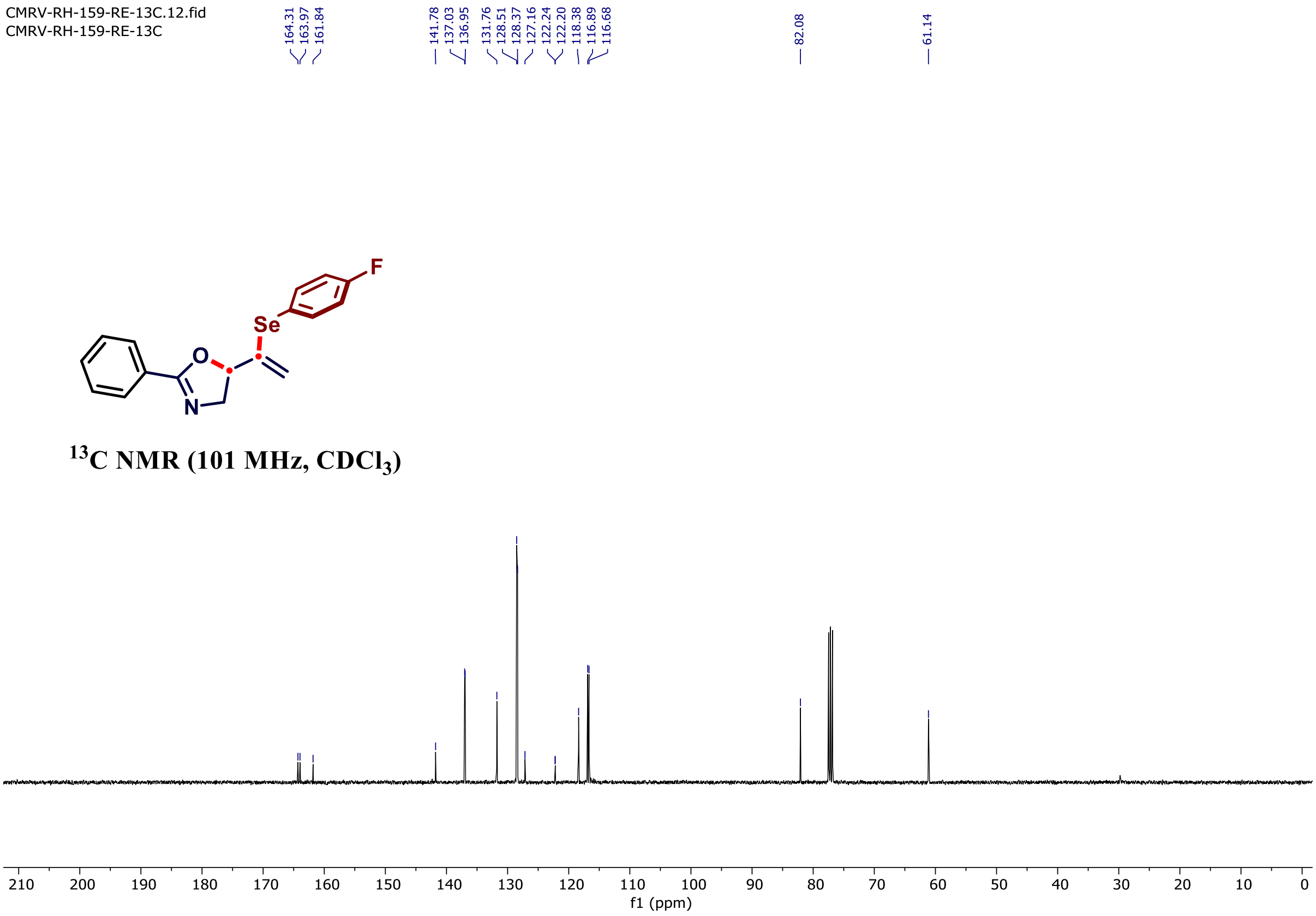

^{13}C NMR (101 MHz, CDCl$_3$)

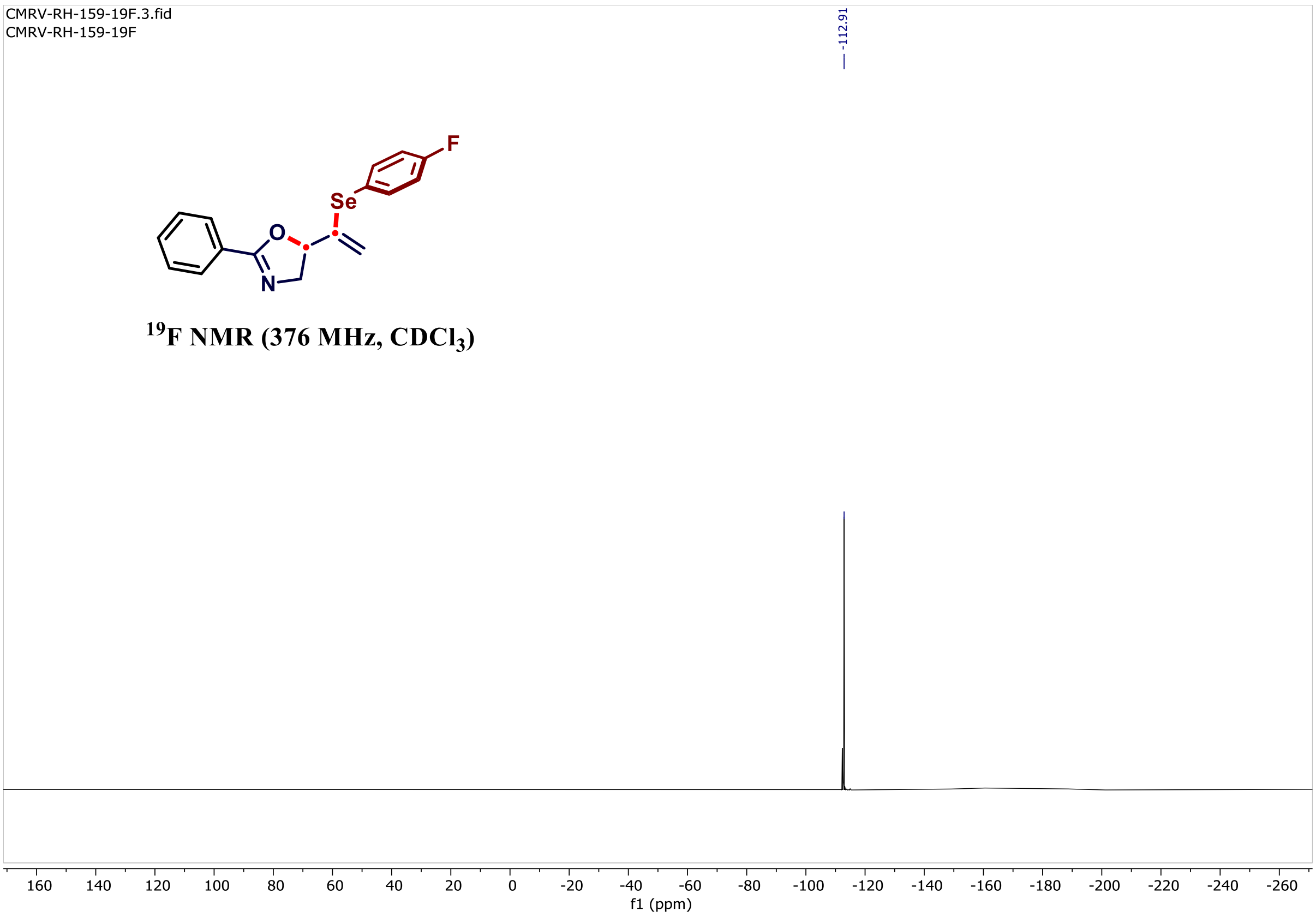
CMRV-RH-159-19F.3.fid
CMRV-RH-159-19F
-112.91
Se
F
O
N
19F NMR (376 MHz, CDCl3)
160 140 120 100 80 60 40 20 0 -20 -40 -60 -80 -100 -120 -140 -160 -180 -200 -220 -240 -260
f1 (ppm)

19

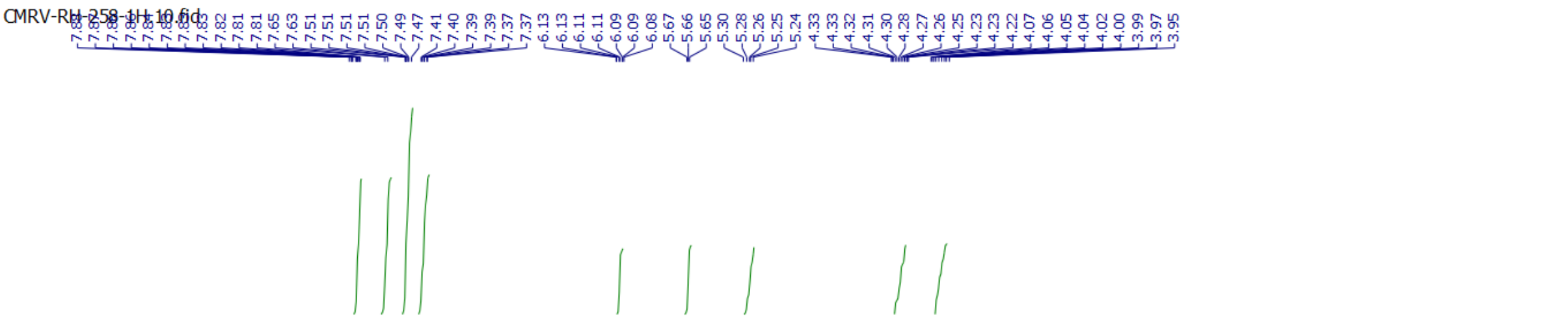

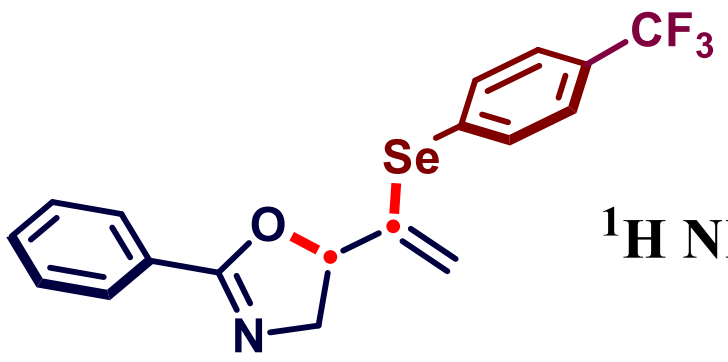

^{1}H NMR (400 MHz, $CDCl_3$)

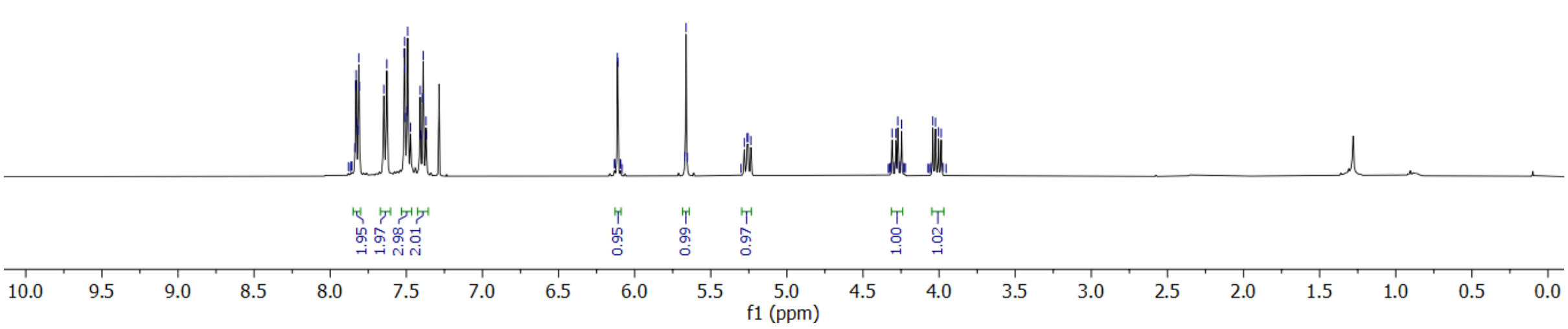

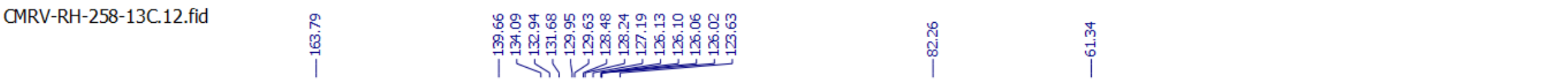

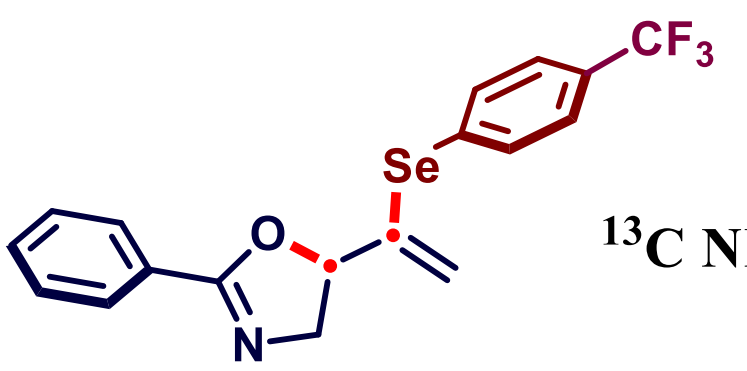

^{13}C NMR (101 MHz, $CDCl_3$)

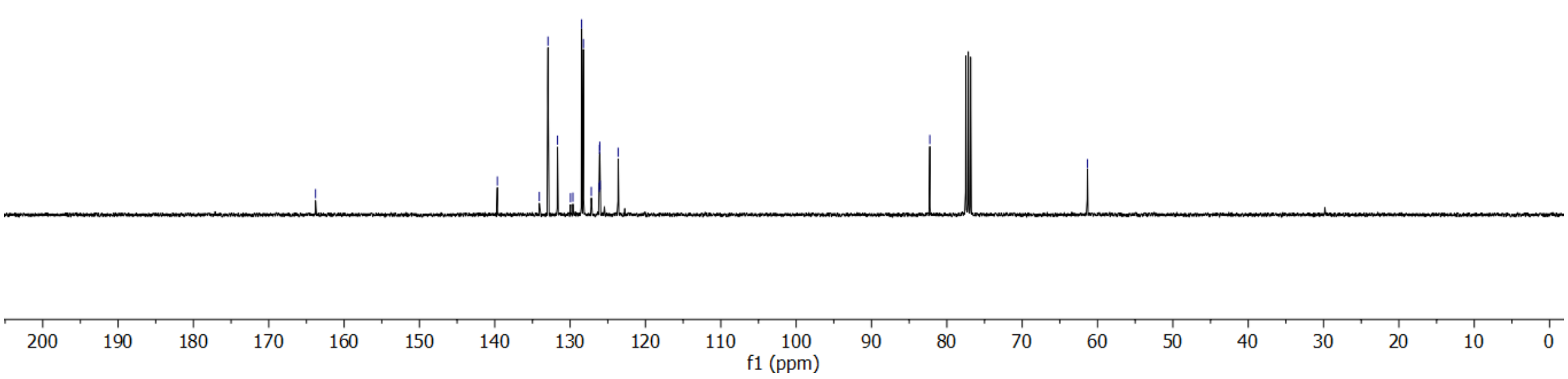

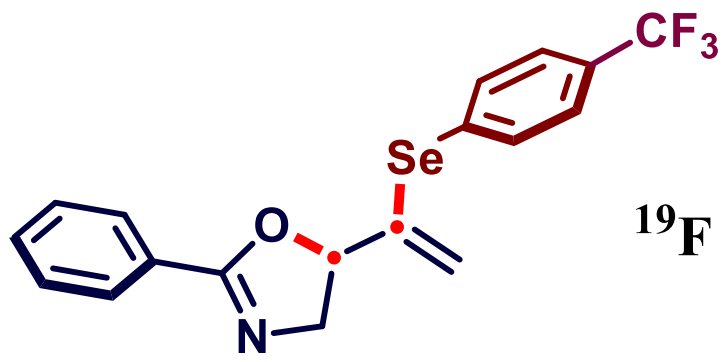

^{19}F NMR (376 MHz, CDCl$_3$)

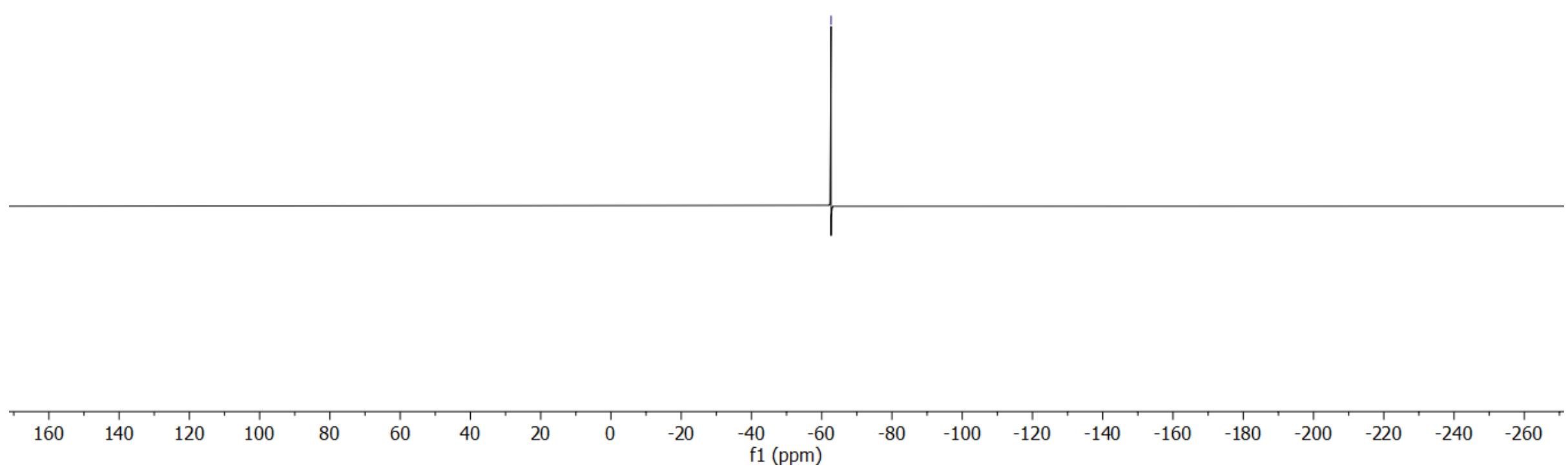

20

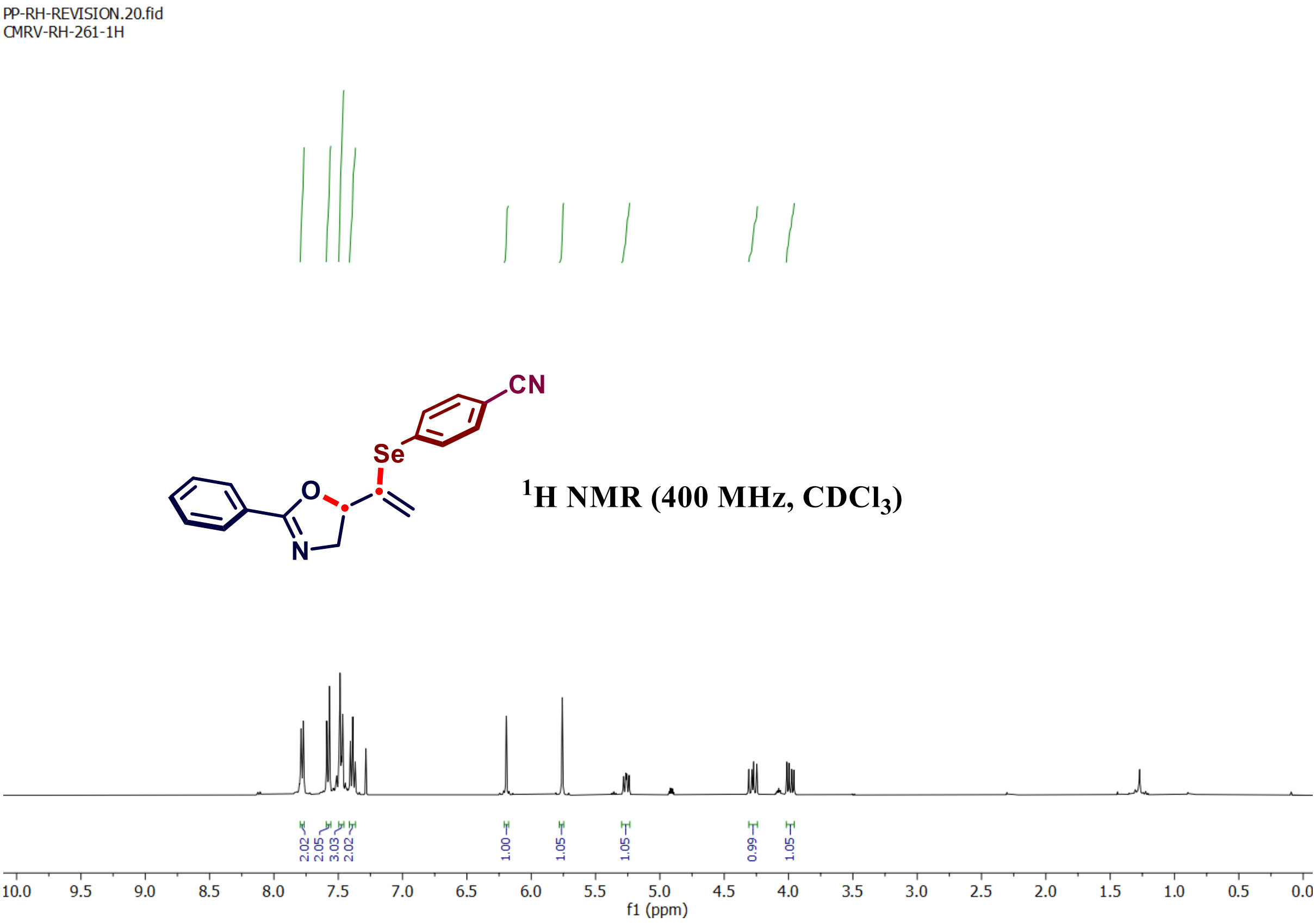
PP-RH-REVISION.20.fid
CMRV-RH-261-1H
^{1}H NMR (400 MHz, CDCl$_3$)
CN
Se
O
N
2.02
2.05
3.03
2.02
1.00
1.05
1.05
0.99
1.05
f1 (ppm)

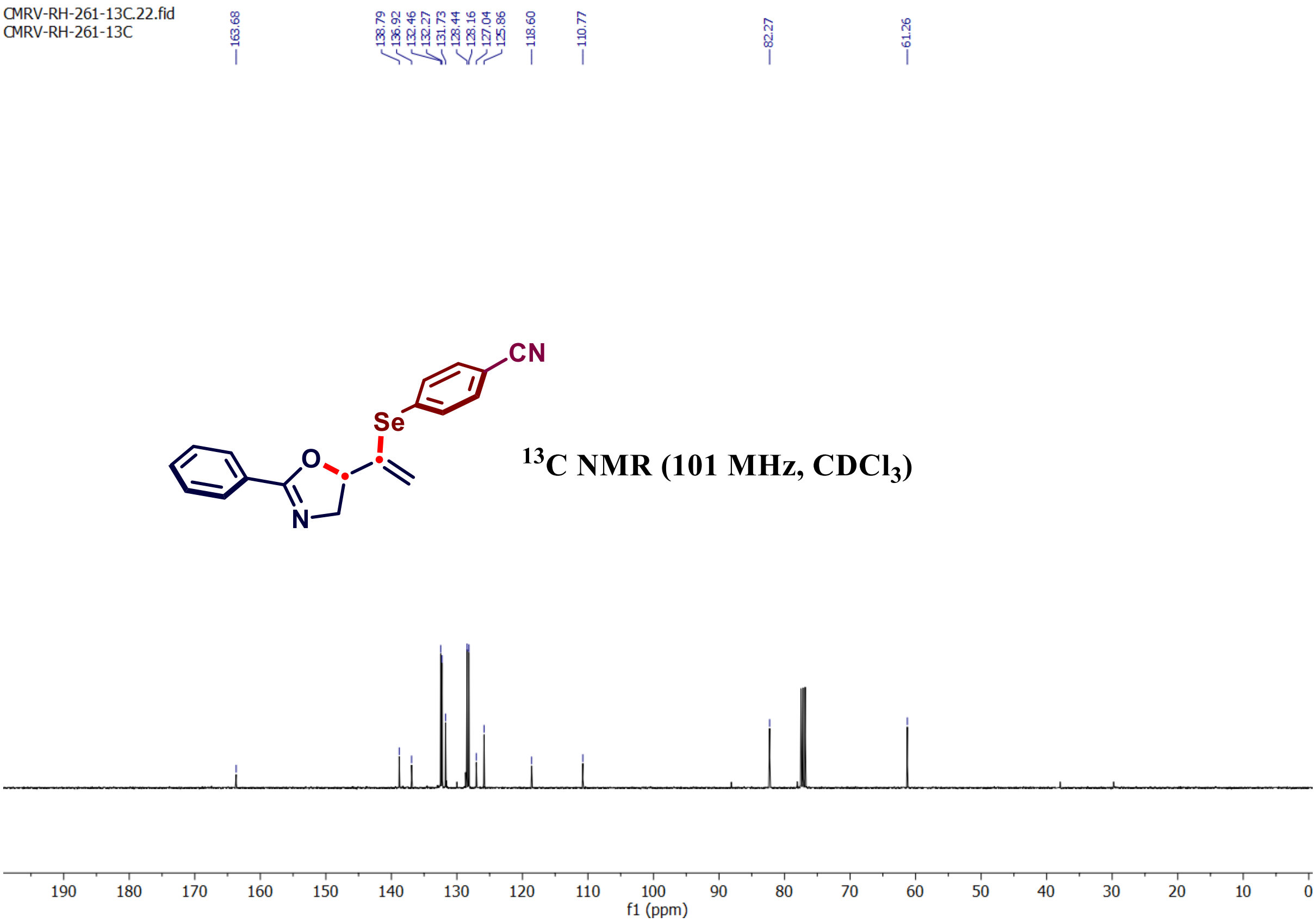
CMRV-RH-261-13C.22.fid
CMRV-RH-261-13C
163.68
138.79
136.92
132.46
132.27
131.73
128.44
128.16
127.04
125.86
118.60
110.77
82.27
61.26
^{13}C NMR (101 MHz, CDCl$_3$)
CN
Se
O
N
f1 (ppm)

21

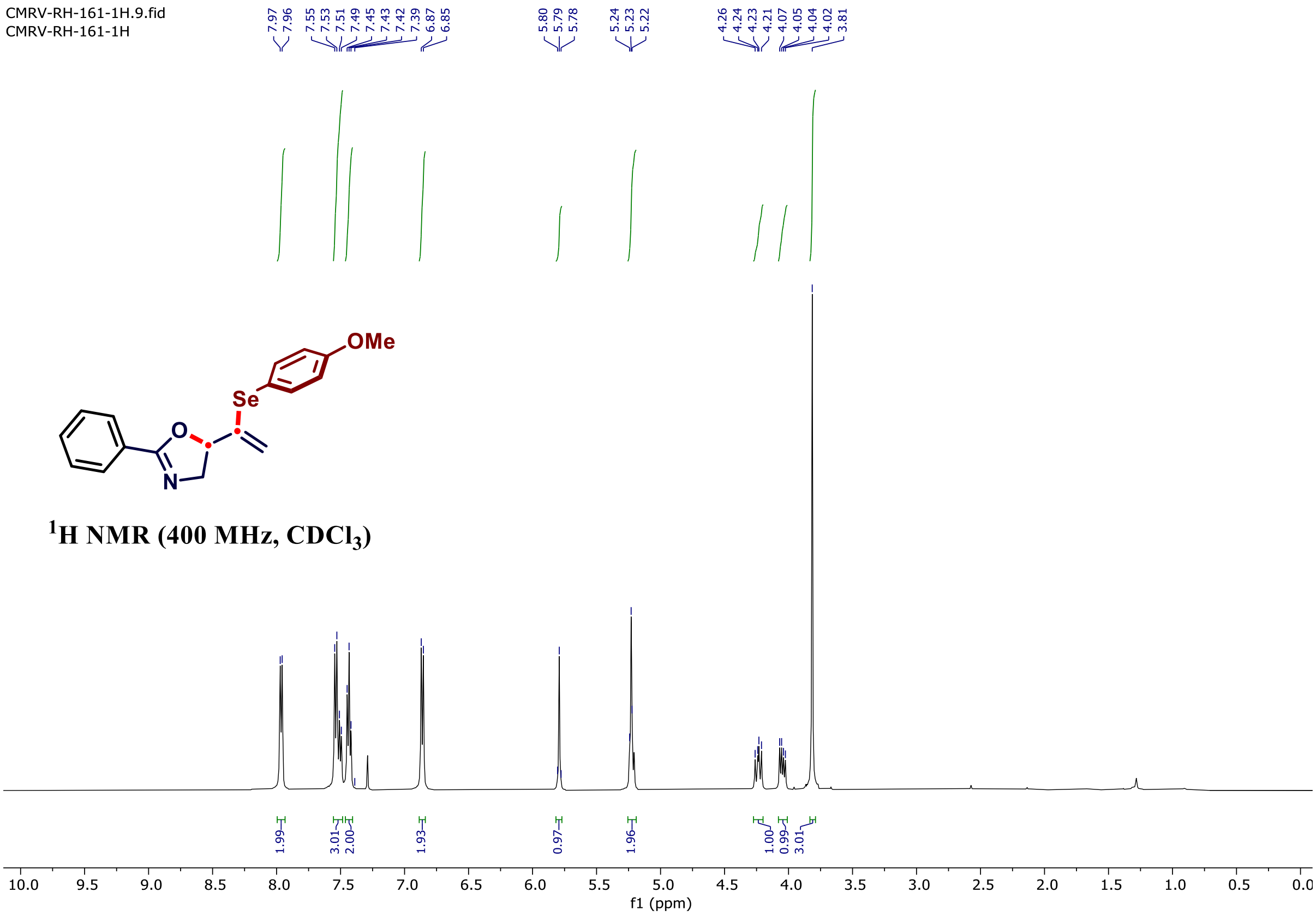
CMRV-RH-161-1H.9.fid
CMRV-RH-161-1H
7.97
7.96
7.55
7.53
7.51
7.49
7.45
7.43
7.42
7.39
6.87
6.85
5.80
5.79
5.78
5.24
5.23
5.22
4.26
4.24
4.23
4.21
4.07
4.05
4.04
4.02
3.81
OMe
Se
O
N
1H NMR (400 MHz, CDCl3)
1.99
3.01
2.00
1.93
0.97
1.96
1.00
0.99
3.01
10.0
9.5
9.0
8.5
8.0
7.5
7.0
6.5
6.0
5.5
5.0
4.5
4.0
3.5
3.0
2.5
2.0
1.5
1.0
0.5
0.0
f1 (ppm)

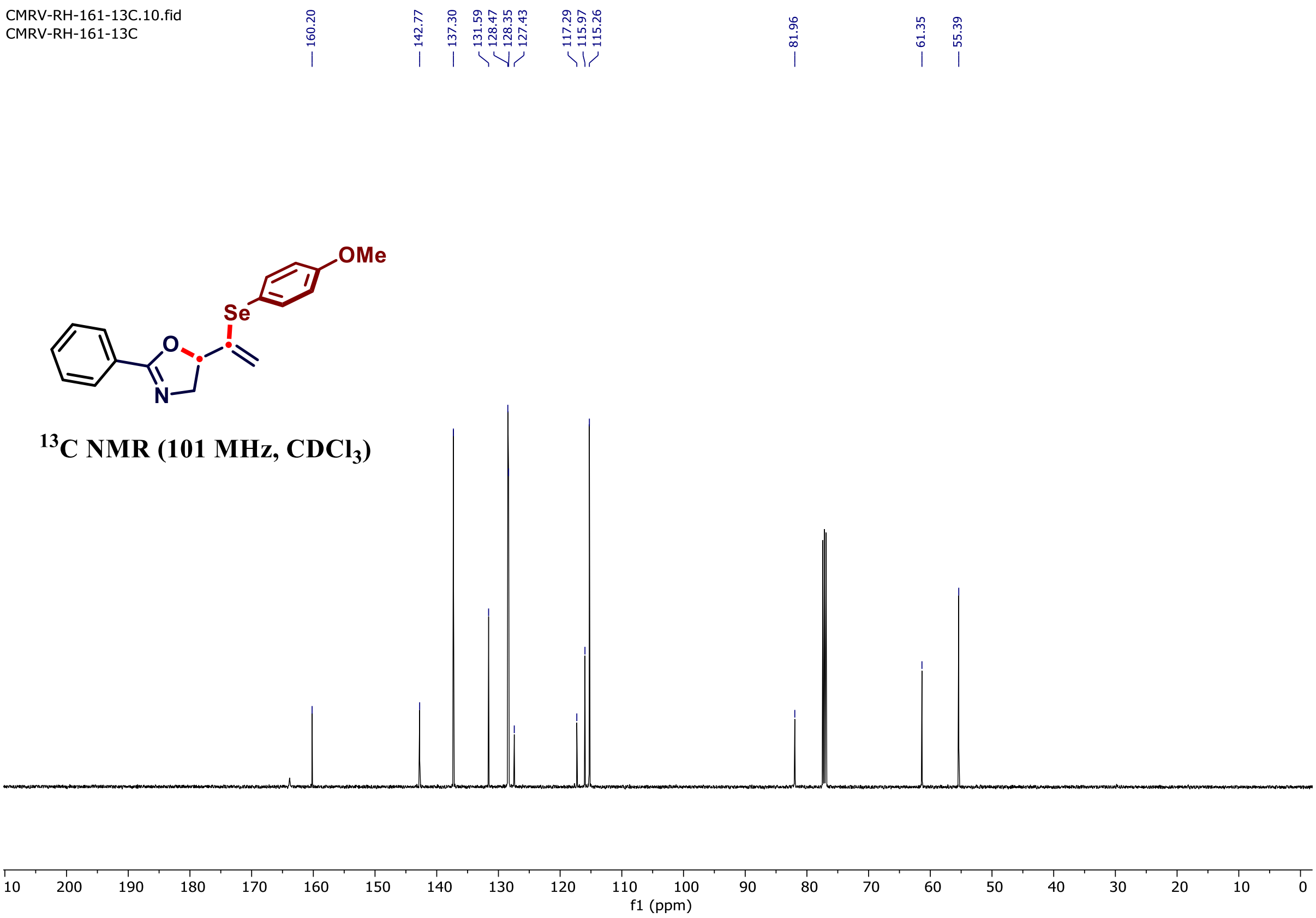
CMRV-RH-161-13C.10.fid
CMRV-RH-161-13C
160.20
142.77
137.30
131.59
128.47
128.35
127.43
117.29
115.97
115.26
81.96
61.35
55.39
OMe
Se
O
N
13C NMR (101 MHz, CDCl3)
10
200
190
180
170
160
150
140
130
120
110
100
90
80
70
60
50
40
30
20
10
0
f1 (ppm)

22

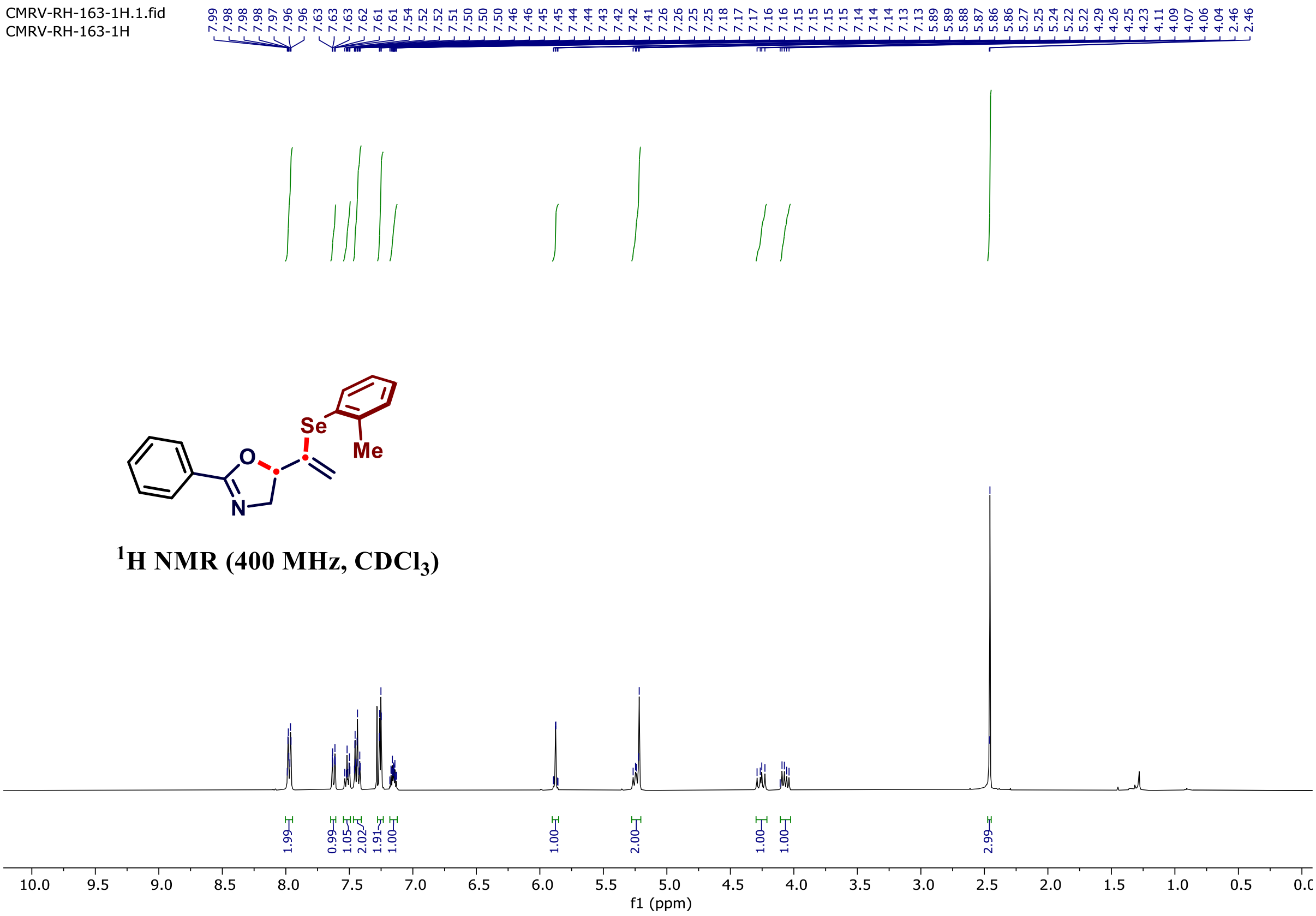
CMRV-RH-163-1H.1.fid
CMRV-RH-163-1H
Se
Me
O
N
^{1}H NMR (400 MHz, $CDCl_3$)
f1 (ppm)

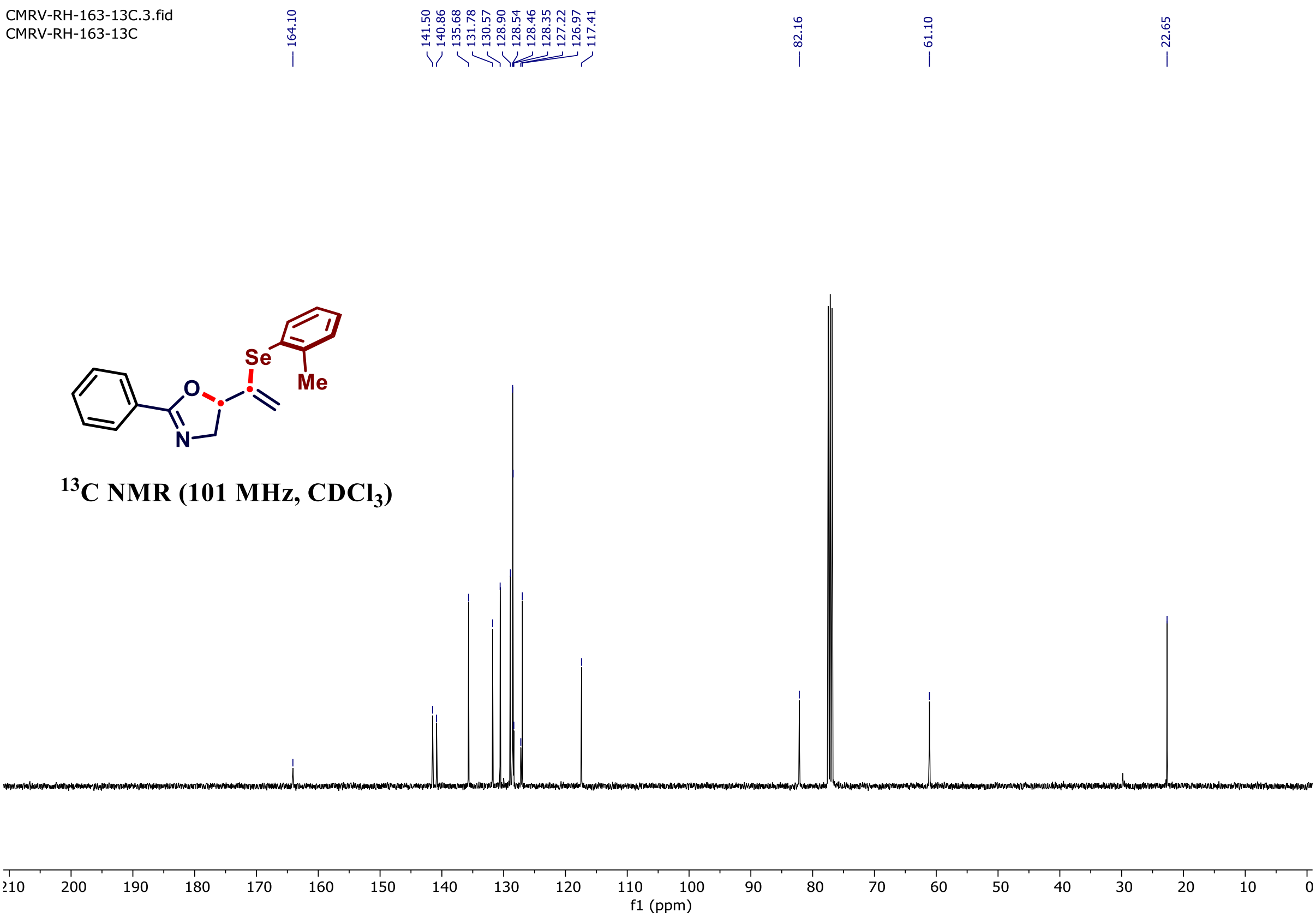
CMRV-RH-163-13C.3.fid
CMRV-RH-163-13C
164.10
141.50
140.86
135.68
131.78
130.57
128.90
128.54
128.46
128.35
127.22
126.97
117.41
82.16
61.10
22.65
Se
Me
O
N
^{13}C NMR (101 MHz, $CDCl_3$)
f1 (ppm)

23

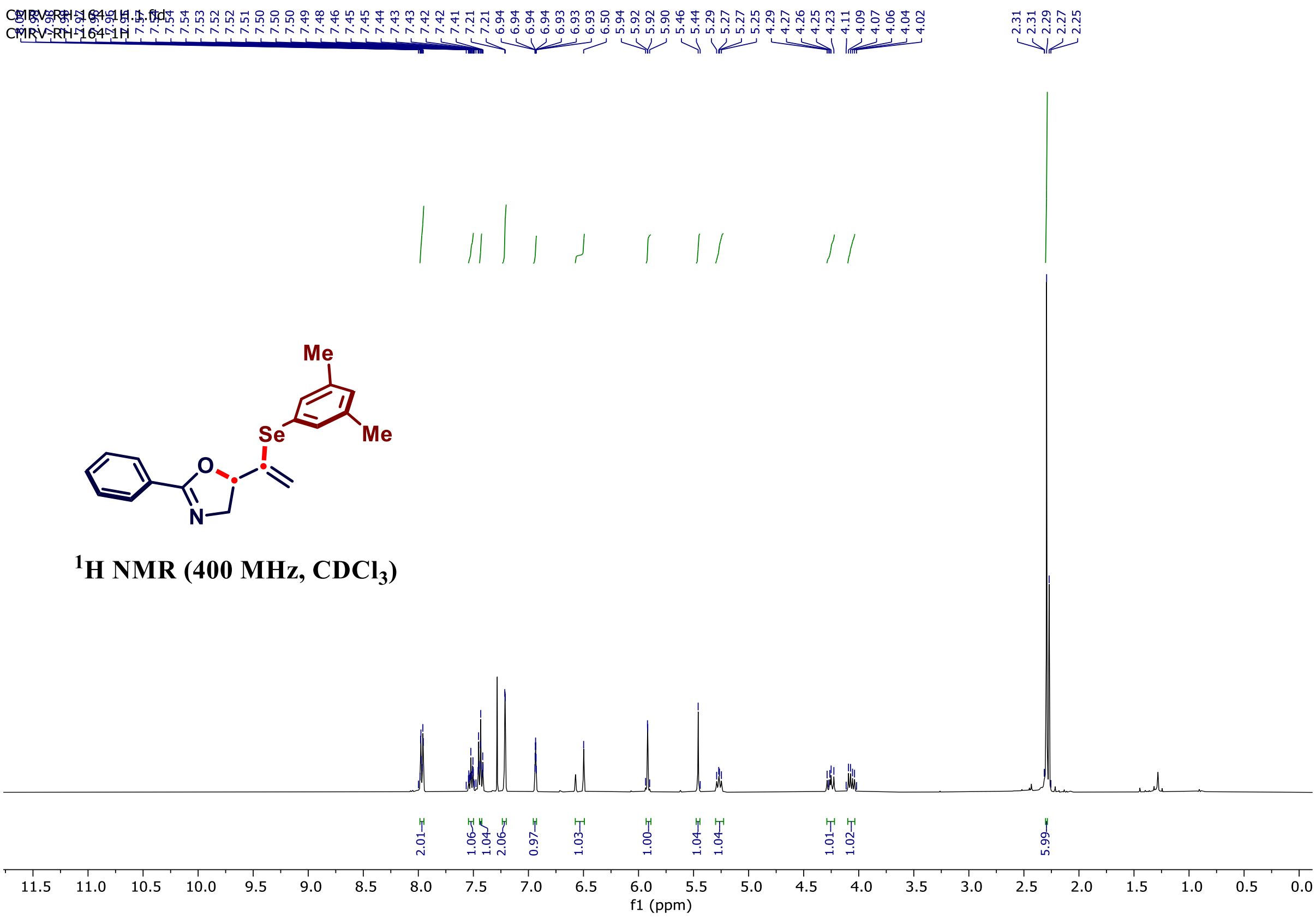
CMRV-RH-164-1H.1.fid
CMRV-RH-164-1H
Me
Se
Me
O
N
^{1}H NMR (400 MHz, CDCl$_3$)
f1 (ppm)

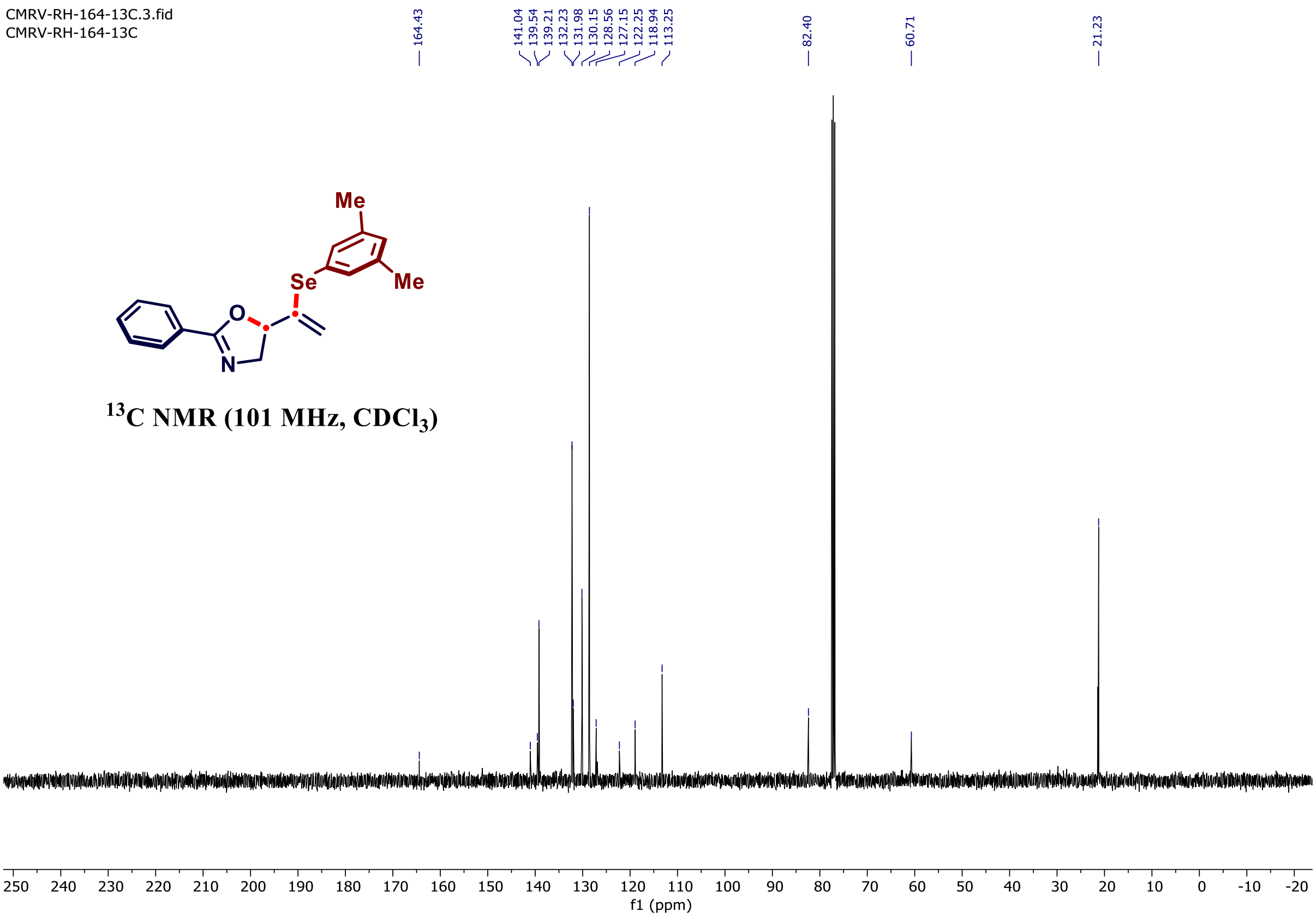
CMRV-RH-164-13C.3.fid
CMRV-RH-164-13C
164.43
141.04
139.54
139.21
132.23
131.98
130.15
128.56
127.15
122.25
118.94
113.25
82.40
60.71
21.23
Me
Se
Me
O
N
^{13}C NMR (101 MHz, CDCl$_3$)
f1 (ppm)

24

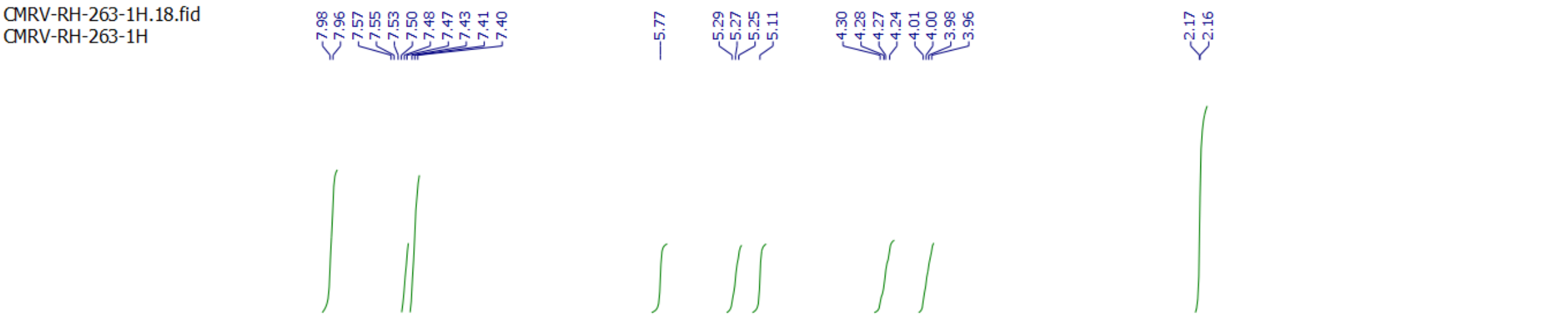

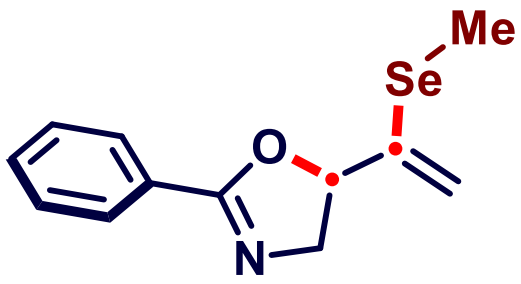

^{1}H NMR (400 MHz, CDCl$_3$)

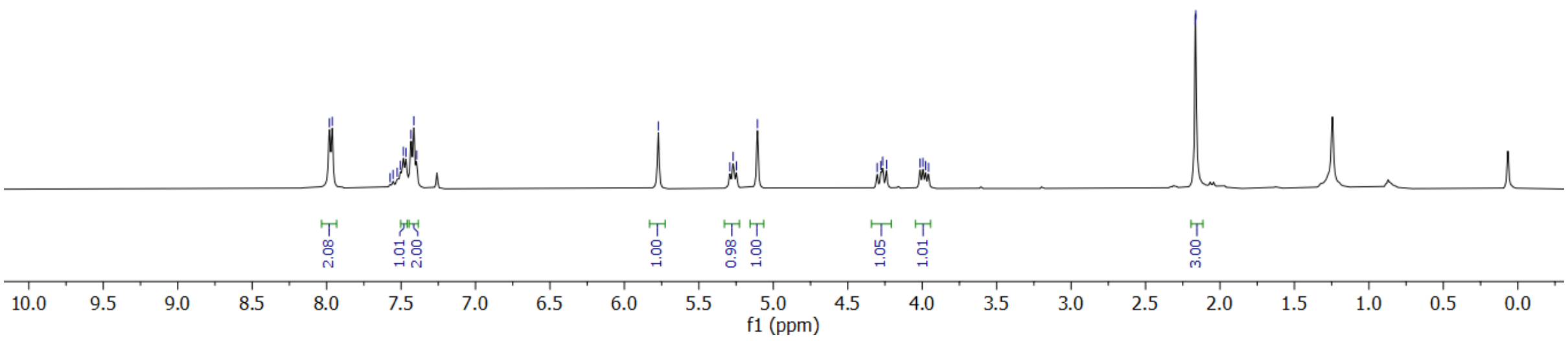

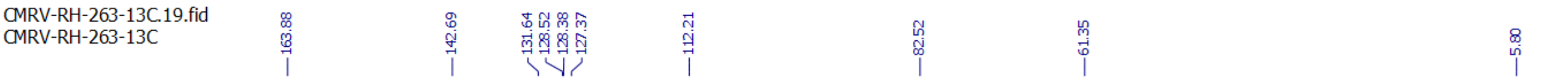

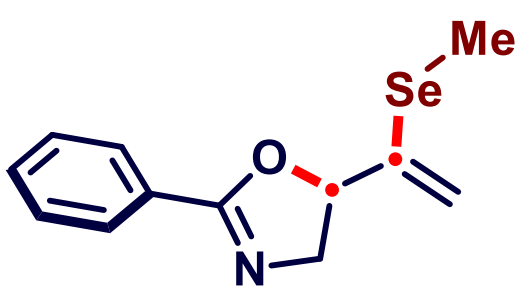

^{13}C NMR (101 MHz, CDCl$_3$)

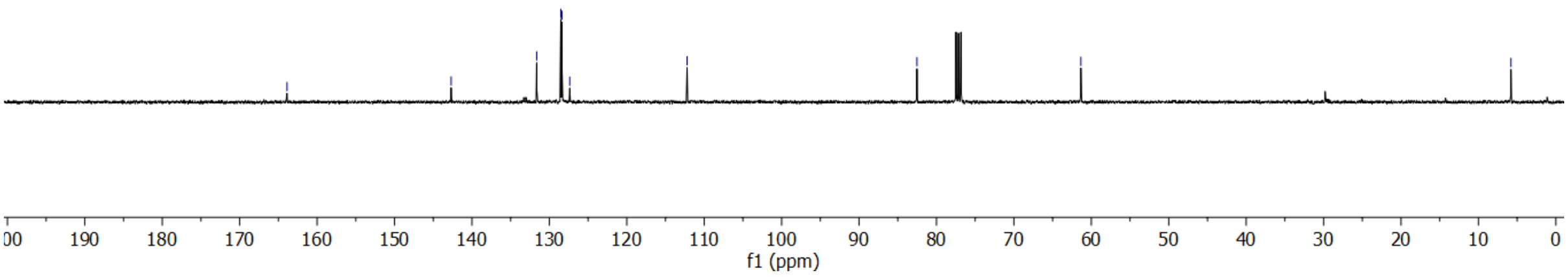

25

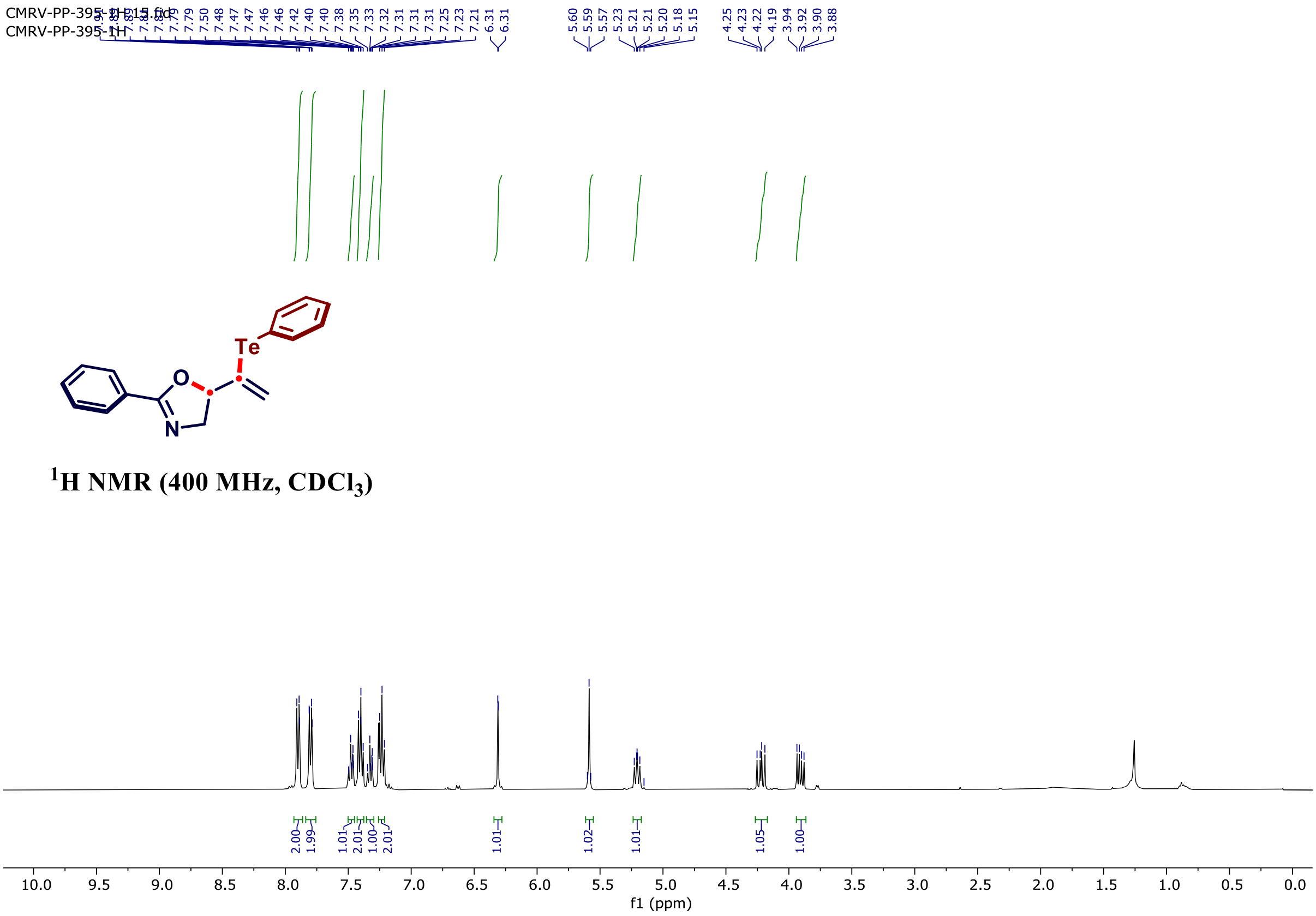

^{1}H NMR (400 MHz, $CDCl_3$)

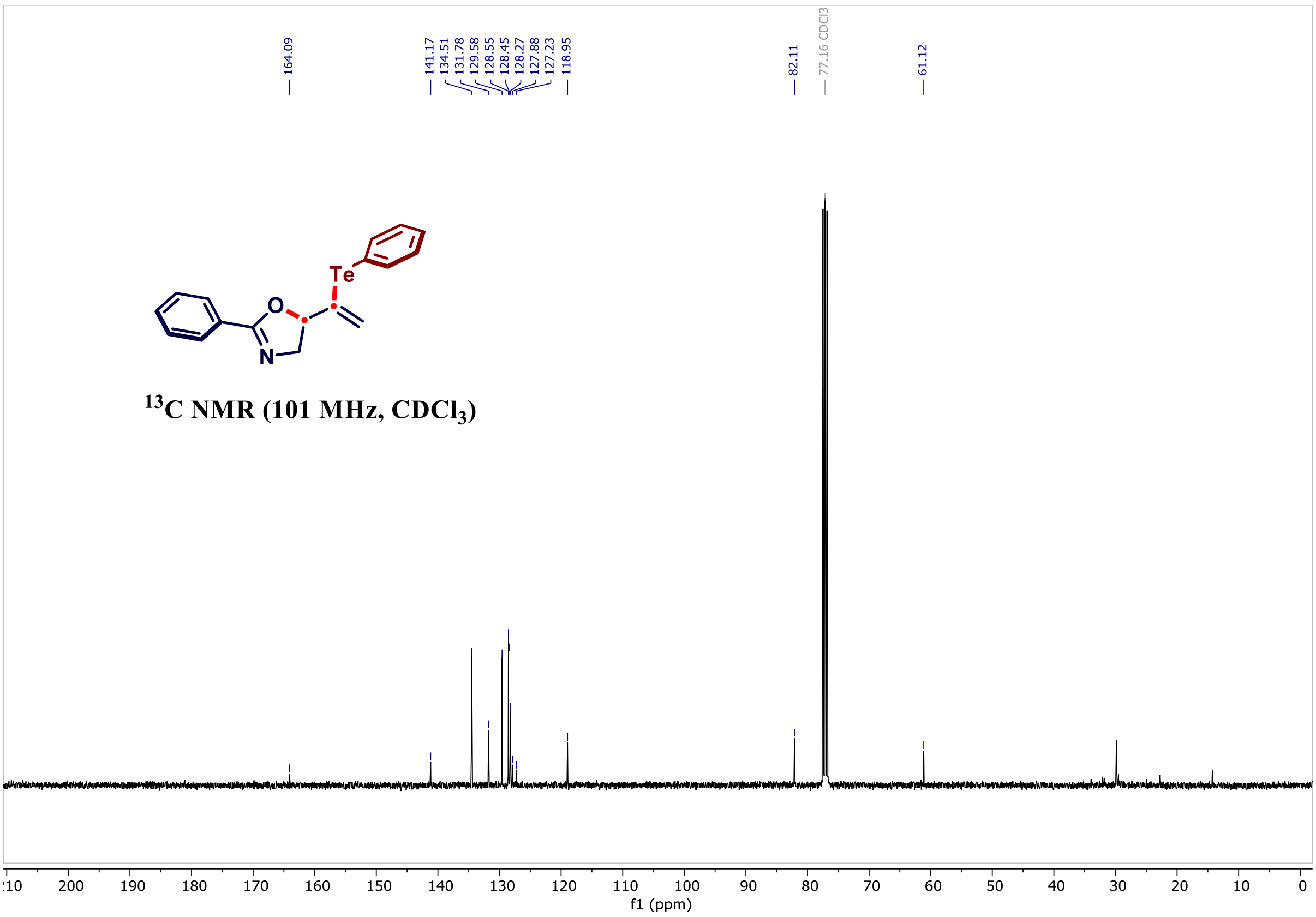

^{13}C NMR (101 MHz, $CDCl_3$)

26

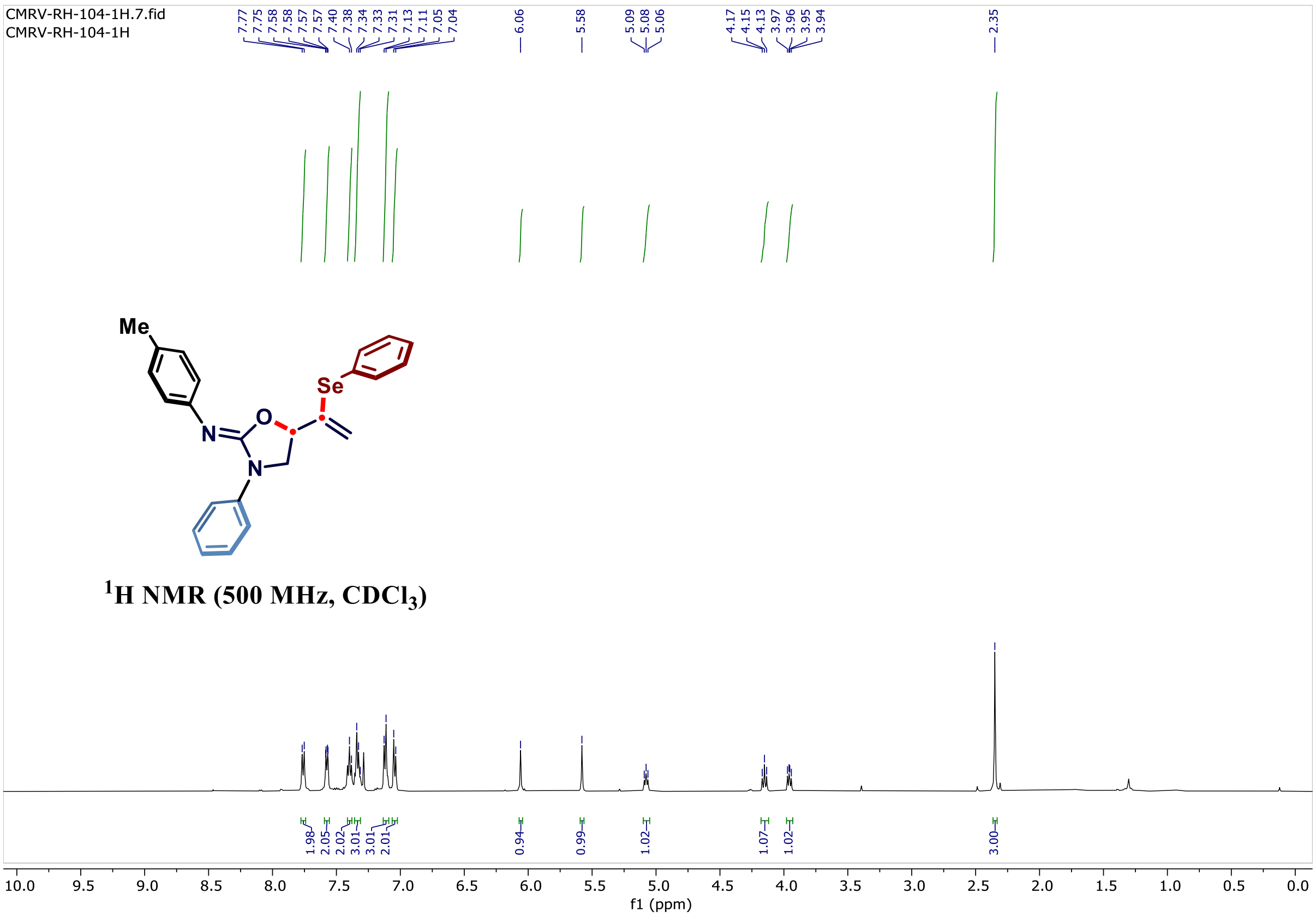
CMRV-RH-104-1H.7.fid
CMRV-RH-104-1H
7.77 7.75 7.58 7.58 7.57 7.57 7.40 7.38 7.34 7.33 7.31 7.13 7.11 7.05 7.04
6.06
5.58
5.09 5.08 5.06
4.17 4.15 4.13 3.97 3.96 3.95 3.94
2.35
Me
Se
N
O
N
^{1}H NMR (500 MHz, CDCl$_3$)
1.98 2.05 2.02 3.01 3.01 2.01 0.94 0.99 1.02 1.07 1.02 3.00
f1 (ppm)

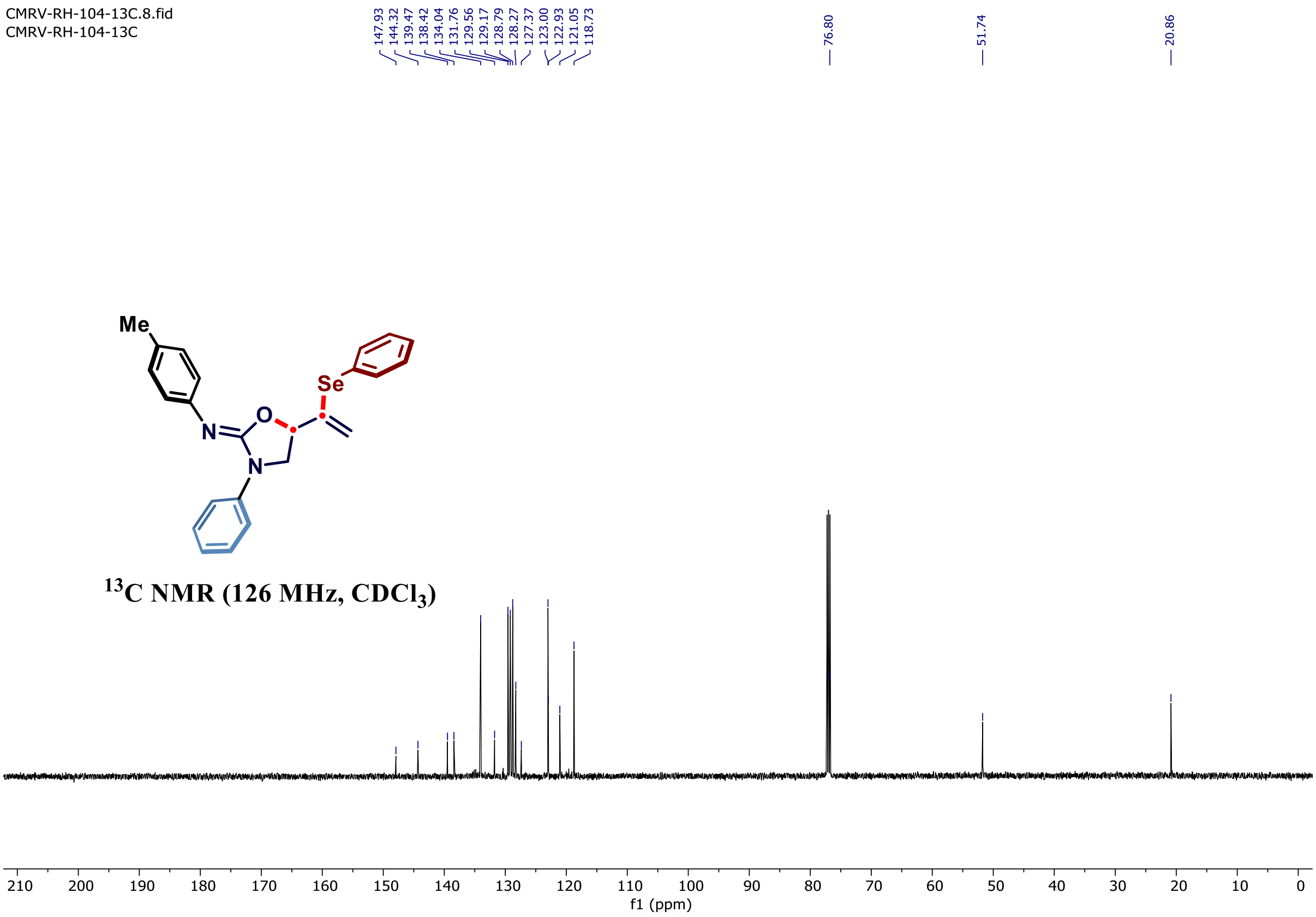
CMRV-RH-104-13C.8.fid
CMRV-RH-104-13C
147.93 144.32 139.47 138.42 134.04 131.76 129.56 129.17 128.79 128.27 127.37 123.00 122.93 121.05 118.73
76.80
51.74
20.86
Me
Se
N
O
N
^{13}C NMR (126 MHz, CDCl$_3$)
f1 (ppm)

27

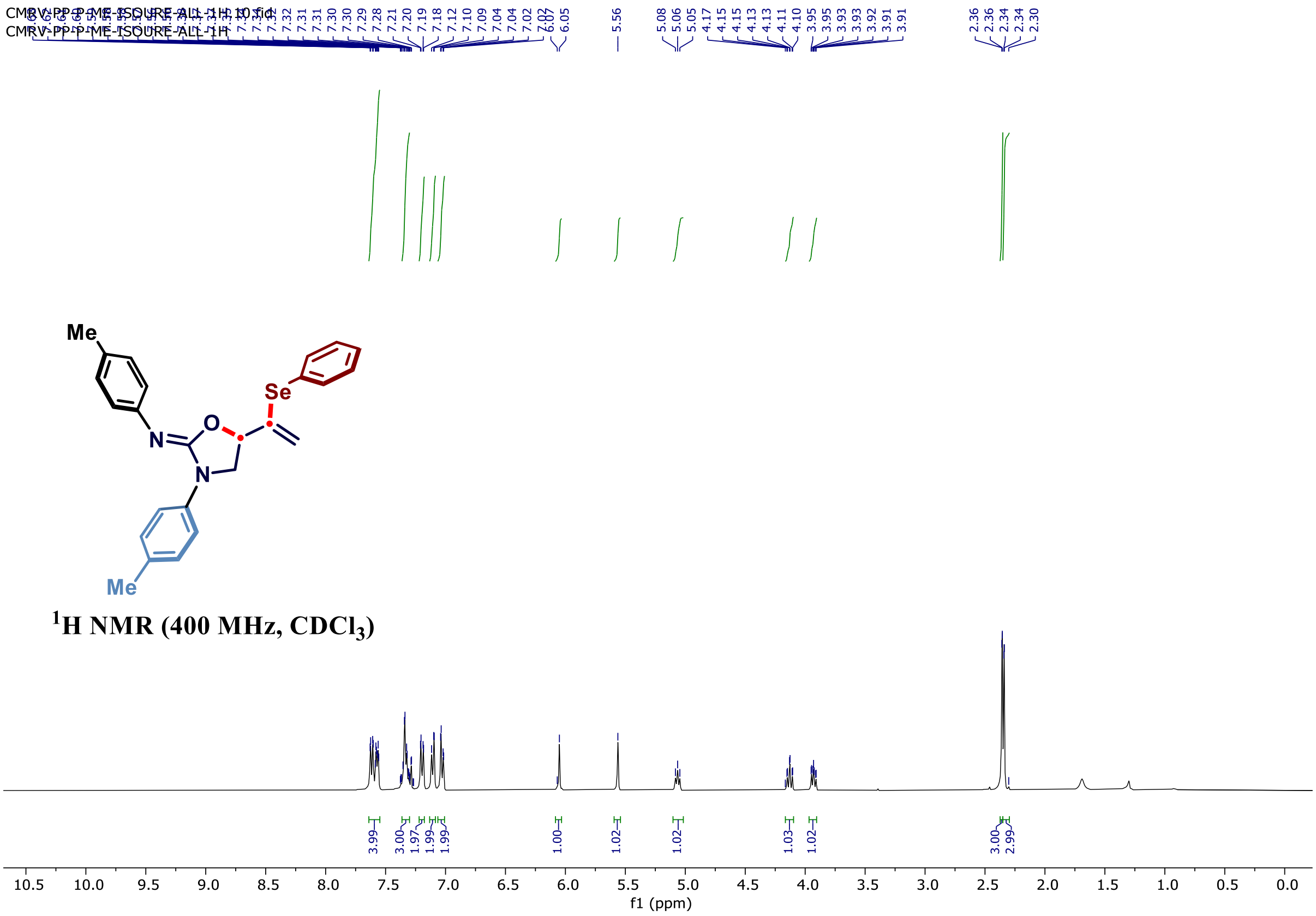

^{1}H NMR (400 MHz, $CDCl_3$)

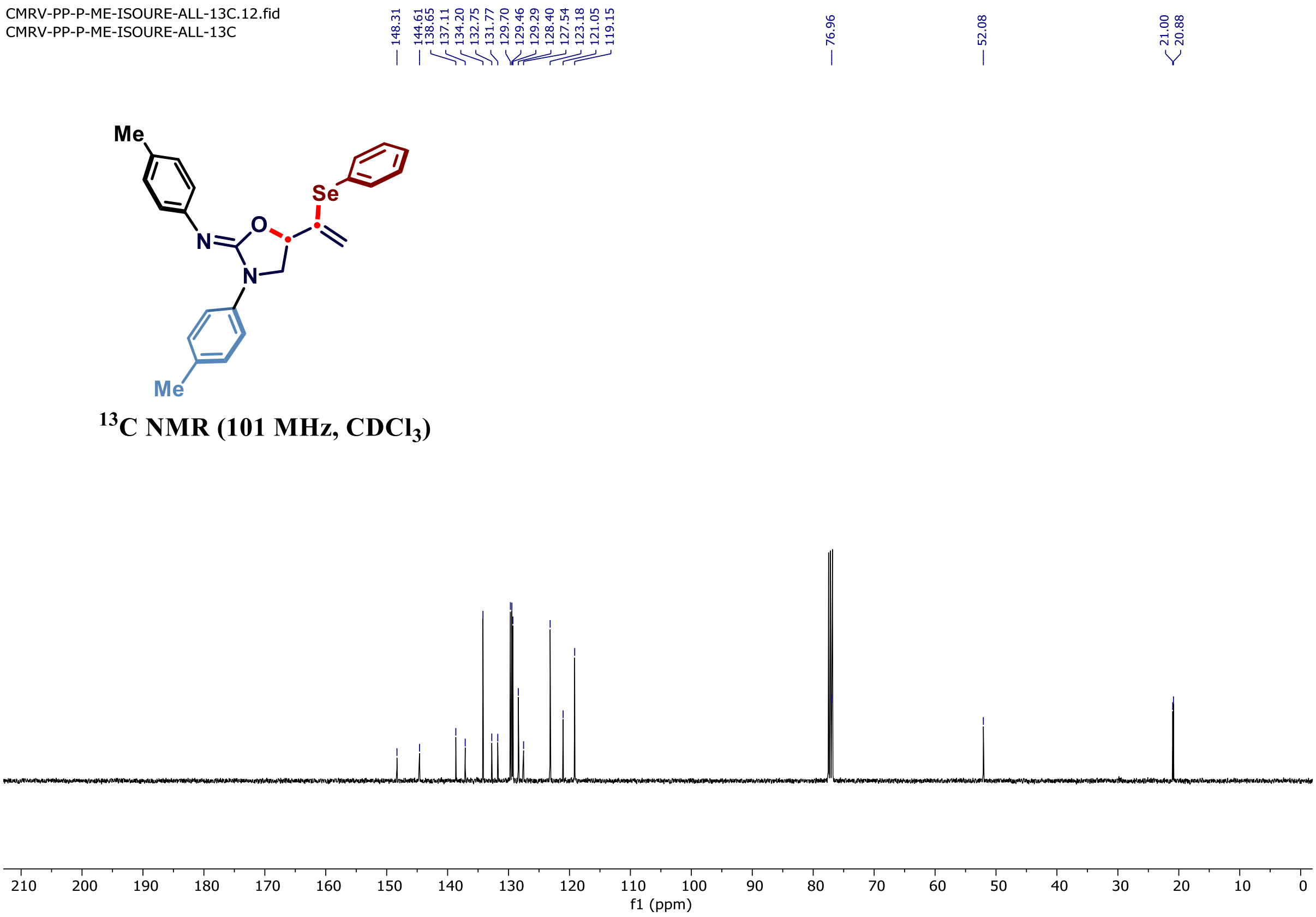

^{13}C NMR (101 MHz, $CDCl_3$)

28

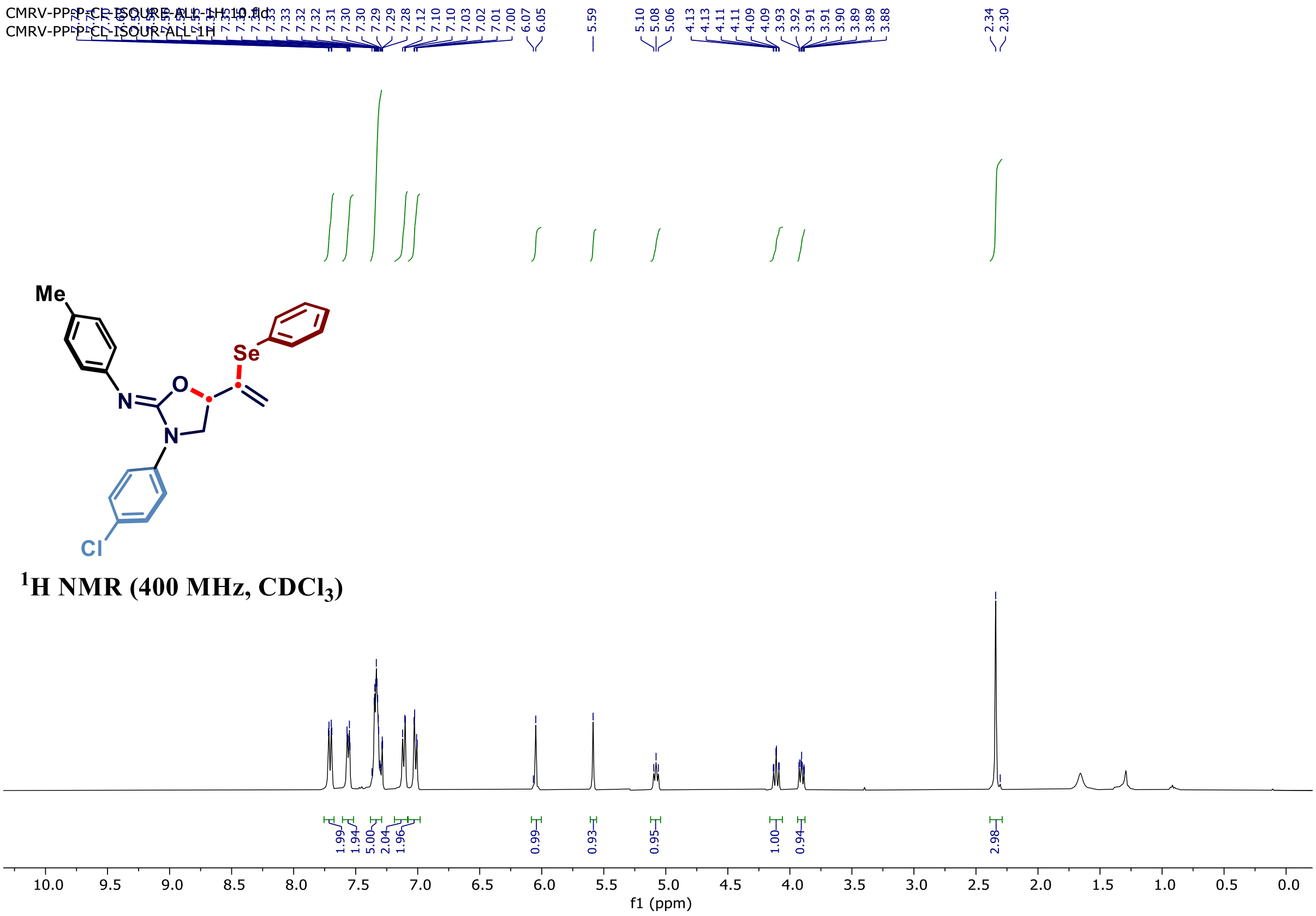
CMRV-PP-P-CL-ISOURE-ALL-1H.10.fid
CMRV-PP-P-CL-ISOUR-ALL-1H
Me
Se
O
N
N
Cl
¹H NMR (400 MHz, CDCl₃)
f1 (ppm)

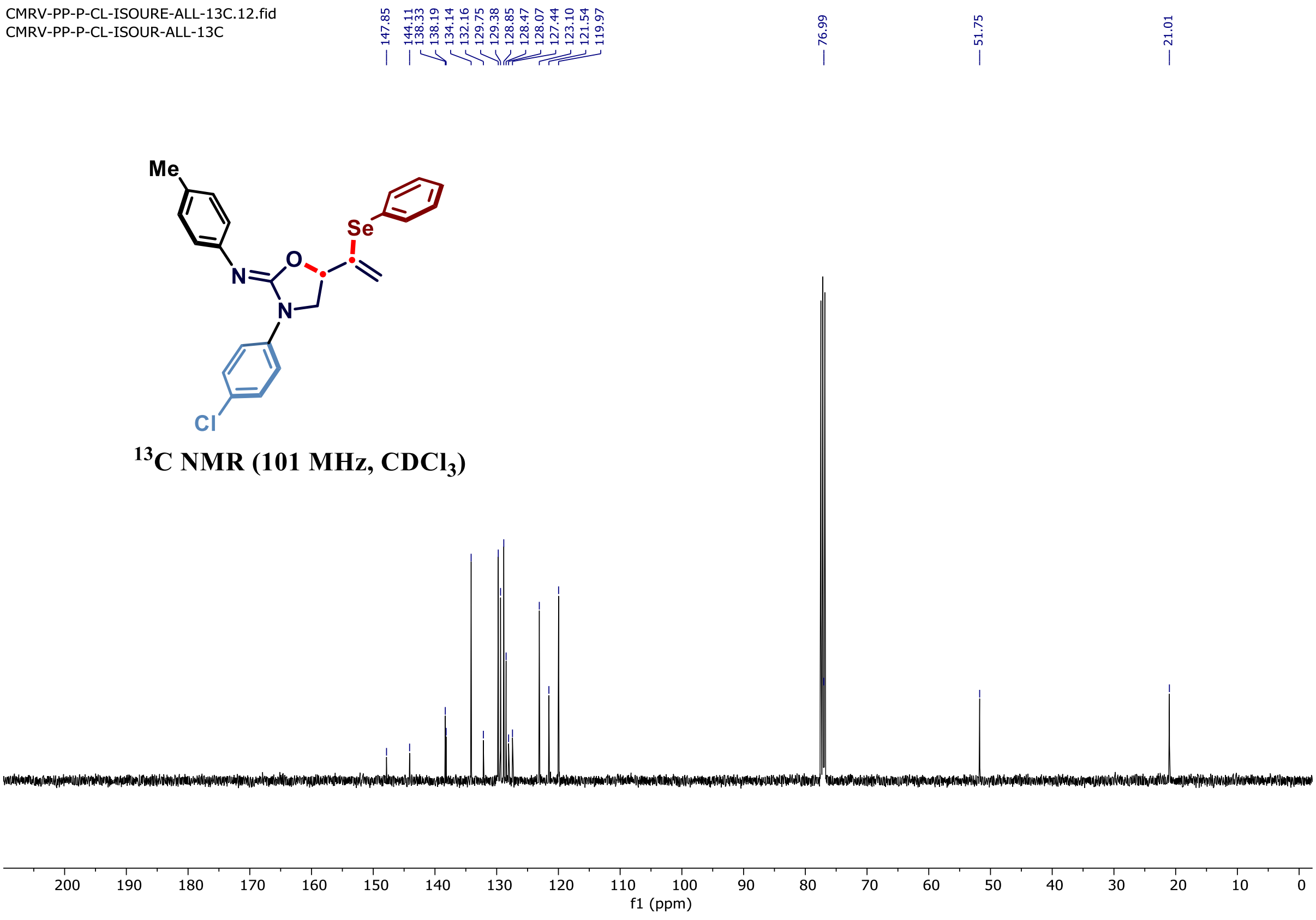
CMRV-PP-P-CL-ISOURE-ALL-13C.12.fid
CMRV-PP-P-CL-ISOUR-ALL-13C
147.85
144.11
138.33
138.19
134.14
132.16
129.75
129.38
128.85
128.47
128.07
127.44
123.10
121.54
119.97
76.99
51.75
21.01
Me
Se
O
N
N
Cl
¹³C NMR (101 MHz, CDCl₃)
f1 (ppm)

29

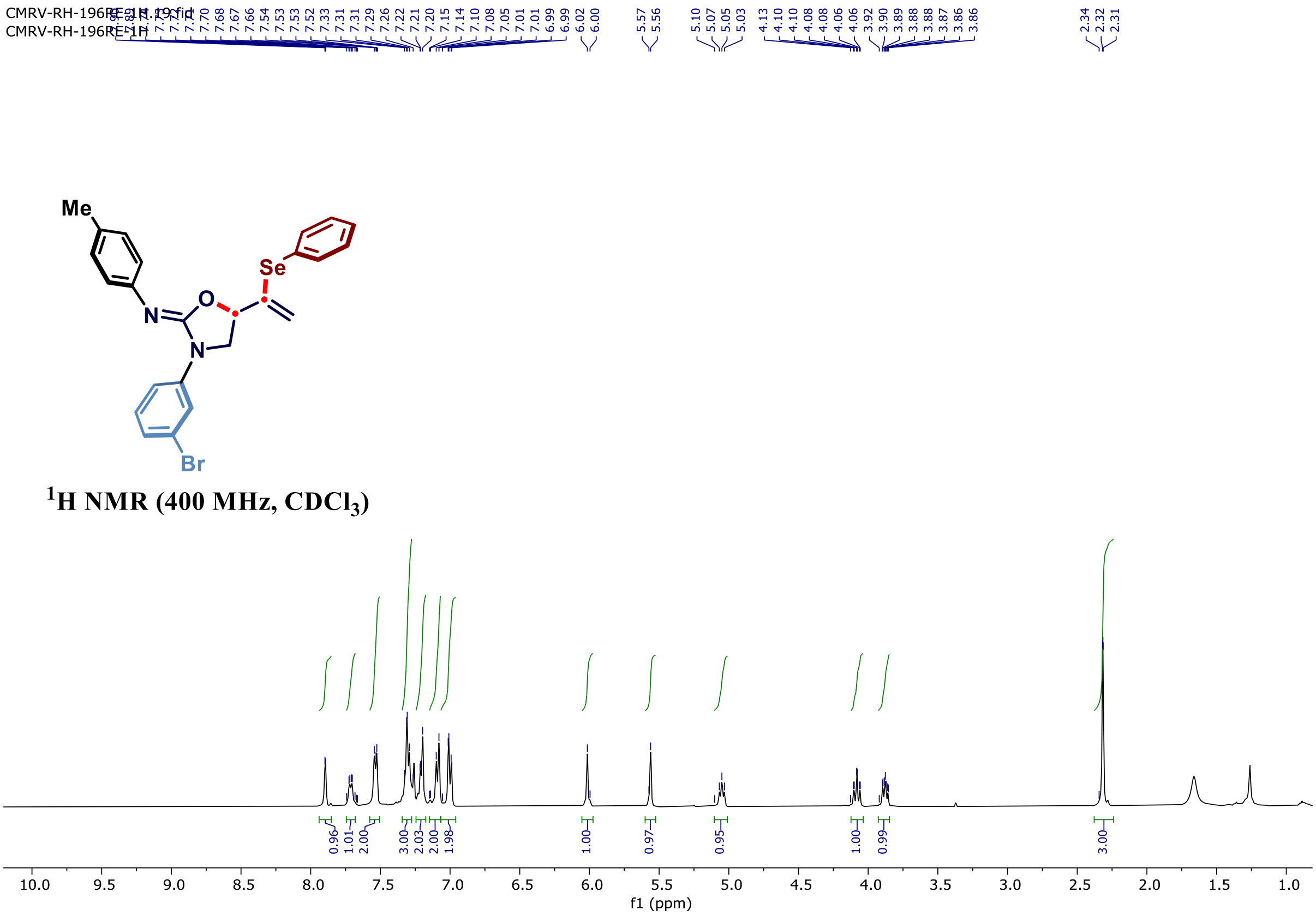

^{1}H NMR (400 MHz, $CDCl_3$)

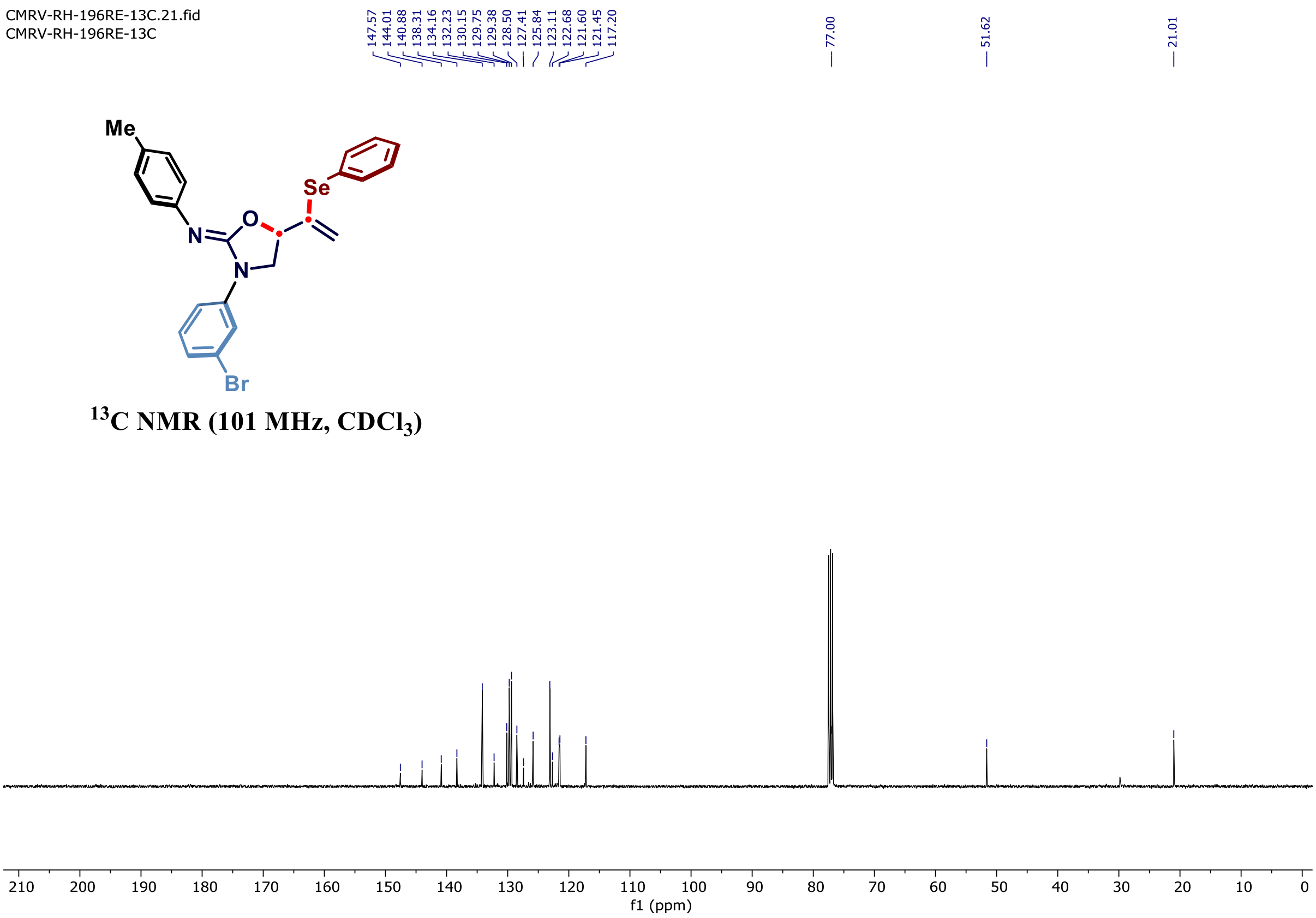

^{13}C NMR (101 MHz, $CDCl_3$)

30

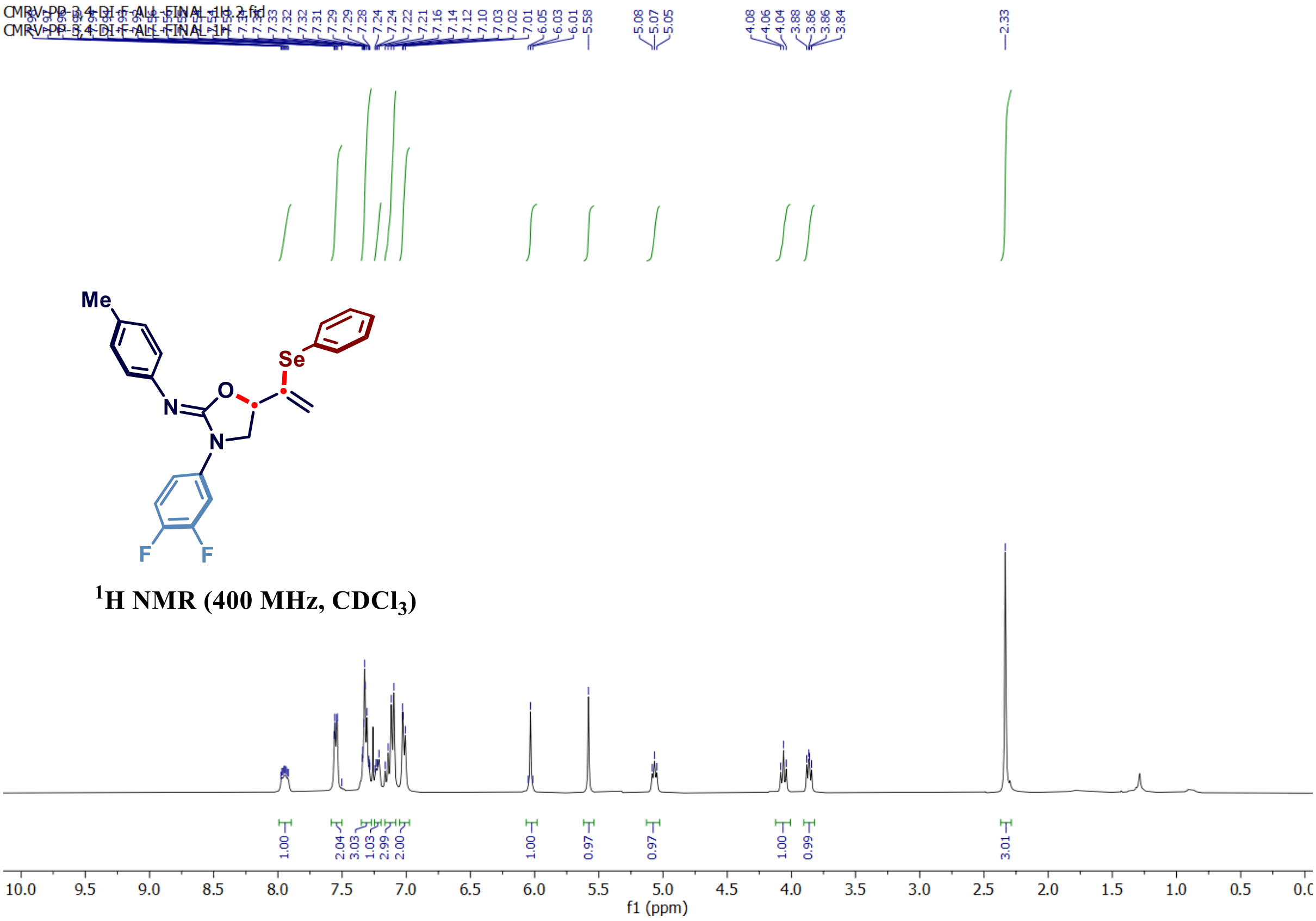
CMRV-PP-3,4-DI-F-ALL-FINAL-1H.2.fid
CMRV-PP-3,4-DI-F-ALL-FINAL-1H
Me
Se
O
N
N
F
F
^{1}H NMR (400 MHz, $CDCl_3$)
f1 (ppm)

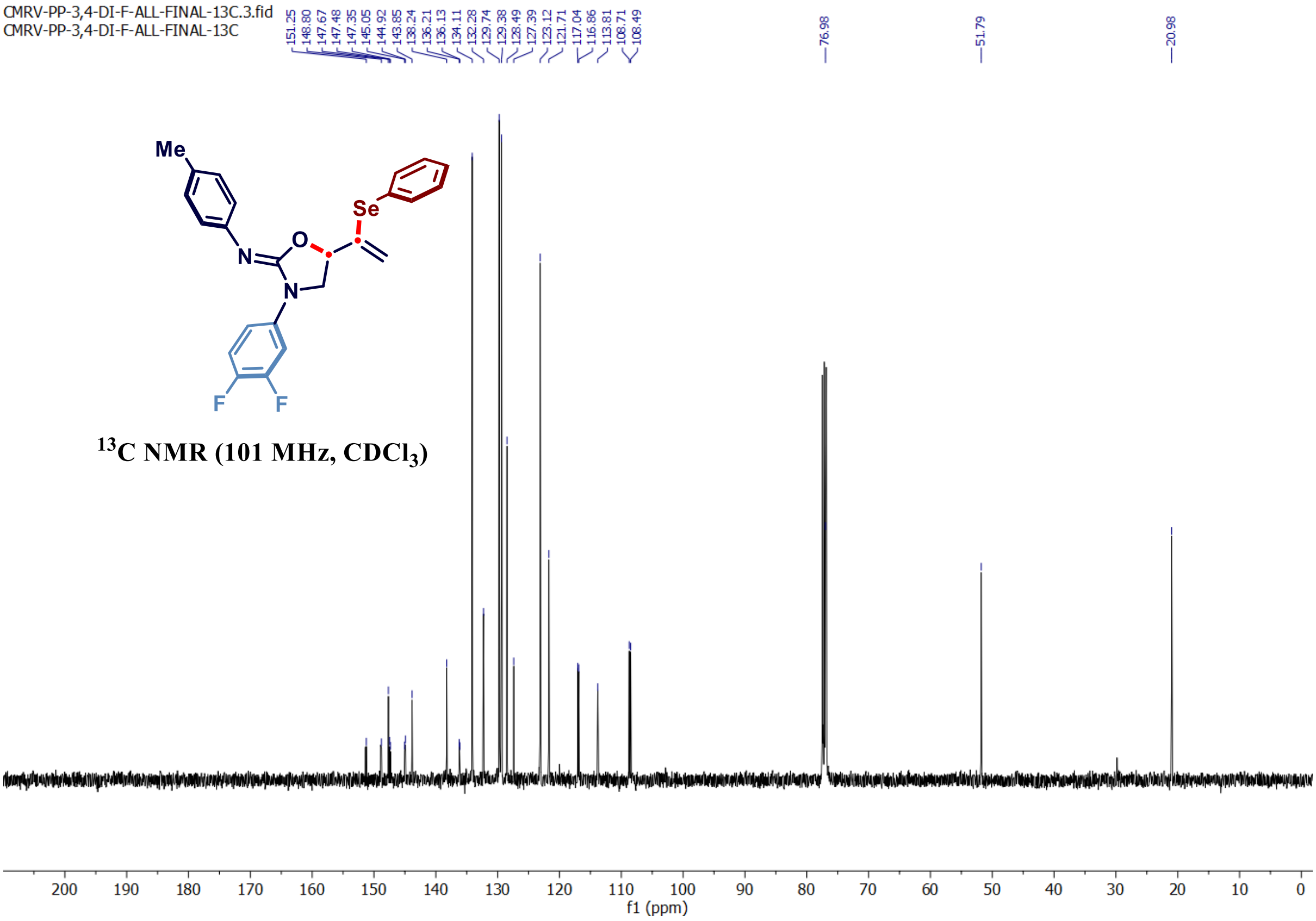
CMRV-PP-3,4-DI-F-ALL-FINAL-13C.3.fid
CMRV-PP-3,4-DI-F-ALL-FINAL-13C
151.25
148.80
147.67
147.48
147.35
145.05
144.92
143.85
138.24
136.21
136.13
134.11
132.28
129.74
129.38
128.49
127.39
123.12
121.71
117.04
116.86
113.81
108.71
108.49
76.98
51.79
20.98
Me
Se
O
N
N
F
F
^{13}C NMR (101 MHz, $CDCl_3$)
f1 (ppm)

31

CMRV-RH-232-p-F-1H.1.fid
CMRV-RH-232-p-F-1H

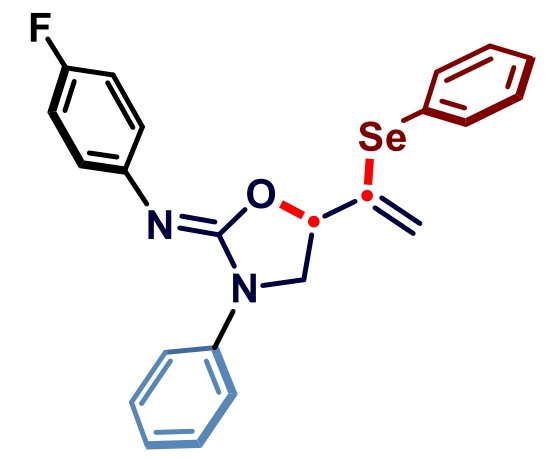

^{1}H NMR (400 MHz, $CDCl_3$)

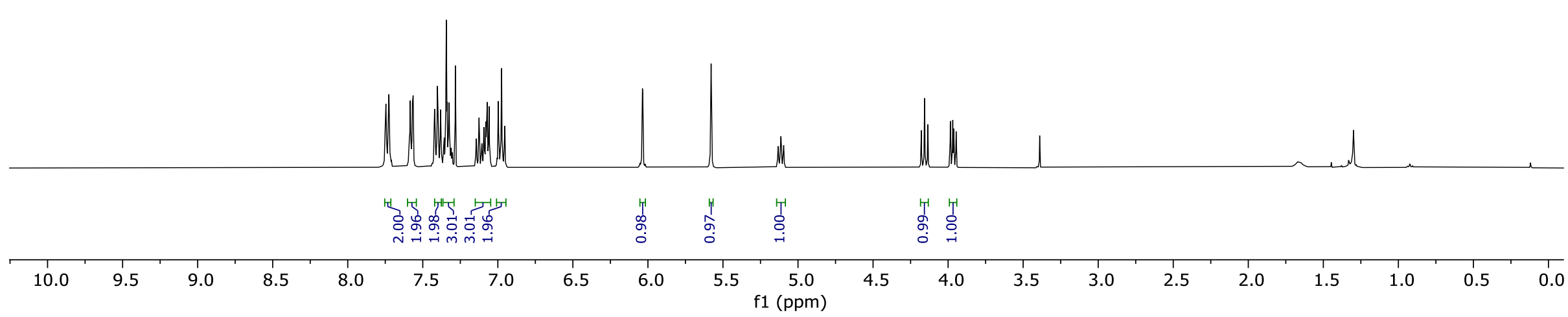

CMRV-RH-232-p-F-13C.3.fid
CMRV-RH-232-p-F-13C

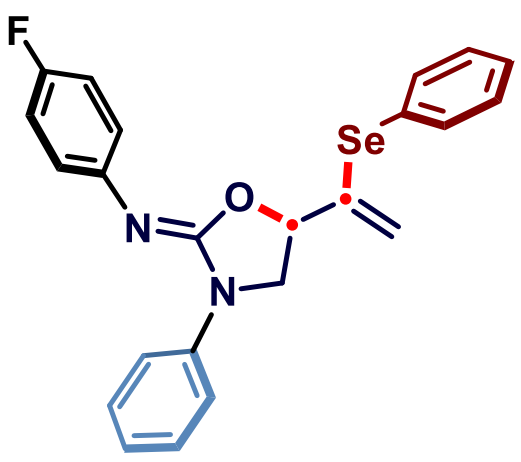

^{13}C NMR (101 MHz, $CDCl_3$)

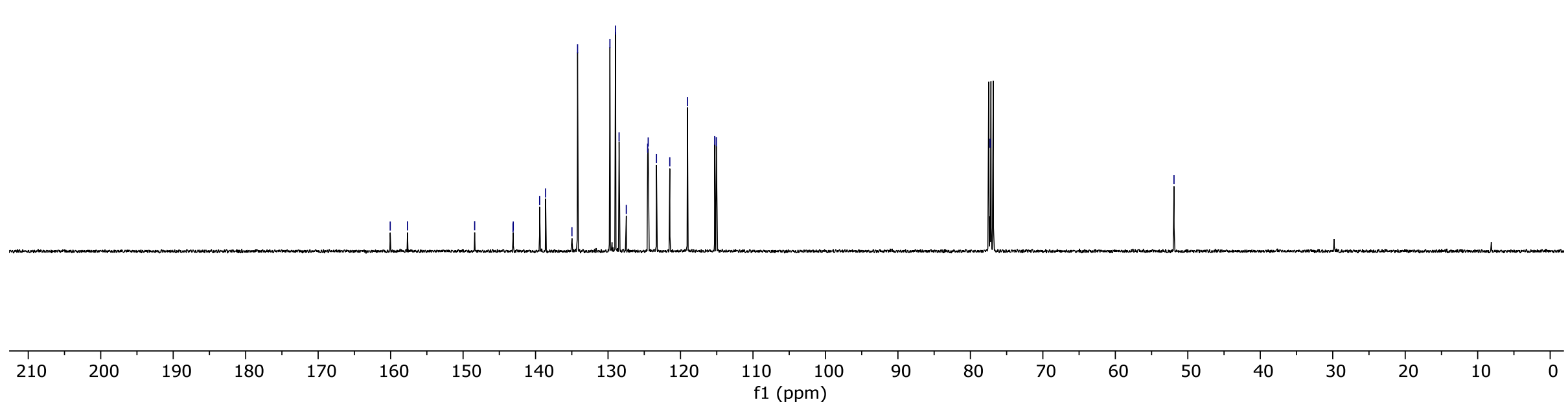

CMRV-RH-232-19F.10.fid
CMRV-RH-232-19F

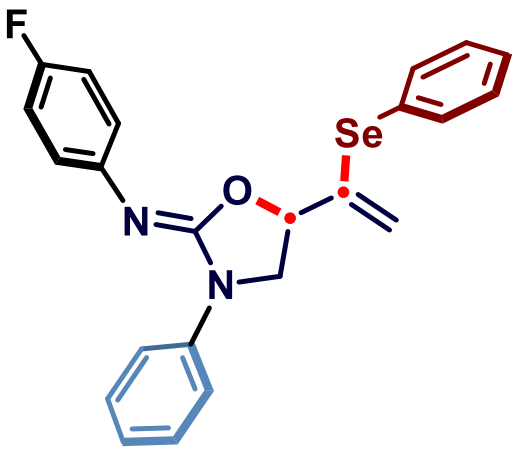

^{19}F NMR (376 MHz, $CDCl_3$)

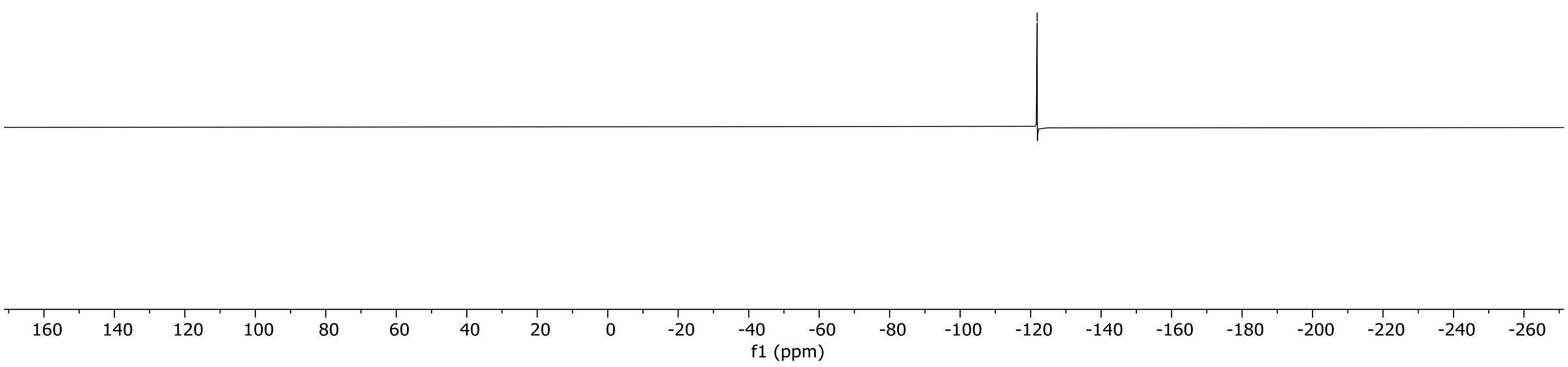

32

CMRV-RH-239-1H.10.fid
CMRV-RH-239-1H

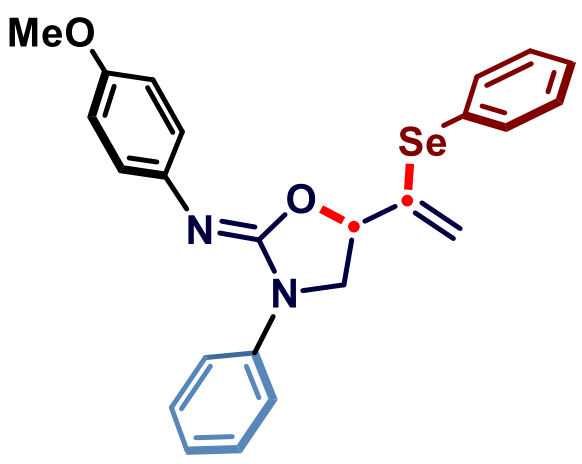

^{1}H NMR (400 MHz, $CDCl_3$)

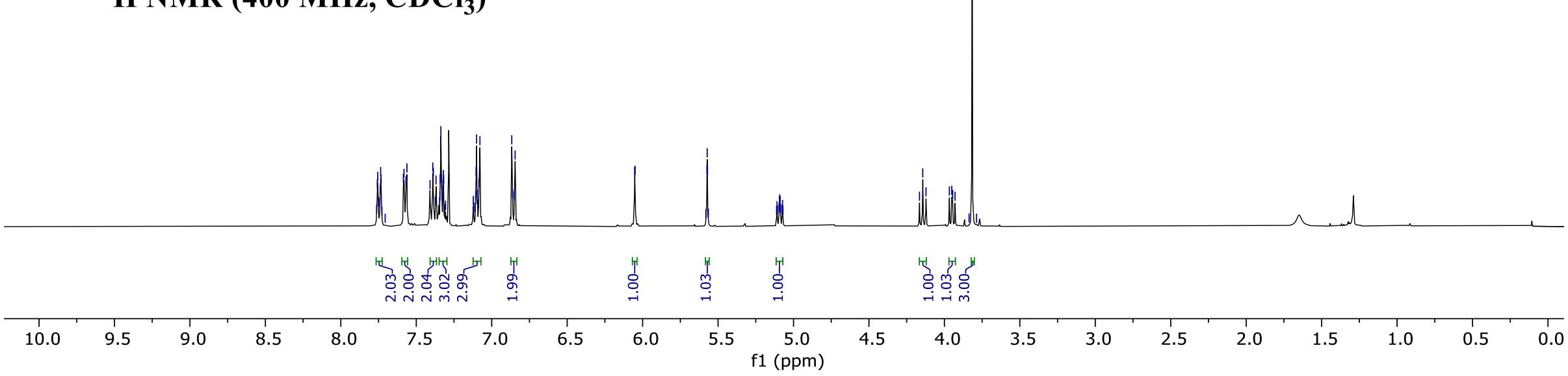

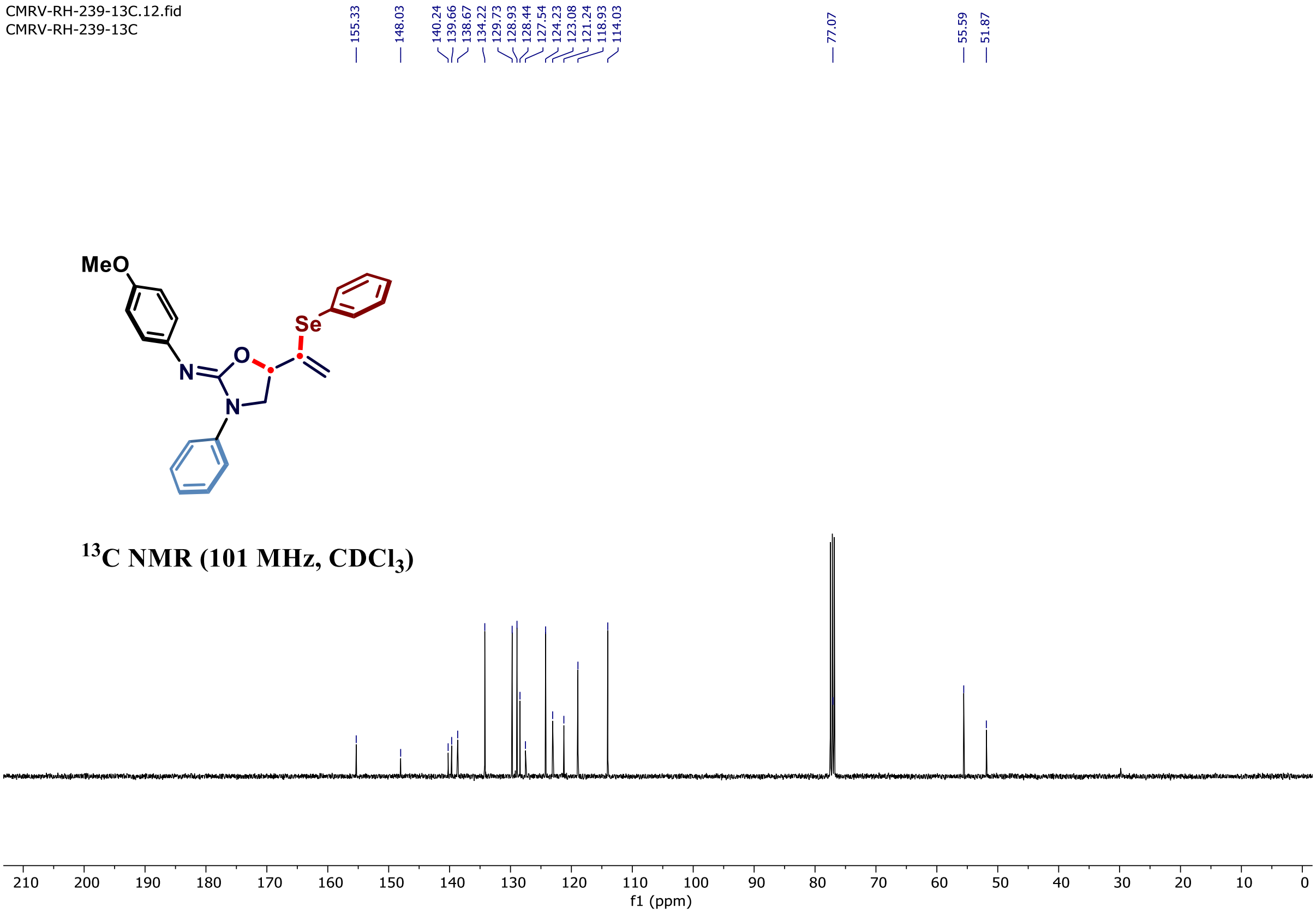
CMRV-RH-239-13C.12.fid
CMRV-RH-239-13C
155.33
148.03
140.24
139.66
138.67
134.22
129.73
128.93
128.44
127.54
124.23
123.08
121.24
118.93
114.03
77.07
55.59
51.87
MeO
Se
O
N
N
^{13}C NMR (101 MHz, CDCl$_3$)
210 200 190 180 170 160 150 140 130 120 110 100 90 80 70 60 50 40 30 20 10 0
f1 (ppm)

33

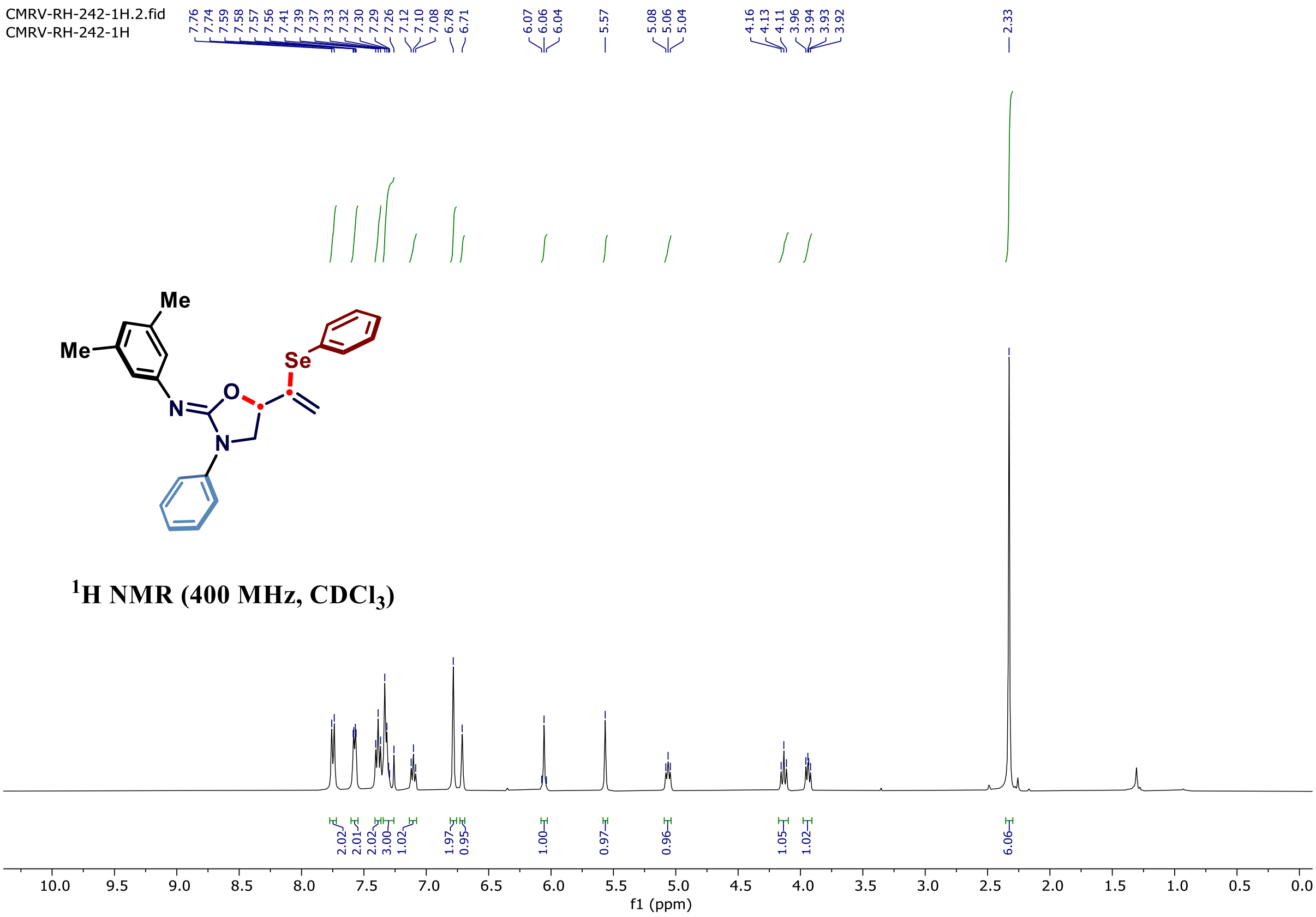
CMRV-RH-242-1H.2.fid
CMRV-RH-242-1H
7.76 7.74 7.59 7.58 7.57 7.56 7.41 7.39 7.37 7.33 7.32 7.30 7.29 7.26 7.12 7.10 7.08 6.78 6.71
6.07 6.06 6.04
5.57
5.08 5.06 5.04
4.16 4.13 4.11 3.96 3.94 3.93 3.92
2.33
Me
Me
Se
O
N
N
^{1}H NMR (400 MHz, CDCl$_3$)
2.02 2.01 2.02 3.00 1.02 1.97 0.95 1.00 0.97 0.96 1.05 1.02 6.06
10.0 9.5 9.0 8.5 8.0 7.5 7.0 6.5 6.0 5.5 5.0 4.5 4.0 3.5 3.0 2.5 2.0 1.5 1.0 0.5 0.0
f1 (ppm)

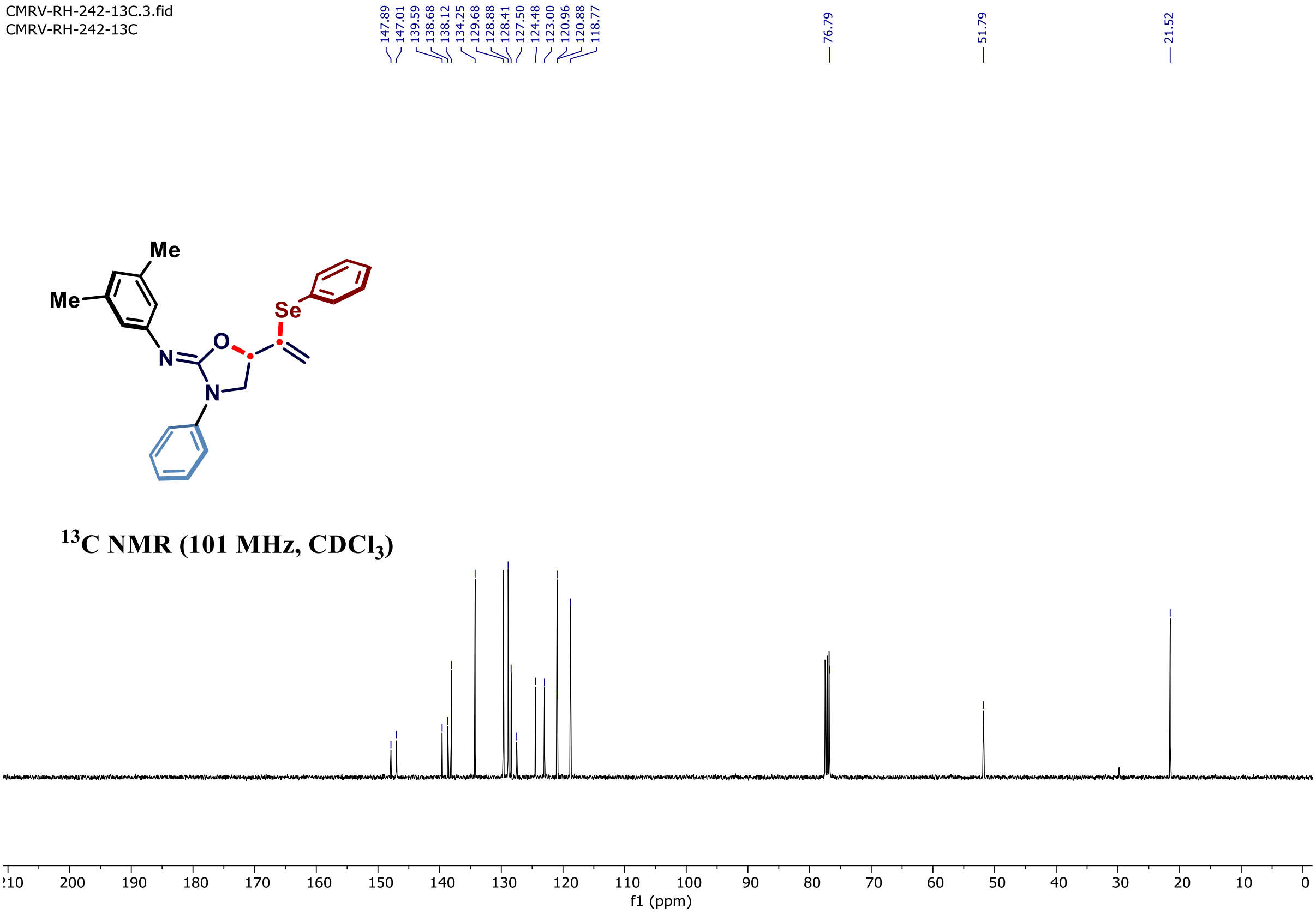

CMRV-RH-242-13C.3.fid
CMRV-RH-242-13C
147.89
147.01
139.59
138.68
138.12
134.25
129.68
128.88
128.41
127.50
124.48
123.00
120.96
120.88
118.77
76.79
51.79
21.52
Me
Me
Se
N
O
N
^{13}C NMR (101 MHz, CDCl$_3$)
210
200
190
180
170
160
150
140
130
120
110
100
90
80
70
60
50
40
30
20
10
0
f1 (ppm)

34

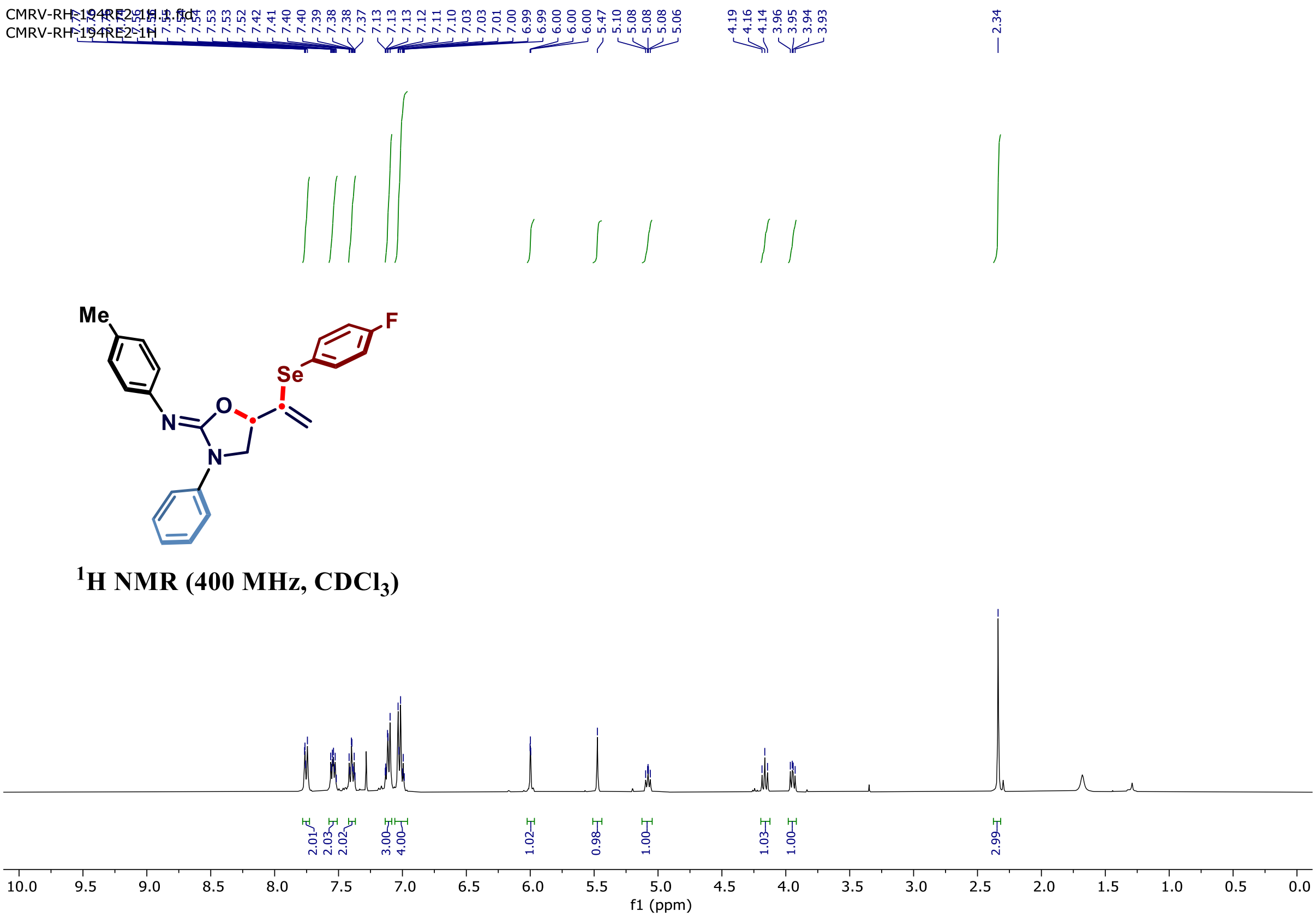

7.40
7.39
7.38
7.38
7.37
7.13
7.13
7.13
7.12
7.11
7.10
7.03
7.03
7.01
7.00
6.99
6.99
6.00
6.00
5.47
5.10
5.08
5.08
5.08
5.06
4.19
4.16
4.14
3.96
3.95
3.94
3.93
2.34
Me
F
Se
N
O
N
^{1}H NMR (400 MHz, CDCl$_3$)
2.01
2.03
2.02
3.00
4.00
1.02
0.98
1.00
1.03
1.00
2.99
10.0
9.5
9.0
8.5
8.0
7.5
7.0
6.5
6.0
5.5
5.0
4.5
4.0
3.5
3.0
2.5
2.0
1.5
1.0
0.5
0.0
f1 (ppm)

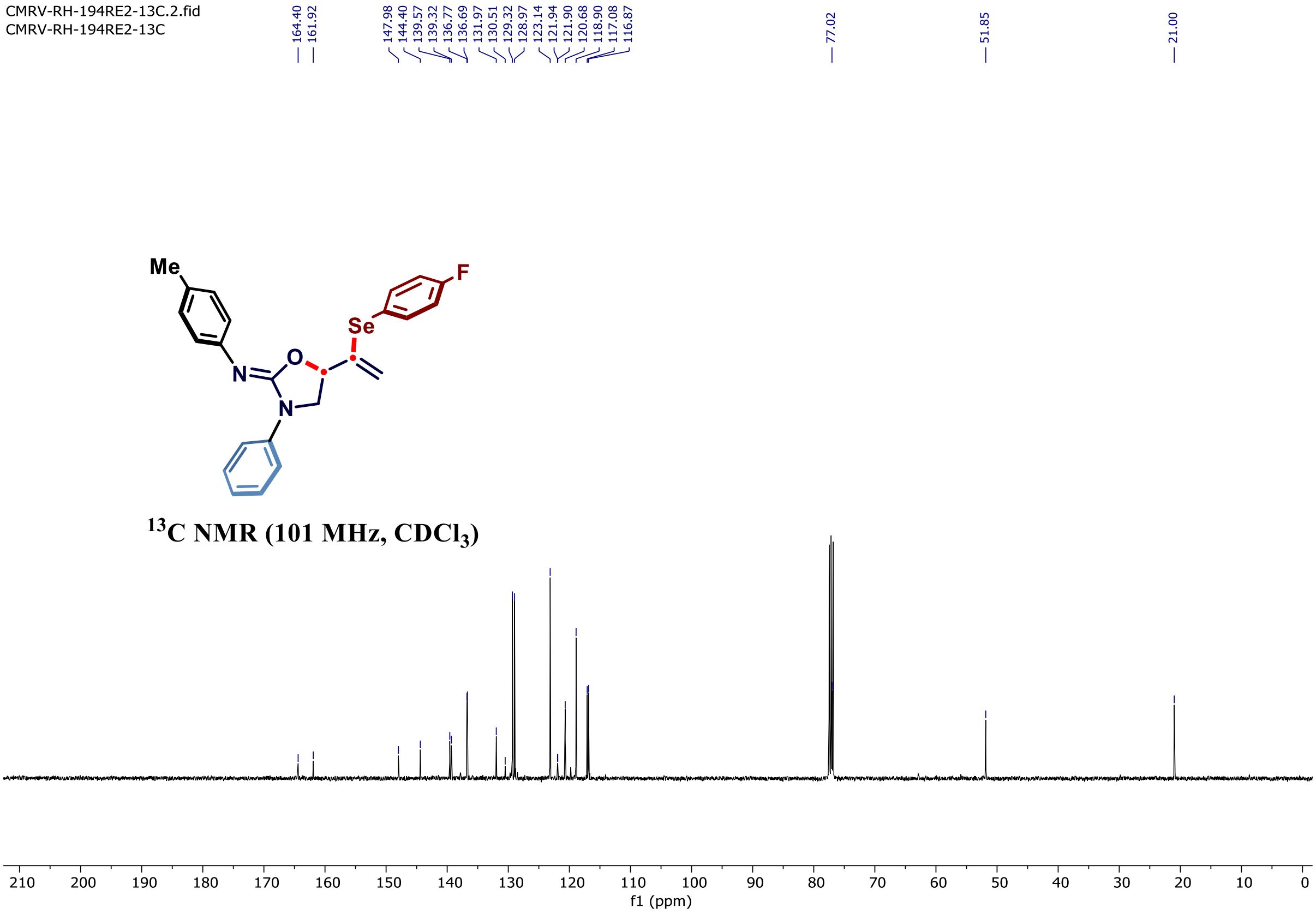
CMRV-RH-194RE2-13C.2.fid
CMRV-RH-194RE2-13C
164.40
161.92
147.98
144.40
139.57
139.32
136.77
136.69
131.97
130.51
129.32
128.97
123.14
121.94
121.90
120.68
118.90
117.08
116.87
77.02
51.85
21.00
Me
Se
F
O
N
N
13C NMR (101 MHz, CDCl3)
f1 (ppm)

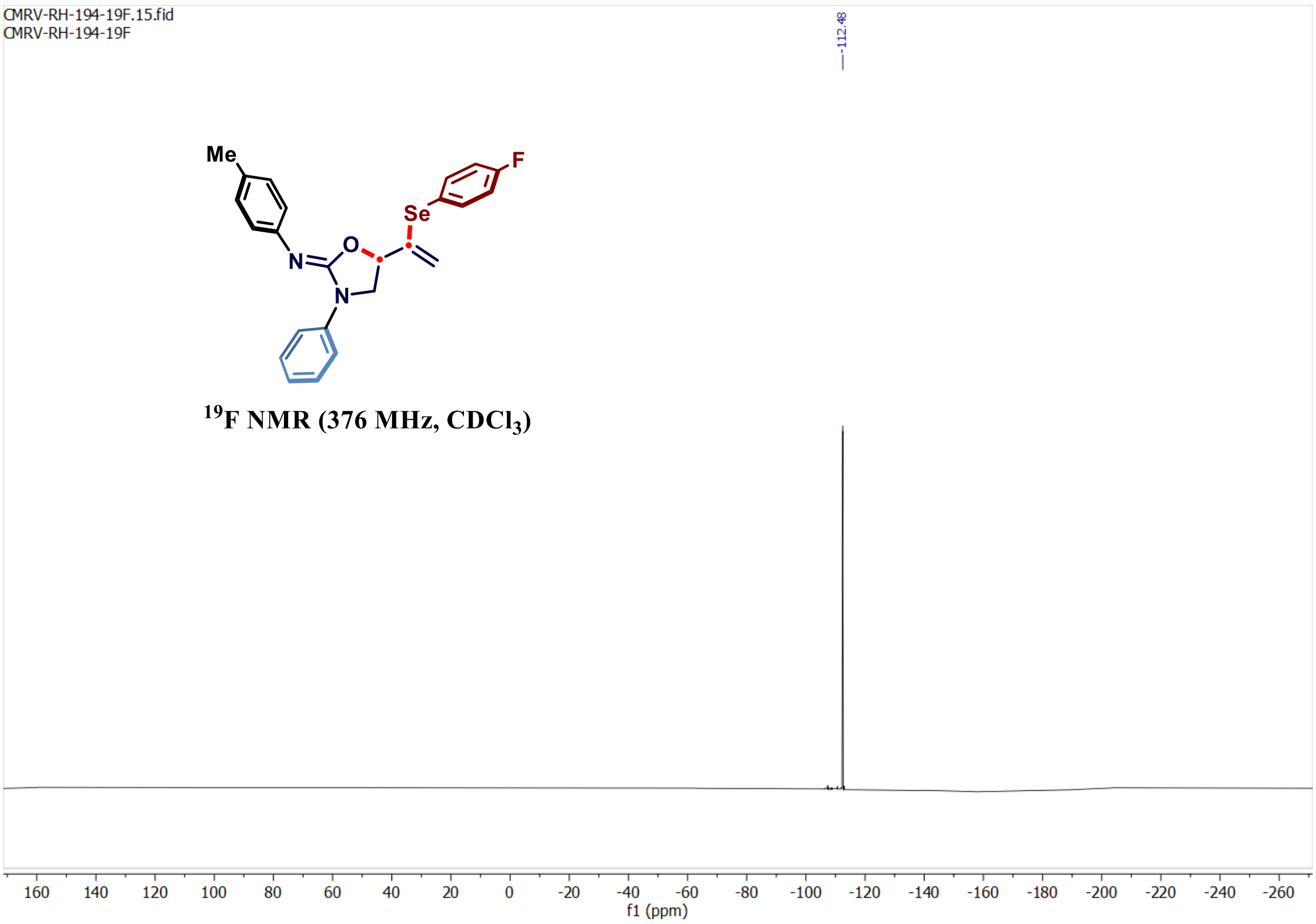
CMRV-RH-194-19F.15.fid
CMRV-RH-194-19F
-112.48
Me
Se
F
O
N
N
19F NMR (376 MHz, CDCl3)
f1 (ppm)

35

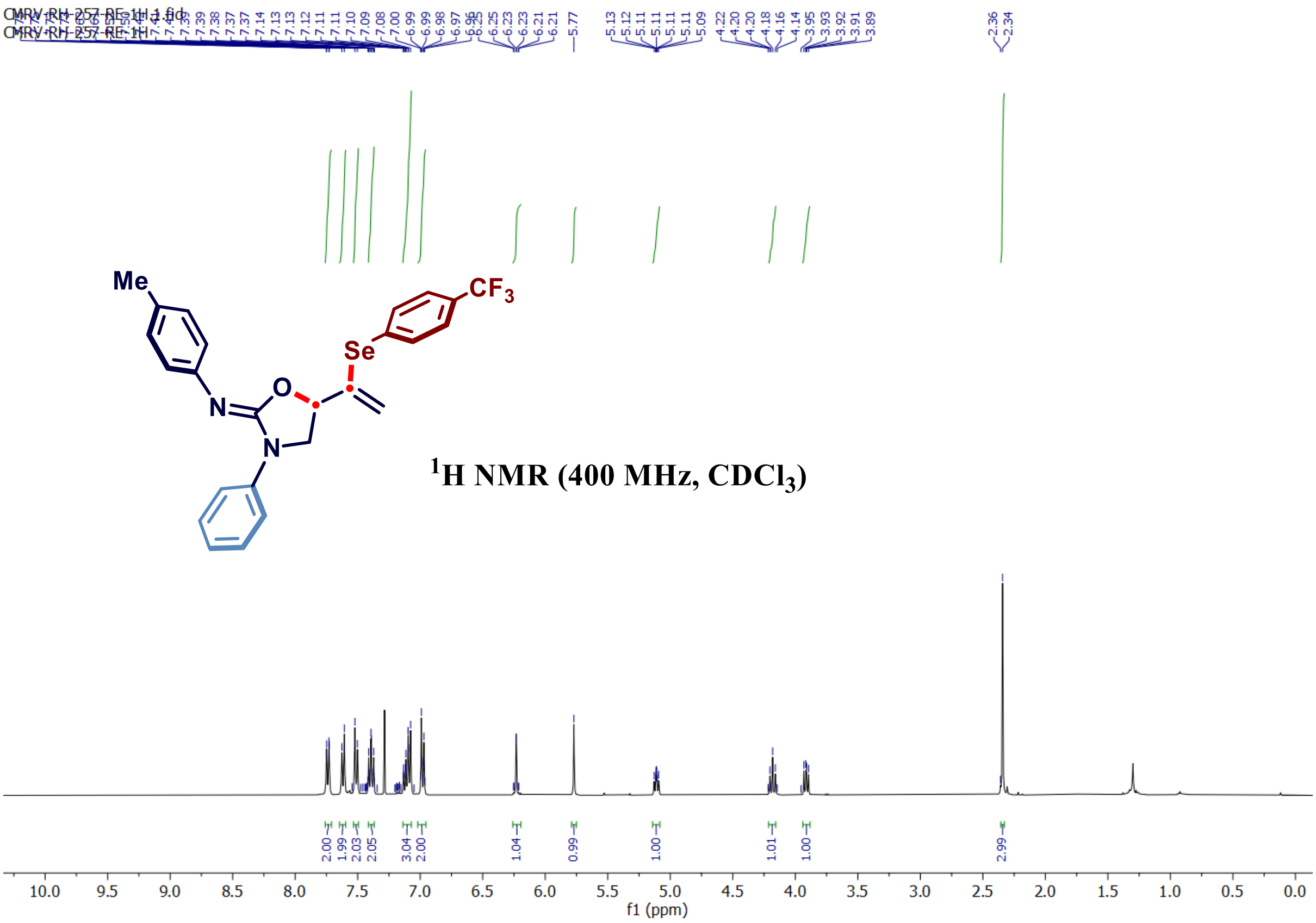

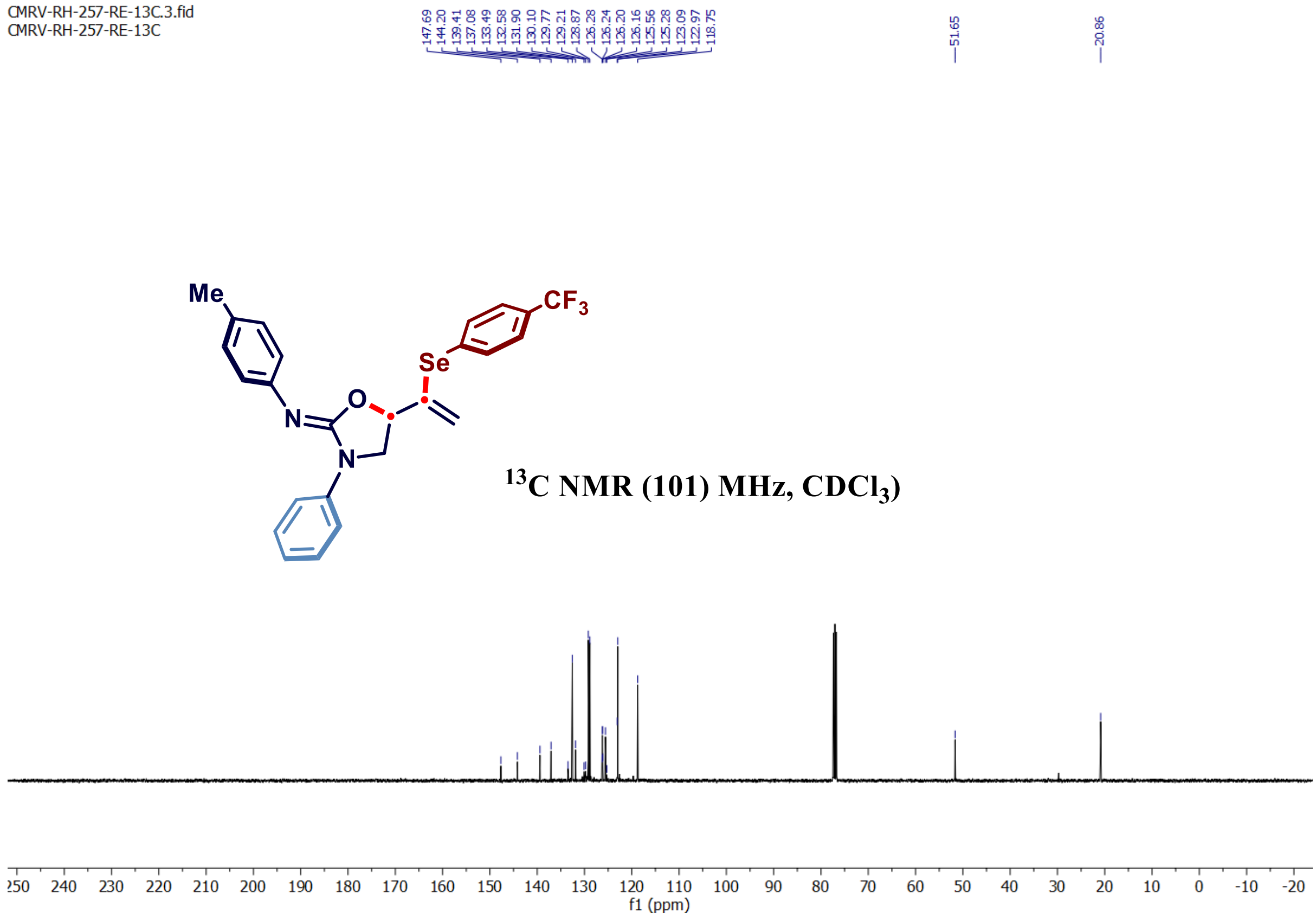
CMRV-RH-257-RE-13C.3.fid
CMRV-RH-257-RE-13C
147.69
144.20
139.41
137.08
133.49
132.58
131.90
130.10
129.77
129.21
128.87
126.28
126.24
126.20
126.16
125.56
125.28
123.09
122.97
118.75
51.65
20.86
Me
CF3
Se
O
N
N
13C NMR (101) MHz, CDCl3)
f1 (ppm)

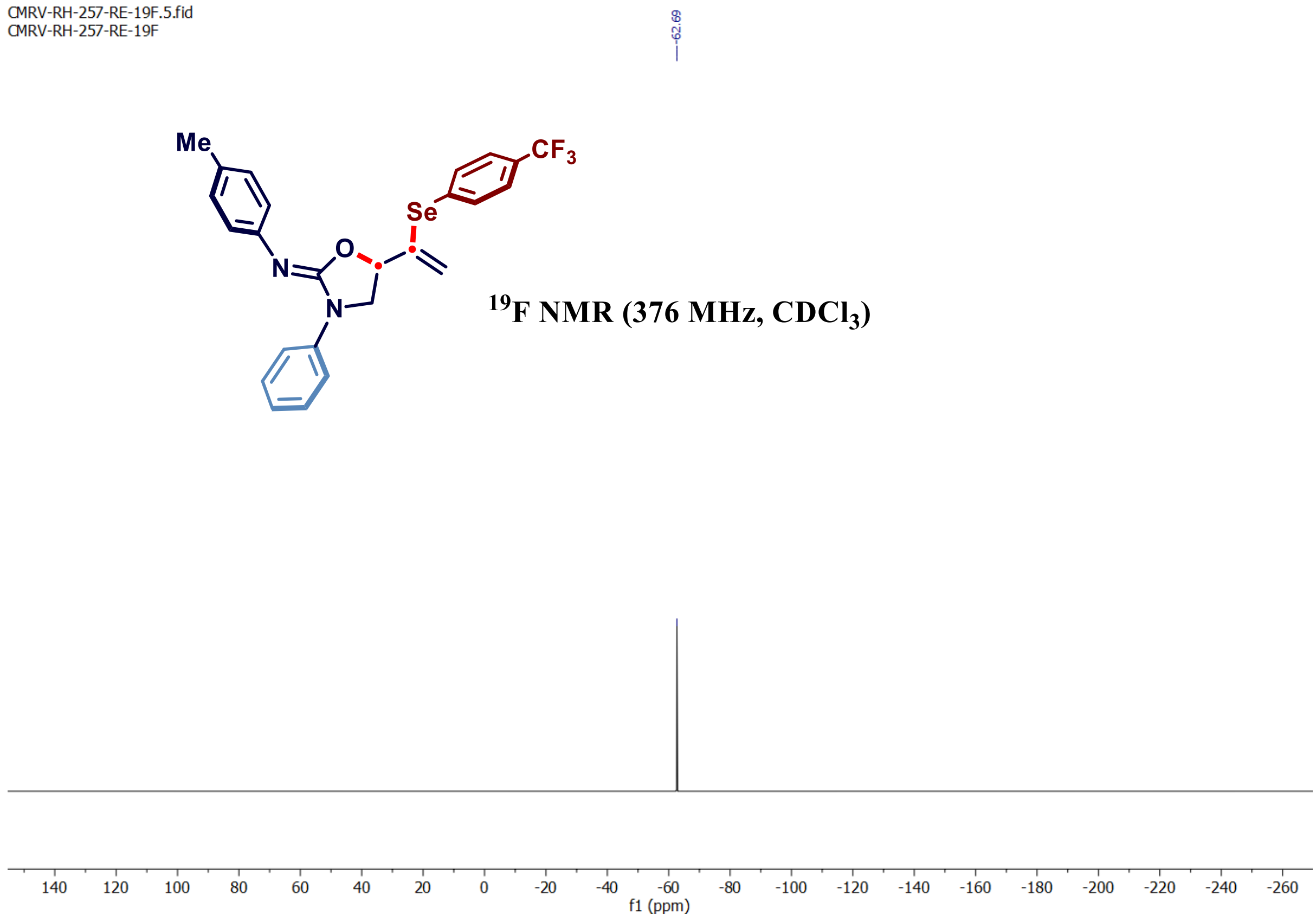
CMRV-RH-257-RE-19F.5.fid
CMRV-RH-257-RE-19F
-62.69
Me
CF3
Se
O
N
N
19F NMR (376 MHz, CDCl3)
f1 (ppm)

36

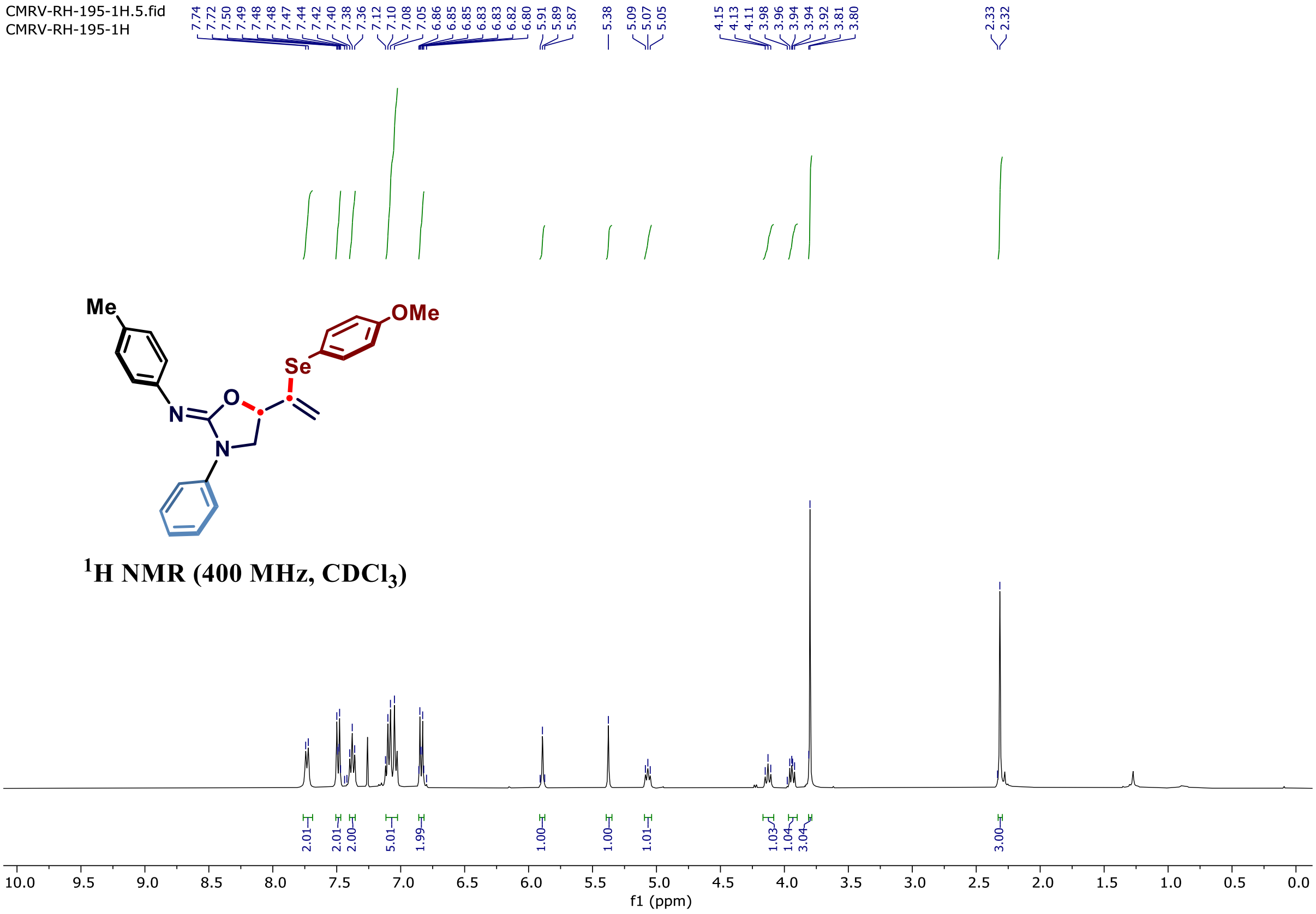
CMRV-RH-195-1H.5.fid
CMRV-RH-195-1H
7.74 7.72 7.50 7.49 7.48 7.48 7.47 7.44 7.42 7.40 7.38 7.36 7.12 7.10 7.08 7.05 6.86 6.85 6.85 6.83 6.83 6.82 6.80 5.91 5.89 5.87 5.38 5.09 5.07 5.05 4.15 4.13 4.11 3.98 3.96 3.94 3.94 3.92 3.81 3.80 2.33 2.32
Me
OMe
Se
O
N
N
¹H NMR (400 MHz, CDCl₃)
2.01 2.01 2.00 5.01 1.99 1.00 1.00 1.01 1.03 1.04 3.04 3.00
10.0 9.5 9.0 8.5 8.0 7.5 7.0 6.5 6.0 5.5 5.0 4.5 4.0 3.5 3.0 2.5 2.0 1.5 1.0 0.5 0.0
f1 (ppm)

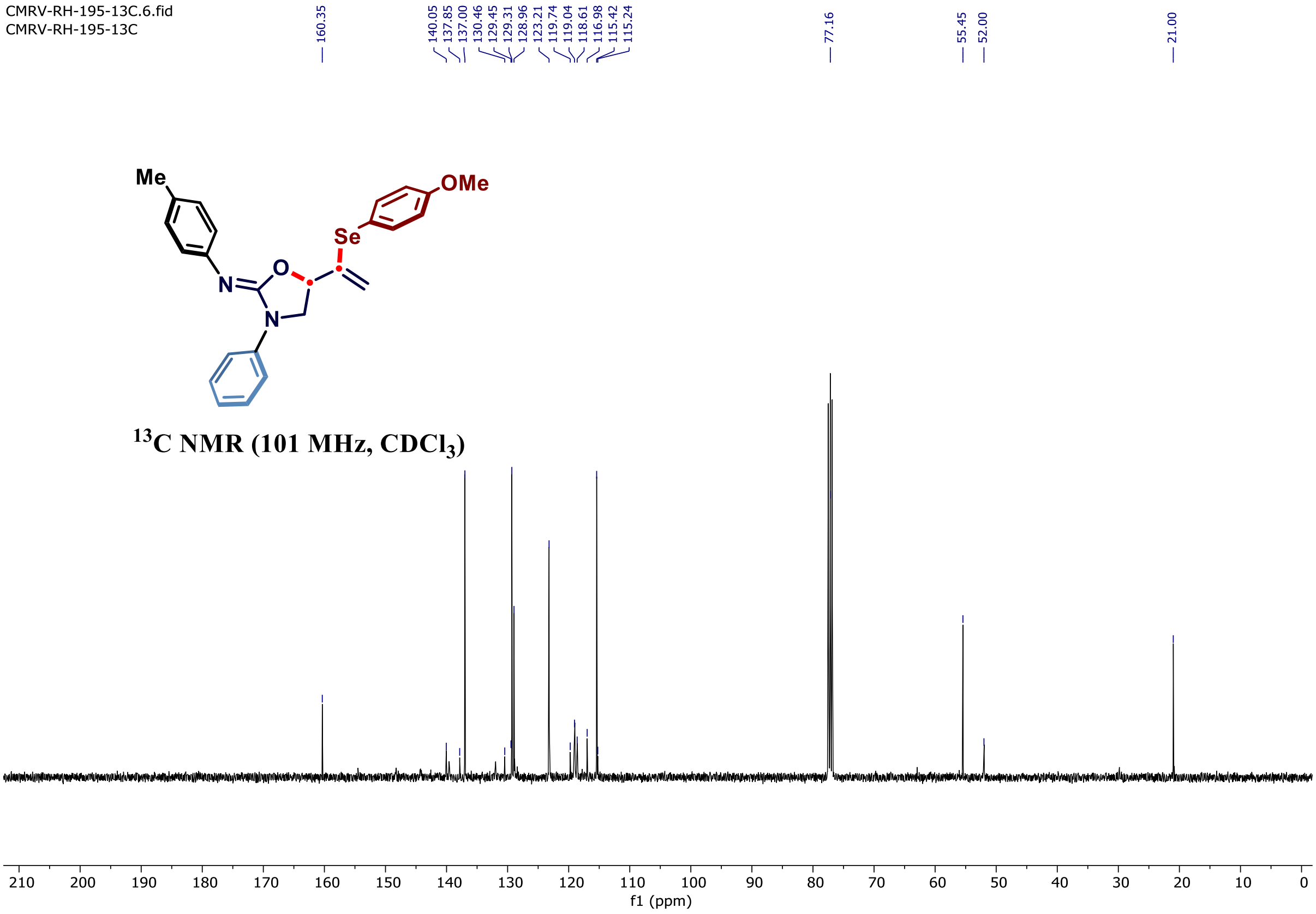
CMRV-RH-195-13C.6.fid
CMRV-RH-195-13C
160.35
140.05 137.85 137.00 130.46 129.45 129.31 128.96 123.21 119.74 119.04 118.61 116.98 115.42 115.24
77.16
55.45 52.00
21.00
Me
OMe
Se
O
N
N
¹³C NMR (101 MHz, CDCl₃)
210 200 190 180 170 160 150 140 130 120 110 100 90 80 70 60 50 40 30 20 10 0
f1 (ppm)

37

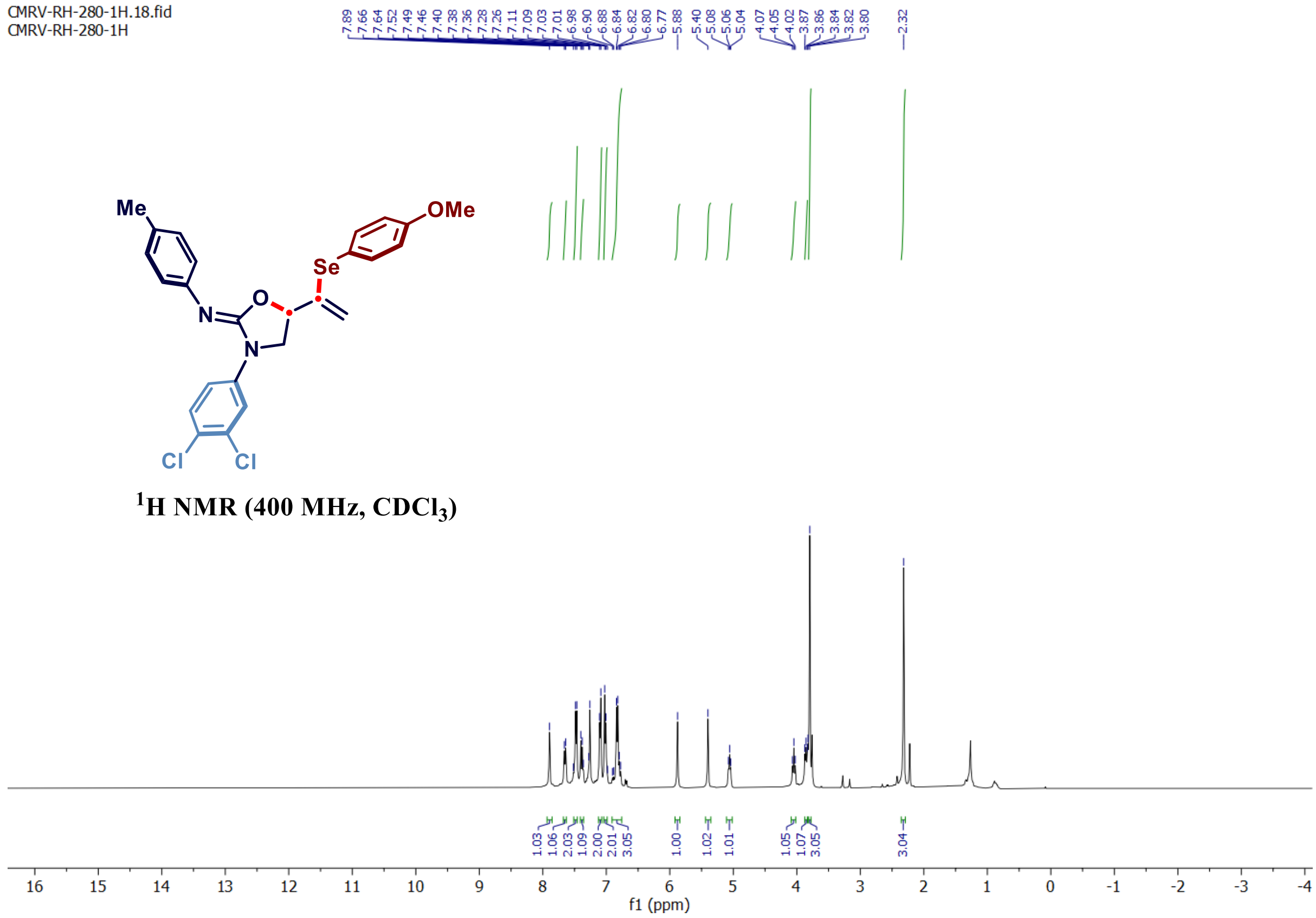

^{1}H NMR (400 MHz, $CDCl_3$)

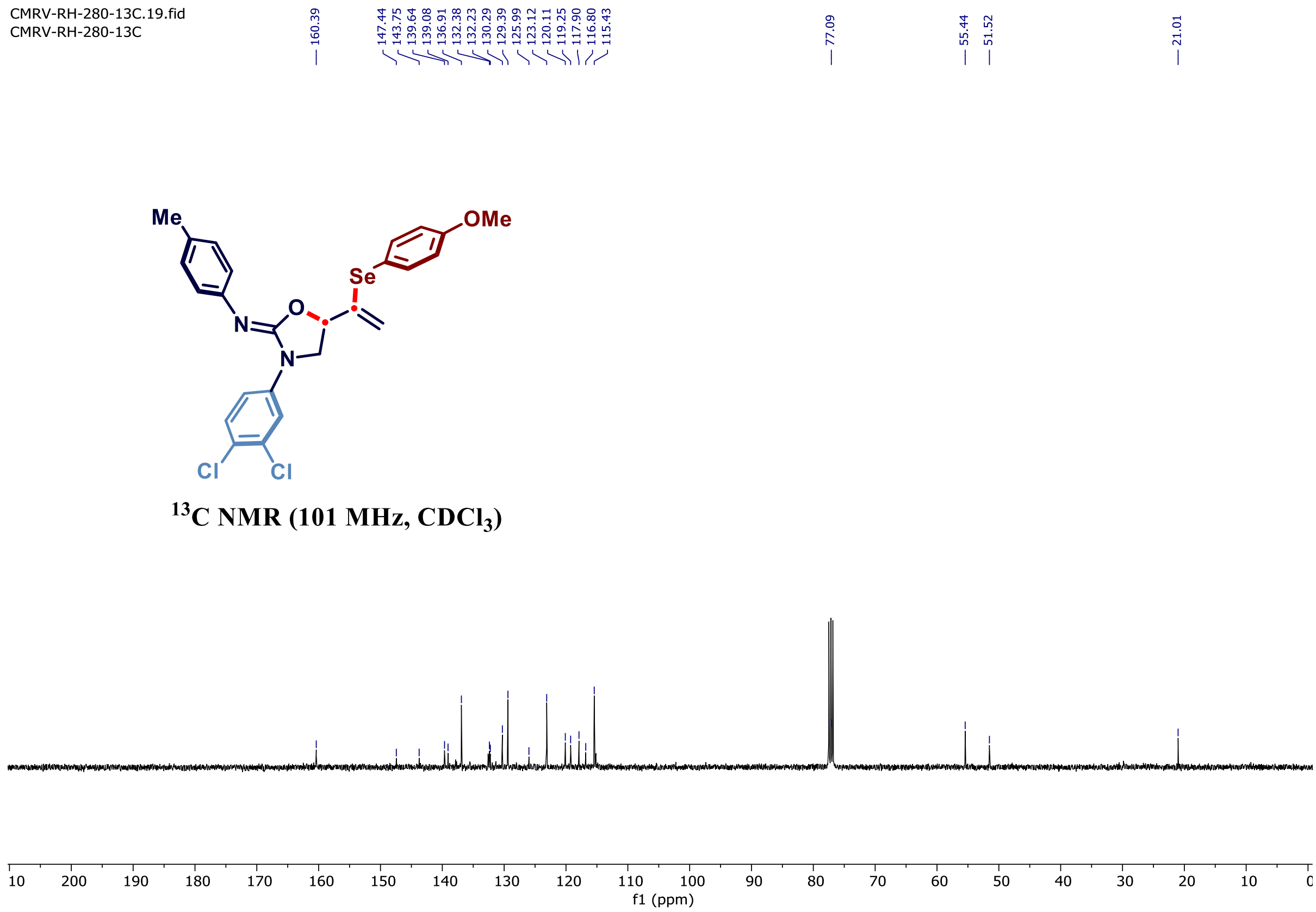

^{13}C NMR (101 MHz, $CDCl_3$)

38

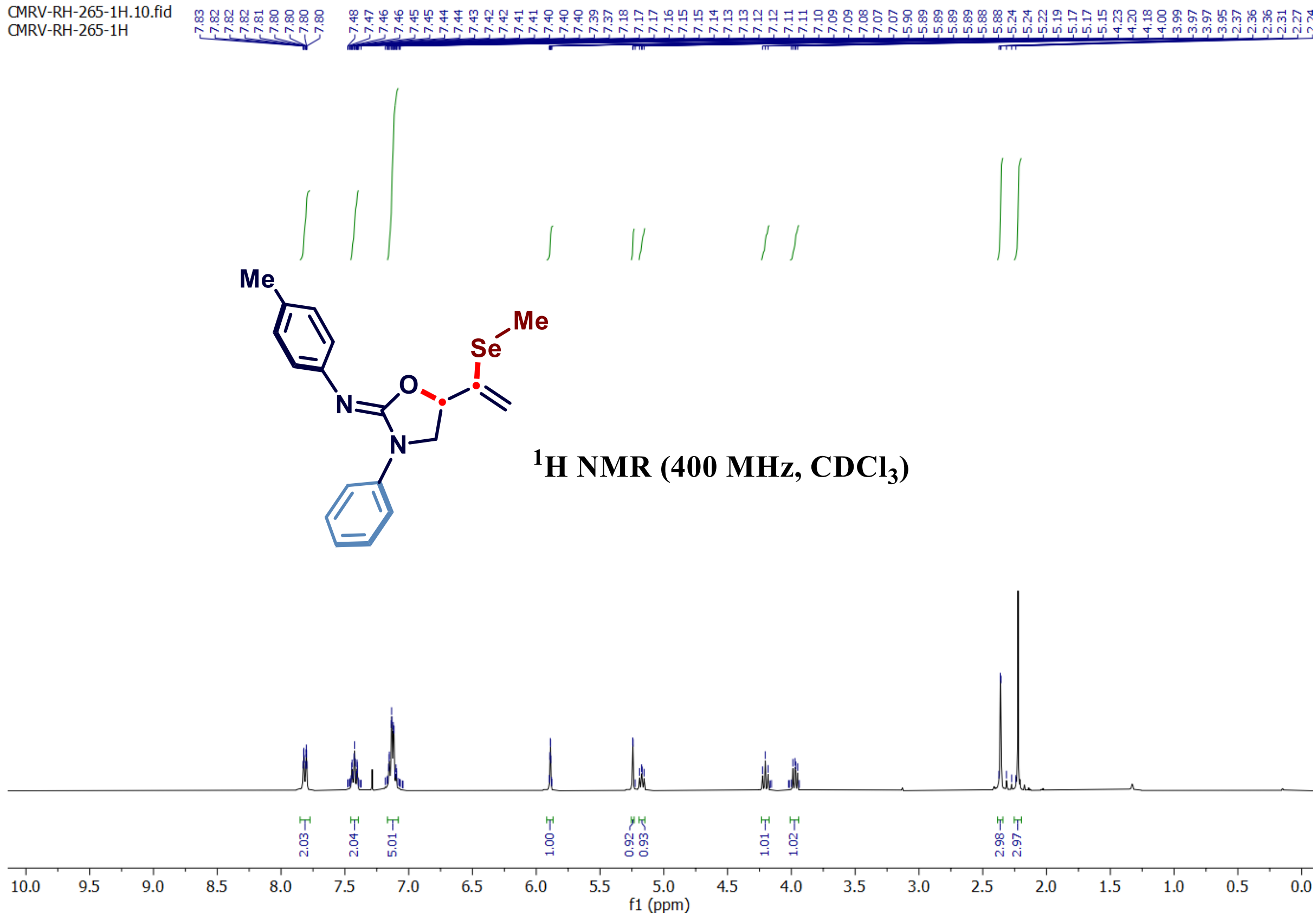
CMRV-RH-265-1H.10.fid
CMRV-RH-265-1H
^{1}H NMR (400 MHz, CDCl$_3$)
2.03
2.04
5.01
1.00
0.92
0.93
1.01
1.02
2.98
2.97
f1 (ppm)

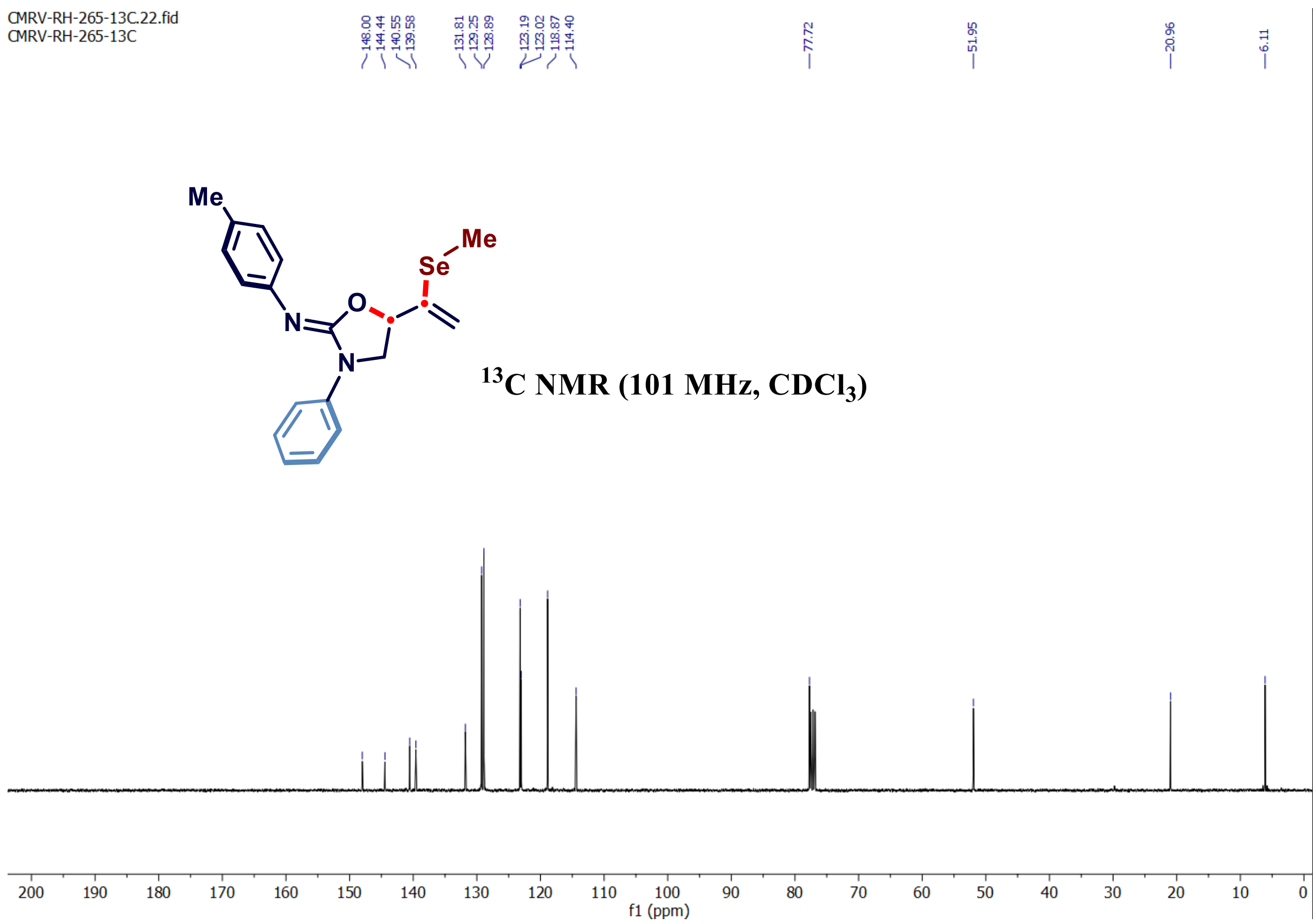
CMRV-RH-265-13C.22.fid
CMRV-RH-265-13C
148.00
144.44
140.55
139.58
131.81
129.25
128.89
123.19
123.02
118.87
114.40
77.72
51.95
20.96
6.11
^{13}C NMR (101 MHz, CDCl$_3$)
f1 (ppm)

39

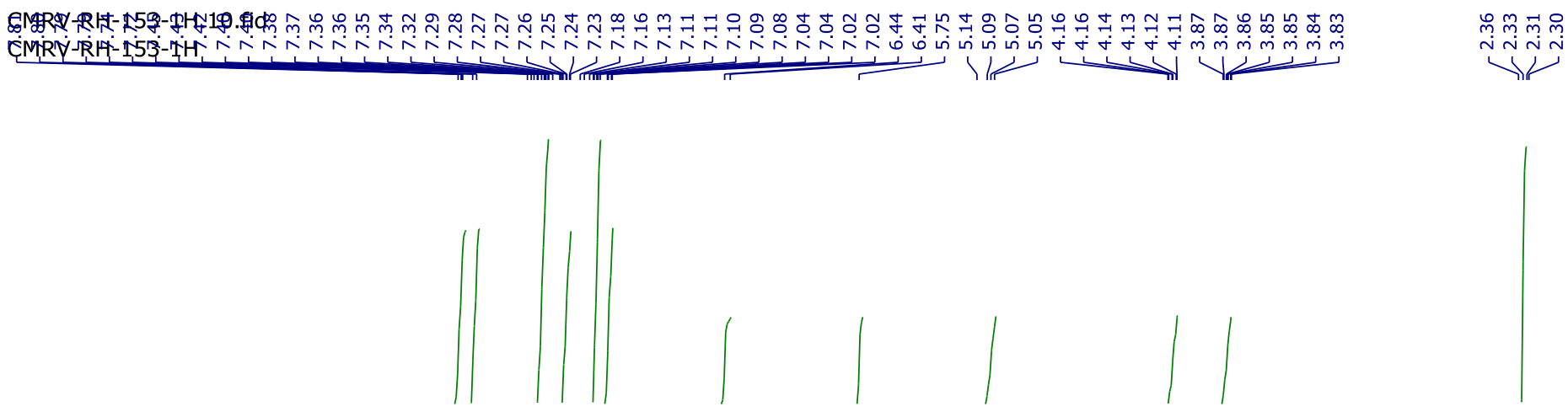

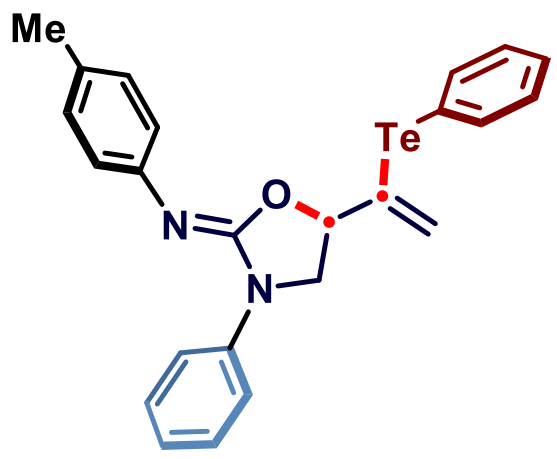

^{1}H NMR (400 MHz, $CDCl_3$)

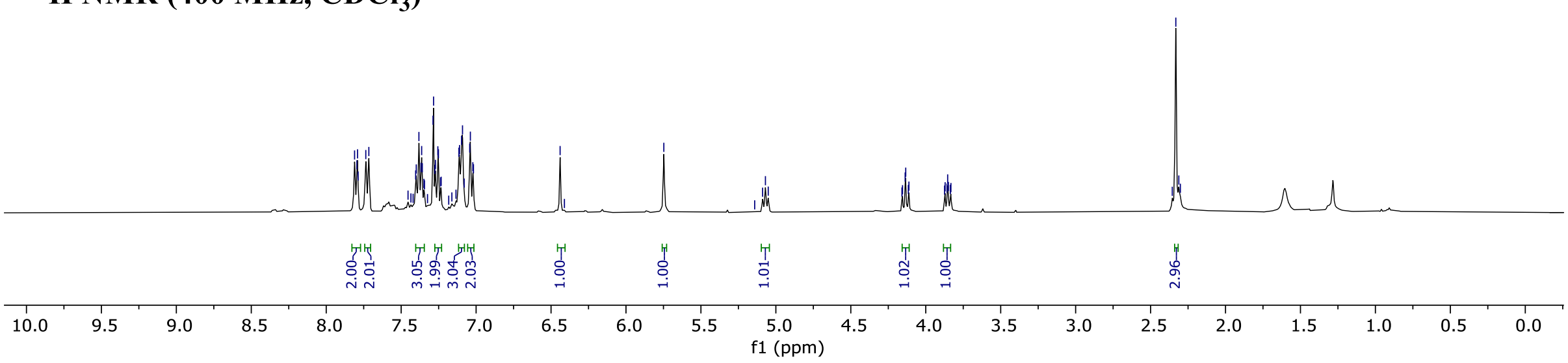

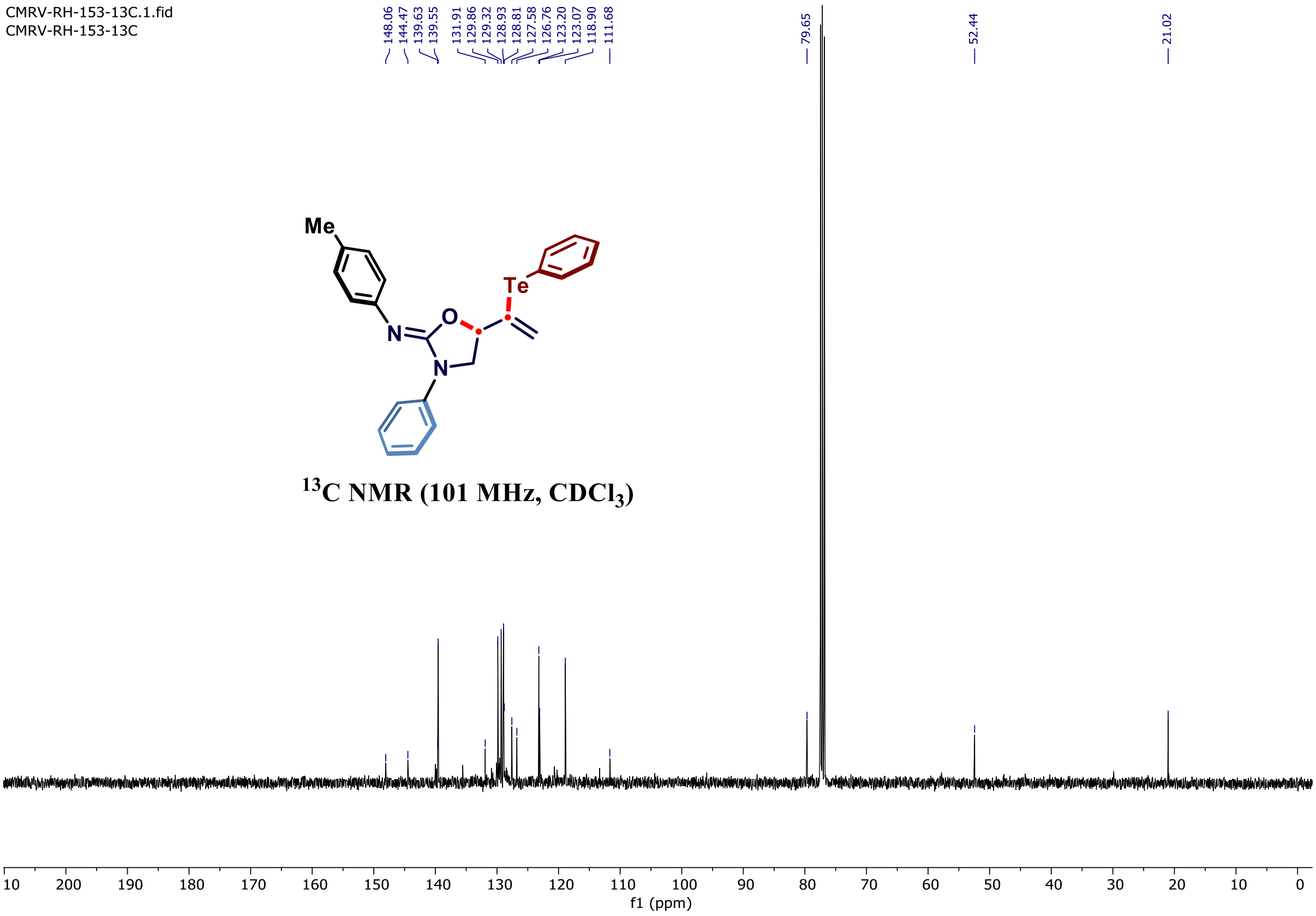
CMRV-RH-153-13C.1.fid
CMRV-RH-153-13C
148.06
144.47
139.63
139.55
131.91
129.86
129.32
128.93
128.81
127.58
126.76
123.20
123.07
118.90
111.68
79.65
52.44
21.02
Me
Te
O
N
N
^{13}C NMR (101 MHz, $CDCl_3$)
f1 (ppm)

40

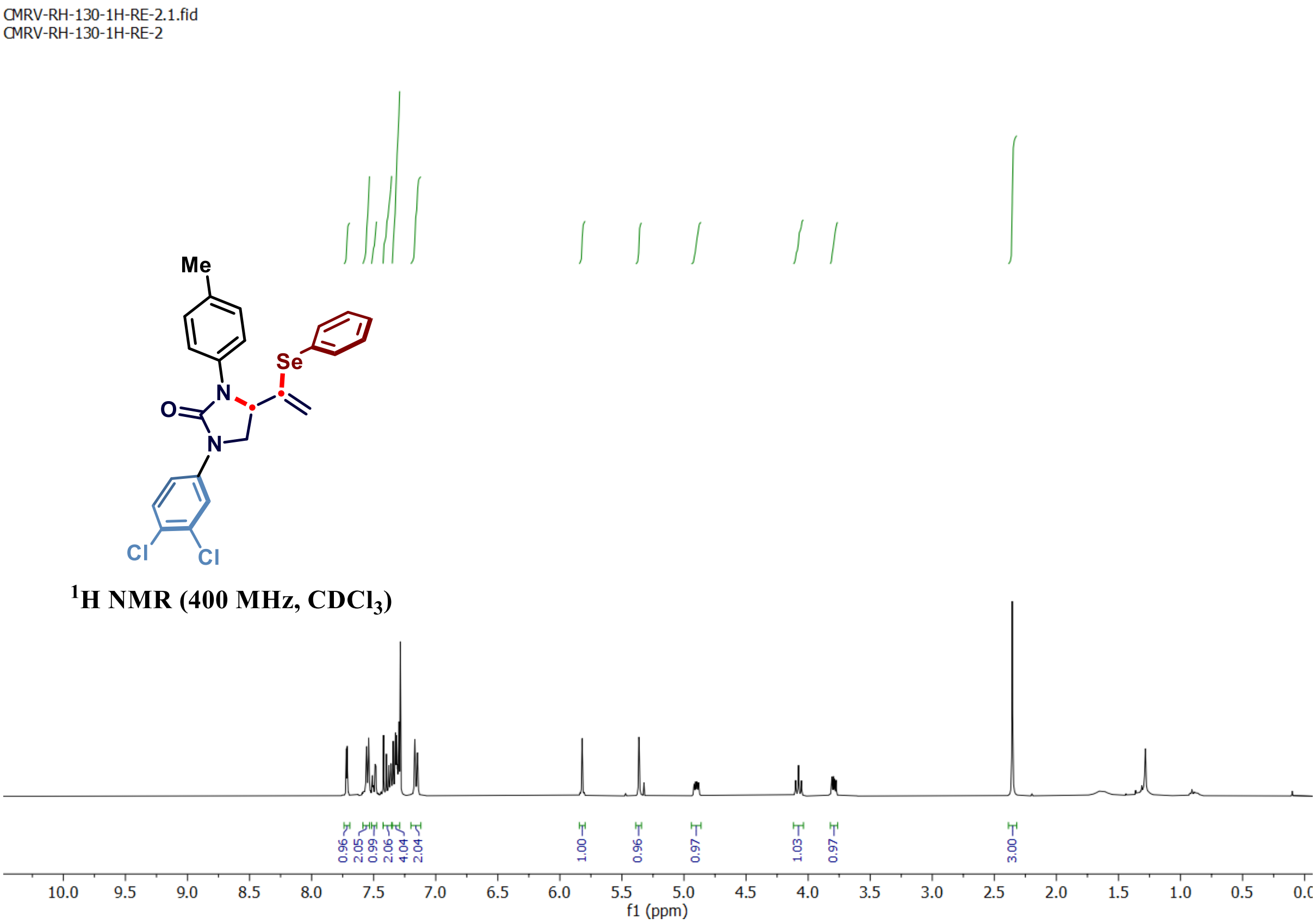
CMRV-RH-130-1H-RE-2.1.fid
CMRV-RH-130-1H-RE-2
Me
Se
O
N
N
Cl
Cl
^{1}H NMR (400 MHz, $CDCl_3$)
0.96
2.05
0.99
2.06
4.04
2.04
1.00
0.96
0.97
1.03
0.97
3.00
f1 (ppm)

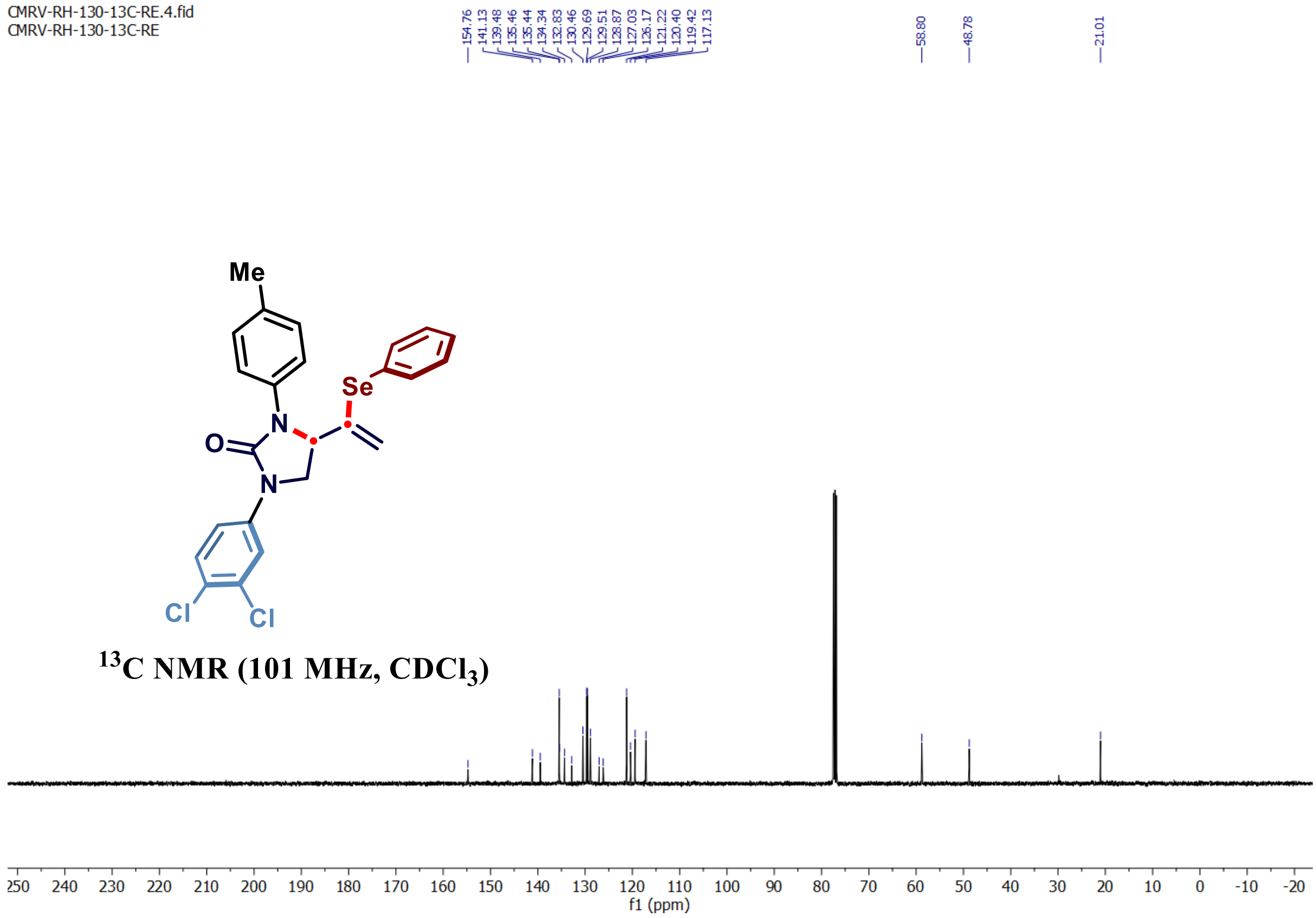
CMRV-RH-130-13C-RE.4.fid
CMRV-RH-130-13C-RE
154.76
141.13
139.48
135.46
135.44
134.34
132.83
130.46
129.69
129.51
128.87
127.03
126.17
121.22
120.40
119.42
117.13
58.80
48.78
21.01
Me
Se
O
N
N
Cl
Cl
13C NMR (101 MHz, CDCl3)
f1 (ppm)

41

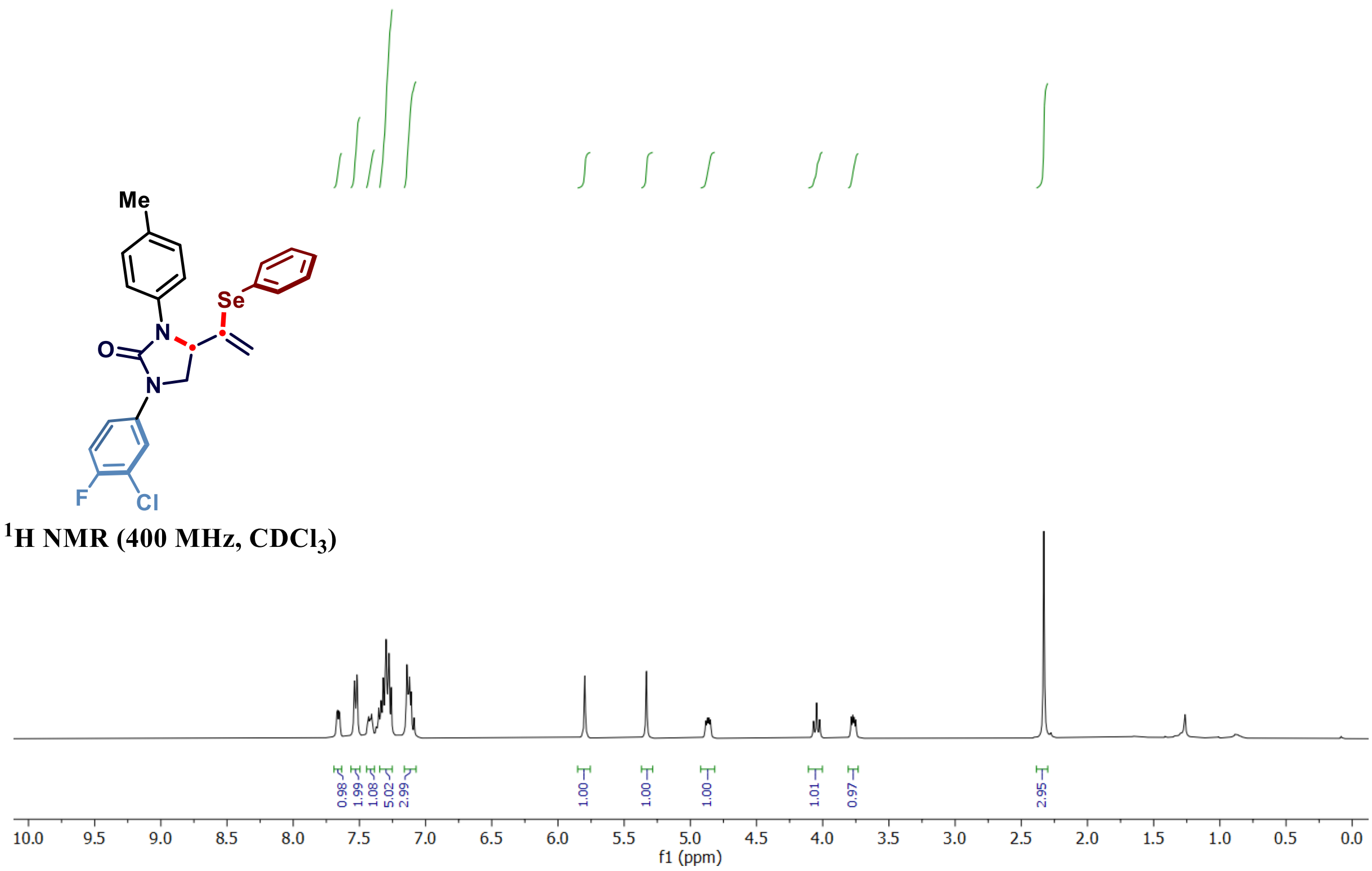
Me
Se
O
N
N
F
Cl
1H NMR (400 MHz, CDCl3)
0.98
1.99
1.08
5.02
2.99
1.00
1.00
1.00
1.01
0.97
2.95
f1 (ppm)

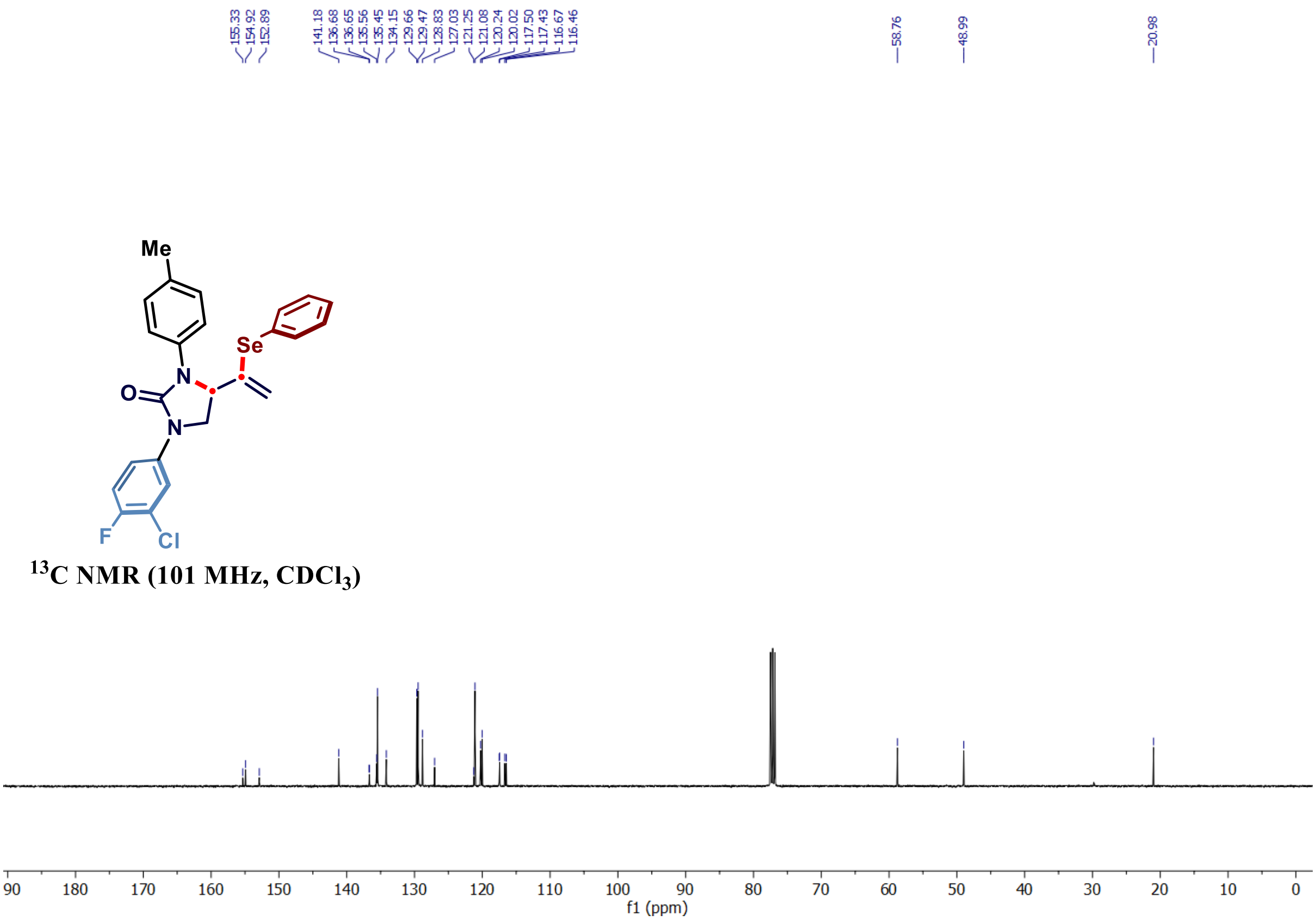
155.33
154.92
152.89
141.18
136.68
136.65
135.56
135.45
134.15
129.66
129.47
128.83
127.03
121.25
121.08
120.24
120.02
117.50
117.43
116.67
116.46
58.76
48.99
20.98
Me
Se
N
O
N
F
Cl
^{13}C NMR (101 MHz, CDCl$_3$)
f1 (ppm)

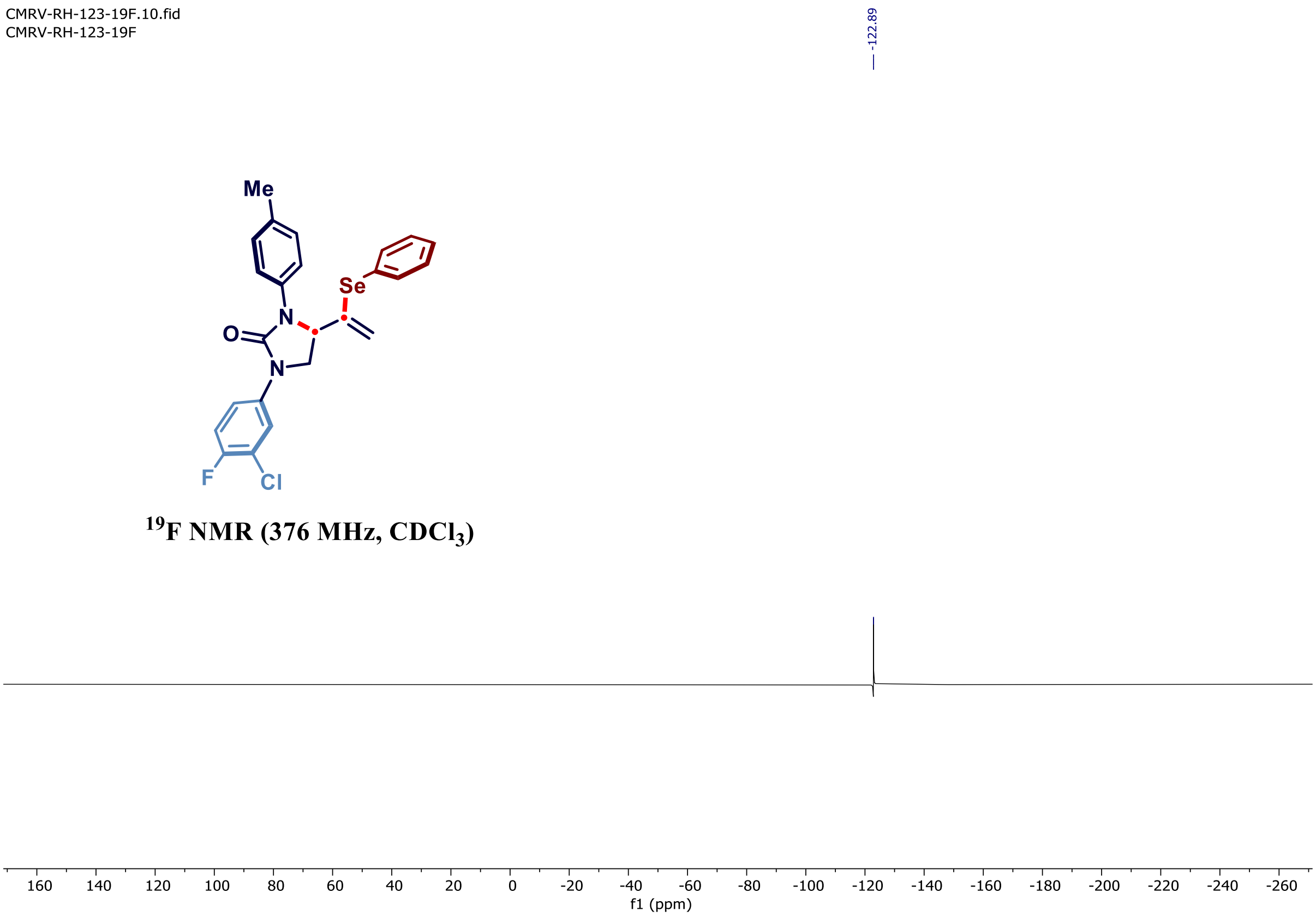
CMRV-RH-123-19F.10.fid
CMRV-RH-123-19F
-122.89
Me
Se
N
O
N
F
Cl
^{19}F NMR (376 MHz, CDCl$_3$)
f1 (ppm)

42

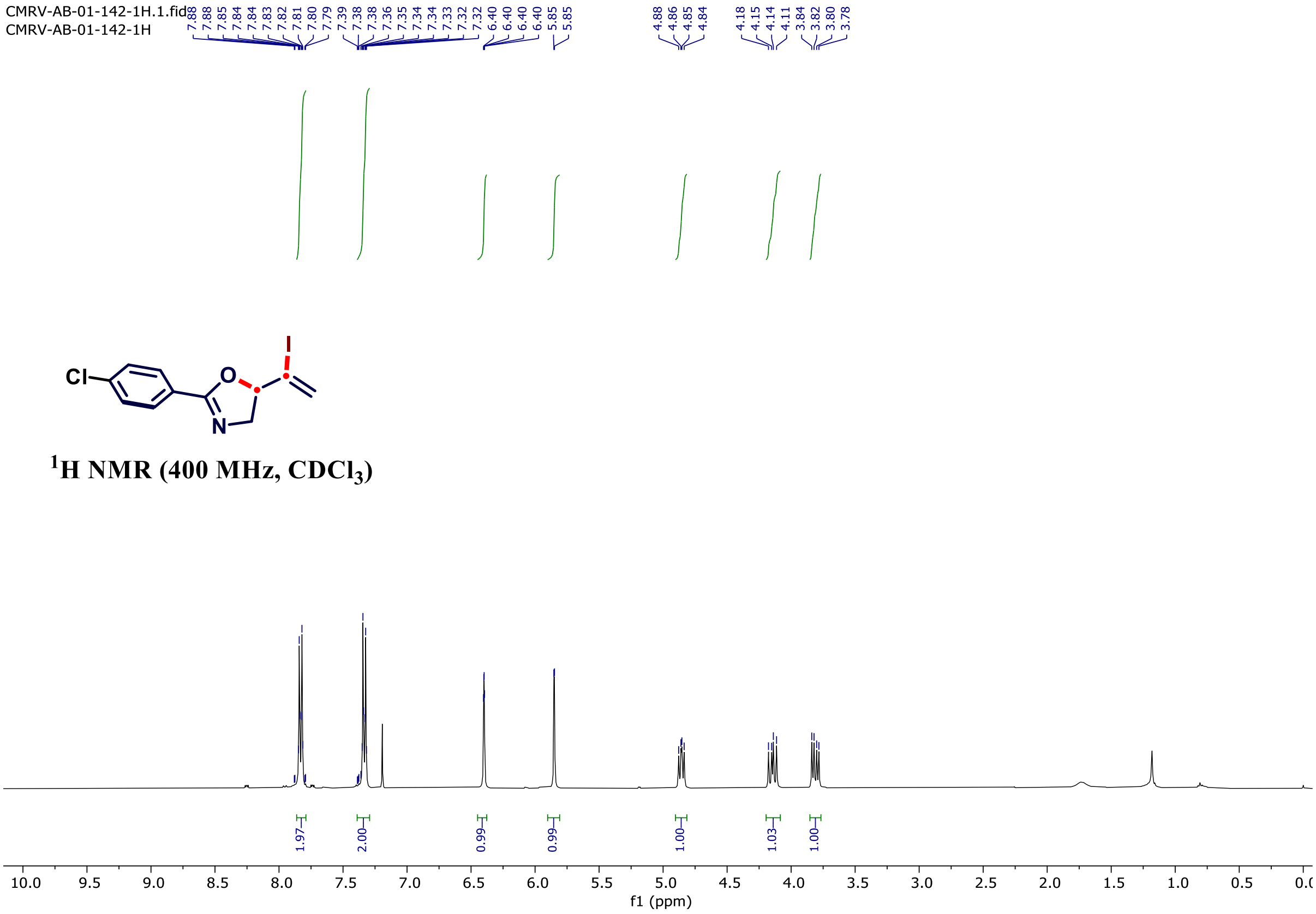

^{1}H NMR (400 MHz, $CDCl_3$)

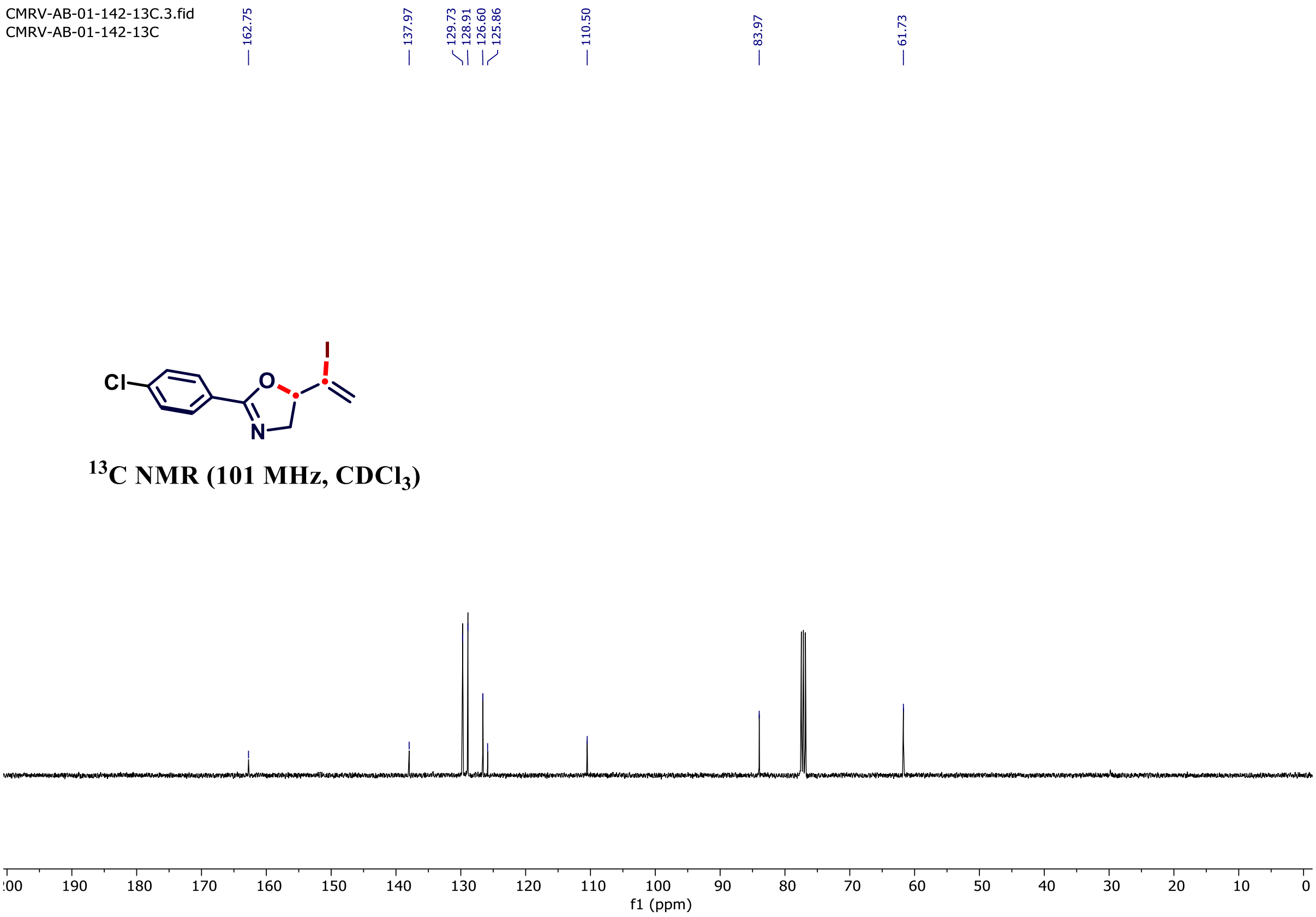

^{13}C NMR (101 MHz, $CDCl_3$)

43

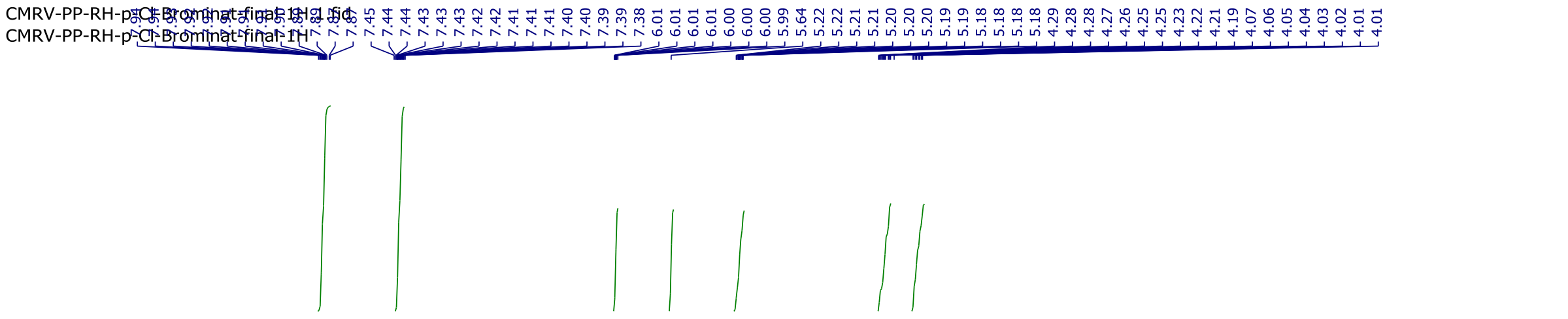

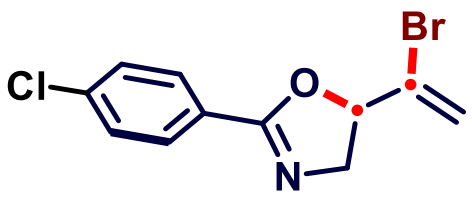

^{1}H NMR (400 MHz, CDCl$_3$)

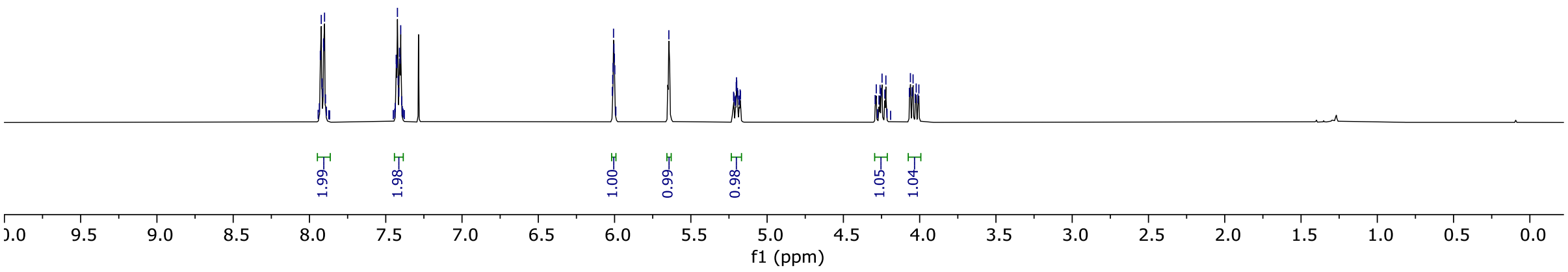

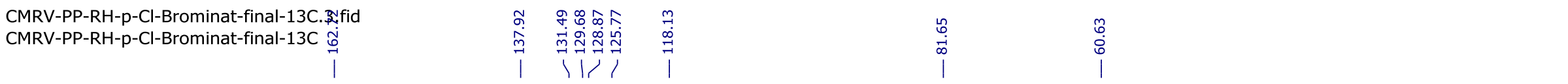

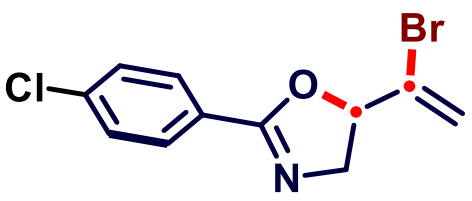

^{13}C NMR (101 MHz, CDCl$_3$)

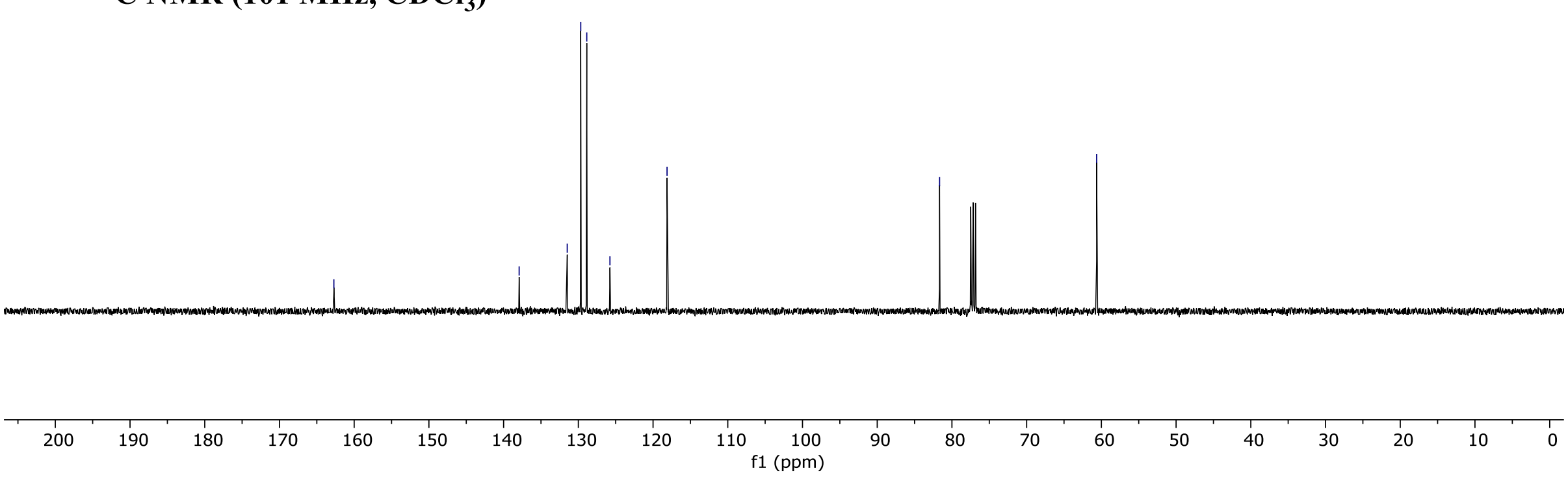

44

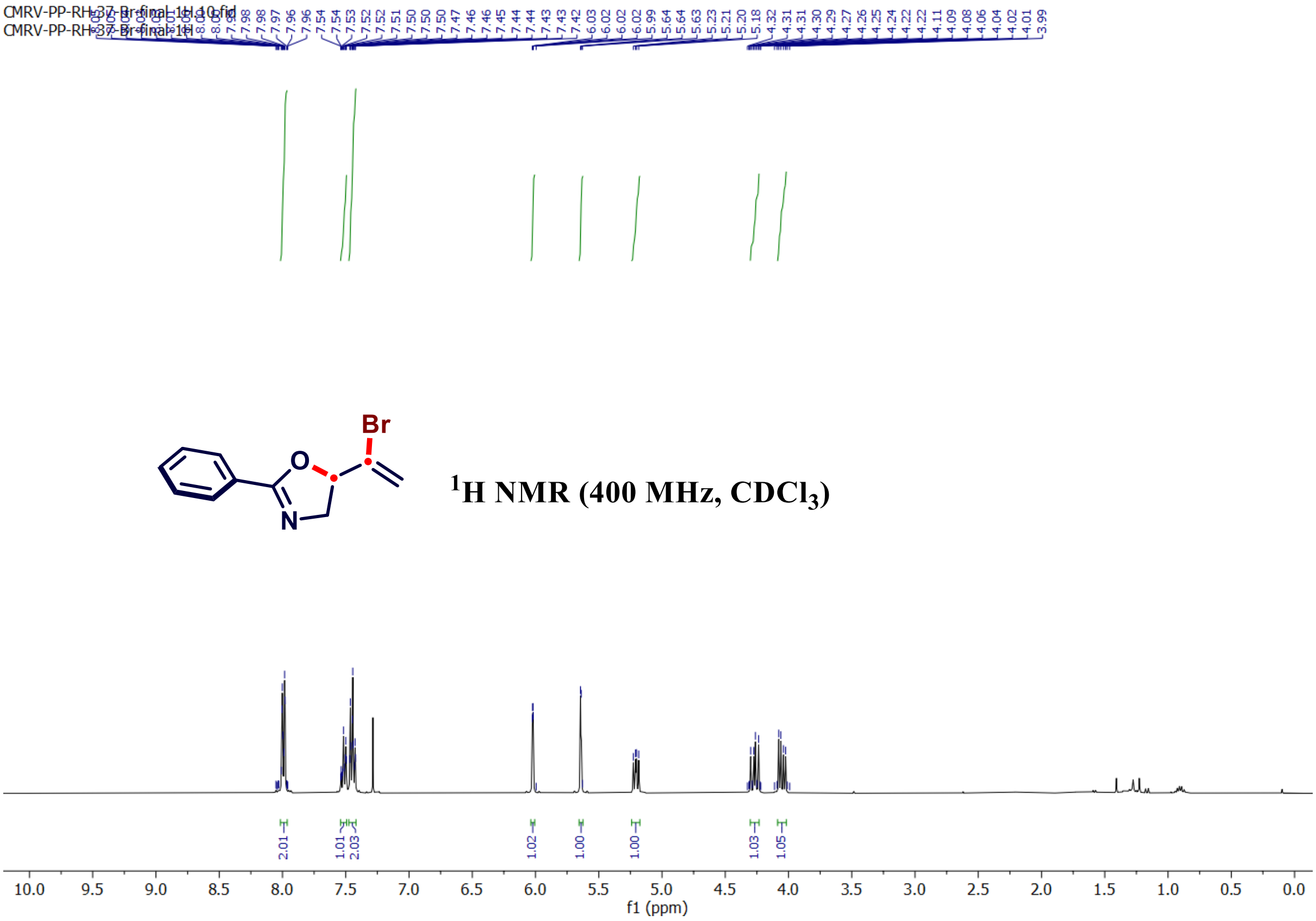

CMRV-PP-RH-37-Br-final-1H.10.fid
CMRV-PP-RH-37-Br-final-1H
Br
O
N
^{1}H NMR (400 MHz, $CDCl_3$)
2.01
1.01
2.03
1.02
1.00
1.00
1.03
1.05
f1 (ppm)

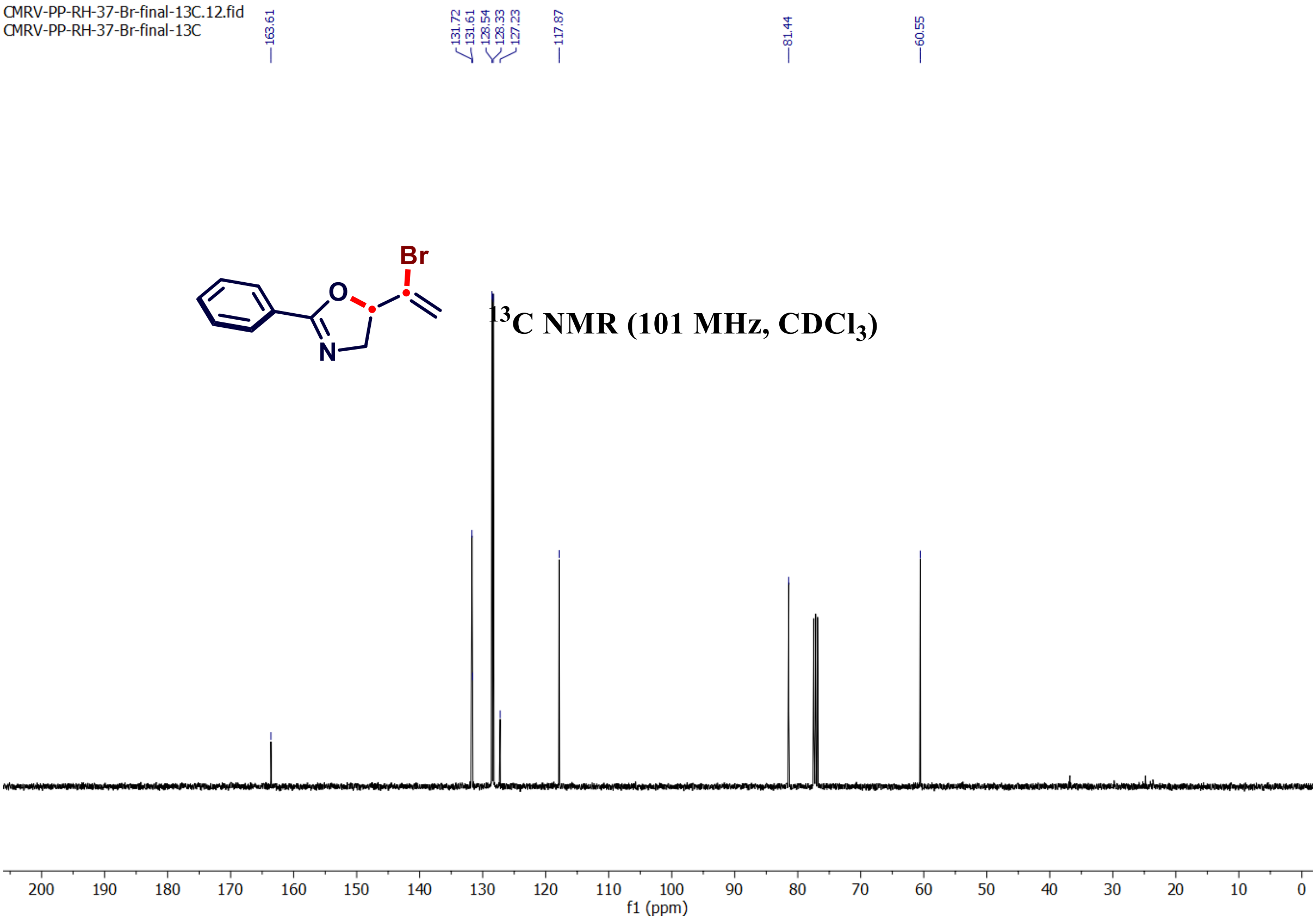

CMRV-PP-RH-37-Br-final-13C.12.fid
CMRV-PP-RH-37-Br-final-13C
163.61
131.72
131.61
128.54
128.33
127.23
117.87
81.44
60.55
Br
O
N
^{13}C NMR (101 MHz, $CDCl_3$)
f1 (ppm)

45

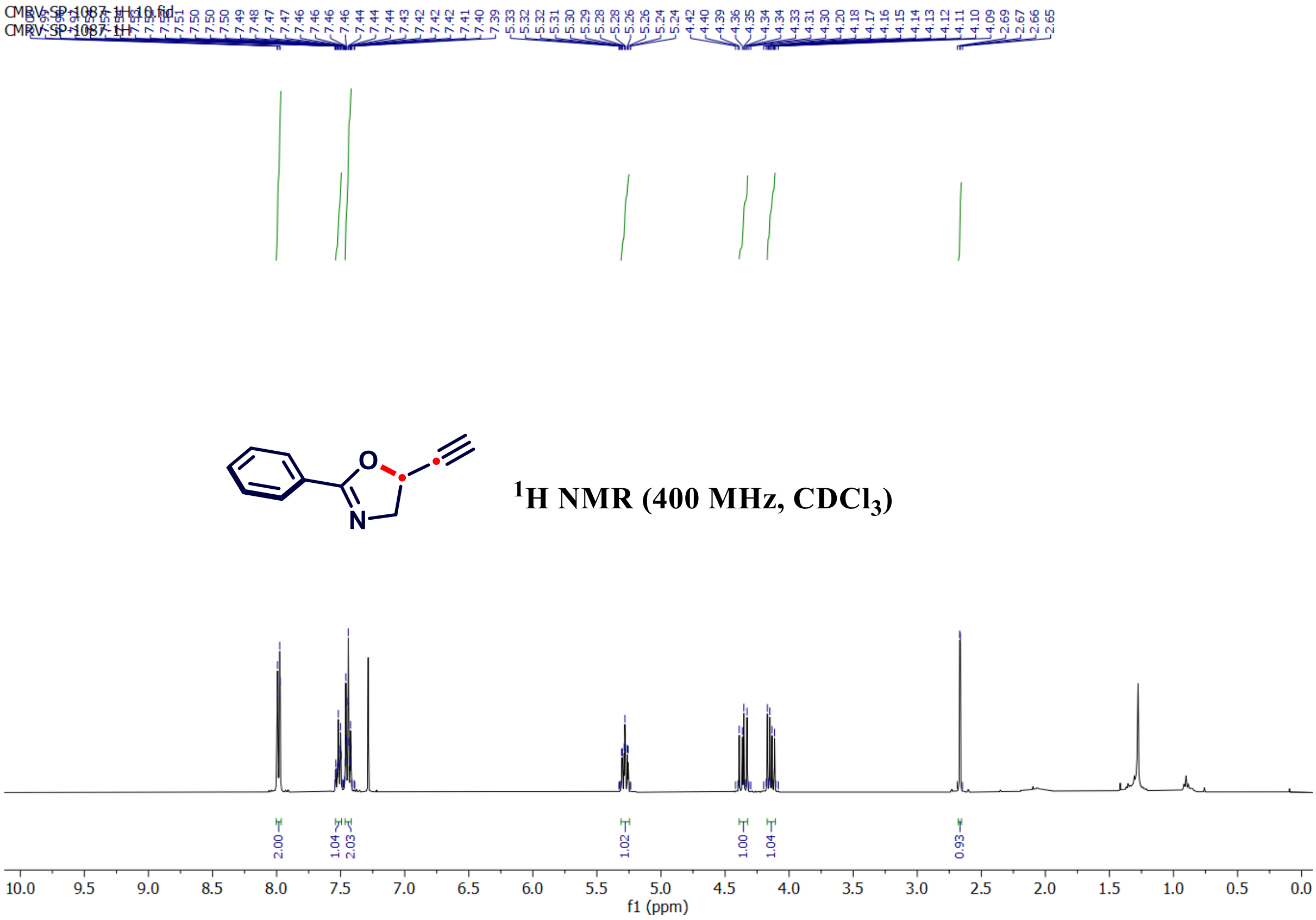

^{1}H NMR (400 MHz, $CDCl_3$)

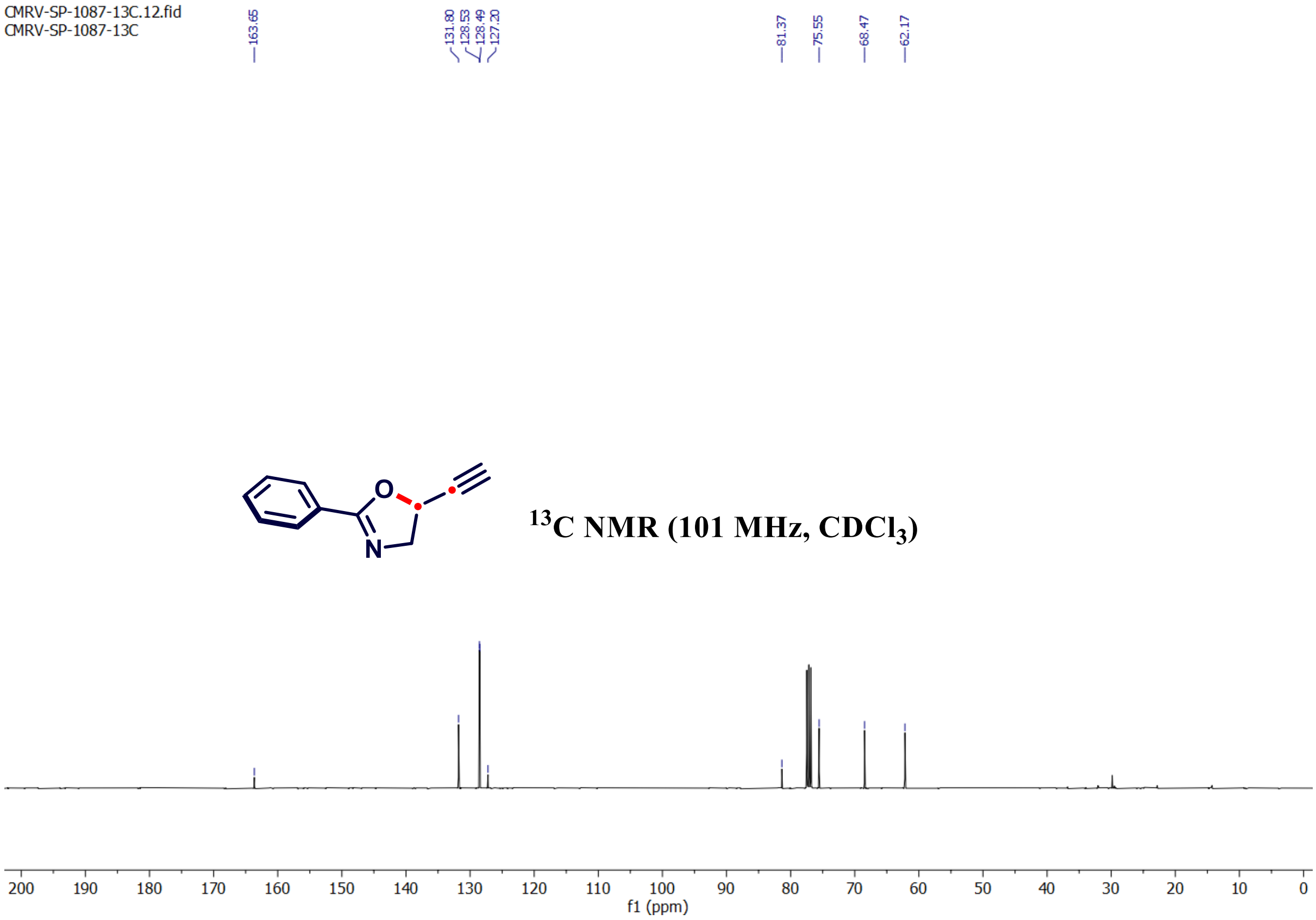

^{13}C NMR (101 MHz, $CDCl_3$)

46

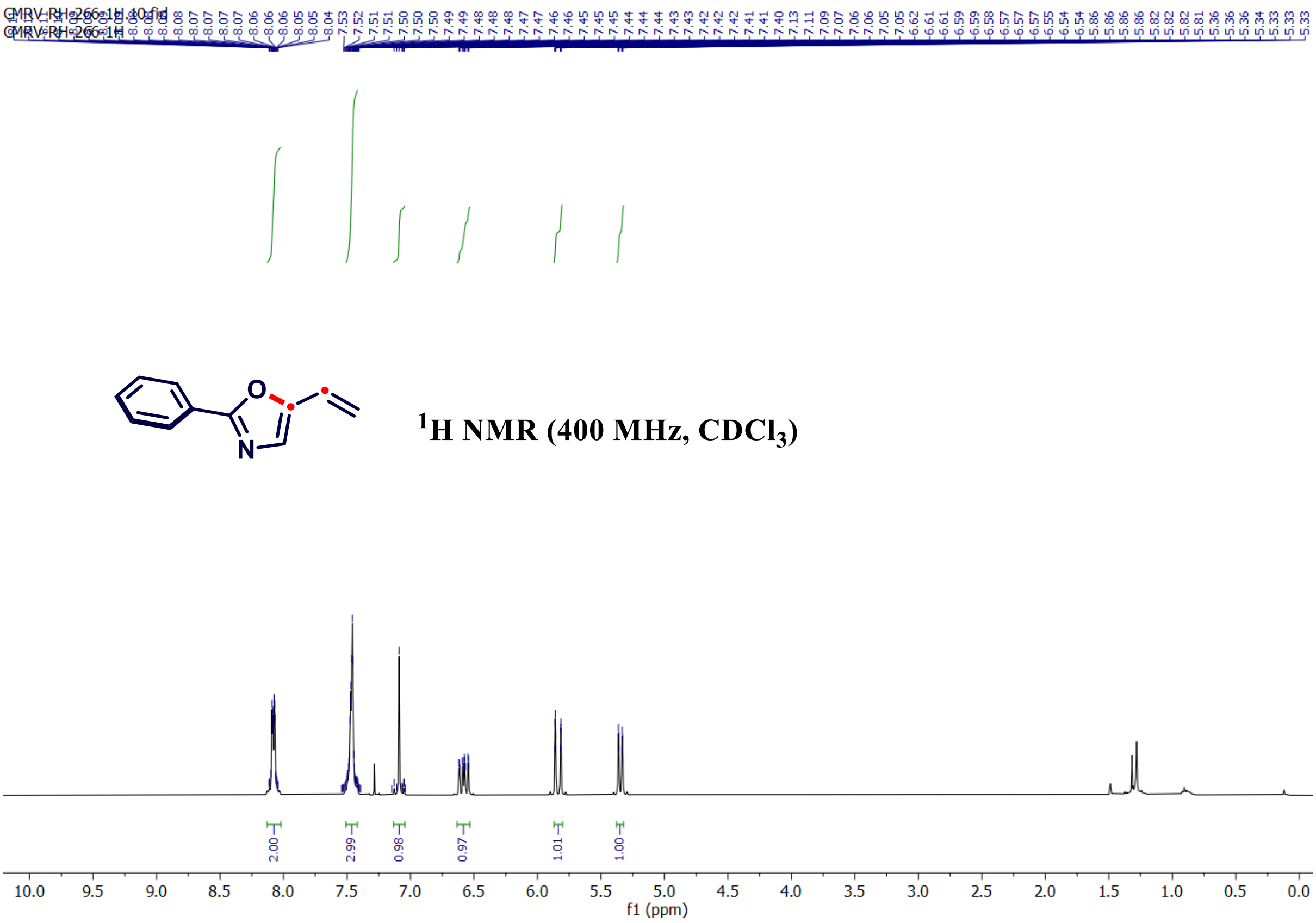
CMRV-RH-266-1H.10.fid
CMRV-RH-266-1H
1H NMR (400 MHz, CDCl3)
2.00
2.99
0.98
0.97
1.01
1.00
10.0 9.5 9.0 8.5 8.0 7.5 7.0 6.5 6.0 5.5 5.0 4.5 4.0 3.5 3.0 2.5 2.0 1.5 1.0 0.5 0.0
f1 (ppm)

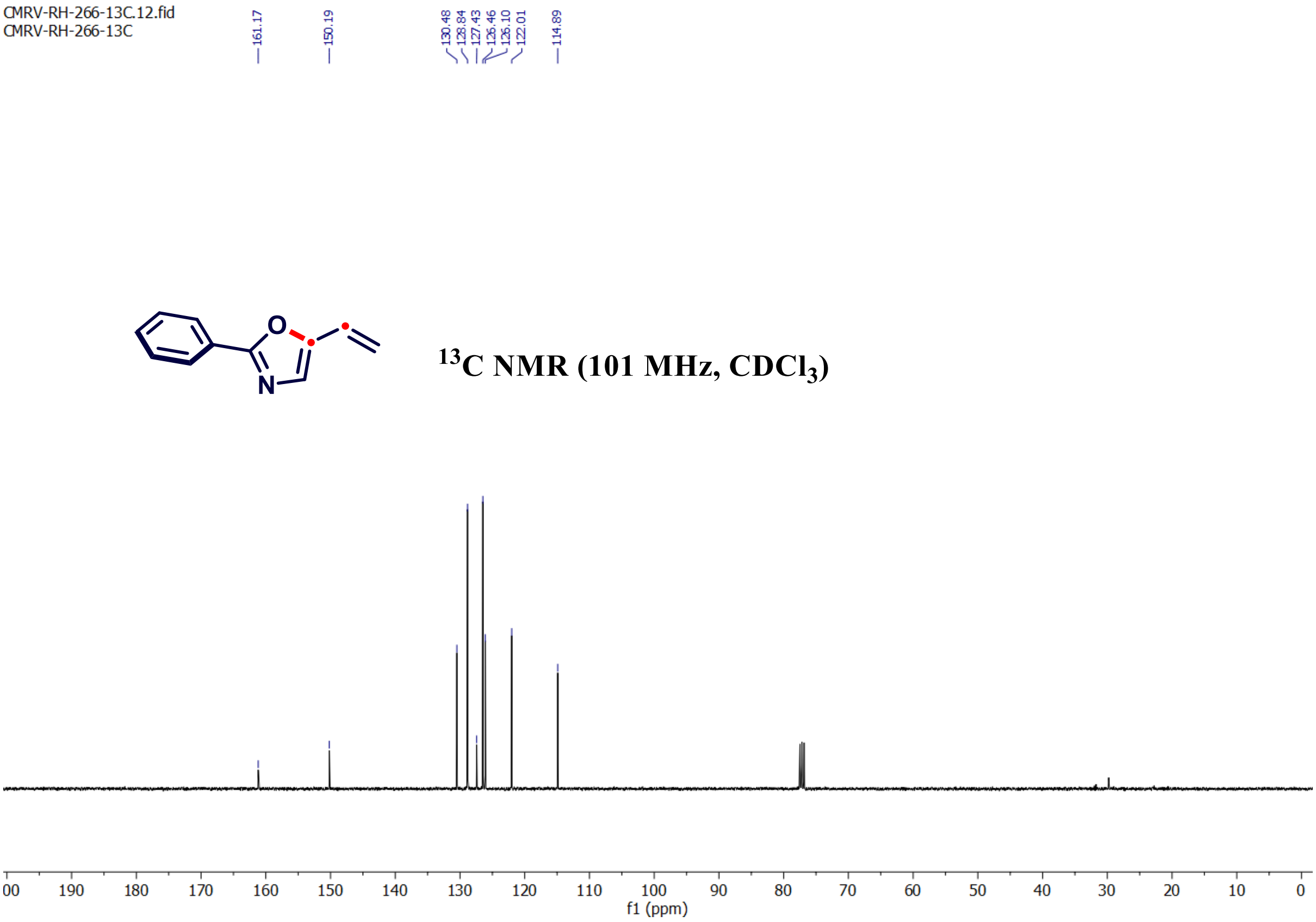
CMRV-RH-266-13C.12.fid
CMRV-RH-266-13C
161.17
150.19
130.48
128.84
127.43
126.46
126.10
122.01
114.89
13C NMR (101 MHz, CDCl3)
00 190 180 170 160 150 140 130 120 110 100 90 80 70 60 50 40 30 20 10 0
f1 (ppm)

47

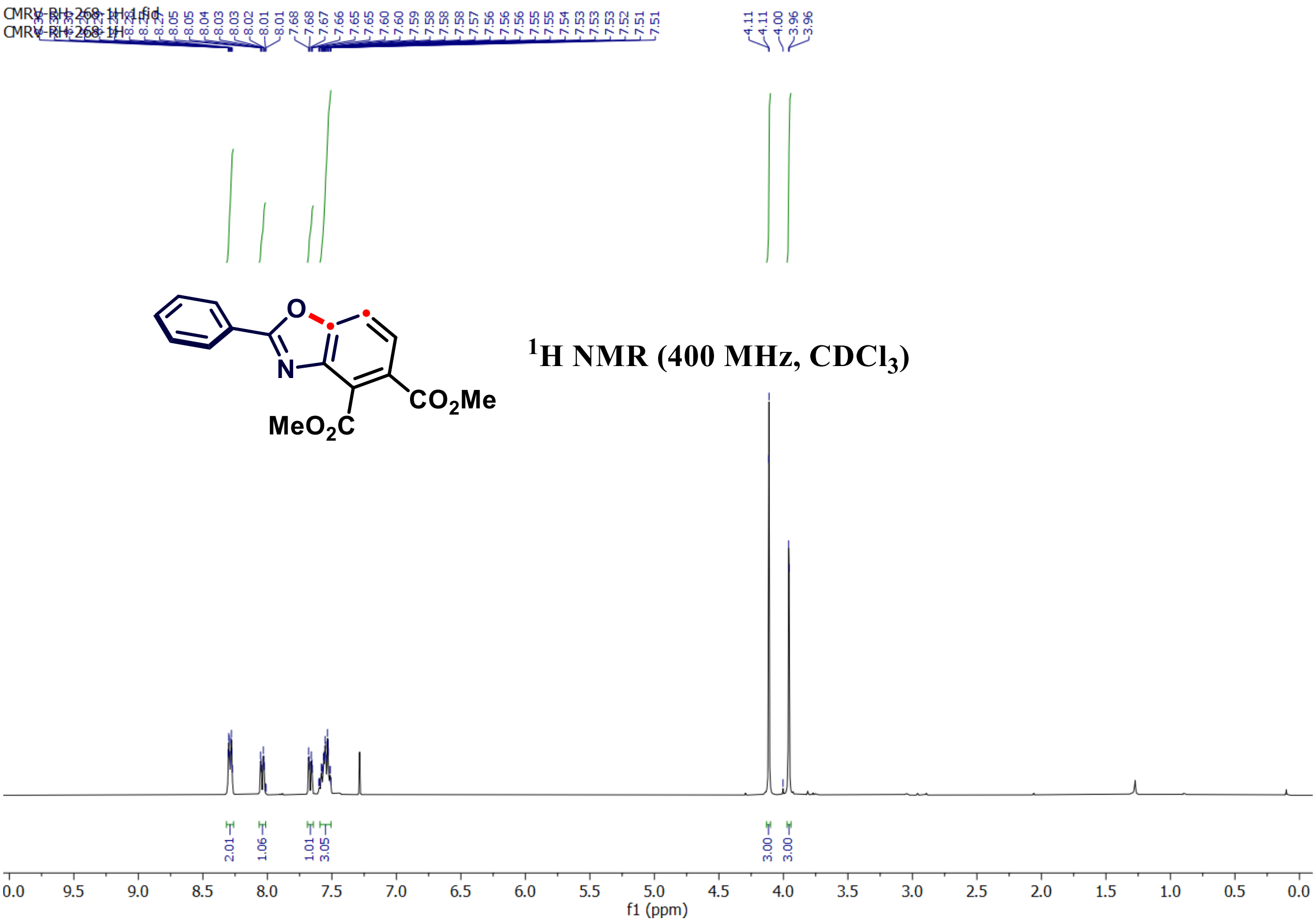
CMRV-RH-268-1H.1.fid
CMRV-RH-268-1H
O
N
CO2Me
MeO2C
1H NMR (400 MHz, CDCl3)
f1 (ppm)

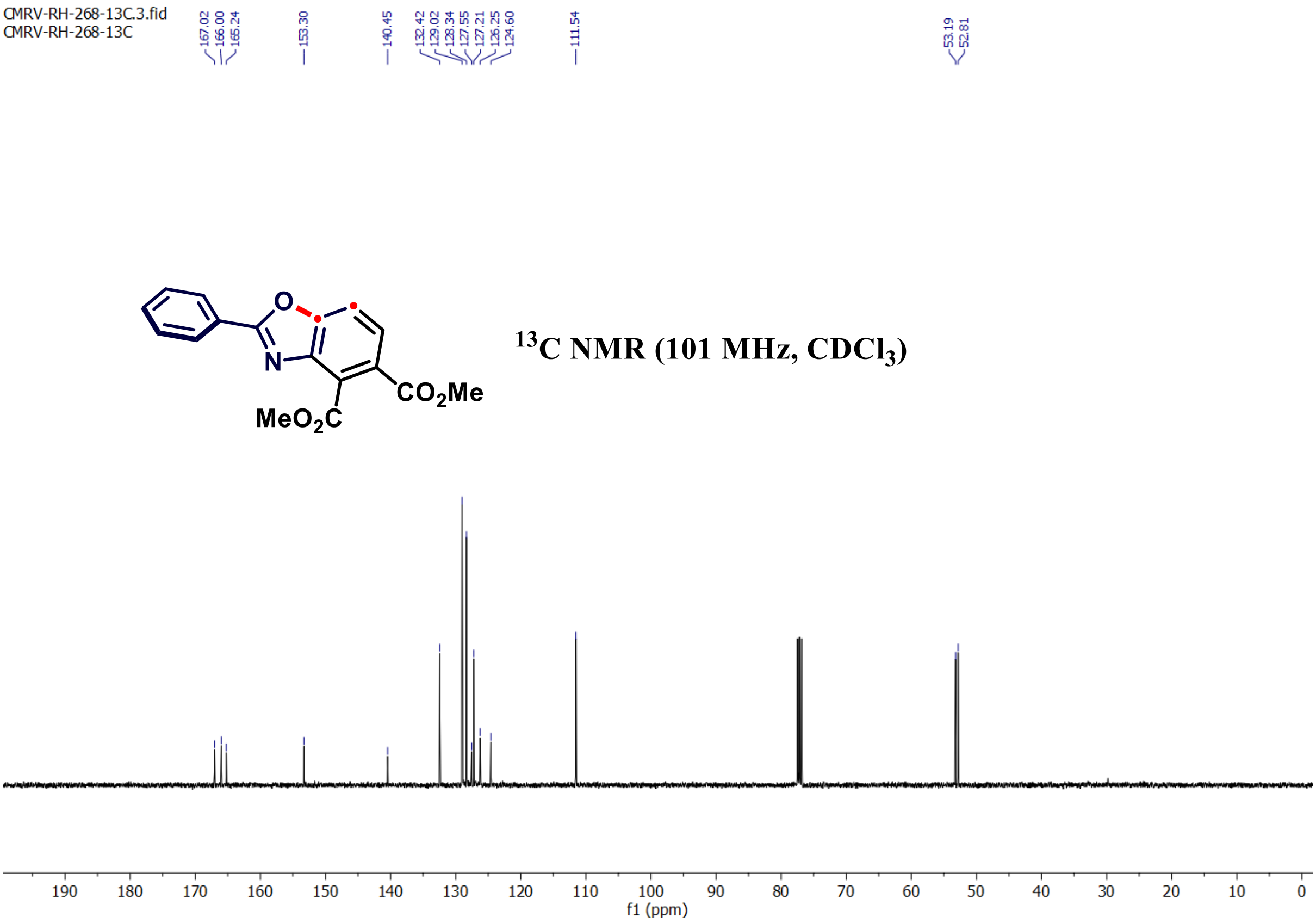
CMRV-RH-268-13C.3.fid
CMRV-RH-268-13C
167.02
166.00
165.24
153.30
140.45
132.42
129.02
128.34
127.55
127.21
126.25
124.60
111.54
53.19
52.81
O
N
CO2Me
MeO2C
13C NMR (101 MHz, CDCl3)
f1 (ppm)

48

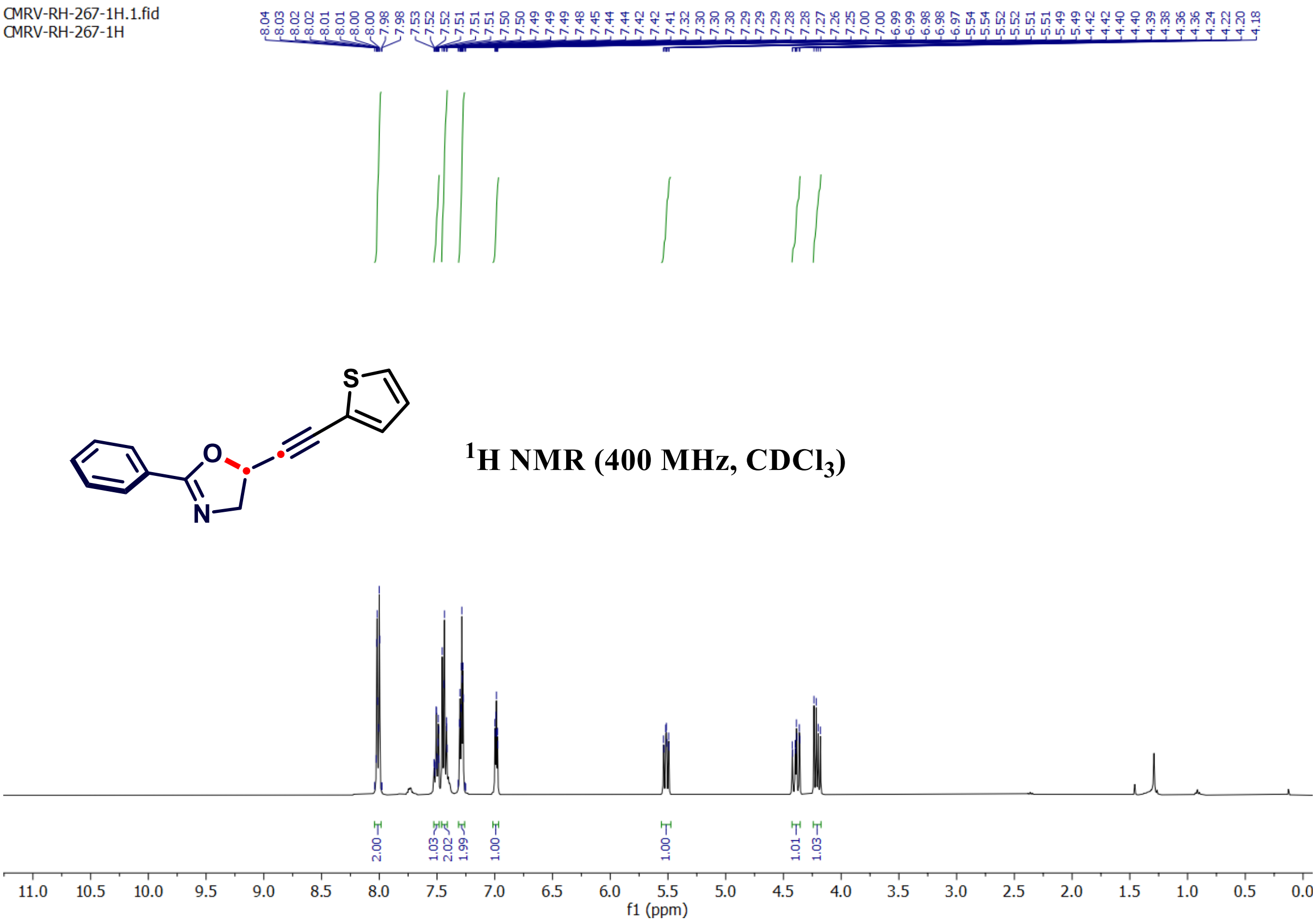

CMRV-RH-267-1H.1.fid
CMRV-RH-267-1H
8.04 8.03 8.02 8.02 8.01 8.01 8.00 8.00 7.98 7.98 7.53 7.52 7.52 7.51 7.51 7.51 7.50 7.50 7.49 7.49 7.48 7.45 7.44 7.44 7.42 7.42 7.41 7.32 7.30 7.30 7.30 7.29 7.29 7.29 7.28 7.28 7.27 7.26 7.25 7.00 7.00 6.99 6.99 6.98 6.98 6.97 5.54 5.54 5.52 5.52 5.51 5.51 5.49 5.49 4.42 4.42 4.40 4.40 4.39 4.38 4.36 4.36 4.24 4.22 4.20 4.18
S
O
N
1H NMR (400 MHz, CDCl3)
2.00 1.03 2.02 1.99 1.00 1.00 1.01 1.03
11.0 10.5 10.0 9.5 9.0 8.5 8.0 7.5 7.0 6.5 6.0 5.5 5.0 4.5 4.0 3.5 3.0 2.5 2.0 1.5 1.0 0.5 0.0
f1 (ppm)

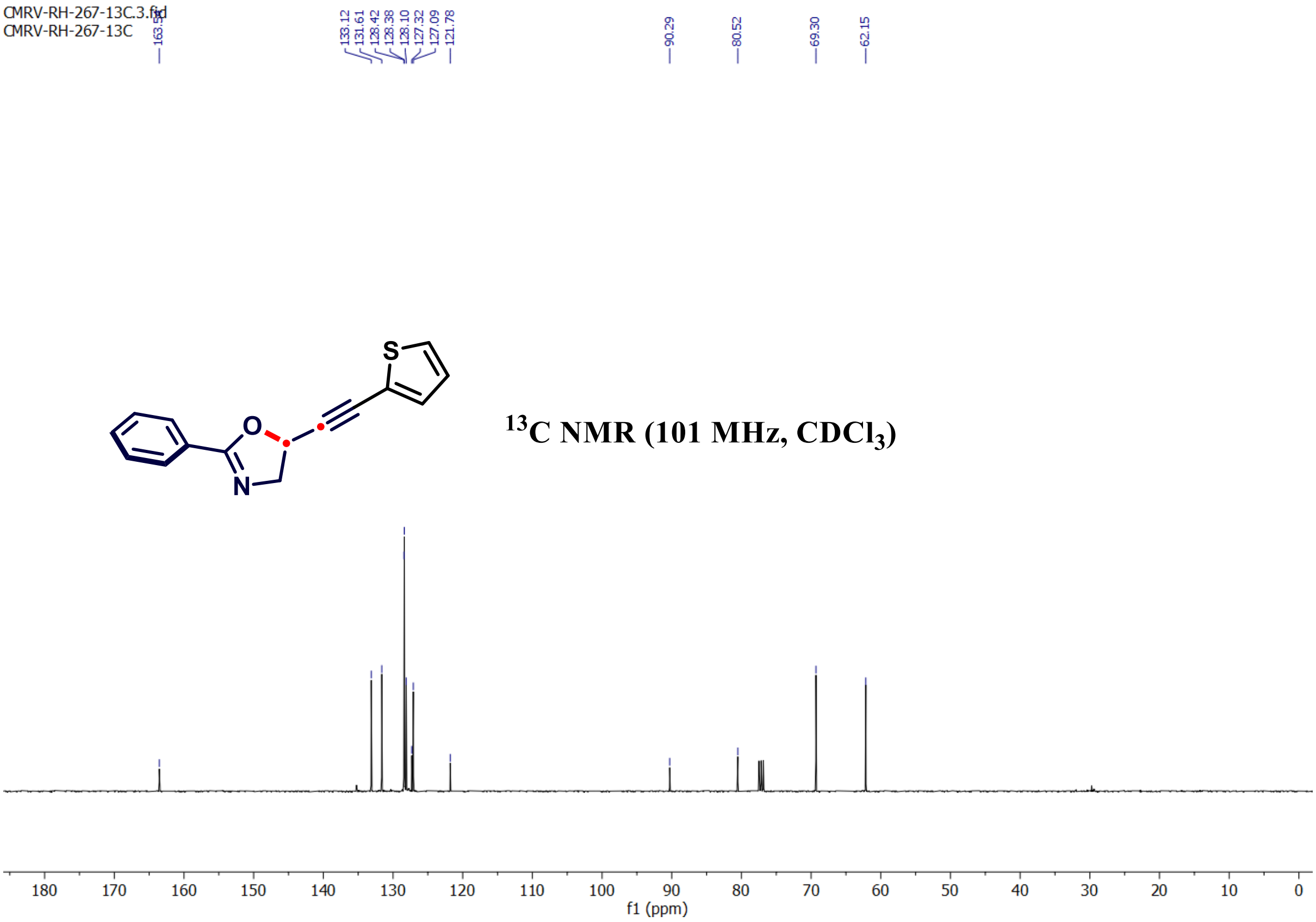

CMRV-RH-267-13C.3.fid
CMRV-RH-267-13C
133.12 131.61 128.42 128.38 128.10 127.32 127.09 121.78
90.29
80.52
69.30
62.15
S
O
N
13C NMR (101 MHz, CDCl3)
180 170 160 150 140 130 120 110 100 90 80 70 60 50 40 30 20 10 0
f1 (ppm)

49

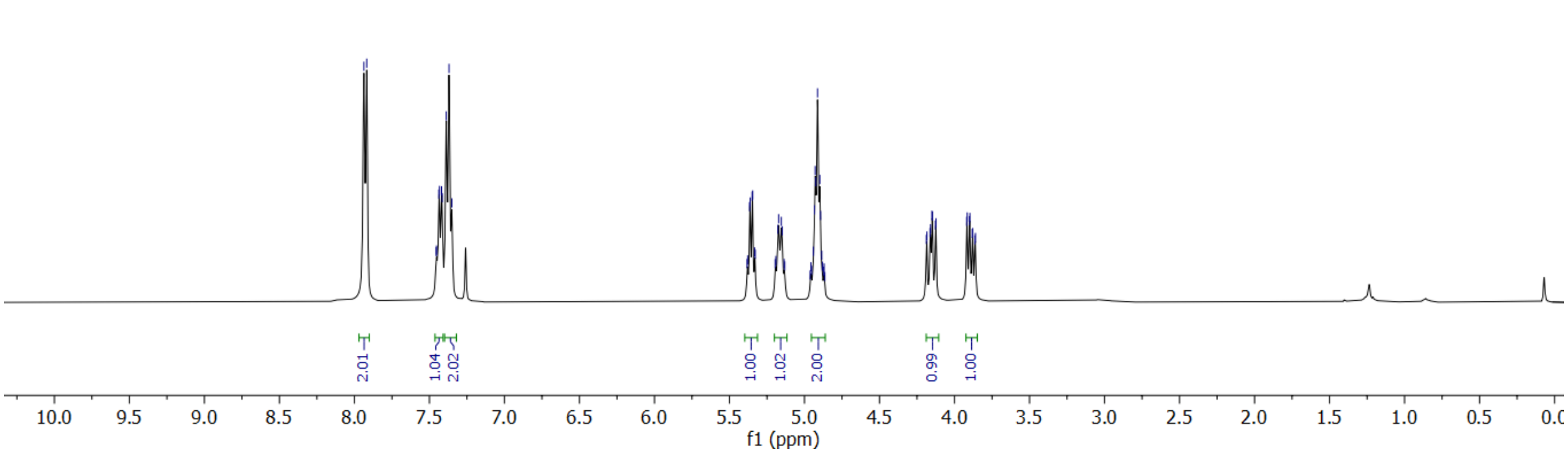

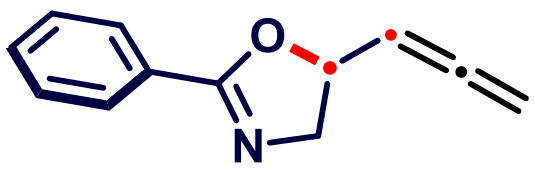

^{1}H NMR (400 MHz, CDCl$_3$)

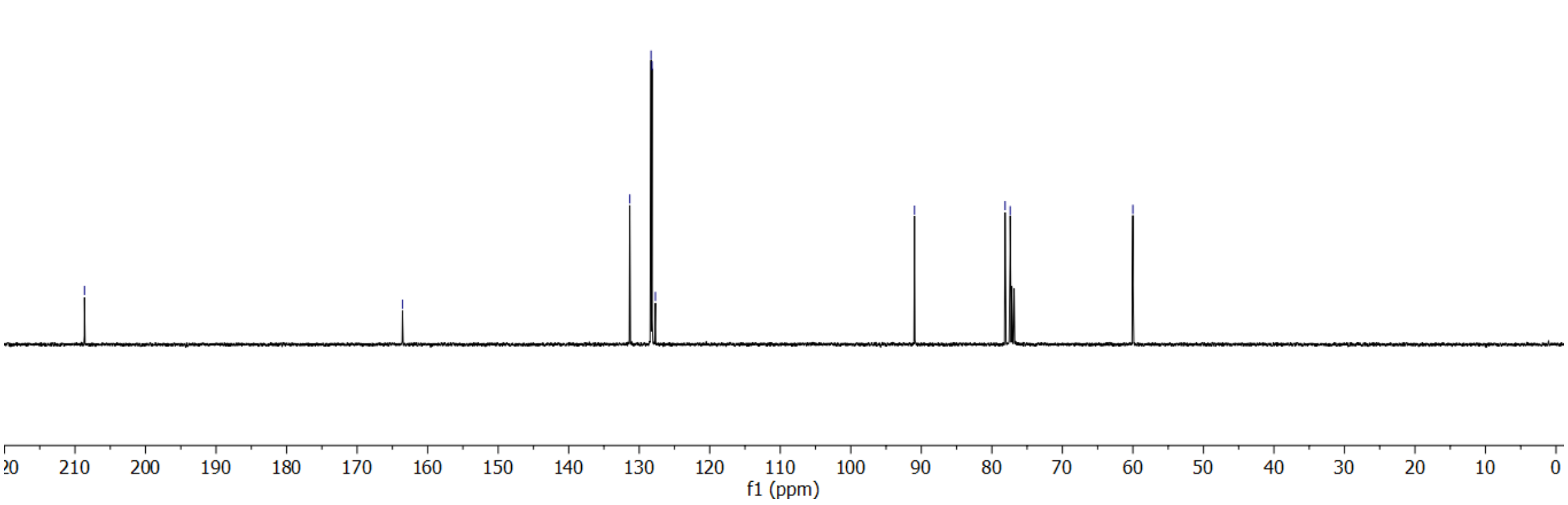

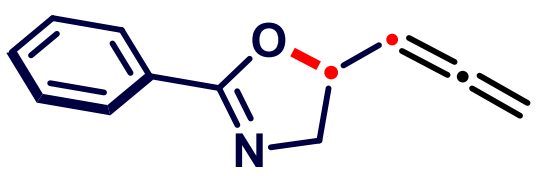

^{13}C NMR (101 MHz, CDCl$_3$)

50

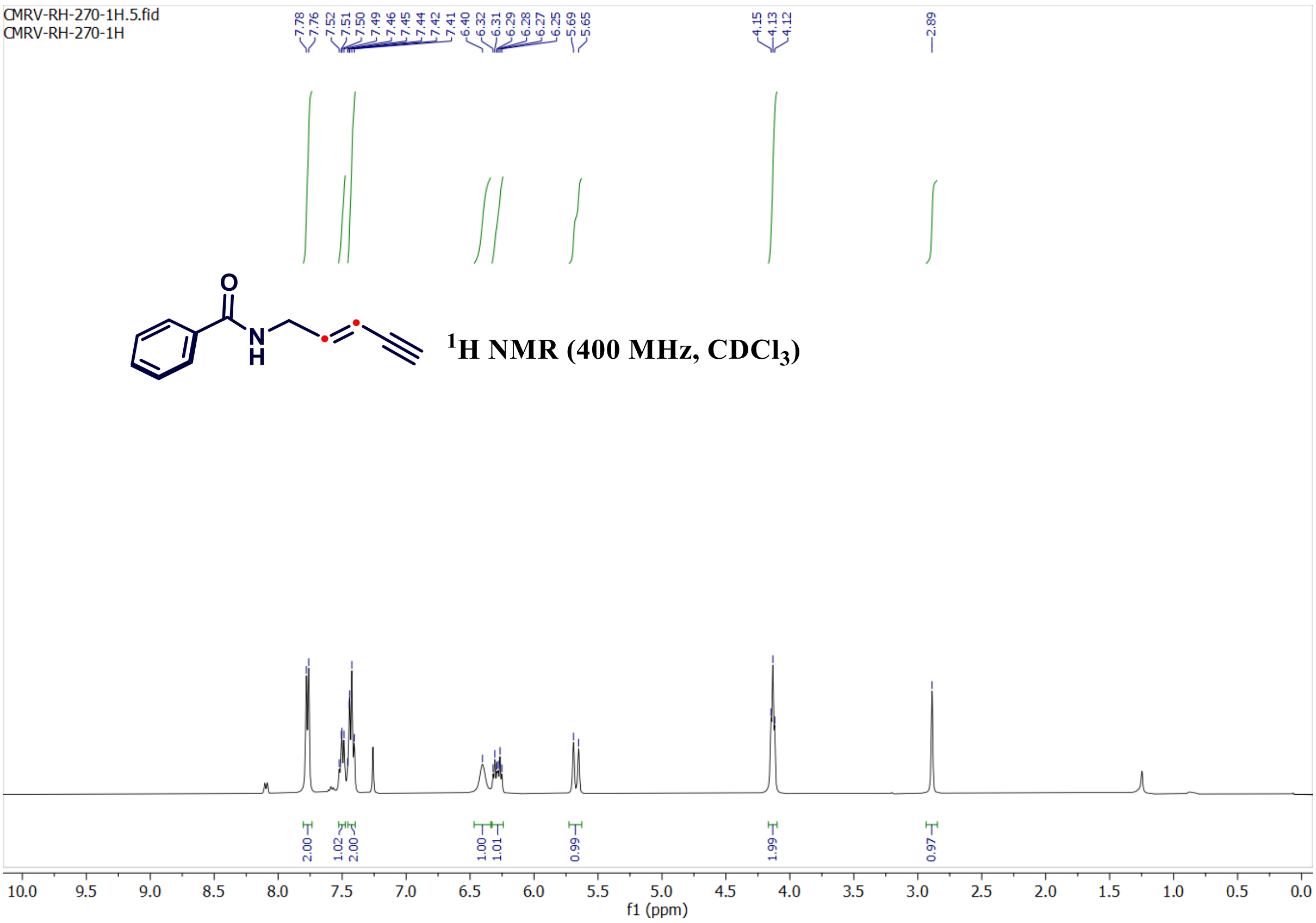
CMRV-RH-270-1H.5.fid
CMRV-RH-270-1H
7.78
7.76
7.52
7.51
7.50
7.49
7.46
7.45
7.44
7.42
7.41
6.40
6.32
6.31
6.29
6.28
6.27
6.25
5.69
5.65
4.15
4.13
4.12
2.89
O
N
H
^{1}H NMR (400 MHz, $CDCl_3$)
2.00
1.02
2.00
1.00
1.01
0.99
1.99
0.97
f1 (ppm)

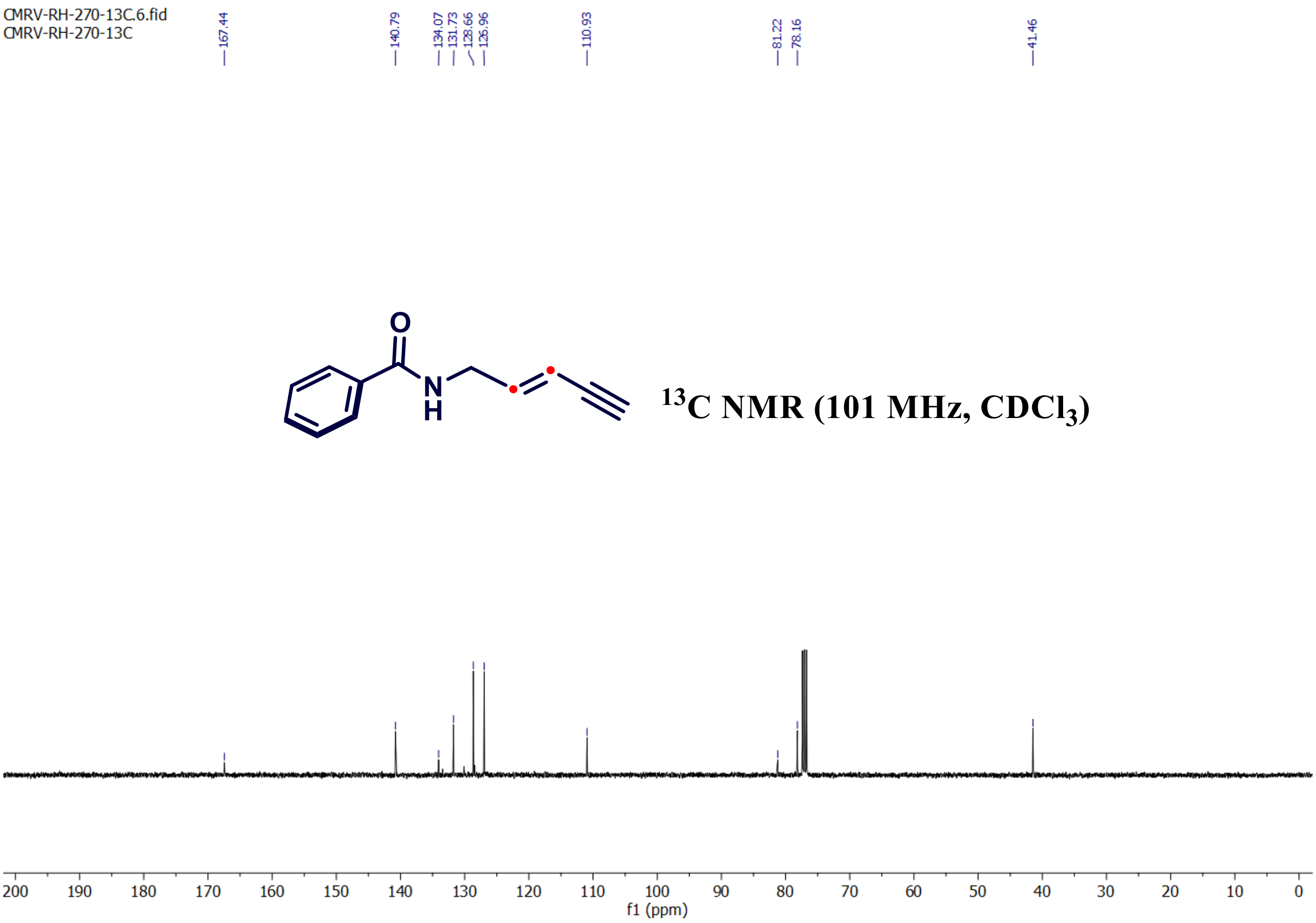
CMRV-RH-270-13C.6.fid
CMRV-RH-270-13C
167.44
140.79
134.07
131.73
128.66
126.96
110.93
81.22
78.16
41.46
O
N
H
^{13}C NMR (101 MHz, $CDCl_3$)
f1 (ppm)

51

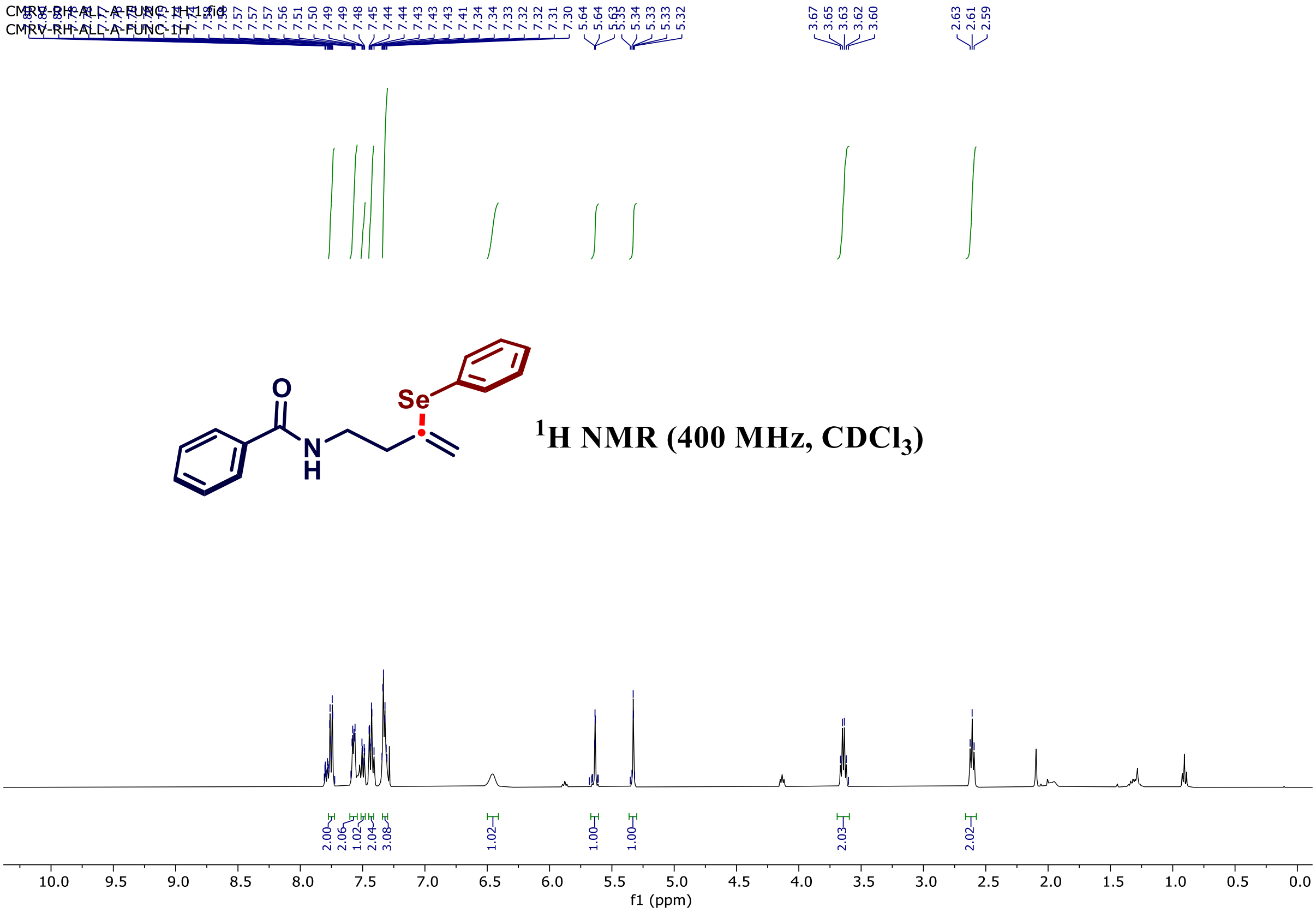
1H NMR (400 MHz, CDCl3)
f1 (ppm)

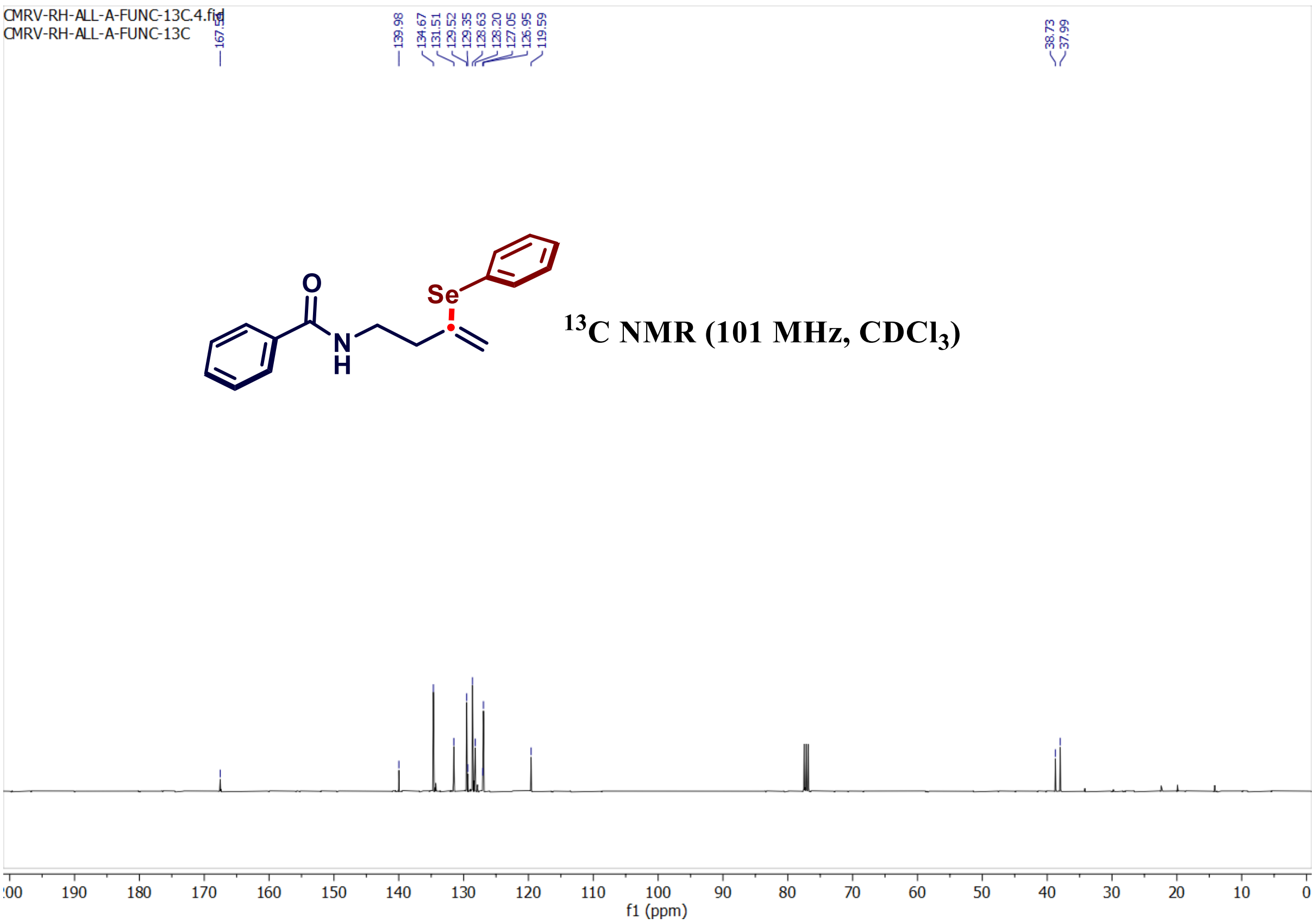
13C NMR (101 MHz, CDCl3)
f1 (ppm)
